# Supplementary material for: The impact of short term Antiretroviral Therapy (ART) interruptions on longer term maternal health outcomes—A randomized clinical trial
Source: PLoS One. 2020 Jan 30;15(1):e0228003. doi: 10.1371/journal.pone.0228003 (PMC6992010; doi:10.1371/journal.pone.0228003)
Supplement: S1 Data — (ZIP) [file pone.0228003.s001.zip › IMPAACT 1077BF_Protocol.pdf]

IMPAACT 1077BF

(DAIDS Document ID 10777)

BreastFeeding Version of the PROMISE Study (Promoting Maternal and Infant Survival Everywhere)

A Multicenter, International Trial of the
International Maternal Pediatric Adolescent AIDS
Clinical Trials Group (IMPAACT)

This file contains the current IMPAACT 1077BF protocol, which is comprised of the following documents, presented in reverse chronological order:

- **Letter of Amendment #5, dated 14 October 2015**
- **Letter of Amendment #4, dated 27 August 2014**
- **Clarification Memorandum #4, dated 4 August 2014 – ZIMBABWE SITES ONLY**
- **Letter of Amendment #3, dated 25 February 2014**
- **Clarification Memorandum #3, dated 5 December 2013**
- **Letter of Amendment #2, dated 30 September 2013 – ZIMBABWE SITES ONLY**
- **Letter of Amendment #1, dated 2 April 2013**
- **Clarification Memorandum #2, dated 22 March 2013**
- **Clarification Memorandum #1, dated 16 January 2013**
- **Protocol Version 3.0, dated 15 October 2012**

Letter of Amendment #5 for:

IMPAACT 1077BF
Breastfeeding Version of the PROMISE Study
(Promoting Maternal and Infant Survival Everywhere)
Version 3.0, dated 15 October 2012
IND # 107,507

DAIDS Document ID 10777

Letter of Amendment Date: 14 October 2015

Information/Instructions to Study Sites from the Division of AIDS

The information contained in this Letter of Amendment (LoA) impacts the IMPAACT 1077BF study and must be submitted to site Institutional Review Boards and/or Ethics Committees (IRBs/ECs) as soon as possible for their review and approval. The information contained in this LoA does not impact the sample informed consent forms for IMPAACT 1077BF. Nonetheless, IRB/EC approval is required prior to implementation of this LoA. Approval must also be obtained from site regulatory entities if applicable per the policies and procedures of the regulatory entities. All IRB/EC and regulatory entity requirements must be followed.

Upon obtaining IRB/EC approval and any other applicable regulatory entity approvals, each site should immediately begin implementing this LoA. Sites are required to submit a LoA registration packet to the DAIDS Protocol Registration Office (DAIDS PRO) at the Regulatory Support Center (RSC). Sites will receive a registration notification for the LoA after the DAIDS PRO verifies that all required registration documents have been received and are complete. Sites should not await this notification before implementing this LoA.

Please file this LoA, all associated IRB/EC and regulatory entity correspondence, and all correspondence with the DAIDS PRO in your essential documents files for IMPAACT 1077BF.

If the IMPAACT 1077BF protocol is amended in the future, the contents of this LoA will be incorporated into the next version of the protocol.

Summary of Modifications and Rationale

On 7 July 2015, 1077BF study sites received formal communications regarding the results of the Strategic Timing of Antiretroviral Treatment (START) study and associated changes to be implemented in 1077BF in response to these results. All sites were instructed that all women in 1077BF should be informed of the START study results and that antiretroviral therapy (ART) should be recommended for all women in 1077BF, based on the START study results.

Given this important change in maternal management, it has been further determined that protocol specifications for the duration of study participation should be modified such that follow-up is completed earlier than originally planned, at the end of September 2016.

The main purpose of this LoA is to implement the above-described modification of the duration of follow-up in the Antepartum, Postpartum, and Maternal Health Components of 1077BF. This LoA also clarifies expectations for infant HIV testing as the duration of study follow-up is completed, provides guidance for management of nevirapine (NVP) prophylaxis for infants in the Postpartum Component, and provides guidance for management of maternal ART regimens in the event of rash.

Implementation

1. Completion of Study Follow-up

Throughout the descriptions of the Antepartum, Postpartum, and Maternal Health Components in the protocol, reference is made to maternal follow-up continuing until 96 weeks after the last delivery in the Antepartum Component (for all women), and to infant follow-up continuing until 104 weeks of age (for each infant). Given that all women in all components are now recommended to initiate ART, all applicable protocol sections are modified to specify that:

- Maternal follow-up will continue per the Schedules of Evaluations (SoEs) in protocol Appendices IA and IC through September 2016. Between July and September 2016, women will complete final study visits according to the “Early D/C or End of Study” column of the SoEs.

- Infant follow-up will continue per the SoE in protocol Appendix IB through September 2016. Infants will remain in follow-up through Week 104 or through the date of a final study visit between July and September 2016, whichever comes first.

  Based on known dates of birth, all infants who do not reach Week 104 before July 2016 will reach Week 74 or a later study time point between July and September 2016. During this period, the relevant scheduled visit — Week 74, Week 86, Week 98, or Week 104 — should be conducted. In the event that an infant is scheduled for two visits between July and September 2016, both visits should ideally be conducted, with the second visit occurring before 30 September 2016. Importantly, all infants who have not reached Week 74 of follow-up prior to July 2016 should complete a final Week 74 visit between July and September 2016.

2. HIV Testing at Final Infant Study Visits

At final infant study visits, among other specified evaluations, HIV testing should be performed per the current SoE as follows:

- For infants who have <u>not</u> achieved complete cessation of breastfeeding: HIV nucleic acid testing should be performed at final Week 74, Week 86, Week 98, and Week 104 visits.

- For infants who have achieved complete cessation of breastfeeding: HIV antibody testing should be performed at final Week 74 and Week 98 visits. HIV antibody testing should also be performed at final Week 86 visits in the event that protocol-specified testing was missed at Week 74; likewise, antibody testing should be performed at final Week 104 visits in the event that protocol-specified testing was missed at Week 98.

The above-described approach will ensure that all infants complete at least 18 months of follow-up with appropriate ascertainment of HIV status prior to completion of follow-up.

3. Management of Infant NVP Prophylaxis

For infants on NVP prophylaxis in the Postpartum Component, if the mother initiates ART as recommended based on the START study results, or if the mother had previously initiated ART, NVP should be discontinued when the mother has been on ART for 12 weeks or when the mother's HIV viral load has been suppressed to undetectable levels (based on HIV RNA PCR testing performed per the maternal Schedule of Evaluations), whichever comes first. If the mother chooses not to initiate ART, infant NVP should be continued per current protocol specifications.

This approach minimizes any potential risks that may be associated with infant exposure to ARVs through direct ingestion and breastmilk ingestion. It also is consistent with the specifications of protocol Section 4.713, which state that infant NVP may be stopped for other medical reasons. In the expected rare circumstance that the above-listed guidance is considered not in the best interest of a mother-infant pair (e.g., in the setting of sustained high maternal viral load) or otherwise cannot be followed, please contact the 1077BF Clinical Management Committee (CMC) for further case-by-case guidance. Please also contact the CMC with any other questions or concerns related to management of any participants.

4. Toxicity Management for Women with Grade 1 or Grade 2 Rash

Protocol Appendix II, Toxicity Management, includes a table that provides guidance on toxicity management for rash. This table was originally intended to apply to both mothers and infants, with the general expectation that relatively few mothers would receive efavirenz (EFV) during study follow-up. Since that time, EFV-containing regimens have become commonly recommended for pregnant and postpartum women and — with the recommendation that all women in 1077BF initiate ART — it is now expected that many women will receive EFV in the last year of study follow-up. Given this expectation, the original protocol specifications for management of rash have been reviewed and are now updated for women on EFV. Specifically,

women on EFV who experience a Grade 1 or Grade 2 rash are permitted to remain on EFV while awaiting the results of ALT testing. If no ALT elevation, fever, or other sign of systemic toxicity is identified, these women may continue EFV with close monitoring and instruction to return to the study site for further evaluation in the event of any worsening of their rash or development of any other signs of systemic toxicity. The CMC should also be contacted for further guidance on a case-by-case basis.

Letter of Amendment #4 for:

IMPAACT 1077BF
Breastfeeding Version of the PROMISE Study
(Promoting Maternal and Infant Survival Everywhere)
Version 3.0, dated 15 October 2012
IND # 107,507

DAIDS Document ID 10777

Letter of Amendment Date: 27 August 2014

Information/Instructions to Study Sites from the Division of AIDS

The information contained in this Letter of Amendment (LoA) impacts the IMPAACT 1077BF study, including the informed consent form (ICF) for the Postpartum Component (1077BP), and must be submitted to site Institutional Review Boards and/or Ethics Committees (IRBs/ECs) as soon as possible for their review and approval. Approval must also be obtained from site regulatory entities if applicable per the policies and procedures of the regulatory entities. All IRB/EC and regulatory entity requirements must be followed.

Upon obtaining IRB/EC approval and any other applicable regulatory entity approvals, each site should immediately begin implementing this LoA and using the updated ICF for 1077BP and, at sites participating in the Hair Substudy, the updated ICF for Specimen Storage and Future Testing. After all required approvals are obtained, the updated 1077BP ICF should be used to re-consent mother-infants pairs who are currently receiving a randomized study drug regimen in 1077BP and the updated Specimen Storage and Future Use ICF should be used at sites participating in the Hair Substudy for participants enrolled to the Hair Substudy. Re-consenting should take place at each applicable participant's next study visit after all required approvals are obtained. Unless otherwise directed by site IRBs/ECs, re-consenting is not required for any other study participants.

Sites are required to submit a LoA registration packet to the DAIDS Protocol Registration Office (DAIDS PRO) at the Regulatory Support Center (RSC). Sites will receive a registration notification for the LoA after the DAIDS PRO verifies that all required registration documents have been received and are complete. Sites should not await this notification before implementing this LoA.

Please file this LoA, all associated IRB/EC and regulatory entity correspondence, and all correspondence with the DAIDS PRO in your essential documents files for IMPAACT 1077BF.

If the IMPAACT 1077BF protocol is amended in the future, the contents of this LoA will be incorporated into the next version of the protocol.

Summary of Modifications and Rationale

Accrual to the Antepartum Component (1077BA) of 1077BF is nearing completion of the original target sample size of 3400 mother-infant pairs. Due to enrollment challenges in other study components, the protocol team has concluded that it will not be possible to achieve the original protocol-specified sample sizes for the Postpartum (1077BP) and Maternal Health (1077BM) Components in a reasonable time and has determined that enrollment into all components will stop enrollment on or before 1 October 2014. There were no concerns with participant safety or quality of study conduct associated with this decision. In addition, adequate statistical power can still be achieved to provide answers to many of the critical research questions that the study was designed to address.

The purpose of the LoA is to:

- Specify the timing of closure of enrollment into each study component
- Extend the duration of study drug regimens in the Postpartum Component (1077BP)
- Permit maternal use of study drug during observational follow-up in 1077BA

This LoA also incorporates the following modifications:

- The addition of a 100 mg capsule formulation of zidovudine, which was previously addressed in Clarification Memorandum #3, dated 5 December 2013.
- The addition of hair to the list of specimens indicated in the Consent Form for Specimen Storage and Future Use. Hair is being collected at select sites, as a biomarker of maternal and infant adherence and exposure to ARVs; addition of hair to the specimen storage and future use consent will allow for the storage and future use of hair samples leftover after study-specific testing has been completed. Re-consenting is required for women enrolled to the Hair Substudy; however, women can refuse to have specimens stored for future use, which will not affect their participation in PROMISE or in the Hair Substudy.

I. Closure of Enrollment

a. Late Presenters Registration (1077BL)
Accrual into Late Presenter Registration (1077BL) and enrollment screens for 1077BL were closed on 18 July 2014 due to low number of participants. Mother-infant pairs registered in 1077BL who do not enter 1077BP will complete six weeks of follow-up in 1077BL, per protocol.

b. Antepartum, Postpartum, and Maternal Health Components (1077BA, 1077BP, 1077BM)
Accrual into these components will be closed when the target of 3400 mother-infant pairs in 1077BA is reached or at 5:00 PM Eastern Daylight Time, on 1 October 2014, *whichever comes first.* The enrollment screens for 1077BA, 1077BP, and 1077BM will close when this time point is reached.

II. Duration of Study Drug Regimen in 1077BP

Once accrual into 1077BP is closed, mother-infant pairs randomized in the Postpartum Component will continue follow-up per the current Schedule of Evaluations. However, mothers randomized to Arm A will continue to receive a triple ARV regimen, through the end of maternal follow-up (96 weeks after the last infant enrolled in 1077BA is delivered). Those who require regimen changes for their own health or regimen changes for failure or toxicity will follow the protocol. Infants randomized to Arm B will receive nevirapine (or 3TC) through cessation of risk for MTCT or completion of follow-up (104 weeks), whichever comes first.

III. Use of study drug during observational follow-up in 1077BA

The protocol-specified duration of the randomized maternal study drug regimens in 1077BA ends at Week 1 (Day 6-14) postpartum. Thereafter, unless they have initiated HAART for their own health, mothers who have not undergone any further randomization and who remain in observational follow-up in 1077BA are referred to the (non-study) standard of care for postpartum women in their country, plus infant NVP through 6 weeks of age. In the event that these women require ARVs but in consultation with their clinician prefer to stay on study drug triple ARVs, study drug may be provided to them.

IV. Follow-Up of All Participants

Follow-up of all enrolled participants will continue for the durations specified in the protocol (per the Schedule of Evaluations). Infants will be followed through 104 weeks (two years) of age; mothers will be followed through 96 weeks after the last infant enrolled in the Antepartum Component is delivered. Although the date of the last infant birth cannot be predicted, the last enrollment in 1077BA is expected by 1 October 2014. The last birth would then be expected within six months after the last enrollment (e.g., by 30 April 2015). Therefore, all maternal and infant follow-up is expected to end by 30 April 2017.

V. Informing Participants

Sites should inform participants of these protocol decisions as some participants will be directly impacted because they will not be randomized to subsequent components. Sites should follow guidance from their IRB/EC regarding how best to inform participants of these changes.

VI. Statistical Implications

The protocol statisticians have assessed the impact these protocol changes may have on the statistical power to detect the differences in efficacy outcome measures between randomized arms that were originally specified in the protocol for each PROMISE component, based on the event rate assumptions specified in the protocol. With the lower than projected accrual of late presenting and formula-feeding mother-infant pairs, the study will have less than 90% power to detect the hypothesized efficacy differences between randomized arms.

If the study participants continue on-study for the originally planned duration of follow-up and if the Postpartum and Maternal Health Component participants remain on their randomized assignments, the study is expected to have adequate (at least 80%) power to detect the hypothesized differences or somewhat larger differences, as follows:

- The Antepartum Component is projected to have adequate power to detect the hypothesized difference in perinatal HIV transmission between randomized arms among breastfeeding women (4% versus 2%).
- The Postpartum Component is projected to have adequate power to detect a difference in breastfeeding HIV transmission between randomized arms that is larger than originally hypothesized (6% versus 3.2% after an average of 15 months of breastfeeding).
- Two of the four primary Maternal Health Comparisons (Comparison 1a: triple ARVs only during pregnancy versus no triple ARVs and Comparison 2a: continuing versus stopping triple ARVs after delivery) are expected to have adequate power to detect the hypothesized differences in rates of maternal progression to AIDS or death (3 year cumulative event rate of 10% versus 6.1%). The other two Maternal Health Comparisons (Comparison 1b: triple ARVs only during breastfeeding versus no triple ARVs and Comparison 2b: continuing versus stopping triple ARVs after breastfeeding cessation) are expected to have inadequate power to detect the hypothesized differences in maternal progression to AIDS or death.

Implementation

Protocol modifications are shown below, using strikethrough for deletions and bold type for additions.

Antepartum Component

1. Section 2.61, the following note is added:

   Note: Women who have completed the randomized 1077BA regimen (up to 14 days post-delivery) should be referred for standard of care for postpartum women in their country. Upon implementation of LoA #4 and after closure to enrollment to subsequent components 1077BP and 1077BM, study-supplied study drug may be provided, through completion of follow-up, to these women if not available locally as standard of care or if a woman and her clinician decide that continuing the study drug regimen would be in her best interest.

Postpartum Component

The following modifications apply only to mother-infant pairs on a randomized study drug regimen in 1077BP:

2. Schema, Design:

   Arm A: Maternal triple ARV prophylaxis through **completion of follow-up** ~~BF cessation or through 18 months postpartum, whichever comes first~~ (with infant NVP prophylaxis through six weeks of age)

   Arm B: Infant NVP prophylaxis through ~~BF~~ cessation **of risk of MTCT** or **completion of follow-up** ~~through 18 months postpartum~~, whichever comes first (with no maternal prophylaxis)

3. Schema, Sample Size:

   The accrual target is approximately, **2,500** ~~4,650~~ mother-infant pairs (all eligible BF mother-infant pairs who were randomized in the Antepartum Component plus up to approximately 1,550 BF late presenters)

4. Schema, Treatment Regimen:

   Arm A (maternal prophylaxis): Maternal triple ARV prophylaxis given from 6 (up to 14) days postpartum through **completion of follow-up** ~~BF cessation or through 18 months postpartum, whichever comes first~~ unless stopped for toxicity~~,~~ **or** other medical reasons ~~or confirmed infant HIV infection (of all the woman's study infants, if multiple births)~~ plus infant NVP prophylaxis through six weeks (42 days) of age (continued from the Antepartum Component study drug regimen). The study-supplied maternal triple ARV regimen is LPV-RTV plus TRV (fixed dose combination FTC-TDF).

   Arm B (infant prophylaxis): Infant NVP prophylaxis given once daily given from 6 (up to 14) days of age (continued from the Antepartum Component study drug regimen) through ~~BF~~ cessation **of risk for MTCT** or **completion of follow-up** ~~through 18 months postpartum~~, whichever comes first unless stopped for infant HIV infection, toxicity, or other medical reasons.

5. Schema, Figure 2:

   Figure Box for Maternal Triple ARV Prophylaxis

   through **completion of follow-up** ~~BF cessation or 18 mos postpartum, whichever comes first~~ plus Infant NVP through 6 weeks of age

   Figure Box for Infant NVP Prophylaxis

   through ~~BF~~ cessation **of risk for MTCT** or **completion of follow-up** ~~18 mos postpartum~~, whichever comes first

6. Section 4.15, Maternal Postpartum Triple ARV Prophylaxis Regimen, first paragraph:

   ZDV is a preferred nucleoside analogue drug for use as part of a triple ARV regimen when used solely for prevention of MTCT during pregnancy because of extensive safety experience. However, in the Postpartum Component of PROMISE, women randomized to receive the triple ARV regimen to prevent postnatal transmission could receive ~~as long as 18 months of the~~ study drugs **for several years**. 3TC-ZDV is associated with bone marrow suppression, manifested by macrocytic anemia and neutropenia, and this may be exacerbated in resource-limited settings where baseline levels of maternal anemia may be high.

7. Section 4.32, 1077BP Step 1:

   Arm A: Maternal triple ARV prophylaxis (with infant NVP prophylaxis through six weeks of age) given through **completion of follow-up** ~~BF cessation or through 18 months postpartum, whichever comes first~~ unless stopped for toxicity~~,~~ **or** other medical reasons ~~or confirmed infant HIV infection (of all of the woman's study infants)~~. The study-supplied triple ARV prophylaxis regimen is LPV-RTV plus TRV (fixed dose combination FTC-TDF).

   Arm B: Infant NVP prophylaxis (no maternal prophylaxis) given once daily through ~~BF~~ cessation **of risk for MTCT** or **completion of follow-up** ~~through 18 months postpartum~~, whichever comes first, unless stopped for toxicity~~,~~ **or** other medical reasons or HIV infection. (See Section 4.612 for additional information on dosing continuation.)

8. Section 4.4, Counseling on HIV and Infant Feeding (Postpartum Component), second paragraph:

   Exclusive BF (EBF) is defined as an infant's consumption of human milk without supplementation of any type (no water, no juice, no non-human milk and no foods) except for vitamins, minerals and medications. It is anticipated that almost all mothers in the study will have stopped breastfeeding by 18 months and also that the benefits of breastfeeding in terms of child survival are minimized by that age. ~~Unless they are assured access to ARV prophylaxis outside of the study as standard of care, study mothers who are still breastfeeding at 18 months postpartum will be advised to stop breastfeeding at that time given that the postpartum study interventions (triple ARV maternal prophylaxis or infant NVP prophylaxis) will not be provided after 18 months.~~ Women will also be counseled against pre-mastication (pre-chewing food by the mother or caregiver) of any foods to be given to infants in view of hygiene and reports of HIV transmission through premastication.

9. Section 4.6, Study Treatment (Postpartum Component), Arm A, Mothers:

- Emtricitabine-Tenofovir disoproxil fumarate fixed dose combination tablet 200 mg/300 mg orally daily from the week 1 postpartum visit until **completion of follow-up** unless stopped for toxicity~~,~~ or other medical reasons. ~~up to eight weeks after complete BF cessation is achieved (defined as completely stopping all exposure to breast milk for > 28 days), i.e., up to 84 days after last exposure to breast milk, 18 months postpartum, or entry into the Maternal Health component, whichever comes first unless stopped for toxicity,~~ ~~**or**~~ ~~other medical reasons or confirmed infant HIV infection (of all the mother's infants). Note that a mother with multiple infants (e.g., twins) will not discontinue her study treatment for the criterion of infant HIV infection if at least one of her study infants remains uninfected.~~

- Lopinavir/ritonavir fixed dose combination tablet 200 mg/50 mg x 2 tablets for a single dose of 400 mg/100 mg and a total daily dose of 800 mg/200 mg orally twice daily from week 1 postpartum visit until **completion of follow-up** unless stopped for toxicity or other medical reason. ~~up to eight weeks after complete BF cessation is achieved (defined as completely stopping all exposure to breast milk for > 28 days), i.e., up to 84 days after last exposure to breast milk, 18 months postpartum, or entry into the Maternal Health component, whichever comes first unless stopped for toxicity,~~ ~~**or**~~ ~~other medical reasons or confirmed infant HIV infection (of all the mother's infants). Note that a mother with multiple infants (e.g., twins) will not discontinue her study treatment for the criterion of infant HIV infection if at least one of her study infants remains uninfected.~~

10. Section 4.6, Study Treatment (Postpartum Component), Arm B, Infants:

- Nevirapine age-based daily dosing (see Section 4.612, Table 1) starting from the week 1 postpartum visit (day 6-14) (as a continuation of the daily dosing regimen begun in the AP Component or LP Registration) and continuing for up to two weeks after complete BF cessation is achieved (defined as completely stopping all exposure to breast milk for > 28 days; i.e., for up to 42 days after last exposure to breast milk) or **completion of follow-up** ~~18 months postpartum~~, whichever comes first, unless stopped for infant HIV infection, toxicity or other medical reasons. If a mother has multiple infants, such as twins, an infant with HIV infection must stop study NVP, but the other infant(s) can continue study drug. Infants who discontinue NVP due to toxicity/intolerability will be provided an alternative regimen of lamivudine (3TC). See Section 4.612 for additional information on continuation of study drug dosing and the alternative regimen.

11. Section 4.612, Duration:

Arm A (Maternal Prophylaxis Arm): Cessation of BF is defined as completely stopping all exposure to breast milk for > 28 days, based on maternal report. The study drugs for women will be continued until **completion of follow-up** unless stopped for toxicity or other medical reason. ~~up to eight weeks after complete BF cessation as defined above is achieved (i.e., up to 84 days after last exposure to breast milk) or 18 months postpartum (week 72-76 postpartum*), whichever comes first, unless stopped for confirmed infant HIV infection (of all the woman's study infants), toxicity, or other medical reasons. Note that a mother with multiple infants (e.g., twins) will not discontinue her study treatment for the criterion of confirmed infant HIV infection if at least one of her study infants remains uninfected.~~

~~*Note: for women who are still breastfeeding at 18 months postpartum and potentially eligible for the Maternal Health Component, the triple ARV regimen should be continued while eligibility for the Maternal Health Component is determined (until either enrolled or determined to be ineligible or unwilling), with continued dosing permitted through week 78 postpartum.~~

Arm B (Infant Prophylaxis Arm): After the first six weeks of age, the study drug for infants (NVP) will be continued for up to two weeks after complete BF cessation as defined above is achieved (i.e., up to 42 days after last exposure to breast milk) or **to completion of follow-up** ~~to 18 months postpartum (week 72-76), whichever comes first~~, unless stopped for infant HIV infection ~~(of all the woman's study infants)~~, toxicity, or other medical reasons. If an infant in Arm B must discontinue NVP due to toxicity/intolerability, he or she will be provided lamivudine (3TC) as an alternative study drug (see regimen below).

12. Section 4.612, Table 1, Once Daily Nevirapine Prophylaxis Doses for Infants, last row:

Infant Age	Total Daily Dose (mg)	Volume of Nevirapine Suspension to Administer (mL)
> 9 Months to Cessation of **risk for MTCT or completion of follow-up** ~~BF or 18 months~~, whichever is first	40mg	4.0 mL

13. Section 4.711, 1077BP STEP 1, Chart:

Chart Box for Infants Arm B

Infant NVP prophylaxis **through cessation of risk for MTCT or completion of follow-up, whichever comes first** ~~for up to 18 months while BF~~

14. Section 4.712, Maternal Triple ARV Prophylaxis Randomization (with Infant Prophylaxis for 6 Weeks), second paragraph:

Women randomized to triple ARV prophylaxis in the Postpartum Component will continue study drug **through completion of follow-up** unless study drug is stopped for toxicity or other medical reason. ~~dosing through complete cessation of BF (as defined above) or through 18 months postpartum (week 72-76 postpartum), whichever comes first, and until randomization into the Maternal Health Component (if eligible and willing; see Section 5.3) unless study drug is stopped early for toxicity, or other medical reasons or confirmed infant HIV infection (of all the mother's infants). The window for the Maternal Health randomization is up to eight weeks after complete BF cessation as defined above is achieved with no plans to re-initiate BF with current infant.~~ See Section 4.715 for management of infants with positive HIV NAT after randomization (and their mothers).

15. Section 4.713, Infant NVP Prophylaxis Randomization (no Maternal Prophylaxis), first paragraph:

    Infants of mother-infant pairs randomized to the infant NVP prophylaxis arm in 1077BP Step 1 will continue the daily infant NVP regimen begun as part of the AP Component or Later Presenter Registration; continuation of the regimen from Postpartum Component entry (Week 1 visit) at 6-14 days after delivery will be considered part of the PP Component study drug regimen. Randomized infants will follow the infant schedule of evaluations listed in Appendix IB. Infants should continue on their study drug until up to two weeks after complete BF cessation as defined above is achieved or through **completion of follow-up** ~~18 months of age (week 72-76)~~, whichever comes first, unless NVP is stopped for toxicity, other medical reasons or for HIV infection.

16. Section 4.715, Management of Infants with a Positive HIV Test after Randomization (and their Mothers), second and third paragraphs:

    Note that if a mother has multiple infants, such as twins, the infant with HIV infection must stop study drug, but the other infant(s) can continue the study drug. If the mother of multiple infants had been randomized to maternal triple ARV prophylaxis in Postpartum Component 1077BP Step 1, and one infant has HIV infection but the other infant(s) is uninfected, the maternal triple ARV prophylaxis regimen should be continued **through completion of follow-up** ~~until cessation of breastfeeding of her uninfected infant(s) or 18 months, whichever comes first~~.

    Mothers of infants with confirmed HIV infection who were randomized to the maternal triple ARV prophylaxis arm ~~are eligible for randomization into the Maternal Health Component following confirmation of the positive infant HIV test (of all their infants if multiple births), and should continue their triple ARV prophylaxis regimen until randomization into the Maternal Health Component to continue or stop their triple ARV prophylaxis regimen (see Section 5.4). Mothers who are ineligible for the Maternal Health Component or refuse participation will be eligible to receive the standard of care for postpartum women in their country and~~ continue follow-up as per Appendix IA **and will continue to receive study drug through completion of follow-up**.

17. Section 4.72, Management including ARV Management of Women and Infant in Postpartum Component, second paragraph:

    Infants of mother-infant pairs randomized to either arm in the Postpartum Component whose mothers require a triple ARV regimen for their own health (enter 1077BP Step 2 or Step 3) should continue their study NVP as long as they meet the protocol-specified criteria (breastfeeding, HIV-uninfected ~~and < 18 months of age~~).

18. Section 4.75, Criteria for Study Drug Discontinuation:

    Women or infants may be discontinued from study treatment temporarily or permanently primarily based on toxicity events and tolerability issues. The DAIDS Table for Grading the Severity of Adult and Pediatric Adverse Events, Version 1.0, dated December 2004 (with Clarification dated August 2009) and the Toxicity Management Guidelines (Appendix II) will be used to guide these decisions, in consultation with the CMC when required and/or when desired by the site investigator). Women and infants who are removed from treatment will be continued to be followed on study (off study drug/on study) and follow the relevant maternal or infant schedule of evaluations. The randomized treatment regimen is given **for the duration specified in Section 4.612** ~~through BF cessation or through 18 months postpartum, whichever comes first, unless stopped for toxicity, other medical reasons or confirmed infant HIV infection (of all the woman's infants)~~.

Study drugs may be discontinued for any of the following reasons:

- Drug-related toxicity (see Toxicity Management - Appendix II)
- Requirement for prohibited concomitant medications
- Clinical reasons believed life threatening by the site investigator, even if not addressed in the Toxicity Management Guidelines (Appendix II)
- Second virologic failure with CD4 $\geq$ 350 cells/mm^3
- Request of the primary care provider if s/he thinks the study treatment is no longer in the best interest of the participant
- Request of the participant
- Documented HIV infection in the infant (if randomized to maternal triple ARV prophylaxis during breastfeeding, mother **will remain on study drug through completion of follow-up** ~~may remain eligible for randomization into Maternal Health Component, see Section 5.3~~)*

Any dispensed study drug remaining after discontinuation must be collected.

* Note: In the case of a multiple birth (e.g., twins), ~~a mother assigned to the maternal the triple ARV prophylaxis arm will not discontinue her study treatment for the criterion of confirmed HIV infant infection if at least one of her study infants remains uninfected;~~ if infants were assigned to infant NVP arm, an infant with confirmed HIV infection must stop study NVP, but if the other infant(s) is uninfected, he/she can continue the study NVP. If infection of an infant cannot be ruled out following an initial positive HIV test, study drug should be permanently discontinued.

Note: Early discontinuation of study product for any reason is not a reason for withdrawal from the study.

19. Section 4.10, Sample Informed Consent Form — Postpartum Component

WHY IS THE PROMISE STUDY BEING DONE

Second paragraph:

This is a consent form to join the Postpartum Part of the PROMISE Study. ~~We will give you detailed information about the Maternal Health Part at a later time, but you are free to ask questions now if you would like~~.

Figure box for Baby Study Drug Group:

The anti-HIV medication is given to the baby throughout the time of breastfeeding or **until completion of the study at 2 years of age** ~~for 18 months after birth~~, whichever comes first

Figure box for Mother Study Drug Group:

The anti-HIV medications are given to the mother throughout the time of breastfeeding **and continuing after that until completion of the study** ~~or for 18 months after delivery, whichever comes first~~ with anti-HIV medication given to the baby through 6 weeks of age

WHAT WILL MY BABY AND I HAVE TO DO IF WE JOIN THIS STUDY, Screening/First Visit, first paragraph:

If you decide that you want to join the Postpartum Part with your baby, we will need to confirm that you and your baby are eligible. ~~If you or your baby is not eligible for the Postpartum Part, we will talk about whether you may be eligible for the Maternal Health Part of the PROMISE Study.~~

HOW MANY WOMEN AND CHILDREN WILL TAKE PART IN THE PROMISE STUDY?

About ~~4650~~ **2500** women and their babies will take part in this postpartum part of the PROMISE study around the world.

WHAT ARE THE RISKS OF THE STUDY?, Side Effects of Anti-HIV Medicines for Babies, second paragraph:

Some babies will continue taking nevirapine **until completion of the study at 2 years of age** ~~for up to 18 months~~ while breastfeeding.

20. Section 6.0, STATISTICAL CONSIDERATIONS FOR PROMISE, after first paragraph:

NOTE: There has been an assessment of the impact that the modifications specified in Letter of Amendment #4 may have on the statistical power to detect the differences in efficacy outcome measures between randomized arms that were originally specified in the protocol for each PROMISE component, based on the event rate assumptions specified in the protocol. With the lower than projected accrual of late presenting and formula-feeding mother-infant pairs, the study will have less than 90% power to detect the hypothesized efficacy differences between randomized arms.

If the study participants continue on-study for the originally planned duration of follow-up and if the Postpartum and Maternal Health Component participants remain on their randomized assignments, the study is expected to have adequate (at least 80%) power to detect the hypothesized differences or somewhat larger differences, as follows:

- **The Antepartum Component is projected to have adequate power to detect the hypothesized difference in perinatal HIV transmission between randomized arms among breastfeeding women (4% versus 2%).**
- **The Postpartum Component is projected to have adequate power to detect a difference in breastfeeding HIV transmission between randomized arms that is larger than originally hypothesized (6% versus 3.2% after an average of 15 months of breastfeeding).**
- **Two of the four primary Maternal Health Comparisons (Comparison 1a: triple ARVs only during pregnancy versus no triple ARVs and Comparison 2a: continuing versus stopping triple ARVs after delivery) are expected to have adequate power to detect the hypothesized differences in rates of maternal progression to AIDS or death (3 year cumulative event rate of 10% versus 6.1%). The other two Maternal Health Comparisons (Comparison 1b: triple ARVs only during breastfeeding versus no triple ARVs and Comparison 2b: continuing versus stopping triple ARVs after breastfeeding cessation) are expected to have inadequate power to detect the hypothesized differences in maternal progression to AIDS or death.**

Antepartum, Postpartum and Maternal Health Components

21. Sections 2.516, 4.61, and 5.414

Generic Name Abbreviation Trade Name	Formulation	Appearance	Storage
Zidovudine<br>ZDV<br>Retrovir®	300 mg tablets	Biconvex, white, round, film-coated tablets	15-25°C (59-77°F)
	**100 mg capsules**	**White, opaque cap and body with "Wellcome" and unicorn logo on cap and "Y9C" and "100" on body**	**15-25°C (59-77°F) and protected from moisture**

22. Appendix VI, Sample Informed Consent for Specimen Storage and Future Use

Introduction

You have decided that you and your baby will participate in this research study to help us find the best ways to prevent babies from getting HIV and to keep mothers and babies healthy. In addition to the tests that you have as part of the study, we are asking now for your permission to save any leftover blood, breast milk**,** ~~and~~ cells ***and [For sites participating in the Hair Substudy: hair]*** from your blood and any of your baby's leftover blood, ~~and~~ cells ***and [For sites participating in the Hair Substudy: hair]*** for future use. These specimens would be saved in a place called a repository, which is a special laboratory with freezers to store the specimens. There are no names on any of the specimens, only a special study number. The people who run the repository and the scientists who later use the specimens will not know your name or your child's name.

<u>How often will these specimens be collected</u>?
As described to you when you agreed to join the study, blood, ~~and~~ breast milk***, and [For sites participating in the Hair Substudy: hair]*** will be collected for study tests at each study visit. After all testing that is planned to be done for the study has been completed, some of your and your baby's blood ***and [For sites participating in the Hair Substudy: hair]***, your breast milk and cells from your or your baby's blood may be leftover. It is these leftover specimens that you are being asked to have stored for future use. You are not being asked to give additional specimens for long term storage.

Clarification Memorandum #4 for:

Site-Specific for CRSs 30303, 30306, 30313 for:

IMPAACT 1077BF
Breastfeeding Version of the PROMISE Study
(Promoting Maternal and Infant Survival Everywhere)
Version 3.0, dated 15 October 2012
IND # 107,507

DAIDS Document ID 10777

Clarification Memorandum Date: 4 August 2014

Information/Instructions to Study Sites

This Clarification Memorandum (CM) is specific to the following Clinical Research Sites (CRSs) located in Zimbabwe: St Mary's Clinic, Chitungwiza (30303); Seke North Clinic, Chitungwiza (30306); and Parirenyatwa Clinic, Harare (30313). This CM is not applicable to any other study sites.

This CM has been approved by the NIAID Medical Officers. Institutional Review Board/Ethics Committee (IRB/EC) approval of this CM is not required by the sponsor prior to implementation; however, sites may submit it to the responsible IRBs/ECs for their information or, if required by the IRBs/ECs, for their approval prior to implementation. None of the clarifications being made impact the sample informed consent forms and the benefit-to-risk ratio for participants is not affected in any way.

This CM should be maintained in each site's essential documents file for IMPAACT 1077BF. It is the responsibility of the Investigator of Record to ensure that all study staff at the sites listed above are made aware of and follow this CM.

Summary of Clarification and Implementation

This CM clarifies that, for women in the Antepartum Component (1077BA) at CRSs 30303, 30306, and 30313, if nevirapine is unavailable from the DAIDS Clinical Research Product Management Center (CRPMC), locally available nevirapine may be supplied. Additions to the text appear in bold below.

Section 2.511, following the bullet points in Sub-section 2.511.3
At enrollment/randomization, it is expected that the assigned maternal and infant regimens (listed above and below, respectively) will use study-supplied study drugs. **Note: For mothers randomized to Step 1 Arm A, locally-supplied nevirapine tablets may be used in the event that study-supplied nevirapine tablets are not available from the CRPMC.** Thereafter, if one or more of the assigned study-supplied study drugs cannot be tolerated, the regimen may be modified (in consultation with the CMC if required per Appendix II) using study-supplied study drugs (see listing in Section 2.516) and/or drugs from other sources.

Section 2.516
In the listing of study-supplied study drugs, nevirapine tablets is listed. However, the Zimbabwe sites may use non-study supplied nevirapine tablets when they are not available from the CRPMC.

Section 7.23
Expedited Adverse Event Report is required for mothers receiving locally-supplied nevirapine tablets in the Antepartum Component (1077BA).

Letter of Amendment #3 for:

IMPAACT 1077BF
Breastfeeding Version of the PROMISE Study
(Promoting Maternal and Infant Survival Everywhere)
Version 3.0, dated 15 October 2012
IND # 107,507

DAIDS Document ID 10777

Letter of Amendment Date: 25 February 2014

Information/Instructions to Study Sites from the Division of AIDS

The information contained in this Letter of Amendment (LoA) impacts the IMPAACT 1077BF study and must be submitted to site Institutional Review Boards (IRBs) and/or Ethics Committees (ECs) as soon as possible for their review and approval. The information contained in this LoA does not impact the sample informed consent forms for IMPAACT 1077BF. Nonetheless, IRB/EC approval is required prior to implementation of this LoA. Approval must also be obtained from site regulatory entities if applicable per the policies and procedures of the regulatory entities. All IRB/EC and regulatory entity requirements must be followed.

Upon receiving IRB/EC approval and approval of any other applicable regulatory entities, this LoA is to be implemented immediately. Sites are still required to submit a LoA registration packet to the DAIDS Protocol Registration Office (DAIDS PRO) at the Regulatory Support Center (RSC). Sites will receive a registration notification for the LoA after the DAIDS PRO verifies that all required registration documents have been received and are complete. Sites should not await this notification before implementing this LoA.

Please file this LoA, all associated IRB/EC and regulatory entity correspondence, and all correspondence with the DAIDS PRO in your essential documents files for IMPAACT 1077BF.

If the IMPAACT 1077BF protocol is amended in the future, the contents of this LoA will be incorporated into the next version of the protocol.

Summary of Modifications

1.) Extends the timeframe for entry into the Maternal Health Component (1077BM) from the Postpartum Component (1077BP) after complete cessation of breastfeeding is achieved to within 29-84 days (from 29 – 42 days) after last infant exposure to breastmilk. (Sections 4.6, 4.612, 4.712, 5.21, 5.311.2, and 5.511 are updated.)
2.) Modifies the definition of exclusionary interruption of triple ARV prophylaxis prior to entry into 1077BM from 1077BP to missing more than 14 consecutive days (from 7 consecutive days) of dosing. (Section 5.311.1 is updated.)
3.) Broadens the protocol-specified expedited reporting requirements to include ALL infant deaths occurring within the infant follow-up period. (Section 7.23 is updated.)

Implementation

Modifications are shown below, using strikethrough for deletions and bold type for additions.

Section 4.6 Study Treatment (Postpartum Component), 2nd paragraph

At entry to 1077BP Step 1 subjects will be randomized to one of two arms:

Arm A:

Mothers:

- Emtricitabine-Tenofovir disoproxil fumarate fixed dose combination tablet 200 mg/300 mg orally daily from the week 1 postpartum visit until up to ~~two~~ **eight** weeks after complete BF cessation is achieved (defined as completely stopping all exposure to breast milk for ≥ 28 days), i.e. up to ~~42~~ **84** days after last exposure to breast milk, 18 months postpartum, or entry into the Maternal Health component, whichever comes first unless stopped for toxicity, other medical reasons or confirmed infant HIV infection (of all the mother's infants). Note that a mother with multiple infants (e.g., twins) will not discontinue her study treatment for the criterion of infant HIV infection if at least one of her study infants remains uninfected.

- Lopinavir/ritonavir fixed dose combination tablet 200 mg/50 mg x 2 tablets for a single dose of 400 mg/100 mg and a total daily dose of 800 mg/200 mg orally twice daily from week 1 postpartum visit until up to ~~two~~ **eight** weeks after complete BF cessation is achieved (defined as completely stopping all exposure to breast milk for ≥ 28 days), i.e., up to ~~42~~ **84** days after last exposure to breast milk, 18 months postpartum, or entry into the Maternal Health component, whichever comes first unless stopped for toxicity, other medical reasons or confirmed infant HIV infection (of all the mother's infants). Note that a mother with multiple infants (e.g., twins) will not discontinue her study treatment for the criterion of infant HIV infection if at least one of her study infants remains uninfected.

Section 4.612 Duration, 1st paragraph

Arm A (Maternal Prophylaxis Arm): Cessation of BF is defined as completely stopping all exposure to breast milk for ≥ 28 days, based on maternal report. The study drugs for women will be continued until up to ~~two~~ **eight** weeks after complete BF cessation as defined above is achieved (i.e., up to ~~42 days~~ **84 days)** after last exposure to breast milk or 18 months postpartum (week 72-76 postpartum*), whichever comes first, unless stopped for confirmed infant HIV infection (of all the woman's study infants), toxicity, or other medical reasons. Note that a mother with multiple infants (e.g., twins) will not discontinue her study treatment for the criterion of confirmed infant HIV infection if at least one of her study infants remains uninfected.

Section 4.712 Maternal Triple ARV Prophylaxis Randomization (with Infant Prophylaxis for 6 Weeks), 2nd paragraph, 2nd sentence

The window for the Maternal Health randomization is up to ~~two~~ **eight** weeks after complete BF cessation as defined above is achieved with no plans to re-initiate BF with current infant.

Section 5.2 Study Design (Maternal Health Component)
Section 5.21 Randomization

Entry and randomization in the Maternal Health Component will occur (i) within ~~two~~ **eight** weeks of complete cessation of BF, at 18 months postpartum (72-78 weeks postpartum), or after confirmation of infant HIV infection (of all the woman's study infants), whichever comes first, among women randomized to maternal triple ARV prophylaxis in the Postpartum Component, regardless of the woman's antepartum assignment, or (ii) as soon as the week 1 visit (6-14 days postpartum) but within 28 days after delivery among women randomized to maternal triple ARV prophylaxis during pregnancy in the Antepartum Component who are ineligible (themselves or their infants) for the Postpartum Component. Women will be randomized to continue the triple ARV regimen or to stop the triple ARV regimen. Women who report that they have stopped exposure to breast milk can be screened for eligibility for the Maternal Health Component during the 28 days after last reported breast milk exposure and should be enrolled during the first ~~two~~ **eight** weeks (~~14~~ **84** days) after reaching complete breastfeeding cessation (defined as completely stopping all exposure to breast milk for $\geq$ 28 days). The triple ARV regimen will be continued during this period until randomization. Women who are randomized to discontinue the triple ARV regimen will do so within 72 hours of randomization.

Section 5.311.1 *(Inclusion Criteria 1077BM Step 1)*

Randomized to triple ARV prophylaxis as part of the Postpartum Component and has continued triple ARV prophylaxis until the current randomization without treatment interruption (defined as more than ~~seven~~ **fourteen** consecutive days of missed dosing) within the previous 30 days; *OR* randomized to triple ARV prophylaxis in the Antepartum Component but ineligible for the Postpartum Component and has continued triple ARV prophylaxis until the current randomization without treatment interruption (defined as more than seven consecutive days of missed dosing) within the previous 30 days.

Section 5.311.2 *(Inclusion Criteria 1077BM Step 1),* 1st paragraph

Within ~~two~~ **eight** weeks after complete BF cessation is achieved (defined as completely stopping all exposure to breast milk for $\geq$ 28 days); i.e., within 29 to ~~42~~ **84** days of last breast milk exposure, or reached 18 months postpartum (whichever comes first). Women who reach 18 months postpartum while still breastfeeding will be eligible for entry within two weeks before and four weeks after the week 74 visit (week 72-78);

Section 5.511 Randomization into Step 1 of the Maternal Health Component (1077BM), 1st bullet

- within 29-~~42~~ 84 days of last exposure to breast milk in women who report that they have ceased BF (i.e. up to **eight** ~~two~~ weeks after BF cessation as defined above) or at 18 months postpartum while still breastfeeding, whichever comes first (women who reach 18 months postpartum while still breastfeeding may be randomized within 72-78 weeks postpartum); or

Section 7.23 Expedited AE Reporting Period, 1st paragraph

The expedited AE reporting period for this study is the entire duration for which the subject is on or exposed to study-supplied drug and for 30 days thereafter. After this and while a participant is still in study follow-up, ~~only~~ suspected, unexpected, serious adverse drug reactions (SUSARs, as defined in the DAIDS EAE Reporting Manual) and fetal deaths occurring at or after 20 weeks gestation (in primary pregnancies and in new pregnancies) that are judged by the site investigator to be related to study-supplied drug must be reported in an expedited manner to DAIDS. (IRIS events are not reportable SUSARs because they are expected.) **In addition, report ALL infant deaths in an expedited fashion for up to 104 weeks of infant follow-up.**

Clarification Memorandum #3 for:

IMPAACT 1077BF
Breastfeeding Version of the PROMISE Study
(Promoting Maternal and Infant Survival Everywhere)
Version 3.0, dated 15 October 2012
IND # 107,507

DAIDS Document ID 10777

Clarification Memorandum Date: 5 December 2013

Information/Instructions to Study Sites

This Clarification Memorandum has been approved by the NIAID Medical Officers. Institutional Review Board/Ethics Committee (IRB/EC) approval of this Clarification Memorandum is not required by the sponsor prior to implementation; however, sites may submit it to the responsible IRBs/ECs for their information or, if required by the IRBs/ECs, for their approval prior to implementation.

None of the clarifications being made impact the sample informed consent forms and the benefit-to-risk ratio for participants is not affected in any way.

This Clarification Memorandum should be maintained in each site's essential documents file for IMPAACT 1077BF. It is the responsibility of the Investigator of Record to ensure that all study staff are made aware of and follow this Clarification Memorandum.

Summary of Clarifications and Implementation

In the listings of study-supplied study drugs in protocol Sections 2.516, 4.61, and 5.414, the 100 mg capsule formulation of zidovudine is added, as shown below, with additions to the text indicated in bold.

Sections 2.516, 4.61, and 5.414

Generic Name<br>Abbreviation<br>Trade Name	Formulation	Appearance	Storage
Zidovudine<br>ZDV<br>Retrovir®	300 mg tablets	Biconvex, white, round, film-coated tablets	15-25°C (59-77°F)
	**100 mg capsules**	**White, opaque cap and body with "Wellcome" and unicorn logo on cap and "Y9C" and "100" on body**	**15-25°C (59-77°F) and protected from moisture**

Letter of Amendment #2

Site-Specific for CRSs 30303, 30306, 30313 for:

IMPAACT 1077BF
Breastfeeding Version of the PROMISE Study
(Promoting Maternal and Infant Survival Everywhere)
Version 3.0, dated 15 October 2012
IND # 107,507

DAIDS Document ID 10777
Letter of Amendment Date: 30 September 2013

Information/Instructions to Clinical Research Sites 30303, 30306 and 30313 from the Division of AIDS

This Letter of Amendment (LoA) is specific to the following Clinical Research Sites (CRSs) located in Zimbabwe: St Mary's Clinic, Chitungwiza (30303); Seke North Clinic, Chitungwiza (30306); and Parirenyatwa Clinic, Harare (30313). This LoA is not applicable to any other study sites.

This LoA impacts the IMPAACT 1077BF study sites in Zimbabwe only and must be submitted to their Institutional Review Boards (IRBs)/Ethics Committees (ECs) as soon as possible for review and approval with revised site-specific informed consent forms. IRB/EC approval is required prior to implementation of this LoA. Approval must also be obtained from other site regulatory entities if applicable per their policies and procedures. All IRB/EC and regulatory entity requirements must be followed.

Upon receiving IRB/EC approval and approval of any other applicable regulatory entities, this LoA is to be implemented immediately. Sites are still required to submit a LoA registration packet to the DAIDS Protocol Registration Office (DAIDS PRO) at the Regulatory Support Center (RSC). Sites will receive a registration notification for the LoA after the DAIDS PRO verifies that all required registration documents have been received and are complete. Sites should not await this notification before implementing this LoA.

This LoA, all associated IRB/EC and regulatory entity correspondence, and all correspondence with the DAIDS PRO should be retained in the essential documents files for IMPAACT 1077BF for each site.
If the IMPAACT 1077BF protocol is amended in the future, the contents of this LoA will be incorporated into the next version of the protocol.

Summary of Revisions and Rationale

Hair sample collection from women randomized to continue a triple ARV regimen in the Maternal Health Component (1077BM Arm A) in Zimbabwe.

The "Hair Substudy" described in protocol Section 1.4 previously involved only collection of hair specimens from consenting women and their infants participating in the Postpartum Component (1077BP) for evaluation as a potential biomarker for adherence to antiretrovirals (ARVs). This LoA extends collection of hair samples to consenting women participating in the Maternal Health Component (1077BM) at the Zimbabwe sites only when they are randomized to continue a triple ARV regimen (Arm A). These samples will be analyzed to assess continued ARV adherence after the period of risk of mother-

to-child HIV transmission has ended and to attempt to validate the hair analysis method at the University of Zimbabwe pharmacokinetics laboratory. Participation in this adherence substudy is optional for individual participants. Because consent for the collection of hair specimens is required, additional wording is added to the 1077BM consent form. However, the decision to participate does not otherwise affect a woman's participation in 1077BM. At the time of implementation of this LoA, all women already randomized to 1077BM Arm A and in follow-up as well as women newly enrolled to 1077BM will be offered participation in the Hair Substudy.

Implementation

Detailed modifications of the protocol text are indicated below using bold type for additions.

1) Section 1.4, PROMISE Substudies: ARV Adherence and Exposure through Hair Substudy, below paragraph 3:

   Site-specific Collection of Hair Samples

   For the Zimbabwe sites only, the Hair Substudy objectives are extended to women who are randomized to Arm A of the Maternal Health Component to additionally assess ARV adherence after the period of risk for maternal-to-child transmission (MTCT). Programmatic data suggest that the post-breastfeeding period is a particularly vulnerable time, when the motivation to adhere to a triple antiretroviral drug regimen may decline. Drug concentration levels in hair samples will provide an objective measure of adherence among healthy women who are no longer at a risk of transmitting HIV to their infant, due to breastfeeding cessation or because the child is already infected. Hair assays have been developed for all prevalent-use NNRTIs and PIs. Women will separately be asked during the informed consent process for the Maternal Health Component to permit collection of hair samples in the event that they are randomized to continue the regimen; however, their participation in the Maternal Health Component is not contingent upon agreement for collection of these additional specimens. Hair will be collected at scheduled visits from all consenting women in Arm A who are beyond the period of risk for MTCT while they continue on a triple ARV regimen in the Maternal Health Component. The samples collected from these women will be larger (more hair strands) than the samples collected in the Postpartum Component to allow for validation of the hair assay in the local Zimbabwe pharmacokinetic laboratory. These samples will be divided in two, with one portion analyzed at the University of California San Francisco (UCSF) Hair Drug Studies Unit and one portion analyzed at the University of Zimbabwe to assess for equivalence of assay results. Only the results from the assays completed the UCSF Hair Drug Studies Unit will be included as part of the PROMISE study data. Women enrolled in the substudy in the Maternal Health Component, who subsequently become pregnant, will have hair collection permanently discontinued upon identification of the new pregnancy.

2) Section 5.7, Sample Informed Consent Form: Maternal Health Component (Zimbabwe sites only):

   Tests and procedures at the study visits

   [For Zimbabwe sites only:]

   - ***[Hair***

     We ask that you allow us to collect about 100 strands of hair from you at each visit while you are receiving anti-HIV medications. If you agree to have your hair collected, this will be done only if you are assigned to the study group that continues the triple anti-HIV medications and you have either completely stopped breastfeeding or your infant is HIV-infected. The hair will be checked to see how much of the anti-HIV medicine is in the hair strands and to help develop

a hair-testing laboratory in Zimbabwe. Medicines go into many places in the body, including the hair. The amount of medicine found in the hair may provide a better idea of how much medicine could be in the body after a long period of time. Collecting the hair from you is voluntary. You will be asked to indicate whether you agree to have hair collected, when you sign this consent form. Half of the hair collected will be kept in the laboratory in Zimbabwe and half will be shipped to a study laboratory outside of Zimbabwe. You will not be told how much of the anti-HIV medicine was found in your hair. This information will be used as a research tool. Your hair samples will be kept during the study period. If you do not agree to have your hair collected, or you change your mind later, you can still participate in this part of the PROMISE study.]

<u>Risks of the Study Tests</u>

[For Zimbabwe sites only: **There is a very small chance that you might be cut during collection of the hair strands with the scissors.*]***

SIGNATURE PAGE

If you have read this consent form (and had it explained to you), and if all your questions have been answered and you agree to take part in this part of the PROMISE study, please sign your name below.

[For Zimbabwe sites only: **Please indicate whether you agree to have your hair collected for this part of the study.*]***

[I agree to allow my hair to be collected for testing how much anti HIV medicine is found in my hair, as discussed in this consent form.]

No ____ Yes ____

3) Appendix IC, Maternal Health Schedule of Evaluations (1077BM, All Steps) (for the Zimbabwe sites only):

TIME BASED VISITS							EVENT BASED VISITS				
	SCREENING [a]	BM ENTRY [b]	WK 4	WK 8 [c]	WK 12	WK 24 & Q12 WKS	EVENT DRIVEN VISIT [d]	PREMATURE D/C OF STUDY DRUG [e]	EARLY D\C OR END OF STUDY [f]	STEP CHANGE ENTRY [g]	STEP CHANGE WK 4 [h]
HAIR COLLECTION											
**Stored Hair (Arm A only)** [10]		**X**	**X**		**X**	**X**			**X**		

10. Zimbabwe sites only: Collect hair from women randomized to Arm A and in whom breastfeeding risk for MTCT has ceased, at all indicated time points, while receiving a triple ARV regimen (Steps 1, 2, or 3). For women who experience a new pregnancy, permanently discontinue hair collection upon identification of the new pregnancy.

Letter of Amendment #1 for:

IMPAACT 1077BF
Breastfeeding Version of the PROMISE Study
(Promoting Maternal and Infant Survival Everywhere)
Version 3.0, dated 15 October 2012
IND # 107,507

DAIDS Document ID 10777

Letter of Amendment Date: 02 April 2013

Information/Instructions to Study Sites from the Division of AIDS

The information contained in this Letter of Amendment (LoA) impacts the IMPAACT 1077BF study and must be submitted to site Institutional Review Boards (IRBs) and/or Ethics Committees (ECs) as soon as possible for their review and approval. The information contained in this LoA does not impact the sample informed consent forms for IMPAACT 1077BF. Nonetheless, IRB/EC approval is required prior to implementation of this LoA. Approval must also be obtained from site regulatory entities if applicable per the policies and procedures of the regulatory entities. All IRB/EC and regulatory entity requirements must be followed.

Upon receiving IRB/EC approval and approval of any other applicable regulatory entities, this LoA is to be implemented immediately. Sites are still required to submit a LoA registration packet to the DAIDS Protocol Registration Office (DAIDS PRO) at the Regulatory Support Center (RSC). Sites will receive a registration notification for the LoA after the DAIDS PRO verifies that all required registration documents have been received and are complete. Sites should not await this notification before implementing this LoA.

Please file this LoA, all associated IRB/EC and regulatory entity correspondence, and all correspondence with the DAIDS PRO in your essential documents files for IMPAACT 1077BF.

If the IMPAACT 1077BF protocol is amended in the future, the contents of this LoA will be incorporated into the next version of the protocol.

This LoA updates HIV testing options for purposes of maternal eligibility determination. The updates restore testing options that were permitted under protocol Version 2.0 and remain appropriate for use under protocol Version 3.0. The updates also modify the definition of a positive result for quantitative HIV RNA PCR.

Inclusion Criteria 2.411.1 (for 1077BA Step 1), 3.312 (for 1077BL), and 4.511.9 (for 1077BP Step 1) are updated as shown below, using strikethrough for deletions and bold type for additions.

2.411.1 Confirmed HIV-1 infection, defined as documented positive results from <u>two</u> samples collected at <u>different</u> timepoints prior to study entry:

Sample # 1 may be tested using any of the following:

- Two rapid antibody tests from two different manufacturers or based on different principles and epitopes
- One EIA OR Western Blot OR immunofluorescence OR chemiluminescence
- One HIV DNA PCR
- One quantitative HIV RNA PCR (~~> 5,000 copies/mL~~ **above the limit of detection**)
- One qualitative HIV RNA PCR
- One total HIV nucleic acid test

Sample #1 may be tested by non-study public or PEPFAR programs. However, both the result and the sample collection date must be recorded in the subject's chart. Source documentation (patient's medical record/chart, MOH register, laboratory results, etc.) must be available if requested.

Sample #2 may be tested using any of the following:

- **Rapid antibody test. If this option is used in combination with two rapid tests on Sample #1, at least one of the three rapid tests must be FDA-approved and the third rapid test must be from a third manufacturer or based on a third principle or epitope.**
- One EIA ~~confirmed by~~ **OR** Western Blot OR immunofluorescence OR chemiluminescence
- One HIV DNA PCR
- One quantitative HIV RNA PCR (~~> 5,000 copies/mL~~ **above the limit of detection**)
- One qualitative HIV RNA PCR
- One total HIV nucleic acid test

Sample #2 must be tested in a laboratory that operates according to GCLP guidelines, participates in appropriate external quality assurance programs and is approved by the IMPAACT Central Laboratory.

3.312 HIV-1 infection, defined as documented positive results from tests performed on one sample at any time prior to Late Presenter Registration

Sample #1 may be tested using any of the following:

- Two rapid antibody tests from <u>two</u> different manufacturers or based on <u>different</u> principles and epitopes
- One EIA OR Western Blot OR immunofluorescence OR chemiluminescence
- One HIV DNA PCR
- One quantitative HIV RNA PCR (~~> 5,000 copies/mL~~ **above the limit of detection**)
- One qualitative HIV RNA PCR
- One total HIV nucleic acid test

Sample #1 may be tested by non-study public or PEPFAR programs. However, both the result and the sample collection date must be recorded in the subject's chart. Source documentation (patient's medical record/chart, MOH register, laboratory results, etc.) must be available if requested.

Note: Confirmatory testing on a second specimen collected at a different timepoint per the algorithm below should be performed as soon as possible and is required for final eligibility determination for and randomization into the Postpartum Component (1077BP) at the week 1 visit (6-14 days postpartum).

Sample #2 may be performed using any of the following:

- **Rapid antibody test. If this option is used in combination with two rapid tests on Sample #1, at least one of the three rapid tests must be FDA-approved and the third rapid test must be from a third manufacturer or based on a third principle or epitope.**
- One EIA ~~confirmed by~~ **OR** Western Blot OR immunofluorescence OR chemiluminescence
- One HIV DNA PCR
- One quantitative HIV RNA PCR (~~> 5,000 copies/mL~~ **above the limit of detection**)
- One qualitative HIV RNA PCR
- One total HIV nucleic acid test

Sample #2 must be tested a laboratory that operates according to GCLP guidelines, participates in appropriate external quality assurance programs and is approved by the IMPAACT Central Laboratory.

4.511.9 *For Registered Late Presenters*: Confirmed maternal HIV-1 infection, defined as documented positive results from two samples collected at different time points at any time prior to entry in 1077BP. (Note: Results of testing performed on sample #1 were required prior to LP registration; results of the testing performed on the second sample are required prior to entry into the PP Component.)

Sample #1 may be tested using any of the following:

- Two rapid antibody tests from different manufacturers or based on different principles and epitopes
- One EIA OR Western Blot OR immunofluorescence OR chemiluminescence
- One HIV DNA PCR
- One quantitative HIV RNA PCR (~~> 5,000 copies/mL~~ **above the limit of detection**)
- One qualitative HIV RNA PCR
- One total HIV nucleic acid test

Sample #1 may be tested by non-study public or PEPFAR programs. However, both the result and the sample collection date must be recorded in the subject's chart. Source documentation (patient's medical record/chart, MOH register, laboratory results, etc.) must be available if requested.

Sample #2 may be tested using any of the following:

- **Rapid antibody test. If this option is used in combination with two rapid tests on Sample #1, at least one of the three rapid tests must be FDA-approved and the third rapid test must be from a third manufacturer or based on a third principle or epitope.**
- One EIA ~~confirmed by~~ **OR** Western Blot OR immunofluorescence OR chemiluminescence
- One HIV DNA PCR
- One quantitative HIV RNA PCR (~~> 5,000 copies/mL~~ **above the limit of detection**)
- One qualitative HIV RNA PCR
- One total HIV nucleic acid test

Sample #2 must be tested in a laboratory that operates according to GCLP guidelines, participates in appropriate external quality assurance programs and is approved by the IMPAACT Central Laboratory.

Clarification Memorandum #2 for:

IMPAACT 1077BF
Breastfeeding Version of the PROMISE Study
(Promoting Maternal and Infant Survival Everywhere)
Version 3.0, dated 15 October 2012
IND # 107,507

DAIDS Document ID 10777

Clarification Memorandum Date: 22 March 2013

Information/Instructions to Study Sites

This Clarification Memorandum (CM) has been approved by the NIAID Medical Officers. Institutional Review Board/Ethics Committee (IRB/EC) approval of this CM is not required by the sponsor prior to implementation; however, sites may submit it to the responsible IRBs/ECs for their information or, if required by the IRBs/ECs, for their approval prior to implementation.

None of the clarifications being made impact the sample informed consent forms and the benefit-to-risk ratio for participants is not affected in any way.

This CM should be maintained in each site's essential documents file for IMPAACT 1077BF. It is the responsibility of the Investigator of Record to ensure that all study staff are made aware of and follow this CM.

Summary of Clarifications

This CM incorporates minor corrections and clarifications of the maternal Schedules of Evaluations and clarifies expectations for repeat evaluation following identification of hyperbilirubinemia attributed to atazanavir and for consulting the Clinical Management Committee.

Implementation

The clarifications listed below are effective immediately and will be incorporated into the next protocol amendment. Additions to the text are indicated in bold; deletions are indicated by strike-through.

1. In Appendix IA, Maternal Schedule of Evaluations, including Late-Presenters and Observational Women (1077BA (ALL STEPS), 1077BP (ALL STEPS), and 1077BL), footnote 7 is clarified as follows:

   7. ALT and serum creatinine for all women. Once the creatinine result is available, the Cockroft-Gault equation to calculate creatinine clearance for women should be used. HB_sAg+ women and women co-enrolled on IMPAACT P1084s (Tenofovir substudy) will have additional chemistries performed as noted in the table below; ~~no additional blood is required~~.

Study Visit(s)	*Additional Chemistries (Local Lab)*	*Targeted women*
Collect at ENTRY and every 4 weeks through L/D. Thereafter, collect at every indicated visit. ~~except Postpartum Week 1.~~ Also collect at Event Driven visits that are indicated for possible HBV exacerbation.	ALT, serum creatinine, AST, alkaline phosphatase, total bilirubin and albumin **(only ALT and serum creatinine are required at Postpartum Week 1)**	HBsAg+ women ONLY
Postpartum Week 38	ALT, serum creatinine, AST, alkaline phosphatase, total bilirubin and albumin	HBsAg+ women ONLY
P1084s Entry (occurs at 1077BA Entry or the Antepartum Week 2 visit), L/D or Postpartum Week 1, Postpartum Weeks 6, 26 and 74	Phosphorus and calcium	Women in IMPAACT P1084s ONLY

2. In Appendix IC, Maternal Health Schedule of Evaluations (1077BM, ALL STEPS), the required timing for administration of adherence interviews is clarified in footnote 3 as follows:

   3. Adherence questionnaires are required **for all women at entry and thereafter** at indicated timepoints for mothers in 1077BM Step 1 Arm A, Step 2 and/or Step 3 while receiving a triple ARV regimen. Adherence questionnaires are not required following premature discontinuation of study drug.

3. In Appendix II, Toxicity Management, the general guidelines for management of grade 3 and grade 4 toxicities are clarified as follows:

   a. Under General Guidelines for Other Grade 3 Toxicities, fourth and sixth paragraphs *(fourth paragraph is shown to provide the context for additions in the sixth paragraph):*

   For Grade 3 clinical and laboratory toxicities assessed as possibly, probably or definitely related to study drug, with the exception of isolated Grade 3 hyperbilirubinemia attributed to ataz**a**navir (ATV), the implicated study drug(s) should be replaced or the entire regimen held, unless the site investigator feels that continuation of the current regimen is in the participant's best interest. If the site investigator feels that continuation of the current regimen is in the participant's best interest, the CMC should be informed. For Grade 3 isolated hyperbilirubinemia attributed to ATV, ATV may be continued unless associated with jaundice or scleral icterus that presents an intolerable cosmetic concern to the participant.

   For all Grade 3 toxicities, **with the exception of isolated Grade 3 hyperbilirubinemia attributed to ATV**, the participant should be re-evaluated weekly until the toxicity improves to Grade ≤ 2 or until stabilized.

   b. Under Guidelines for Grade 4 Toxicities, third paragraph*:*

   For all Grade 4 toxicities, with the exception of isolated Grade 4 hyperbilirubinemia attributed to ataz**a**navir (ATV), all study drugs should be held until improvement of the toxicity to Grade ≤ 2 (for infants on NVP prophylaxis, NVP should be replaced with 3TC). Alternatively, the site investigator may continue study drug only if he or she has compelling evidence that the toxicity is NOT related to study drug. In this case, consultation with the CMC is required within 3 working days. The participant should be re-evaluated weekly until the toxicity improves to Grade ≤ 2 or until stabilized. For Grade 4 isolated hyperbilirubinemia attributed to ATV, ATV may be continued unless associated with jaundice or scleral icterus that presents an intolerable cosmetic concern to the participant**; weekly re-evaluation is not required for Grade 4 isolated hyperbilirubinemia attributed to ATV**.

4. For consistency within and across protocol sections, expectations for consultation with the Clinical Management Committee (CMC) are clarified in Section 4.6, Study Treatment (Postpartum Component), as follows:

   At enrollment/randomization, it is expected that the assigned maternal and infant regimens (listed above) will use study-supplied study drugs. Thereafter, if one or more of the assigned study-supplied study drugs cannot be tolerated, the regimen may be modified (in consultation with the CMC if required per Appendix II) using study-supplied study drugs (see listing in Section 4.61) and/or drugs from other sources. In addition, for mothers who may have experienced intolerance or toxicity to one or more of the ARVs included in the preferred maternal regimen while pregnant, consideration may be given to use of alternative regimens from the time of randomization into 1077BP~~, in~~ **(**consultation with the CMC **available but not required)**. Study site clinicians are advised to consult the CMC regarding such participants in advance of the Week 1 postpartum visit, at which randomization into 1077BP takes place.

Clarification Memorandum #1 for:

IMPAACT 1077BF
Breastfeeding Version of the PROMISE Study
(Promoting Maternal and Infant Survival Everywhere)
Version 3.0, dated 15 October 2012
IND # 107,507

DAIDS Document ID 10777

Clarification Memorandum Date: 16 January 2013

Information/Instructions to Study Sites

This Clarification Memorandum has been approved by the NIAID Medical Officers. Institutional Review Board/Ethics Committee (IRB/EC) approval of this Clarification Memorandum is not required by the sponsor prior to implementation; however, sites may submit it to the responsible IRBs/ECs for their information or, if required by the IRBs/ECs, for their approval prior to implementation.

None of the clarifications being made impact the sample informed consent forms and the benefit-to-risk ratio for participants is not affected in any way.

This Clarification Memorandum should be maintained in each site's essential documents file for IMPAACT 1077BF. It is the responsibility of the Investigator of Record to ensure that all study staff are made aware of and follow this Clarification Memorandum.

Summary of Clarifications and Rationale

Minor clarifications of the required timing of evaluations are incorporated and minor inconsistencies are corrected. In addition, protocol specifications for grading the severity of hemoglobin values are clarified.

Implementation

The modifications included in this Clarification Memorandum will be incorporated into the next protocol amendment as specified below. Additions to the text are indicated in bold; deletions are indicated by strike-through.

1. Sections 2.613.2, 4.722, and 5.522, Criteria for entering Step 3 (1077BA, 1077BP, and 1077BM)

The note clarifying entry into Step 3 (1077BA, 1077BP, and 1077BM) has been changed to agree with other sections of the protocol. Protocol Sections 2.613.2, 4.722, and 5.522 will now agree with Sections 2.613.4, 4.724 and 5.524 as follows:

NOTE: If a participant experiences one of the above conditions but the condition is judged by the study clinician as due to non-adherence, systemic illness or other explanatory circumstance, such that a change of regimen is not indicated~~, with approval from the CMC,~~ entry into Step 3 is not required **(consultation with the CMC available but not required).**

2. **Sections 2.62, 4.73, and 5.53, Concomitant Medications (1077BA, 1077BP, and 1077BM)**

The requirement to routinely record on a case report form whether or not a participant has used alternative, complementary medications/preparations is eliminated. This information will continue to be collected in source documents and recorded as necessary on other applicable case report forms. Protocol Sections 2.62, 4.73, and 5.53 are modified as follows:

Sections 2.62 and 4.73, 3rd bullet

- ~~For both mothers and infants, the names of alternative, complementary medications/preparations are not required – only whether or not such substances have been used since the last visit.~~

Section 5.53, 1st paragraph, last sentence

~~The names of alternative, complementary medications/preparations are not required – only whether or not such substances have been used since the last visit.~~

3. **Section 4.4 Counseling on HIV and Infant Feeding (Postpartum Component)**

Under *Continued Reassessment of AFASS Criteria during PROMISE Visits*, the sixth paragraph is changed to agree with the Infant Schedule of Evaluations (SoE) in Appendix IB. The language is clarified as follows:

Each individual situation for BF will be reassessed at the week 22 and week 50 visits using the AFASS screening tool. In addition, as part of the infant feeding practices questionnaire, this will be assessed at ~~4 weekly~~ **12 weekly** intervals to determine whether breastfeeding has been stopped.

4. **Section 7.22 Grading Severity of Events**

Severity grading of hemoglobin values for HIV-negative participants ≥ 57 days of age is clarified as follows:

The current Division of AIDS Table for Grading the Severity of Adult and Pediatric Adverse Events, Version 1.0, dated December 2004 (with Clarification dated August 2009) must be used and is available on the RSC website at http://rsc.tech-res.com and in the study MOP. **With respect to grading hemoglobin laboratory values for HIV-negative participants ≥ 57 days of age, the DAIDS grading table provides two options, one based on absolute values and the other based on decreases from baseline; the absolute values alone and not the scheme based on decreases from baseline should be used for this study.**

5. **Appendix IA Maternal Schedule of Evaluations, including Late-Presenters and Observational Women (1077BA (ALL STEPS), 1077BP (ALL STEPS), and 1077BL)**

- Footnote 3 is changed to modify the timing for collection of smoking and alcohol intake status, as follows:

  3. All diagnoses identified in Pediatric/Maternal Diagnoses (which can be found at www.fstrf.org), Maternal Endpoint Diagnoses (Appendix IV), ≥ grade 3 signs and symptoms, and any grade sign or symptom that leads to a change in treatment, ARVs, interval bone fractures, and concomitant medications as defined in the protocol will be collected. Smoking and alcohol intake status will be collected at L/D (or week 1), ~~week 14~~ **week 26,** then every 24 weeks, and at the end of the study**.** Gynecologic status will be collected at week 14, week 50, and then every 48 weeks.

- Footnote 13 is changed to clarify the timing of hair collection, as follows:

  13. Participating Sites (1077BP only): Collect hair from mothers ~~in 1077BP Step 1 Arm A (maternal prophylaxis) at indicated time points while receiving triple ARV prophylaxis; also collect hair from all mothers following entry into 1077BP Step 2 or 1077BP Step 3 while breastfeeding and receiving a triple ARV regimen. Instructions for collection of the hair may be found in the Laboratory section of~~

~~the MOP.~~ **at indicated time points while receiving a 1077BP triple ARV regimen (Steps 1, 2 and 3) and breastfeeding (until complete cessation of breastfeeding). Collect at Early D/C if prior to Week 74; not necessary to collect at Early D/C or End of Study at anytime thereafter.**

6. Appendix IB Infant Schedule of Evaluations (1077BA, 1077BL, 1077BP)

- Under *Stored EDTA plasma, (DBS) dried blood spots*, in the table, it is clarified that storage is required ONLY at time points when HIV nucleic acid testing is performed.

- Footnote c is updated to remove reference to "End of Study" visits as follows:

  c. Infants of late-presenting women who are NOT enrolled in 1077BP should complete evaluations for "Early D/C" ~~or End of Study visit~~ at Week 6. For infants of Hep B positive women it is not necessary to collect the additional stored plasma.

- Footnote 7 (Chemistries) is updated to clarify that ALT testing, after Week 6, is required for infants in 1077BP Step 1 Arm B while receiving NVP**.** It is also updated to clarify that ALT testing is required for Late Presenter (1077BL) infants exiting the study at Week 6.

  A reference for footnote 7 is added to the indicator (X) for chemistries in the 'Early D/C' column.

  Footnote 7 is modified as follows:

  7. Infants will have chemistries assessed at the times indicated in the table below; no additional blood is required.

Study Visit(s)	*Chemistries (Local Laboratory)*	*Targeted infants*
Birth, Weeks 1 **and** 6~~, and 26~~	ALT	All infants
Weeks **26**, 38, and every 12 weeks while receiving NVP	ALT	Infants in 1077BP Step 1 Arm B (infant NVP) ONLY
**Early D/C**	**ALT**	**Infants in 1077BL completing Early D/C visits at Week 6 ONLY**
P1084s Entry (Week 1), Weeks 10, 26 and 74	Creatinine, phosphorus and calcium	Infants enrolled on IMPAACT P1084s (Tenofovir substudy) ONLY

- The numbering of footnotes 11 and 12 was previously switched and is now corrected.

7. Appendix IC Maternal Health Schedule of Evaluations (1077BM)

- For consistency with requirements in 1077BA and 1077BP (Appendix IA, Maternal SoE), the following changes are added:

  - an indicator (X) is added for Adherence Interview in the 'Premature D/C of Study Drug' column.
  - indicators are added for complete blood count (CBC) and CD4/CD8 counts in the 'Event Driven Visit' column (3mL for each) and the corresponding total blood volume is updated (24-31mL).

- Footnote 9 is added to further specify requirements for complete blood counts and CD4/CD8 counts in the 'Wk 24 & Q12 Wks' column, as follows:

9. **CD4/CD8 must be performed in a DAIDS IQA/UKNEQAS Lab. Sites performing CD4/CD8 counts in a dual platform laboratory must perform CBCs whenever CD4/CD8 cell counts are performed. At visits when CD4/CD8 counts are required, but CBCs are not (Weeks 36, 60, 84, 108, 132, 156, 180, etc), collect an additional 1 mL of blood for the CBC.**

IMPAACT 1077BF

(DAIDS Document ID 10777)

Breastfeeding Version of the PROMISE Study (Promoting Maternal and Infant Survival Everywhere)

A Multicenter, International Trial of the
International Maternal Pediatric Adolescent AIDS
Clinical Trials (IMPAACT) Group

Sponsored by:

The US National Institute of Allergy and Infectious Diseases (NIAID)
and
The *Eunice Kennedy Shriver*
National Institute of Child Health and Human Development (NICHD)

Pharmaceutical Support Provided by:

Abbott, Gilead Sciences, Boehringer Ingelheim,
and GlaxoSmithKline

IND # 107,507

**IMPAACT HIV Prevention Scientific Committee Chairs:**	Patricia Flynn, MD<br>Benjamin Chi, MD
**Protocol Chair:**	Mary Glenn Fowler, MD, MPH
**Protocol Vice Chairs:**	James McIntyre, FRCOG<br>Tsungai Chipato, MBChB, FRCOG, MCE<br>Patricia Flynn, MD
**NIAID Medical Officers and Medical Monitor:**	Lawrence Fox, MD, PhD<br>Karin L. Klingman, MD<br>Renee Browning, RN, MSN
**NICHD Medical Officers:**	Lynne Mofenson, MD<br>George Siberry, MD, MPH<br>Heather Watts, MD

Final Version 3.0
15 October 2012

IMPAACT 1077BF PROTOCOL TEAM ROSTER

Protocol and PMTCT Chair
Mary Glenn Fowler, MD, MPH
Professor, Department of Pathology
Johns Hopkins Medical Institute
Makerere U. - JHU Research Collaboration
Upper Mulago Hill Road
Kampala, Uganda
Phone: 256 414 532 091
Email: mgfowler@mujhu.org

Vice Chair and Late Presenters Chair
Patricia M. Flynn, MD
St. Jude Children's Research Hospital
Department of Infectious Disease
Memphis, TN 38105
Phone: (901) 595-2338
Email: pat.flynn@stjude.org

Maternal Health Chair
Judith Currier, MD, MSc
Professor of Medicine
UCLA CARE Center
Los Angeles, CA 90035
Phone: (310) 557-1891
Email: jscurrier@mednet.ucla.edu

Protocol Virologist
Susan Fiscus, PhD
Professor/Director of the Retrovirology Core Laboratory
UNC School of Medicine
Chapel Hill, NC 27599-7290
Phone: (919) 966-6872
Email: fiscussa@med.unc.edu

Protocol Immunologists
Katherine Luzuriaga, MD
University of Mass. Med. School
Worcester, MA 01605-2377
Phone: (508) 856-6282
Email: katherine.luzuriaga@umassmed.edu

Adriana Weinberg, MD
University of Colorado Health Sciences Center
Denver, CO 80262
Phone: (303) 315-4624
Email: adriana.weinberg@uchsc.edu

Vice Chairs
James McIntyre, FRCOG
Anova Health Institute
Johannesburg 2103, South Africa
Phone: 27 11 7155802
Email: mcintyre@pixie.co.za

Tsungai Chipato, MBChB, FRCOG, MCE
Senior Lecturer
Department of Obstetrics and Gynecology
College of Health Sciences
University of Zimbabwe
Harare, Zimbabwe
Phone: 263 4 308848
Email: tchipato@zol.co.zw

NIAID Medical Officers
Lawrence Fox, MD, Ph.D.
TRP, DAIDS, NIAID, NIH
6700-B Rockledge Drive, MSC 7624
Bethesda, MD 20892
Phone: (301) 402-0129
Email: lfox@niaid.nih.gov

Karin L. Klingman, MD
TRP, DAIDS, NIAID, NIH
6700-B Rockledge Drive, MSC 7624
Bethesda, MD 20892
Cell Phone: (240) 281-1511
Office Phone: (301) 435-3772
Email: kklingman@niaid.nih.gov

NIAID Medical Monitor
Renee Browning, RN, MSN
Henry M. Jackson Foundation
NIAID, NIH
Bethesda, MD 20892-7620
Phone: (301) 435-3770
Email: browningr@niaid.nih.gov

NICHD Medical Officers
Lynne M. Mofenson, MD
Pediatric, Adolescent and Maternal AIDS Branch
National Institute of Child Health and Human Development (NICHD)
US National Institutes of Health
Rockville, MD 20852
Phone: (301) 435-6870
Email: LM65D@nih.gov
Email: Lynne.Mofenson@nih.hhs.gov

George K. Siberry, MD, MPH
Pediatric, Adolescent and Maternal AIDS Branch
National Institute of Child Health and Human Development (NICHD)
US National Institutes of Health
Bethesda, MD 20892-7510
Phone: (301) 496-7350
Email: siberryg@mail.nih.gov

IMPAACT 1077BF PROTOCOL TEAM ROSTER

Heather Watts, MD
Pediatric, Adolescent and Maternal AIDS Branch
National Institute of Child Health and Human Development (NICHD)
US National Institutes of Health
Bethesda, MD 20892
Phone: (301) 435-6874
Email: wattsh@mail.nih.gov

Protocol Pharmacist
Lynette Purdue, PharmD
National Institutes of Health
NIAID, DAIDS, PAB
Bethesda, MD 20892-7620
Phone: (301) 435-3744
Email: lpurdue@niaid.nih.gov

Protocol Statisticians
David Shapiro, PhD
Center for Biostatistics in AIDS Research
Harvard School of Public Health
Boston, MA 02115-6017
Phone: (617) 432-2426
Email: shapiro@sdac.harvard.edu

Terrence Fenton, EdD
Principal Research Scientist
SDAC/Harvard School of Public Health
Pediatric Section
Boston, MA 02115-6017
Phone: (617) 632-2009
Email: fenton@sdac.harvard.edu

Mae P. Cababasay, MS
SDAC/Harvard School of Public Health
Boston, MA 02115
Phone: (617) 432-4516
Email: maec@sdac.harvard.edu

Paula Britto, MS
Statistical & Data Analysis Center
Harvard School of Public Health
Boston, MA 02115
Phone: (617) 432-2522
Email: britto@sdac.harvard.edu

Yan Wang, MS
SDAC/Harvard School of Public Health
Center for Biostatistics in AIDS Research
Boston, MA 02115
Phone: (617) 432-3254
Email: ywang@sdac.harvard.edu

Li Liu, PhD
SDAC/Harvard School of Public Health
Boston, MA 02115
Phone: (617) 432-1460
Email: lliu@sdac.harvard.edu

Sean Brummel, PhD
SDAC/Harvard School of Public Health
Boston, MA 02115
Phone: (617) 423-1197
Email: sbrummel@sdac.harvard.edu

Konstantia Angelidou, PhD
Center for Biostatistics in AIDS Research
Harvard School of Public Health
Boston, MA 02115
Phone: (617) 432-2524
Email: kangelid@sdac.harvard.edu

Protocol Data Managers
Michael Basar, BS
Lead Data Manager
Frontier Science & Technology Research Foundation
Amherst, NY 14226-1056
Phone: (716) 834-0900 Ext. 7271
Email: basar.michael@fstrf.org

Linda Millar, BS
Co-Lead Data Manager
Frontier Science & Technology Research Foundation
Amherst, NY 14226-1056
Phone: (716) 834-0900 Ext. 7240
Email: millar.linda@fstrf.org

Kathleen Kaiser
Data Manager
Frontier Science & Technology Research Foundation
Amherst, NY, 14226-1056
Phone: (716) 834-0900 Ext. 7289
Email: kaiser.kathleen@fstrf.org

John Gaeddert, MPH
Data Manager
Frontier Science & Technology Research Foundation
Amherst, NY 14226-1056
Phone: (716) 834-0900 Ext. 7477
Email: gaeddert.john@fstrf.org

Linda Marillo, BA
Frontier Science & Technology Research Foundation
Amherst, NY 14226-1056
Phone: (716) 834-0900
Email: marillo@fstrf.org

IMPAACT 1077BF PROTOCOL TEAM ROSTER

CEPAC Investigators
Andrea Ciaranello, MD
Massachusetts General Hospital
50 Staniford Street, 9th floor
Boston, MA 02114
Phone: (617) 726-3812
Email: aciarnello@partners.org

Kenneth Freedberg, MD, MS
Massachusetts General Hospital
50 Staniford Street, 9th floor
Boston, MA 02114
Phone: (617) 724-3341
Email: kfreedberg@partners.org

Field Representatives
Linda Barlow-Mosha
Makerere University
Johns Hopkins Univ Research Collaboration
MU-JHU Mulago Research House
Kampala, Uganda
Phone: 256 071 270 3994
Email: lbarlow@mujhu.org

Mary Patricia Toye, RN, MS
Program Manager/Research Coordinator
Baystate Med Center and Children's Hospital
Phone: (413) 794-5399
Email: maripat.toye@bhs.org

Protocol Pharmacologist
Mark Mirochnick, MD
Boston Medical Center - Maternity 6
Boston, MA 02118
Phone: (617) 414-3754
Email: mark.mirochnick@bmc.org

Hepatitis B Substudy Investigator
Debika Bhattacharya, MD
David Greffen School of Medicine, UCLA
Los Angeles, CA 90095
Phone: (310) 206-0527
E-mail: debikab@mednet.ucla.edu

Laboratory Data Coordinators
Amy Jennings, BS
Frontier Science and Technology Research Foundation
4033 Maple Rd
Amherst, NY 14226
Phone: (716) 834-0900 Ext. 7438
Email: jennings@fstrf.org

Adam Manzella, MA
Frontier Science and Technology Research Foundation
4033 Maple Rd.
Amherst, NY 14226
Phone: (716) 834-0900 Ext. 7418
Email: manzella@fstrf.org

Amanda Zadzilka, BS
Frontier Science and Technology Research Foundation
4033 Maple Rd.
Amherst, NY 14226
Phone: (716) 834-0900 Ext. 7282
Email: zadzilka@fstrf.org

Laboratory Technologists
William B. Kabat, BS
The Children's Memorial Hospital
2300 Children's Plaza
Chicago, IL 60614-3394
Phone: (312) 227-6290
Email: bkabat@childrensmemorial.org

Amy James Loftis, BS
IMPAACT Central Laboratory
University of North Carolina at Chapel Hill
Chapel Hill, NC 27599
Phone: (919) 966-6963
Email: amy_james@med.unc.edu

PMTCT Investigators
Benjamin Chi, MD
Ctr. for Inf. Disease Res.in Zambia (CIDRZ)
Lusaka, Zambia
Phone: 260 966 859 179
Email: benjamin.chi@cidrz.org

Marc Lallemant, MD, MS
Institut de Recherche pour le Developpement
Program for HIV Prevention and Treatment
Chiang Mai 50200, Thailand
Phone: 66 53 814633
Email: marc@phpt.org

Late Presenters Investigators
Taha E. Taha, MD, PhD
Johns Hopkins University
Bloomberg School of Public Health
615 North Wolfe Street, Room E-6011
Baltimore, MD 21205
Phone: (410) 614-5255
Email: ttaha@jhsph.edu

IMPAACT 1077BF PROTOCOL TEAM ROSTER

Dhayendre Moodley, PhD, MSc
CAPRISA - Umlazi Clinical Research Site
Nelson R. Mandela School of Medicine
Durban 4051
South Africa
Phone: 27 31 2604685
Email: moodleyd1@ukzn.ac.za

Karin Nielsen, MD, MPH
UCLA School of Medicine
MCIC - Department of Pediatrics
Division of Infectious Disease
Los Angeles, CA 90095-1752
Phone: (310) 206-6369
Email: knielsen@mednet.ucla.edu

Maternal Health Investigator
Arlene Bardeguez, MD, FACOG, MPH
New Jersey Medical School
Dept. of Obstetrics, Gynecology & Women's Health
Newark, NJ 07103-2714
Phone: (973) 972-5482
Email: bardegad@umdnj.edu

Other Protocol Investigators
Anna Coutsoudis, PhD
University of KwaZulu-Natal
Dept of Pediatrics and Child Health
DDMRI Building
Congella 4013, South Africa
Phone: 27 31 260 4489
Email: coutsoud@ukzn.ac.za

Amita Gupta, MD
Johns Hopkins University
600 N. Wolfe St.
Baltimore, MD 21287
Phone: (410) 502-7696
Email: agupta25@jhmi.edu

Risa Hoffman, MD, MPH
Division of Infectious Diseases
David Geffen School of Medicine at UCLA
10833 Le Conte Ave 37-121 CHS
Los Angeles, CA 90095
Phone: (310) 623-0617
Email: rhoffman@mednet.ucla.edu

Elizabeth McFarland, MD
Associate Professor of Pediatrics
Univ. of Colorado Health Sciences Center
Pediatric Infectious Diseases
Denver, CO 80262-0001
Phone: (303) 315-2304
Email: betsy.mcfarland@uchsc.edu

Lynda Stranix-Chibanda, MBChB, MMED
Dept. of Paediatrics and Child Health College of Health Sciences
University of Zimbabwe
Phone: 263 4 704890
Email: lynda@uz-ucsf.co.zw

Gerhard B. Theron, MD
Department of Obstetrics and Gynaecology
Stellenbosch University
Tygerberg Cape Town 7505
South Africa
Phone: 27 21 9389209
Email: gbth@sun.ac.za

ICAB Representative
Lindiwe Msweli
Community Liaison
Umlazi Catholic Relief Services
Durban, KwaZulu-Natal 4001
South Africa
Phone: 27 31 260 4677
Email: mswelil5@ukzn.ac.za

Protocol Specialists
Anne Coletti, MS
Family Health International
12 Madison Street #2
Medford, MA 02155
Phone: (919) 544-7040 x11238
Email: acoletti@fhi360.org

Kathleen George, MPH
Family Health International
P.O. Box 13920
Durham, NC 27709
Phone: 919-544-7040 x11150
Email:kgeorge@fhi360.org

Megan Valentine, PA
Family Health International
P.O. Box 13920
Durham, NC 27709
Phone: (919) 544-7040 x11186
Email: mvalentine@fhi360.org

Pharmaceutical Industry Representatives
Marisol Martinez
Abbott Laboratories
13192 SW 141 Street
Miami, FL 33186
Phone: (305) 588-1750
Email: marisol.martinez@abbott.com

IMPAACT 1077BF PROTOCOL TEAM ROSTER

James F. Rooney, MD
Gilead Sciences, Inc.
333 Lakeside Drive
Foster City, CA 94404
Phone: (650) 522-5708
Email: jim.rooney@gilead.com

Oxana Ivanova
Gilead Sciences, Inc.
333 Lakeside Drive
Foster City, CA 94404
Phone: (650) 372-7656
Email: Oxana.Ivanova@gilead.com

Danielle Poulin Porter, PhD
Gilead Sciences
333 Lakeside Drive
Foster City, CA 94404
Phone: (650)522-1368
Email: danielle.porter@gilead.com

Wendy Snowden, PhD
GlaxoSmithKline R&D
United Kingdom
Phone: (440) 208-9664139
Email: wendy.x.snowden@gsk.com

Helen Watson
GlaxoSmithKline R&D
United Kingdom
Phone: (440) 208-9664503
Email: Helen.A.Watson@GSK.com

IMPAACT 1077BF SITE INVESTIGATORS

Shandukani Rahima Moosa Satellite Site
Harry Moultrie, MBChB, MSc
Chris Hani Baragwanath Hospital
Hospital Street, Soweto
Johannesburg, Gauteng 1864
South Africa
Phone: 27 11 9388189
Email: harrym@witsecho.org.za

Ashraf Coovadia, MBChB, FCP, Dip HIV Man
Rahima Moosa Mother and Child Hospital (Previously Coronation)
University of the Witwatersrand
Johannesburg
South Africa
Phone: 27 11 470 9290
Email: Ashraf.Coovadia@wits.ac.za

Renate Strehlau, MBChB, Dip HIV Man, DCH
Rahima Moosa Mother and Child Hospital (Previously Coronation)
Johannesburg
South Africa
Phone: 27 11 470 9168
Email: Renate.Strehlau@wits.ac.za

Stellenbosch University
Gerhard B. Theron, MD
Department of Obstetrics and Gynaecology
Stellenbosch University
Tygerberg Cape Town 7505
South Africa
Phone: 27 21 9389209
Email: gbth@sun.ac.za

Mark Cotton, MD
Parow Valley, KIDCRU - Ward J8
Tygerberg, Cape Town 7505
South Africa
Phone: 27 21 9384219
Email: mcot@sun.ac.za

Magdel Rossouw, MBChB
Parow Valley, KIDCRU - Ward J8
Tygerberg, Cape Town 7505
South Africa
Phone: 27 21 9384148
Email: magdel@sun.ac.za

Durban Paediatric HIV
Raziya Bobat, M.D, MBChB, FCPaeds
Nelson R. Mandela School of Medicine
University of KwaZulu-Natal, Durban 4013
South Africa
E-mail: bobat@ukzn.ac.za

Motshidi Sebitloane, FCOG, MMed
Nelson R. Mandela School of Medicine
University of Kwa Zulu Natal, Durban 4013
South Africa
Phone: 27 31 260 4250/4432
Email: sebitloanem@ukzn.ac.za

CAPRISA-Umlazi Clinical Research Site
Dhayendre Moodley, PhD MSc
Nelson R. Mandela School of Medicine
Durban 4051
South Africa
Phone: 27 31 2604685
Email: moodleyd1@ukzn.ac.za

Soweto IMPAACT
Avy Violari, MD
Diepkloof, Soweto
Johannesburg, Gauteng 1864
South Africa
Phone: 27 11 9899707
Email: violari@mweb.co.za

University of North Carolina – Lilongwe
Portia Kamthunzi, MBBS, Mtropaed, DTCH
University of North Carolina Project
Kamuzu Central Hospital/Tidziwe Centre
Lilongwe, Malawi
Phone: 265 1755056 or 265 999553034
Email: portia85117@yahoo.co.uk

Mina Hosseinipour, MD, MPH
Kamuzu Central Hospital/Tidziwe Centre
Lilongwe, Malawi
Phone: 265 1758938
Email: minach@med.unc.edu

College of Medicine – Johns Hopkins University
Newton Kumwenda, MPH, PhD
P.O. Box 1131
Blantyre, Malawi
Phone: 265 1875129
Email: nkumwenda@jhu.medcol.mw

Mac Mallewa
P.O. Box 1131
Blantyre, Malawi
Phone: 265 1875129
Email: mmallewa@mlw.medcol.mw

IMPAACT 1077BF SITE INVESTIGATORS

Kilimanjaro Christian Medical Centre
Pendo Mlay, MD
KCMC-Duke University Collaboration
P.O. Box 3010
Moshi Tanzania,
Phone: 255 786 907 074
Email: Pendomlay1975@gmail.com

Anne Buchanan, MD
KCMC-Duke University Collaboration
P.O. Box 3010
Moshi Tanzania
Phone: 255 787 955 649
Email: ann.buchanan@duke.edu

George Clinic
Namwinga Chintu, MD, MTrop, Paed, MMed (Ped)
Centre for Infectious Disease Research in Zambia (CIDRZ)
Lusaka, Zambia
Phone: 260 977 611050
Email: namwinga.chintu@cidrz.org

Mwangelwa Mubiana-Mbewe, MBChB, MMed, MBA
Centre for Infectious Disease Research in Zambia (CIDRZ)
Lusaka, Zambia
Phone: 260 977 611050
Email: Mwangelwa.Mbewe@cidrz.org

Makerere University – JHU Research Collaboration
Maxensia Owor, MBChB, MMED
Makerere University
Kampala, Uganda
Phone: 256 41 4541044
Email: maxowor@mujhu.org

Jim Aizire, MBChB, MHS
Makerere University
Kampala, Uganda
Phone: 256 414 541044
Email: jaizire@mujhu.org

University of Zimbabwe - St. Mary's, Seke North and Parirenyatwa
Tsungai Chipato, MBChB, FRCOG, MCE
Department of Obstetrics and Gynecology
University of Zimbabwe
Harare, Zimbabwe
Phone: 263 4 308848
Email: tchipato@zol.co.zw

BJ Government Medical College
Ramesh Bhosale, MD
Department of Obstetrics and Gynecology
Pune, Maharashtra 411001
India
Phone: 91 20 26128000 Ext. 233
Email: drrameshbhosale@yahoo.com

Sandhya Khadse, MD
Pune, Maharashtra 411001
India
Phone: 91 20 26052419
Email: sandhyakhadse@yahoo.com

TABLE OF CONTENTS

STUDY MANAGEMENT X

GLOSSARY XI

1.0 GENERAL INTRODUCTION TO THE PROMISE PROTOCOL 1

1.1 OVERVIEW OF THE PROMISE PROTOCOL 1
1.2 BACKGROUND 1
1.3 RATIONALE 3
1.4 PROMISE SUBSTUDIES 6
1.5 GENERAL INTRODUCTION REFERENCES 11

2.0 ANTEPARTUM COMPONENT 14

2.1 RATIONALE (ANTEPARTUM COMPONENT) 17
2.2 STUDY OBJECTIVES (ANTEPARTUM COMPONENT) 30
2.3 STUDY DESIGN (ANTEPARTUM COMPONENT) 31
2.4 SELECTION AND ENROLLMENT OF SUBJECTS (ANTEPARTUM COMPONENT) 32
2.5 STUDY TREATMENT (ANTEPARTUM COMPONENT) 36
2.6 SUBJECT MANAGEMENT (ANTEPARTUM COMPONENT) 41
2.7 REFERENCES – ANTEPARTUM COMPONENT 49
2.8 SAMPLE INFORMED CONSENT FORM – ANTEPARTUM COMPONENT SCREENING 53
2.9 SAMPLE INFORMED CONSENT FORM – ANTEPARTUM COMPONENT ENROLLMENT 58

3.0 ADMINISTRATIVE MANAGEMENT OF LATE PRESENTERS 69

3.1 RATIONALE (LATE PRESENTERS) 69
3.2 STUDY DESIGN (LATE PRESENTERS) 69
3.3 SELECTION AND REGISTRATION (LATE PRESENTERS) 69
3.4 STUDY TREATMENT (LATE PRESENTERS) 71
3.5 SUBJECT MANAGEMENT FOR 1077BL (LATE PRESENTERS) 73
3.6 ADDITIONAL INFORMATION (LATE PRESENTERS) 73
3.7 LATE PRESENTERS REFERENCES 73
3.8 SAMPLE INFORMED CONSENT FORM – LATE PRESENTER REGISTRATION 74

4.0 POSTPARTUM COMPONENT 83

4.1 RATIONALE (POSTPARTUM COMPONENT) 85
4.2 STUDY OBJECTIVES (POSTPARTUM COMPONENT) 93
4.3 STUDY DESIGN (POSTPARTUM COMPONENT) 94
4.4 COUNSELING ON HIV AND INFANT FEEDING (POSTPARTUM COMPONENT) 96
4.5 SELECTION AND ENROLLMENT OF SUBJECTS (POSTPARTUM COMPONENT) 97
4.6 STUDY TREATMENT (POSTPARTUM COMPONENT) 100
4.7 SUBJECT MANAGEMENT (POSTPARTUM COMPONENT) 106
4.8 CLINICAL PHARMACOLOGY PLAN (POSTPARTUM COMPONENT) 115
4.9 REFERENCES – POSTPARTUM COMPONENT 118
4.10 SAMPLE INFORMED CONSENT FORM – POSTPARTUM COMPONENT 121

5.0 MATERNAL HEALTH COMPONENT 133

5.1 OVERALL DESIGN AND RATIONALE (MATERNAL HEALTH COMPONENT) 135
5.2 STUDY DESIGN (MATERNAL HEALTH COMPONENT) 139
5.3 SELECTION AND ENROLLMENT OF SUBJECTS (MATERNAL HEALTH COMPONENT) 140
5.4 STUDY TREATMENT (MATERNAL HEALTH COMPONENT) 143
5.5 SUBJECT MANAGEMENT (MATERNAL HEALTH COMPONENT) 145
5.6 MATERNAL HEALTH REFERENCES 153
5.7 SAMPLE INFORMED CONSENT FORM – MATERNAL HEALTH COMPONENT 155

6.0 STATISTICAL CONSIDERATIONS FOR PROMISE 165

6.1 STATISTICAL CONSIDERATIONS FOR ANTEPARTUM COMPONENT OF PROMISE....165
6.2 STATISTICAL CONSIDERATIONS FOR THE POSTPARTUM COMPONENT OF PROMISE 172
6.3 STATISTICAL CONSIDERATIONS FOR MATERNAL HEALTH COMPONENT OF PROMISE 177
6.4 STATISTICAL REFERENCES 186

7.0 REQUIREMENTS FOR CASE REPORT FORM RECORDING AND SAE REPORTING....187

7.1 CASE REPORT FORM (CRF) RECORDING REQUIREMENTS 187
7.2 ADVERSE EVENT REPORTING TO DAIDS....187

8.0 HUMAN SUBJECTS CONSIDERATIONS....190

8.1 IRB/EC REVIEW AND SAMPLE INFORMED CONSENT 190
8.2 SUBJECT CONFIDENTIALITY 190
8.3 STUDY DISCONTINUATION 191

APPENDIX IA MATERNAL SCHEDULES OF EVALUATIONS (1077BA, 1077BL, 1077BP)....192

APPENDIX IB INFANT SCHEDULE OF EVALUATIONS 197

APPENDIX IC MATERNAL HEALTH SCHEDULE OF EVALUATIONS 200

APPENDIX II TOXICITY MANAGEMENT....203

APPENDIX III OVERVIEW OF INTERIM MONITORING OF PROMISE....215

APPENDIX IV MATERNAL ENDPOINT DIAGNOSES 217

APPENDIX V SAMPLE CONSENT FORM WOMEN WHO BECOME PREGNANT ON STUDY DRUGS 220

APPENDIX VI SAMPLE CONSENT FOR SPECIMEN STORAGE AND FUTURE USE....224

APPENDIX VII HEPATITIS B SUBSTUDY ANALYSIS AND MONITORING PLAN 227

STUDY MANAGEMENT

For complete guidance on study management questions and communications, please see Section 1 of the IMPAACT 1077BF Manual of Procedures (MOP).

Email the Computer Support Group at the Data Management Center (DMC) (user.support@fstrf.org) to have relevant site personnel added to the protocol email group (promise.prot1077bf@fstrf.org) immediately after completing protocol registration. Inclusion in the protocol e-mail group will ensure that sites receive important information about the study during its implementation and conduct.

General Questions: Email questions concerning any aspect of protocol interpretation and/or study implementation not listed below, including administrative, ethical, regulatory, clinical, counseling, data and laboratory operations, to promise.questions@fstrf.org. See Figure 1-1 in Section 1 of the 1077BF MOP for more information on communication with the PROMISE Questions Email Group.

Clinical Management Questions and Notifications: Email questions concerning clinical management of study subjects and adverse experiences to the study Clinical Management Committee (CMC): promise.cmc1077bf@fstrf.org. Questions related to participant eligibility, potential enrollment of an ineligible participant, and/or deviation from other protocol requirements for screening and enrollment should also be directed to the CMC. See Figures 1-2 and 1-3 in Section 1 of the 1077BF MOP for more information on communications with the CMC. Do not include the randomized/registered study arm in correspondence with the CMC unless specifically requested or necessary for the clinical management question being asked.

Co-Enrollment: Email questions related to co-enrollment in 1077BF and other studies to the CMC: promise.cmc1077bf@fstrf.org.

Randomization/Registration: For randomization/registration questions or problems and study identification number (SID) lists, email rando.support@fstrf.org or call the DMC Randomization Desk at (716) 834-0900 x7301.

Computer and Screen Problems: For computer and screen problems, email user.support@fstrf.org or call the DMC at (716) 834-0900 x7302.

Product Package Inserts or Investigator Brochures: Product package inserts or investigator brochures may be accessed on the DAIDS Regulatory Support Center (RSC) web site: http://rsc.tech-res.com.

Study Drug: For questions or problems regarding study drug, dose, supplies, records, and returns, contact the DAIDS Protocol Pharmacist at Lpurdue@niaid.nih.gov or (301) 496-8213.

Study Drug Orders: Email the Clinical Research Products Management Center (BIO.CRPMC.Ph@Thermofisher.com) or call (301) 294-0741.

Expedited Adverse Event (EAE) Reporting/Questions: Contact the DAIDS RSC Safety Office via email (RSCSafetyOffice@tech-res.com) or phone (1-800-537-9979 or +1-301-897-1709) or fax (1-800-275-7619 or +1-301-897-1710). For questions about the DAIDS Adverse Experience Reporting System (DAERS), email DAIDS-ESSupport@niaid.nih.gov. Questions may also be sent from within the DAERS application.

GLOSSARY	
**Study Terms**	
1077BA	Antepartum (AP) Component of 1077BF
1077BL	Late Presenters (LP) Registration/Administrative Management of 1077BF
1077BP	Postpartum (PP) Component of 1077BF
1077BM	Maternal Health (MH) Component of 1077BF
Study drug	Drug provided to a study participant consistent with protocol specifications for the participant's current component and step, regardless of source
Study-supplied study drug	Study drugs provided to participants from a supply obtained from the DAIDS Clinical Research Products Management Center or from study-specific supplies of didanosine and efavirenz provided or reimbursed by Westat
Step 1	Initial step of the AP, PP and MH Components into which eligible women are entered and randomized
Step 2	Step of the AP, PP and MH components into which a woman is moved/registered when she reaches an indication for ARV treatment for her own health according to specified criteria
Step 3	Step of the AP, PP and MH components into which a woman currently on a triple ARV regimen is moved/registered when she reaches an indication to switch to a second line regimen according to specified criteria
Cessation of breastfeeding	Completely stopping all exposure to breast milk for $\geq$ 28 days, according to the mother's report
**Acronyms**	
3TC	Lamivudine
3TC-ZDV	Combivir (fixed dose combination Lamivudine-Zidovudine)
ABC	Abacavir
ACTG	AIDS Clinical Trials Group
AE	Adverse Event
AER	Adverse Event Report
AFASS	Acceptable, feasible, affordable, sustainable, and safe
ALT	Alanine aminotransferase
ANC	Absolute neutrophil count
AP	Antepartum
ART	Antiretroviral therapy
ARV	Antiretroviral
AST	Aspartate aminotransferase
AUC	Area under the curve
BF	Breastfeeding
BHITS	Breastfeeding and HIV International Transmission Study
CBV	Combivir
CDC	US Centers for Disease Control and Prevention
CEPAC	Cost-Effectiveness of Preventing AIDS Complications
CI	Confidence Interval
CMC	Clinical Management Committee (of the study)
Cr/Cr CL	Creatinine/Creatinine Clearance
CRF	Case Report Form
CRPMC	Clinical Research Products Management Center

GLOSSARY	
CTX	Cotrimoxazole
d4T	Stavudine
DAERS	DAIDS Adverse Event Reporting System
DAIDS	Division of AIDS, NIAID
DBS	Dried blood spot
ddI	Didanosine
DHHS	Department of Health and Human Services (of the United States)
DMC	Data Management Center
DSMB	Data and Safety Monitoring Board
DXA	Dual Energy X-Ray Absorptiometry
EAE	Expedited Adverse Event
EBF	Exclusive Breast Feeding
EC	Ethics Committee
ECU	European Collaborative Study
EFV	Efavirenz
EGPAF	Elizabeth Glaser Pediatric AIDS Foundation
ELISA	Enzyme-Linked ImmunoSorbent Assay
FANTA	Food and Nutrition Technical Assistance
FAO	Food and Agriculture Organization
FDA	US Food and Drug Administration
FDC	Fixed dose combination
FF	Formula feeding
FTC	Emtricitabine
GCLP	Good clinical lab practice
GFATM	Global Fund to Fight AIDS, Tuberculosis and Malaria
HAART	Highly Active Antiretroviral Therapy
HBV	Hepatitis B Virus
HIV	Human Immunodeficiency Virus
HFIAS	Household Food Insecurity Access Scale
ICH	International Conference on Harmonization
IMPAACT	International Maternal Pediatric Adolescent AIDS Clinical Trials Group
IP	Intrapartum
IRB	Institutional Review Board
IRIS	Immune Reconstitution Inflammatory Syndrome
LAR	Legally Authorized Representative
L/D	Labor and delivery
LFT	Liver Function Test
LP	Late presenter
LPV	Lopinavir
LPV-RTV	Lopinavir-Ritonavir (Kaletra, Aluvia)
MOH	Ministry of Health
MOP	Manual of Procedures
MTCT	Mother-to-Child Transmission
NAT	Nucleic Acid Test

GLOSSARY	
NFV	Nelfinavir
NIAID	US National Institute of Allergy and Infectious Diseases
NICHD	Eunice Kennedy Shriver US National Institute of Child Health and Human Development
NIH	US National Institutes of Health
NNRTI	Non-Nucleoside Reverse Transcriptase Inhibitor
NRTI	Nucleoside Reverse Transcriptase Inhibitor
NVP	Nevirapine
OHRP	Office for Human Research Protections of the US Department of Health and Human Services
OI	Opportunistic Infection
PACTG	Pediatric AIDS Clinical Trials Group
PEPFAR	US President's Emergency Plan for AIDS Relief
PI	Protease Inhibitor
PK	Pharmacokinetic
PMTCT	Prevention of Mother-to-Child Transmission
PP	Postpartum
PoR	Pharmacist of Record
PROMISE	Promoting Maternal and Infant Survival Everywhere
PSWP	Protocol-Specific Web Page (of the IMPAACT website: www.impaactgroup.org)
QOL	Quality of Life
RAB	Regulatory Affairs Branch, DAIDS
RE	regulatory entity
RPV	Rilpivirine
RSC	DAIDS Regulatory Support Center
RTV	Ritonavir
SAE	Serious Adverse Event
sd	Single dose
SDMC	Statistical and Data Management Center
SDAC	Statistical and Data Analysis Center
SID	Study Identification Number
SGOT	Serum Glutamic Oxaloacetic Transaminase
SGPT	Serum Glutamic Pyruvic Transaminase
SIP	Site Implementation Plan
SMART	Strategies for Management of Antiretroviral Therapy Trial
SWEN	Six Week Extended Dose Nevirapine Trial
TB	Tuberculosis
TDF	Tenofovir disoproxil fumarate
TMP-SMX	Trimethoprim-Sulfamethoxazole
TRV	Truvada (fixed dose combination Emtricitabine-Tenofovir disoproxil fumarate)
ULN	Upper limit of normal
VQA	Virus Quality Assurance Program
WBC	White blood count
WHO	World Health Organization
WITS	Women and Infants Transmission Study
ZDV	Zidovudine

1.0 GENERAL INTRODUCTION TO THE PROMISE PROTOCOL

1.1 Overview of the PROMISE Protocol

The Promoting Maternal and Infant Survival Everywhere (PROMISE) Protocol is a research protocol of the IMPAACT network designed to address in an integrated and comprehensive fashion three critical questions currently facing HIV-infected pregnant and postpartum women and their infants:

1. What is the optimal intervention for the prevention of antepartum and intrapartum transmission of HIV?
2. What is the optimal intervention for the prevention of postpartum transmission in breastfeeding (BF) infants?
3. What is the optimal intervention for the preservation of maternal health after the risk period for prevention of mother-to-child-transmission ends (either at delivery or cessation of BF)?

The overall PROMISE protocol has three separate interventional components to address each of these three questions. Due to variations in the standard of care for HIV-infected pregnant and postpartum women and their infants at different IMPAACT sites, not all of these questions are relevant at all sites of the network. Three versions of the PROMISE protocol have been developed, each containing only those components relevant to the different settings of the IMPAACT network. Each version (including 1077BF) is a single protocol and must be reviewed and approved as such.

This version of the PROMISE protocol (IMPAACT 1077BF) is intended for those sites where the standard method of infant feeding is breastfeeding. All three of the interventional components described above are relevant to this version of the PROMISE protocol.

Organization of the Protocol Document

The next four sections following this general introduction (Sections 2.0-5.0) describe the rationale, design and procedures specific to the Antepartum Component, Registration of Late Presenters, the Postpartum Component and the Maternal Health Component. Each of these sections also includes a component-specific sample informed consent form. To avoid redundancy, sections detailing information, requirements and procedures that are common to all three of the components and to late presenters (statistical considerations, CRF recording/adverse event reporting and human subjects considerations) follow thereafter, as Sections 6.0, 7.0 and 8.0. Included in the appendices are the Schedules of Evaluations for mothers and infants, toxicity management guidelines and other tools, sample informed consent forms for women who get pregnant again while on study drug and for specimen storage and additional information regarding the Hepatitis B Substudy.

1.2 Background

In the absence of preventive interventions, 25-40% of infants born to HIV-infected mothers get infected, approximately 10% during pregnancy, 15% during delivery and 15% or more through BF. In the absence of therapy, more than half of these infected infants will die within two years of life.

Over the past two decades, considerable strides have been made in the prevention of MTCT of HIV. The administration during pregnancy of virologically suppressive triple antiretroviral (ARV) drug combinations, conventionally referred to as “Highly Active Antiretroviral Therapies” (HAART), cesarean section delivery and infant formula-feeding (FF) have led to a decrease of the risk of MTCT to less than 2% and the virtual elimination of new pediatric HIV infection in the US, Europe and other resource-advantaged settings. Remaining issues essentially revolve around improved service delivery, in particular the detection of all HIV-infected women early enough during pregnancy so that ARV prophylaxis is fully

effective as well as the relative safety of the ARV drug combinations used during pregnancy for the fetus, the child and the mother, especially the issue of the safety for the mother of stopping triple ARV regimens used solely for prevention of MTCT.

In contrast, in resource-limited settings, the incidence of pediatric HIV infection remains extremely high. It was estimated that about 330,000 new pediatric HIV infections occurred in 2011 (1). While this partly reflects the enormous number of women of reproductive age infected with HIV (17.7 million at the end of 2006), it also highlights the lack of implementation of known, effective prevention methods in many resource-limited settings. It was estimated that in 2011, only 57% of the 1.5 million HIV-infected pregnant women worldwide had been offered an intervention to protect their child from HIV infection (1). In most cases, these HIV-infected women identified late during pregnancy or even only at delivery receive very short courses of ARV drugs, thereby limiting their efficacy. Moreover, the vast majority of women breastfeed. Indeed, infant feeding in resource-limited settings poses a major dilemma. While the use of formula or other replacement feeding completely eliminates the risk of postnatal HIV, when sanitary conditions are inadequate, it is associated with an increase of infant mortality and morbidity, which often outweighs the risk of HIV transmission.

Although BF infants are protected against other causes of mortality and morbidity, they are exposed to HIV until BF cessation, which also is associated with a significant increased risk of morbidity and mortality. Implementation of the most effective interventions for PMTCT has lagged in resource-limited areas for a variety of reasons, including feasibility and cost. However, with increased commitment from foreign donors and governments, many barriers to providing more efficacious – but more complex – PMTCT regimens are being overcome. Through national and international programs (such as the President's Emergency Plan for AIDS Relief (PEPFAR), the Global Fund to fight AIDS, Tuberculosis and Malaria (GFATM), the Elizabeth Glaser Pediatric AIDS Foundation (EGPAF)), access to PMTCT for pregnant women and HAART for immunocompromised patients is rapidly expanding. While issues of human resources and drug access are still concerns, the infrastructure necessary to provide higher standards of clinical care is increasingly available worldwide. For HIV-infected women who do not need treatment for their own health, in 2010 the World Health Organization (WHO) recommended initiation of antiretroviral prophylaxis (either maternal ZDV/single dose nevirapine (sdNVP) prophylaxis or triple drug prophylaxis with regimens that include ZDV or TDF as one of the three ARV drugs for PMTCT beginning as early as 14 weeks gestation followed by infant prophylaxis for six weeks and, in breastfeeding settings, continued infant or maternal prophylaxis until breastfeeding cessation). (See revised WHO recommendations on the use of antiretroviral drugs for treating pregnant women and preventing HIV infection in infants, 10 July 2010, http://www.who.int/entity/hiv/pub/mtct/antiretroviral2010/en/index.html). The WHO issued a Programmatic Update in April 2012 expressing a preference for use of triple ARV prophylaxis (called "Option B") because of potential program simplification and harmonization with adult treatment guidelines and also discussed consideration of initiation of life-long treatment in all pregnant women regardless of CD4 count (called "Option B+") (see http://www.who.int/hiv/pub/mtct/programmatic_update2012/en/index.html). The WHO acknowledged that the presumed benefits of the Option B and B+ prophylaxis options need to be critically evaluated and that systems and support requirements will need careful consideration when policymakers consider implementation (2). As this international context evolves, it is critical that resource-appropriate approaches to PMTCT are evaluated for efficacy, safety and cost-effectiveness, to determine optimal strategies for implementation.

On June 18-19, 2012, the Division of AIDS assembled a panel of independent experts including an international group of ethicists, clinicians, researchers, a representative from an African Ministry of Heath, and an HIV-infected female community member, to advise the Institute on the ethical viability of the PROMISE study as currently designed given the changing landscape of PMTCT guidelines. The

panel concluded that the current evidence continues to demonstrate similar efficacy for PMTCT for the PROMISE antepartum and postpartum interventions, although programmatic implementation issues may differ, and therefore found no compelling reason why randomization in PROMISE would be unethical. They noted that the WHO Program Update http://www.who.int/hiv/pub/mtct/programmatic_update2012/en/index.htm) stated, "There is an urgent need to assess country experience and evidence that address the preferences among Options A, B, and B+." The panel concluded that PROMISE will provide evidence that will prove to be valuable for addressing some of the current evidence gaps related to future clinical, policy and program decisions. The panel noted that some of the planned secondary analyses—particularly those related to some of the key programmatic, operational and clinical questions occupying the field—were also likely to yield evidence that could be extremely valuable for the on-going management of the implementation of various regimens, including Option B (triple antiretroviral drug prophylaxis) and B+ (life-long antiretroviral drug for all pregnant women). For example, PROMISE is designed to provide evidence about various aspects of ARV adherence and retention in care, HIV drug resistance associated with the various regimens, and safety of the increased ARV exposure for the fetus/infant. The panel also noted that the informed consent procedures implemented across the trial sites must properly appraise women of the implications of their choice to participate in the PROMISE trial, including in countries implementing Option B/B+ the fact that they could be randomized to receive a level of care that may be different than that provided in the national program, along with the risks and benefits associated with each level of care.

1.3 Rationale

Use of either triple ARV prophylaxis or zidovudine (ZDV), initiated at 28 weeks of pregnancy plus peripartum single dose nevirapine (sdNVP) in women with higher CD4 counts (i.e., $\geq$ 350 cells/mm^3) who do not need immediate therapy for their own health, seems to reduce intrauterine and intrapartum transmission of HIV from mother to child to similarly low rates, and the WHO now recommends that one of these two approaches be initiated as early as 14 weeks gestation. However, there are not yet randomized clinical trial data directly comparing the two strategies and their relative benefits in terms of efficacy, safety, feasibility and cost-effectiveness. Recent results suggest that the provision of triple ARV prophylaxis to women during BF or provision of ARV prophylaxis to their infants during BF can considerably reduce the risk of HIV postnatal transmission while maintaining the health benefits of BF, and in 2010 the WHO recommended that one of these two strategies be employed but the two have not yet been directly compared in a randomized trial with sufficient sample size to provide strong evidence of the superiority of one strategy or the equivalence of these strategies.

Although no increase in disease progression has been seen so far in studies of pregnant women with relatively high CD4 cell counts who stop triple ARV drug regimens after delivery (3-5), the available data remain limited and the consequences in terms of safety and toxicity of stopping triple ARV regimens used solely for PMTCT among women with high CD4 cell counts is not known, nor is the benefit of continuing triple ARV regimens indefinitely following initiation during pregnancy or BF.

The PROMISE study will be conducted both in settings in which formula feeding (FF) is acceptable, feasible, affordable, sustainable and safe (AFASS) for HIV-infected women, as well as in more resource-limited settings where these AFASS criteria are not met, and the WHO recommends exclusive BF for at least the first six months of life with introduction of appropriate complementary foods thereafter and continued BF for the first 12 months of life. With the ultimate objective of "promoting maternal and infant survival everywhere" (PROMISE), in resource-limited as well as resource-advantaged settings, PROMISE has been designed to answer the intricate questions related to the optimal intervention for the prevention of intrauterine and intrapartum transmission of HIV, the prevention of HIV transmission through BF, the preservation of maternal health and the prevention of infant morbidity and mortality related to BF cessation.

Building upon the wealth of expertise and the diversity of the IMPAACT network, the PMTCT Scientific Committee has designed an integrated research protocol with three sequential randomization components, each designed to address one of the following three main objectives:

1. *Antepartum Component:* To compare the efficacy of triple ARV prophylaxis versus ZDV initiated at 14 weeks of pregnancy (or as soon as possible thereafter) plus sdNVP/Truvada (TRV) prophylaxis for the prevention of HIV *in utero* and intrapartum transmission in HIV-infected pregnant women with CD4 cell count $\geq$ 350 cells/mm^3 in both FF and BF settings in resource-limited countries; and to compare the safety of the trial antepartum study regimens.
2. *Postpartum Component:* To compare the efficacy and safety of maternal triple ARV prophylaxis versus daily infant NVP prophylaxis for the prevention of mother-to-child transmission (PMTCT) through BF, among women with CD4 cell count $\geq$ 350 cells/mm^3 who received antepartum ARV prophylaxis or who first present at labor/delivery.
3. *Maternal Health Component:* To assess the clinical benefit and safety of an antepartum maternal triple ARV regimen versus the ZDV + sdNVP + TRV tail regimen used for PMTCT and, in those women who receive the antepartum triple ARV regimen, continuing versus stopping the regimen, among those who do not require treatment for their own health (CD4 cell count $\geq$ 350 cells/mm^3) in both FF and BF settings.

The sequential randomization design has several advantages. It is statistically efficient because women and their infants may contribute to answering more than one question and is also flexible with respect to allowing the inclusion of different types of participants (e.g., late presenters or FF women) in only certain components of the trial. This design is also robust to modifications of the interventions in the various components that might occur during the conduct of the study due to external findings. For example, if release of results of an external study of a PMTCT intervention requires modification of the treatment arms in the PROMISE Antepartum Component, then the PROMISE Postpartum and Maternal Health Components would remain evaluable. The Antepartum Component of PROMISE would remain valid, although its power would be diminished depending on when the changes occurred.

The PROMISE team recognizes that IMPAACT sites vary in their antepartum standard of care for women with CD4 $\geq$ 350 cells/mm^3 and that standards of care for PMTCT prophylaxis are rapidly changing. Three versions of the PROMISE protocol have been developed: one for resource-limited country sites where breastfeeding is standard and where all three objectives – antepartum, postpartum and maternal health – are addressed (1077BF); one for resource-limited country sites where formula feeding is standard and only the antepartum and maternal health objectives are addressed (1077FF); and a final protocol for sites in which formula feeding and maternal triple drug prophylaxis are standard, addressing only the maternal health objective (1077HS). Data from 1077BF and 1077FF will be combined to address the antepartum and maternal health objectives.

The Antepartum Component randomization will be to the ZDV + sdNVP + TRV tail or one of two triple drug prophylaxis regimen strategies: 3TC-ZDV/LPV-RTV or FTC-TDF/LPV-RTV. While the greatest experience in pregnancy is with the 3TC-ZDV dual NRTI backbone, the current 2010 WHO guidelines include FTC-TDF or 3TC as a recommended dual NRTI backbone for pregnant women (http://www.who.int/hiv/pub/mtct/antiretroviral2010/en/index.html), and the April 2012 Programmatic Update is recommending that the preferred triple drug regimen during pregnancy is a TDF-based triple regimen (http://www.who.int/hiv/pub/mtct/programmatic_update2012/en/index.html). The U.S. perinatal guidelines recommend TDF as an alternative NRTI for use in pregnancy (http://www.aidsinfo.nih.gov/guidelines/html/3/perinatal-guidelines/0/). The inclusion of two different triple prophylaxis regimen arms will also allow for comparison of maternal and infant safety outcomes related to drugs included in the WHO-recommended regimens for use during pregnancy and breastfeeding. Also, because there are

limited data available specifically regarding the safety of TDF use in pregnancy for the mother and the infant, PROMISE will co-enroll women and infants in a substudy called IMPAACT P1084s that will compare potential TDF toxicity endpoints (bone and renal) in women and their infants exposed to TDF during pregnancy those women and infants who were not exposed to TDF during pregnancy.

Table 1 shows the number of mothers and infants targeted to be randomized in each component of PROMISE for each protocol version. It is important to note that, although 1077BF has three randomization components and 1077FF has two randomization components, the overall number of *unique* mother-infant pairs to be enrolled in PROMISE is much less than the sum of the component sample sizes. This is because 1077BF has only two points of entry (the Antepartum Component for eligible women who present prior to labor and the Late Presenter Registration for eligible women who present in labor or within five days after delivery) and 1077FF has only one point of entry (the Antepartum Component); the remaining PROMISE components will only enroll women and/or infants who participated in one of these initial randomization components. In Table 1, the numbers of unique subjects are italicized: 1077BF is targeted to enroll a total of 5,900 unique mother-infant pairs (3,400 during pregnancy and approximately 2,500 during labor or within 5 days after delivery); 1077FF is targeted to enroll a total of 1,000 unique mother-infant pairs (all during pregnancy); and 1077HS is targeted to enroll a total of 2,000 women (all after delivery).

Table 1: Targeted Number of Mother-Infant Pairs, Women or Infants to be Enrolled in each PROMISE Component and Protocol Version

PROMISE Component	1077BF	1077FF	1077HS
**Antepartum Randomization**	*3,400 pairs**	*1,000 pairs**	0
**Late Presenters Registration**	*2,500 pairs**	0	0
**Postpartum Randomization**			
From Antepartum Component**	3,100 pairs	0	0
Late Presenters	1,550 pairs*	0	0
**Maternal Health Randomization**			
After delivery**	100 women***	475 women	*2,000 women**
After BF MTCT risk ceases**	2,100 women	0	0

*Initial enrollment in PROMISE (in *italics*). It is projected that a total of 2,500 late presenting mother-infant pairs will need to be registered to the Late Presenter Registration in order to identify 1,550 late presenting mother-infant pairs who are eligible for the Postpartum Component.
**For 1077BF and 1077FF, the numbers shown are only the numbers of pairs, women or infants who are projected to meet eligibility criteria and agree to be randomized in that component. In addition, all women and infants who participated in a previous PROMISE randomization but are not eligible for or do not agree to be randomized in a subsequent randomization will continue to be followed on-study as a comparison group.
***Projected number of women in the Antepartum triple ARV prophylaxis arm who will be ineligible for the Postpartum Randomization due to infant ineligibility or stillbirth but will still be eligible for the Maternal Health randomization.

The protocol team considered whether to open the Postpartum and Maternal Health randomizations to women who were otherwise eligible but had not participated in a prior component of PROMISE but decided against this strategy as it would further complicate an already complex protocol and potentially introduce biases. The rationale for not enrolling such "external" women in either the Postpartum or Maternal Health Component is as follows:

- External women would not contribute to key study objectives that require combined data from multiple components of PROMISE: evaluation of the efficacy and safety of the combined Antepartum and Postpartum randomized interventions from pregnancy through 18 months

postpartum; assessment of potential interactions between the Antepartum and Postpartum randomized interventions; and assessment of potential interactions between the Maternal Health randomized intervention (stopping or continuing the triple ARV regimen) and the prior duration of triple ARV prophylaxis (e.g., during breastfeeding only vs. during both pregnancy and breastfeeding). Allowing enrollment of external women would reduce the number of PROMISE "graduates" (women participating in previous components) who could be enrolled to address these objectives and would thereby reduce power, unless the sample size for each component was increased, which would further increase the high cost of PROMISE.

- PROMISE graduates will be easier to enroll and will have reliable medical histories that are readily available. External women would likely have received more heterogeneous interventions during pregnancy/breastfeeding and could differ from PROMISE graduates with respect to key characteristics; for example, while PROMISE component graduates and external women would all be required to have a CD4 count $\geq$ 350 cells/mm^3 at the time of the Maternal Health randomization, PROMISE graduates would also have been required to have a CD4 count $\geq$ 350 cells/mm^3 at the time of their initial enrollment in PROMISE, a criterion which may not hold or may not be assessable for external women.

- Enrolling external women would increase the already high cost of PROMISE because all women enrolled in the Antepartum or Postpartum Component of PROMISE will be followed for the duration of the PROMISE trial for maternal health outcomes whether or not they enroll in a subsequent component of PROMISE; this cost increase would occur even if the sample sizes for each component are not changed, because allowing external women to enroll would increase the total number of unique mother-infant pairs.

1.4 PROMISE Substudies

The PROMISE study includes six substudies as outlined below. These include investigations into ARV resistance; cost-effectiveness of the maternal and infant ARV strategies being evaluated; Hepatitis B/HIV co-infection; ARV adherence and exposure through hair measures; immunologic correlates of protection from HIV transmission as measured in breast milk and plasma; and the safety and pharmacokinetics of Tenofovir. The objectives for the first five of these are included as part of the main protocol and the associated assessments are covered in the schedules of evaluation and the study informed consent forms for each component. The Tenofovir safety substudy is described in a separate protocol (IMPAACT P1084s) because it requires additional specimens and assessments and a separate informed consent form.

ARV Resistance Substudy

As use of antiretroviral drugs (ARVs) for HIV-1 prevention and treatment increases globally, resistance to ARVs will likely become more common. Emergence of drug resistance may be related to several factors including: use of regimens that are not fully suppressive, poor adherence to ARV regimens (because of interruption in the availability of ARVs, toxicities, co-morbidities and/or non-compliance), and the low HIV-1 genetic threshold for resistance to some drugs. Host genetic factors may also affect bioavailability of ARVs, influencing emergence of resistance in some settings. The PROMISE study provides a number of opportunities to explore the likelihood of the development of resistance in women and infants exposed to different antenatal and postnatal regimens for PMTCT, and in women in the Maternal Health Component who are continuing the triple drug regimen indefinitely after BF cessation (as would be done in "Option B+", in which life-long ART is started on all pregnant women regardless of CD4 cell count). Given increasing data suggesting adherence to antiretroviral therapy is significantly decreased postpartum, it is important to evaluate if continuing triple drugs reduces or alternatively enhances the development of drug resistance (6-8). Because women entering PROMISE may already

have received ARVs for PMTCT in a prior pregnancy, we may detect resistant HIV variants at baseline, and this may affect the efficacy of the PROMISE PMTCT regimens.

The PROMISE study will be conducted at sites worldwide; therefore, women infected with a variety of HIV-1 subtypes will be enrolled. Previous studies show that HIV-1 subtype can dramatically affect the emergence and persistence of ARV resistance in women and infants in the setting of PMTCT. PROMISE will be the first study to compare HIV transmission and the development/duration of ARV resistance in women and infants infected with a large variety of HIV-1 subtypes.

Evaluations to be conducted as part of the ARV resistance substudy include HIV-1 resistance testing, population sequencing, minority variants analysis and HIV-1 subtype determination. Other related studies may include characterization of HIV viruses from women and their infants (e.g., sequencing of regions other than *pol*, and assays measuring phenotypic resistance, replication capacity and HIV tropism), and to evaluate the host response to HIV infection. These analyses may involve comparisons between groups, tests of association between resistance status and clinical outcomes, or analysis of descriptive information concerning various aspects of resistance. Because we will not know in advance which women will transmit HIV to their infants, or which specimens will eventually be selected for resistance testing, specimens will be stored for resistance testing at selected study visits. However, resistance testing will not be done for all subjects or at all time points.

Cost-Effectiveness Substudy

The cost of ARV drugs, as well as of HIV care more broadly, has become a primary concern in both resource-rich and resource-limited settings as therapy has become more effective over the past decade. While PMTCT with sdNVP has been shown to be both efficacious and cost-effective, whether the additional benefits of triple ARV prophylaxis compared to less complex regimens such as ZDV/sdNVP prophylaxis provide adequate value, considering the additional costs, remains a question. Further, the cost and value of providing pregnant women who have CD4 counts $\geq$ 350 cells/mm^3 with triple ARV prophylaxis, and continuing that regimen after delivery if formula feeding or after breastfeeding cessation if breastfeeding (the “Option B+”, in which life-long ARV is started on all pregnant women regardless of CD4 cell count), remain uncertain. The PROMISE study will allow a detailed assessment of the cost-effectiveness of these interventions, providing policy makers in a multitude of countries results that can be used directly in decision-making.

To understand and disseminate the policy implications of the PROMISE trial, the team has added internationally-recognized expertise in HIV cost-effectiveness analysis by collaborating with the Cost-Effectiveness of Preventing AIDS Complications (CEPAC) team. The CEPAC model is a widely published HIV simulation model, which incorporates data on natural history, treatment efficacy, cost and quality of life to project long term outcomes and policy relevance from shorter-term clinical trial data. The CEPAC model has been used to help inform HIV practice and guidelines for care in the United States, France, South Africa, India, Côte d’Ivoire and the Caribbean (9-20). The model is a state-transition Monte Carlo simulation of HIV disease in adults and is updated regularly with data in the four domains described above. Investigators will determine the cost and cost-effectiveness of different strategies for PMTCT on the survival and morbidity of pregnant women and outcomes in children of these women in resource-poor settings.

The analyses will reflect outcomes limited to the timeframe of the trial, as well as projected beyond the end of the trial. Outcomes will include opportunistic infections (OIs), significant non-AIDS-related clinical events (cardiovascular, renal, hepatic and malignant disease), mortality and total direct medical costs. Cost-effectiveness is reported in dollars per year of life saved as well as dollars per quality-adjusted life year saved. For each of the main randomizations, simulations will be conducted to

understand the cost-effectiveness of the trial strategies. Each strategy will be compared to the others in order of increasing costs, and cost-effectiveness will be calculated incrementally. Any strategy which is more expensive but less effective than another strategy will be considered "dominated." Results will be tabulated as well as presented as efficiency frontiers, allowing the clinician or policy analyst to understand the tradeoff of cost for additional clinical benefit, and these results will be compared to the country-specific GDP.

Hepatitis B Substudy

Hepatitis B virus (HBV) co-infection is common, affecting greater than 10% of HIV-infected individuals in resource-limited settings (21-23). Although the impact of HIV disease on HBV co-infection has been studied in non-pregnant adults, little is known about the effects of HIV on HBV during pregnancy, particularly the optimal antepartum triple ARV prophylaxis regimen in HIV/HBV co-infected women. Accordingly, in its 2009 consensus statement on hepatitis B, the NIH has identified the study of the risks and benefits of antiviral therapy in pregnancy as a top research priority (24). However, in many resource-limited settings, HBV screening is not available and HIV/HBV co-infected pregnant women subsequently receive various regimens of HBV-active PMTCT drugs. Additionally, although US and WHO guidelines recommend the use of two drugs active against HBV in co-infected patients starting treatment (25), this standard has not been routinely applied to pregnant women. The WHO guidelines recommend use of two drugs active against HBV (e.g., TDF + 3TC) for pregnant women with HBV coinfection who require HBV treatment but acknowledge the limited data about potential maternal and infant bone toxicity with use of TDF.

This substudy will explore HBV disease outcomes among HIV/HBV co-infected women entering the PROMISE Antepartum Component. In the Antepartum Component, hepatitis B positive women will be randomized in a 1:1:1 ratio to a ZDV-based ARV regimen vs. 3TC-ZDV/LPV-RTV vs. FTC-TDF/LPV-RTV. Follow-up of these women (and their infants) will continue throughout their participation in the main study, including randomizations to ARV regimens during postpartum and post-breastfeeding follow-up. Assuming an approximate 3.5% to 7% prevalence of HBV co-infection within the main study population, approximately 154 – 308 women and their infants will be evaluated as part of this substudy. It is hypothesized that, after eight weeks; HIV/HBsAg+ co-infected pregnant women assigned to receive FTC-TDF/LPV-RTV will have larger decreases in hepatitis B viral load from baseline, when compared to women who were assigned to receive 3TC-ZDV/LPV-RTV.

The primary objective of this substudy is to compare the anti-HBV efficacy of antepartum 3TC-ZDV/LPV-RTV (single HBV active therapy) vs. FTC-TDF/LPV-RTV (combination HBV-active therapy) as measured by changes in maternal HBV DNA viral loads during the antepartum period (primary endpoint at 8 weeks), a key predictor of HBV vertical transmission. Other HBV outcomes that will be evaluated are: 1) mother-to-child transmission of HBV and HBV characteristics (including genotype, drug resistance, pre-core and core promoter mutants and DNA viral load) among babies contracting HBV and among transmitting mother-infant pairs; 2) maternal HBV DNA viral loads and presence of HBV drug resistance at delivery and postpartum; 3) HBV virologic and biochemical changes after cessation of the triple ARV regimen; and 4) maternal anemia at delivery among HIV/HBV co-infected women. Analysis plans and monitoring for this substudy is further described in Appendix VII.

ARV Adherence and Exposure through Hair Substudy

Data from HIV pre-exposure prophylaxis (PrEP) trials (26, 27) and PMTCT settings, including those in resource-limited countries (6-8, 28) indicate that actual adherence to prophylactic antiretroviral (ARV) agents is not always concordant with self-report or other commonly-used adherence measures. The iPrEx trial demonstrated a profound relationship between high adherence and effectiveness of ARVs for PrEP,

and the importance of an objective biomarker (ARV levels in peripheral blood mononuclear cells (PBMCs)) of adherence (29). The PEARL study similarly evaluated the importance of an independent adherence biomarker for PMTCT, specifically umbilical cord blood ARV levels (28). Objective biomarkers of adherence may be particularly critical for monitoring ARV use as prophylaxis for HIV-uninfected persons in whom HIV viral loads cannot serve as surrogate markers of adherence. However collection of PBMCs, cord blood or breast milk in PrEP or PMTCT settings is expensive and burdensome.

ARVs are incorporated from the systemic circulation into hair over weeks to a month. Hair concentrations of ARVs are the strongest predictor of virologic success in large prospective cohorts of HIV-infected patients on treatment (30-34) and are much stronger independent predictors of outcomes than self-reported adherence or single plasma levels. This substudy will examine the use of hair samples to monitor ARV adherence and exposure in infants and mothers in PROMISE. Besides their utility in monitoring adherence, quantitative measurements of ARV levels in infants whose mothers are receiving HAART can examine the kinetics of ARV transfer during pregnancy and breastfeeding. Preliminary studies using hair measures in infants born of HIV-infected mothers have demonstrated significant transfer of lopinavir (LPV) and ritonavir (RTV) during pregnancy, but negligible transfer of both of these agents during breastfeeding (35). ARV concentrations in infants may be particularly determinative of protection, toxicities and resistance if acquisition does occur.

The objectives of this substudy include assessing adherence/exposure to lopinavir/ritonavir (LPV-RTV) for women and their breastfeeding infants randomized to 1077BP Step1 Arm A and to nevirapine (NVP) for infants randomized to 1077BP Step 1 Arm B by measuring hair drug concentrations; assessing the kinetics of LPV and RTV transfer from mother to baby during breastfeeding; assessing the relationship between hair drug concentrations and infant acquisition of HIV infection in each arm; assessing the relationship between hair drug concentrations and secondary outcomes, including maternal or infant toxicities; and assessing the relationship between hair drug concentrations and plasma ARV drug concentrations in selected mother-infant pairs. For sites that elect to conduct this substudy, women will separately be asked during the informed consent process for the Postpartum Component of 1077BF to permit collection of hair samples from themselves and their infants; participation in the Postpartum Component of 1077BF is not contingent upon agreement for collection of these additional specimens. Hair will be collected at monthly visits from all infants while breastfeeding whose mothers consent and from all mothers on a triple ARV regimen (prophylaxis or HAART) while breastfeeding.

Correlates of Protection Substudy

HIV-1 has been detected in breast milk as both cell-free and cell-associated virus. It is unclear which virus population is responsible for infant breast milk transmission, as both the level of breast milk viral RNA and the number of infected breast milk cells are associated with risk of transmission to the infant (36-42). Early studies indicate that transmission of HIV continues to occur at a rate of 2 to 4% with optimal, monitored maternal or infant prophylactic therapy (43-47). Importantly, cell-associated virus remains detectable in milk during antiretroviral therapy (48, 49) and may account for the breast milk transmission that continues to occur in the setting of antiretroviral prophylaxis. Moreover, both maternal and infant toxicity associated with the use of antiretroviral treatment, as well as noncompliance and the development of drug resistant mutations in the virus, may hinder large-scale implementation of maternal antiretroviral therapy during lactation.

Interestingly, in the absence of maternal or infant antiretroviral prophylaxis, only 10% of breastfeeding infants born to HIV-infected mothers become infected via breast milk ingestion in spite of chronic virus exposure for up to two years. This relatively low rate of breast milk transmission suggests that immune responses in milk could be contributing to protection of the breastfeeding infant. HIV transmission via

breast milk is associated with both high maternal plasma and breast milk virus load (39-41). Maternal peripheral blood CD4+ T lymphocyte count is also strongly correlated with risk of breast milk HIV transmission (38, 50, 51), suggesting a possible role for the maternal immune response in preventing HIV transmission via breast milk. However, maternal immune correlates of protection against breast milk transmissions of HIV have not been defined. Also, antibody responses against the V1V2 region of the gp120 Env protein have recently been implicated as important in protection against HIV acquisition (52). Therefore, characterization of virus-specific antibody immune responses in breast milk and their effect on local virus replication and transmission will be important in developing strategies for immunologic interventions to impede breast milk transmission of HIV.

The primary objective of this substudy is to compare the HIV-specific heterologous and autologous neutralizing and binding antibody responses in plasma and breast milk of postnatal transmitting and nontransmitting mothers at the time of postnatal HIV transmission. Approximately 40 mother-infant pairs with breast milk transmission and 80 non-transmitting mother-infant pairs are targeted for these analyses. No additional breast milk or blood will be collected from maternal participants. For infants > 6 weeks of age who have a positive result on an initial HIV test, additional plasma may be collected when the specimen for confirmatory testing is obtained (or as soon thereafter as possible); collection of this blood is covered as part of informed consent form for the Postpartum Component of 1077BF. There are otherwise no subject participation requirements.

Tenofovir Safety Substudy (IMPAACT P1084s)

For many women, TDF may be an effective and well-tolerated part of a combination ARV regimen that treats maternal illness (HIV, HBV or both) and prevents maternal-to-child transmission antepartum, perinatally and through breast milk. TDF is now included as one of the WHO-recommended ARV drug options for triple ARV regimens for treatment and PMTCT prophylaxis in pregnant and breastfeeding women. Based on animal and non-pregnant human studies, the potential TDF toxicities of greatest concern are renal toxicity and bone toxicity and fetal/infant growth restriction. No major toxicity signals have been reported despite increasing use of TDF in pregnant and lactating women worldwide. Available evidence indicates that maternal TDF during breastfeeding is unlikely to produce significant direct infant systemic exposure to the bioactive form of tenofovir (53). Limited studies have provided some reassuring data that TDF during pregnancy does not negatively impact offspring bone or growth outcomes (54-56). However, the effects of prolonged maternal TDF use on pregnant/lactating women and their infants have not been adequately studied. The PROMISE study offers an opportunity to evaluate in more detail the safety of TDF-containing triple ARV prophylaxis in pregnancy compared to non-TDF containing ARV regimens and less complex ZDV-containing prophylaxis that are currently more commonly used. As pregnant women will be randomly assigned to TDF-containing and non-TDF containing ARV regimens, antepartum enrollment of these women in this study will allow for further evaluation of their renal function, bone turnover and bone density and thus assess the potential differences due to TDF. In addition, the infants of these women can be assessed for differential effects of antepartum TDF vs. no TDF on infant growth, on baseline bone status, and on baseline renal status. This substudy is described in a separate protocol entitled: IMPAACT P1084s, Maternal and Infant Monitoring for Evidence of Toxicity Related to Tenofovir Exposure: The Bone and Kidney Health Substudy of 1077 PROMISE.

1.5 General Introduction References

(1) World Health Organization. Together we will end AIDS – 2012. 2012, Geneva, Switzerland. URL: http://www.unaids.org/en/media/unaids/contentassets/documents/epidemiology/2012/20120718_togetherwewillendaids_en.pdf.

(2) World Health Organization. Programmatic Update – use of antiretroviral drugs for treating pregnant women and preventing HIV infection in infants – Executive Summary April 2012. Geneva, Switzerland. URL: http://www.who.int/hiv/pub/mtct/programmatic_update2012/en/index.html

(3) Watts DH, Lu M, Thompson B, et al. Treatment interruption after pregnancy: effects on disease progression and laboratory findings. *Infect Dis Obstet Gynecol.* 2009;2009:456717.

(4) Palacios R, Senise J, Vaz M, Diaz R, Castelo A. Short-term antiretroviral therapy to prevent mother-to-child transmission is safe and results in a sustained increase in CD4 T-cell counts in HIV-1-infected mothers. *HIV Med.* Mar 2009;10(3):157-162.

(5) Tai JH, Udoji MA, Barkanic G, et al. Pregnancy and HIV disease progression during the era of highly active antiretroviral therapy. *J Infect Dis.* Oct 1 2007;196(7):1044-1052.

(6) Mepham S, Zondi Z, Mbuyazi A, Mkhwanazi N, Newell ML. Challenges in PMTCT antiretroviral adherence in northern KwaZulu-Natal, South Africa. AIDS Care. 2011 Jun;23(6):741-7.

(7) Mirkuzie AH, Hinderaker SG, Sisay MM, Moland KM, Mørkve O. Current status of medication adherence and infant follow up in the prevention of mother to child HIV transmission programme in Addis Ababa: a cohort study. J Int AIDS Soc. 2011 Oct 21;14:50.

(8) Kreitchmann R, Harris DH, Kakehasi F, et al. Antiretroviral adherence during pregnancy and postpartum in Latin America. AIDS Pat Care STDs 2012;26:DOI: 10.1089/apc.2012.0013.

(9) Freedberg KA, Scharfstein JA, Seage GR, 3rd, et al. The cost-effectiveness of preventing AIDS-related opportunistic infections. JAMA 1998; 279:130-6.

(10) Walensky RP, Weinstein MC, Smith HE, Freedberg KA, Paltiel AD. Optimal allocation of testing dollars: the example of HIV counseling, testing, and referral. Med Decis Making 2005; 25:321-9.

(11) Paltiel AD, Walensky RP, Schackman BR, et al. Expanded HIV screening in the United States: effect on clinical outcomes, HIV transmission, and costs. Ann Intern Med 2006;145:797-806.

(12) Walensky RP, Weinstein MC, Yazdanpanah Y, et al. HIV drug resistance surveillance for prioritizing treatment in resource-limited settings. AIDS 2007;21:973-82.

(13) Sax PE, Islam R, Walensky RP, et al. Should resistance testing be performed for treatment-naive HIV-infected patients? A cost-effectiveness analysis. Clin Infect Dis 2005;41:1316-23.

(14) Freedberg KA, Hirschhorn LR, Schackman BR, et al. Cost-effectiveness of an intervention to improve adherence to ART in HIV-infected patients. J Acquir Immune Defic Syndr 2006;43 Suppl 1:S113-8.

(15) Schackman BR, Gebo KA, Walensky RP, et al. The lifetime cost of current human immunodeficiency virus care in the United States. Med Care 2006;44:990-7.

(16) Walensky RP, Paltiel AD, Losina E, et al. The survival benefits of AIDS treatment in the United States. J Infect Dis 2006;194:11-9.

(17) Sax PE, Losina E, Weinstein MC, et al. Cost-effectiveness of enfuvirtide in treatment-experienced patients with advanced HIV disease. J Acquir Immune Defic Syndr 2005;39:69-77.

(18) Walensky RP, Goldie SJ, Sax PE, et al. Treatment for primary HIV infection: projecting outcomes of immediate, interrupted, or delayed therapy. J Acquir Immune Defic Syndr 2002;31:27-37.

(19) Freedberg KA, Losina E, Weinstein MC, et al. The cost effectiveness of combination antiretroviral therapy for HIV disease. N Engl J Med 2001;344:824-31.

(20) Goldie SJ, Yazdanpanah Y, Losina E, et al. Cost-effectiveness of HIV treatment in resource-poor settings--the case of Cote d'Ivoire. N Engl J Med 2006;355:1141-53.

(21) Law WP, Duncombe CJ, Mahanontharit A, et al. Impact of viral hepatitis co-infection on response to antiretroviral therapy and HIV disease progression in the HIV-NAT cohort. AIDS 2004;18(8):1169-77.

(22) Matee MI, Magesa PM, Lyamuya EF. Seroprevalence of human immunodeficiency virus, hepatitis B and C viruses and syphilis infections among blood donors at the Muhimbili National Hospital in Dar es Salaam, Tanzania. BMC Public Health 2006;6:21.

(23) Sutcliffe S, Taha TE, Kumwenda NI, Taylor E, Liomba GN. HIV-1 prevalence and herpes simplex virus 2, hepatitis C virus, and hepatitis B virus infections among male workers at a sugar estate in Malawi. J Acquir Immune Defic Syndr 2002;31(1):90-7.
(24) Sorrell MF, Belongia EA, Costa J, et al. National Institutes of Health Consensus Development Conference Statement: management of hepatitis B. Ann Intern Med 2009;150:104-10.
(25) Hammer SM, Eron JJ, Jr., Reiss P, et al. Antiretroviral treatment of adult HIV infection: 2008 recommendations of the International AIDS Society-USA panel. JAMA 2008;300(5):555-70.
(26) Grant RM, Lama JR, Anderson PL, et al. Preexposure chemoprophylaxis for HIV prevention in men who have sex with men. N Engl J Med 2010;363 (27):2587-2599.
(27) Amico R, Liu A, McMahan V, et al. Adherence Indicators and PrEP Drug Levels in the iPrEx Study. 18th Conference on Retroviruses and Opportunistic Infections (CROI), Boston, MA. 2011; Paper#98LB.
(28) Stringer EM, Ekouevi DK, Coetzee D, et al. Coverage of nevirapine-based services to prevent mother-to-child HIV transmission in 4 African countries. JAMA 2010;304(3):293-302.
(29) Anderson P, Lama J, Buchbinder S, et al. Interpreting Detection Rates of Intracellular FTC-TP and TFV-DP: The iPrEx Trial. 18th Conference on Retroviruses and Opportunistic Infections (CROI), Boston, MA. 2011; Paper#96LB.
(30) van Zyl GU, van Mens TE, McIlleron H, et al. Low lopinavir plasma or hair concentrations explain second line protease inhibitor failures in a resource-limited setting. J Acquir Immune Defic Syndr 2011;56(4):333-339.
(31) Gandhi M, Ameli N, Bacchetti P, et al. Protease inhibitor levels in hair strongly predict virologic response to treatment. AIDS 2009;23(4):471-478.
(32) Gandhi M, Ameli N, Bacchetti P, et al. Concentrations of Efavirenz in Hair Are Strongly Correlated with Virologic Response. 16th Conference on Retroviruses and Opportunistic Infections (CROI), Montreal, Canada. 2009; paper 692.
(33) Gandhi M, Bacchetti P, Ameli N, et al. Atazanavir Concentration in Hair is the Strongest Predictor of Outcomes on Antiretroviral Therapy. Clin Infect Dis. 2011;52(10):1267-75.
(34) Gandhi M, Greenblatt RM, Bacchetti P, et al. A Single Nucleotide Polymorphism in CYP2B6 Leads to >3-fold Increases in Efavirenz Concentrations in Intensive PK Curves and Hair Samples in HIV-infected women. Journal of Infectious Diseases.2012 (in press)
(35) Achan J, Cohan D, Aweeka F, et al. Lopinavir and Efavirenz Concentrations in Hair Samples as a Marker of Cumulative Exposure among Postpartum Women and Breastfeeding Infants in Uganda; O-14; 4th International Workshop on HIV and Pediatrics, Washington DC, July 2012.
(36) Semba RD, Kumwenda N, Hoover DR, et al. Human immunodeficiency virus load in breast milk, mastitis, and mother-to-child transmission of human immunodeficiency virus type 1. J Infect Dis. 1999;180:93-98.
(37) Richardson BA, John-Stewart GC, Hughes JP, et al. Breast-milk infectivity in human immunodeficiency virus type 1-infected mothers. J Infect Dis. 2003;187:736-740.
(38) Pillay K, Coutsoudis A, York AD, Kuhn L, Coovadia HM. Cell-free virus in breast milk of HIV-1-seropositive women. J Acquir Immune Defic Syndr. 2000;24:330-336.
(39) Koulinska IN, Villamor E, Msamanga G, et al. Risk of HIV-1 transmission by breastfeeding among mothers infected with recombinant and non-recombinant HIV-1 genotypes. Virus Res. 2006;120:191-198.
(40) Rousseau CM, Nduati RW, Richardson BA, et al. Association of levels of HIV-1-infected breast milk cells and risk of mother-to-child transmission. J Infect Dis. 2004;190:1880-1888.
(41) Rousseau CM, Nduati RW, Richardson BA, et al. Longitudinal analysis of human immunodeficiency virus type 1 RNA in breast milk and of its relationship to infant infection and maternal disease. J Infect Dis. 2003;187:741-747.
(42) John, G. C., R. W. Nduati, D. A. Mbori-Ngacha, B. A. Richardson, D. Panteleeff, A. Mwatha, J. Overbaugh, J. Bwayo, J. O. Ndinya-Achola, and J. K. Kreiss. 2001. Correlates of mother-to-child human immunodeficiency virus type 1 (HIV-1) transmission: association with maternal plasma HIV-1 RNA load, genital HIV-1 DNA shedding, and breast infections. J Infect Dis 183:206-212.

(43) Bedri, A., B. Gudetta, A. Isehak, S. Kumbi, S. Lulseged, Y. Mengistu, A. V. Bhore, R. Bhosale, V. Varadhrajan, N. Gupte, J. Sastry, N. Suryavanshi, S. Tripathy, F. Mmiro, M. Mubiru, C. Onyango, A. Taylor, P. Musoke, C. Nakabiito, A. Abashawl, R. Adamu, G. Antelman, R. C. Bollinger, P. Bright, M. A. Chaudhary, J. Coberly, L. Guay, M. G. Fowler, A. Gupta, E. Hassen, J. B. Jackson, L. H. Moulton, U. Nayak, S. B. Omer, L. Propper, M. Ram, V. Rexroad, A. J. Ruff, A. Shankar, and S. Zwerski. 2008. Extended-dose nevirapine to 6 weeks of age for infants to prevent HIV transmission via breastfeeding in Ethiopia, India, and Uganda: an analysis of three randomised controlled trials. Lancet 372:300-313.

(44) Kumwenda, N. I., D. R. Hoover, L. M. Mofenson, M. C. Thigpen, G. Kafulafula, Q. Li, L. Mipando, K. Nkanaunena, T. Mebrahtu, M. Bulterys, M. G. Fowler, and T. E. Taha. 2008. Extended antiretroviral prophylaxis to reduce breast-milk HIV-1 transmission. N Engl J Med 359:119-129.

(45) Kilewo, C., K. Karlsson, A. Massawe, E. Lyamuya, A. Swai, F. Mhalu, and G. Biberfeld. 2008. Prevention of mother-to-child transmission of HIV-1 through breast-feeding by treating infants prophylactically with lamivudine in Dar es Salaam, Tanzania: the Mitra Study. J Acquir Immune Defic Syndr 48:315-323.

(46) Palombi, L., M. C. Marazzi, A. Voetberg, and N. A. Magid. 2007. Treatment acceleration program and the experience of the DREAM program in prevention of mother-to-child transmission of HIV. Aids 21 Suppl 4:S65-71.

(47) Chasela, C. S., M. G. Hudgens, D. J. Jamieson, D. Kayira, M. C. Hosseinipour, A. P. Kourtis, F. Martinson, G. Tegha, R. J. Knight, Y. I. Ahmed, D. D. Kamwendo, I. F. Hoffman, S. R. Ellington, Z. Kacheche, A. Soko, J. B. Wiener, S. A. Fiscus, P. Kazembe, I. A. Mofolo, M. Chigwenembe, D. S. Sichali, and C. M. van der Horst. Maternal or infant antiretroviral drugs to reduce HIV-1 transmission. N Engl J Med 362:2271-2281.

(48) Shapiro, R. L., T. Ndung'u, S. Lockman, L. M. Smeaton, I. Thior, C. Wester, L. Stevens, G. Sebetso, S. Gaseitsiwe, T. Peter, and M. Essex. 2005. Highly active antiretroviral therapy started during pregnancy or postpartum suppresses HIV-1 RNA, but not DNA, in breast milk. J Infect Dis 192:713-719.

(49) Lehman, D. A., M. H. Chung, G. C. John-Stewart, B. A. Richardson, J. Kiarie, J. Kinuthia, and J. Overbaugh. 2008. HIV-1 persists in breast milk cells despite antiretroviral treatment to prevent mother-to-child transmission. Aids 22:1475-1485.

(50) Semba, R. D., N. Kumwenda, T. E. Taha, D. R. Hoover, T. C. Quinn, Y. Lan, L. Mtimavalye, R. Broadhead, P. G. Miotti, L. van der Hoeven, and J. D. Chiphangwi. 1999. Mastitis and immunological factors in breast milk of human immunodeficiency virus-infected women. J Hum Lact 15:301-306.

(51) Embree, J. E., S. Njenga, P. Datta, N. J. Nagelkerke, J. O. Ndinya-Achola, Z. Mohammed, S. Ramdahin, J. J. Bwayo, and F. A. Plummer. 2000. Risk factors for postnatal mother-child transmission of HIV-1. Aids 14:2535-2541.

(52) Haynes BF, Gilbert PB, McElrath MJ, Zolla-Pazner S, et al. Immune-correlates analysis of an HIV-1 vaccine efficacy trial. N Engl J Med. 2012 Apr 5;366(14):1275-86.

(53) Benaboud S, Pruvost A, Coffie PA, Ekouévi DK, Urien S, Arrivé E, Blanche S, Théodoro F, Avit D, Dabis F, Tréluyer JM, Hirt D. Concentrations of tenofovir and emtricitabine in breast milk of HIV-1-infected women in Abidjan, Cote d'Ivoire, in the ANRS 12109 TEmAA Study, Step 2. Antimicrob Agents Chemother. 2011 Mar;55(3):1315-7.

(54) Viganò A, Mora S, Giacomet V, Stucchi S, Manfredini V, Gabiano C, Salvini F, Cellini M, Tamburrini E, Puzzovio M, Zuccotti GV. In utero exposure to tenofovir disoproxil fumarate does not impair growth and bone health in HIV-uninfected children born to HIV-infected mothers. Antivir Ther. 2011;16(8):1259-66.

(55) Nurutdinova D, Onen NF, Hayes E, Mondy K, Overton ET. Adverse effects of tenofovir use in HIV-infected pregnant women and their infants. Ann Pharmacother. 2008;42(11):1581-5. Epub 2008 Oct 28.

(56) Siberry GK, Williams PL, Mendez H, Seage GR 3rd, Jacobson DL, Hazra R, Rich KC, Griner R, Tassiopoulos K, Kacanek D, Mofenson LM, Miller T, Dimeglio LA, Watts DH; for the Pediatric HIVAIDS Cohort Study (PHACS). Safety of tenofovir use during pregnancy: early growth outcomes in HIV-exposed uninfected infants. AIDS. 2012;26:1151-9.

2.0 ANTEPARTUM COMPONENT: PREVENTION OF IN UTERO AND INTRAPARTUM MTCT

SCHEMA: ANTEPARTUM COMPONENT
(DMC Enrollment Screen/CRF Identifier: 1077BA)

DESIGN: Randomized strategy trial

POPULATION: HIV-infected pregnant women (both with and without HBV) who intend to BF with documented CD4 cell count at screening of $\geq$ 350 cells/mm^3 or greater than or equal to the country-specific threshold for initiation of treatment, if that threshold is > 350 cells/mm^3; enrolled from 14 weeks gestation forward and prior to the onset of labor; and who are ARV-naïve except for ARVs given for PMTCT; and their infants.

SAMPLE SIZE: For 1077BA, the accrual target is approximately 3,400 eligible, pregnant HIV-infected women who intend to BF and their infants; this target may be adjusted by the protocol team as needed to achieve the numbers of evaluable mother-infant pairs required for the Antepartum Component, Postpartum Component, and Maternal Health Component data analyses.

STRATIFICATION: By hepatitis B surface antigen (HBsAg) positive or negative status, and by country

TREATMENT REGIMEN: As outlined below, eligible women who do not need ARV treatment for their own health and their unborn infants will be randomized to one of the primary ARV regimens being evaluated (Step 1); should they subsequently need ARV treatment for their own health, women will proceed to Step 2 (for first line therapy) and/or Step 3 (for second line therapy) as outlined below. All enrolled infants will receive Nevirapine daily through at least six weeks of age, regardless of study arm. The study drug regimens for mothers and infants are detailed in Section 2.5.

<u>1077BA Step 1</u>: At entry, participants will be randomized in a 1:1:1 ratio to one of three regimens:

Arm A: ZDV + sdNVP + TRV tail
Arm B: Triple ARV regimen of 3TC-ZDV/LPV-RTV
Arm C: Triple ARV regimen of FTC-TDF/LPV-RTV

Women will receive ZDV from study entry through delivery, nevirapine and Truvada (TRV) intrapartum and TRV postpartum for 7 days or through the week 1 visit, whichever is later (Arm A). Women will receive the triple ARV study drug regimen (Arms B and C) from study entry through the 1 week visit (day 6-14) postpartum.

1077BA Step 2 - Applies to:

- 1077BA Step 1 Arm A mothers (ZDV + sdNVP + TRV tail), regardless of HBV status, who reach an indication for initiating triple ARV treatment for their own health.

- 1077BA Step 1 Arm B or C (triple ARV prophylaxis) mothers who were not randomized to the Postpartum Component, e.g., declined or were ineligible, have not been randomized in the Maternal Health Component, and have stopped the triple ARV regimen but continue follow-up and then later require triple ARV treatment for their own health.

- 1077BA Step 1 Arm B or C mothers (triple ARV prophylaxis) who reach an indication for triple ARV treatment for their own health while on a triple ARV prophylaxis regimen but do not meet the criteria for switching to a second line regimen and entry into Step 3.

All women entering Step 2 must complete a step change entry visit. For women not on a triple ARV regimen entering Step 2, the 1077BA Step 2 entry visit must be completed prior to initiation of the triple ARV regimen.

1077BA Step 3 - Applies to:

- Mothers from 1077BA Step 1 Arm B or C (while they are receiving triple ARV prophylaxis) or 1077BA Step 2 who are being followed on a triple ARV regimen for treatment if they meet the criteria for switching to a second line regimen.

The 1077BA Step 3 Entry visit must be completed prior to the first dose of the second regimen. This change can occur at any time after randomization but is anticipated to occur most often in mothers who are not enrolled on the Postpartum Component but continue to be followed.

Infants: All infants will receive NVP daily through at least six weeks of age regardless of the mother's study arm assignment.

STUDY DURATION: The total duration for the Antepartum, Postpartum and Maternal Health Components of IMPAACT 1077BF combined is expected to be approximately five years. All women will be followed until 96 weeks after the last woman in the Antepartum Component of 1077BF delivers (approximately 2-5 years, depending on rate of accrual); all infants will be followed through 104 weeks of age. Most women will remain in the Antepartum Component only from entry through the Week 1 visit (6-14 days postpartum) and then transition to a subsequent study component; those who do not enter a subsequent study component will continue to be followed in the Antepartum Component observational follow-up.

OBJECTIVES:

Primary Objectives

1. To evaluate the comparative efficacy of maternal antepartum triple ARV prophylaxis versus antepartum ZDV + sdNVP + TRV tail to reduce antepartum and intrapartum HIV transmission, as measured by the transmission rate through the Week 1 visit (6-14 days), when regimens are initiated ≥ 14 weeks gestation and prior to the onset of labor
2. To assess and compare the safety and tolerability of the three ARV regimens, including adverse pregnancy outcomes (e.g., stillbirths, prematurity, low birth weight and congenital anomalies)

Secondary Objectives

1. To assess HIV transmission rates at birth by study arms
2. To assess 24-month HIV-free survival and overall survival in infants by maternal study arm (in conjunction with infants in the Postpartum Component)
3. To evaluate adherence to the maternal ARV regimens
4. To assess rates and patterns of maternal and infant resistance to the maternal and infant ARV strategies
5. To evaluate cost-effectiveness and feasibility of the trial ARV strategies
6. To assess rates of maternal suppression to HIV RNA < 400 copies/mL according to timing of ARV drug initiation before delivery
7. In HIV/HBV co-infected women, to compare the anti-HBV efficacy of two antepartum triple ARV regimens with single (3TC-ZDV) vs. dual (FTC-TDF) HBV-active drugs, assessed as change in hepatitis B viral load during the antepartum period, as well as several other HBV-specific outcomes (MTCT of HBV and HBV characteristics in babies, presence of HBV drug resistance, HBV virologic and biochemical changes after ARV prophylaxis cessation, and maternal anemia at delivery); see Appendix VII for additional details on the HBV substudy objectives, analyses and monitoring.

SITES: This version of the PROMISE study, 1077BF, is to be conducted at IMPAACT sites and other NIAID- and NICHD-funded sites in east and southern Africa and other parts of world where breastfeeding is the norm.

Figure 1 – Antepartum Component Randomization (Step 1) in IMPAACT 1077BF (1077BA)

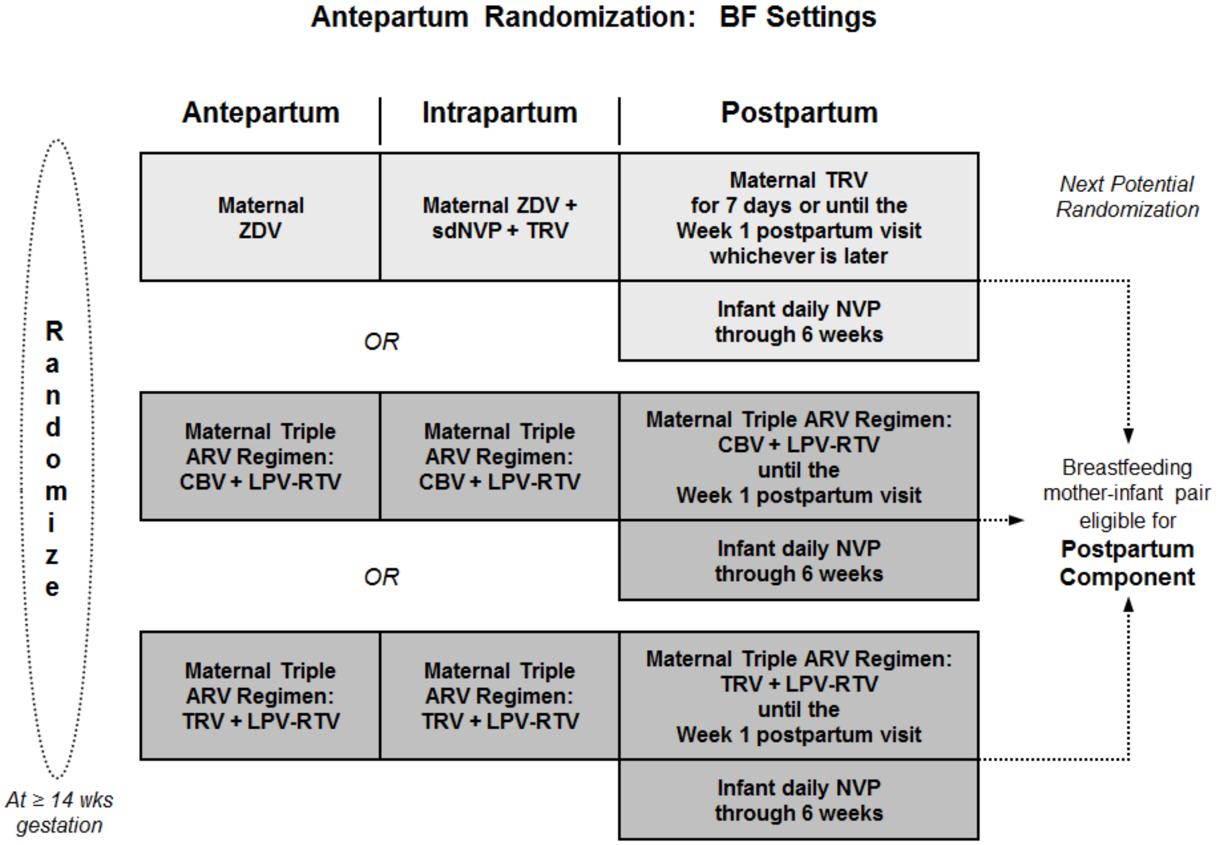

2.1 Rationale (Antepartum Component)

An important research issue that requires further investigation is determination of the optimal ARV prophylaxis for PMTCT among healthy women with high CD4 cell counts. Among HIV-infected pregnant women with higher CD4 counts (> 350 cells/mm^3), it is currently unclear whether triple ARV prophylaxis regimens will be safe and significantly reduce antepartum and intrapartum/early postpartum transmission when compared to less complex ZDV plus single dose intrapartum interventions. The Antepartum Component of PROMISE will address this question. A pre-entry CD4 of $\geq$ 350 cells/mm^3 was chosen based on recently updated guidance to initiate treatment in this population in adults (1, 2).

Routine use of triple ARV prophylaxis has been implemented for PMTCT in resource-richer countries as the standard of care and PMTCT rates of under 2% have been reported based on observational data (3-5). In settings with greater resource limitations, the World Health Organization (WHO) 2010 guidelines recommend either daily maternal ZDV and sdNVP or triple ARV prophylaxis beginning as early as 14 weeks gestation plus infant prophylaxis for six weeks after birth and – in breastfeeding settings – either continued maternal or infant prophylaxis through breastfeeding cessation for PMTCT for HIV-infected pregnant women who do not yet require ARV treatment for their own care, despite acknowledged limitations in direct evidence regarding some aspects of these recommendations (1). In studies of ZDV (in some cases with 3TC added) with sdNVP, transmission rates of 1.1-3.9% at six weeks of age have been reported, even when including all pregnant women regardless of CD4 lymphocyte count (6-8). In several studies in developing countries where triple ARV regimens were provided for all pregnant women regardless of CD4 lymphocyte count, including DREAM, AMATA, and Mitra-plus, transmission rates at four to six weeks of age ranged from 1.2-4.1% among BF infants. In the Kisumu Breastfeeding Study

(KiBS), the transmission rate at six weeks among women with CD4 lymphocyte counts > 250 cells/mm^3 who received triple ARV prophylaxis was 3.8% and at 12 months was 5.5% (9). While it is difficult to compare data between studies because of differences in populations, BF rates and duration, ARVs available, and obstetrical management, the reported transmission rates with ZDV + sdNVP and triple ARV prophylaxis were similar in these studies conducted in resource limited settings.

In addition, potential triple ARV prophylaxis regimen-related toxicity among women who do not yet require triple ARV treatment for their own care and their ARV-exposed infants is a concern in settings with minimal laboratory monitoring available. In a study from Germany, HAART regimens used during pregnancy were associated with a 2.22-fold (95% CI 1.06-4.64) increased risk of anemia and a 2.15-fold (95% CI 1.02-4.55) increased risk of neutropenia in infants compared to infants born to women receiving single or double nucleoside analogue reverse transcriptase inhibitor (NRTI) regimens in pregnancy (10). In the Women and Infants Transmission Study (WITS), infants born to women who received HAART during pregnancy were associated with larger decreases in neutrophils and lymphocytes compared to infants exposed to a single drug prophylaxis regimen during pregnancy. Anemia and neutropenia may be more common among women and infants in low resource settings in the absence of ARV therapy, and these complications may be magnified by triple ARV treatment use. Severe hepatotoxicity with NVP-based HAART has been reported in pregnant women with high CD4 lymphocyte counts in Mozambique as well as in the US and Canada (11-13).

Another concern with widespread use of triple ARV prophylaxis regimens during pregnancy is the potential for an increase in pregnancy complications, specifically preterm birth. Studies from Europe have consistently shown an increased risk of preterm delivery among women receiving combination regimens including protease inhibitor agents, especially starting before pregnancy, while US data have generally not shown such an increase (14). Data from Cote d'Ivoire found an increased risk of low birth weight among women receiving triple ARV regimens with NVP of 22.3% compared to 9.4% with ZDV + sdNVP and 12.3% with 3TC-ZDV + sdNVP (15). Longer duration of triple ARV prophylaxis was associated with an increased risk. Other pregnancy complications which must be monitored and compared between women exposed to triple ARV prophylaxis and those exposed to less complicated ARV regimens include stillbirth and pre-eclampsia. In a study in Botswana of 9,504 HIV-infected pregnant women who delivered between 2009 and 2011 at 6 government hospitals, receipt of triple drug regimens (regardless of regimen) was independently associated with preterm delivery, small for gestational age, and stillbirth compared to births to HIV-infected women on no drugs and those receiving ZDV alone during pregnancy, particularly in women with CD4 count > 200 cells/mm^3 (16).

The inclusion of two different triple prophylaxis regimen arms will also allow for comparison of maternal and infant safety outcomes related to drugs included in the WHO-recommended regimens for use during pregnancy and breastfeeding. Also, because there are limited data available specifically regarding the safety of TDF use in pregnancy for the mother and the infant, PROMISE will co-enroll women and infants in a substudy called IMPAACT P1084s that will compare potential TDF toxicity endpoints (bone and renal) in women and their infants exposed to TDF during pregnancy those women and infants who were not exposed to TDF during pregnancy.

With widespread use of triple ARV prophylaxis regimens during pregnancy, the effects of stopping these ARV regimens on maternal health are also concerns as data from SMART and other trials suggest harm from structured treatment interruption (17). The data regarding risks of stopping triple ARV regimens are discussed more fully in the maternal health section of the protocol (Section 5.0). An additional concern is potential mitochondrial toxicity in the infant. Mitochondrial toxicity has been described in both adults and children exposed to nucleoside agents, and combination therapy may increase this rare but serious risk (18-22).

Finally, the cost of implementing triple ARV prophylaxis for use among women with CD4 counts above current thresholds for treatment is an important consideration. Obtaining data on the comparative efficacy of triple ARV prophylaxis and a less complex ZDV/sdNVP regimen for PMTCT will inform policy decisions regarding these interventions. Modeling of cost effectiveness is an important component of this trial.

While the risk of PMTCT has been shown to be reduced to 1-2% or less in high resource settings, triple ARV prophylaxis as standard of care in these settings has been adopted without direct comparison to ZDV + sdNVP regimens, and without evaluation of the safety of triple ARV prophylaxis discontinuation following delivery in women who do not require therapy. In resource-limited settings, transmission rates have been similar in observational studies among women receiving ZDV + sdNVP and those receiving triple ART prophylaxis. Given the increased expense, both in drug and monitoring costs, potential increased toxicity, potential for adverse pregnancy outcomes, and uncertain long-term effects on maternal and infant health with triple ARV prophylaxis use, the potential benefits of triple ARV prophylaxis on PMTCT and maternal health must be carefully compared to outcomes with the current standard of ZDV+ sdNVP.

Rationale for Use of Antenatal ZDV plus sdNVP Regimen for Women Who Do Not Require Antiretroviral Treatment for Their Own Health

This regimen was chosen for the comparison arm of the antenatal randomization, based on current WHO recommendations for HIV-infected pregnant women with higher CD4 counts, who do not require ARV treatment for their own health. Antepartum ZDV has been shown to be efficacious compared to placebo and has a favorable third trimester safety profile based on short course trials from Thailand, west and southern Africa (23-25). In the HIVNET 012 trial, sdNVP given at the onset of labor and to the newborn was shown to be highly efficacious compared to an ultra short course of ZDV given at labor and to mothers and newborns for one week post-delivery. As discussed above, the combination of short course ZDV + sdNVP has resulted in transmission rates of 1.1-3.9% at four to six weeks of age in both FF and BF settings (6-8).

However, a concern with use of the ZDV + sdNVP regimen for women with lower CD4 counts is that sdNVP has the potential for development of NVP resistance, and such resistance may in turn increase the risk of virologic treatment failure if treatment is started within the first 6-12 months following delivery. Several studies have subsequently tested whether continuing women on up to a week of postpartum nucleoside ARVs to help cover the long drug half-life of NVP will lessen the risk of development of NVP resistance.

Rationale for Use of Tenofovir disoproxil fumarate (TDF)-Emtricitabine (FTC) (Truvada, TRV) "Tail" to Reduce the Risk of Resistance Following sdNVP

Because development of NVP resistance following sdNVP is associated with low maternal CD4 lymphocyte count and the women enrolled in PROMISE will all have CD4 count $\geq$ 350 cells/mm^3 at study entry, the women in PROMISE will be less likely to acquire drug resistance than women who have lower CD4 cell counts.

Available data suggest that ARV drugs used in addition to sdNVP reduce the development of resistance following sdNVP exposure. For example, use of ZDV + sdNVP results in lower rates of NVP resistance than use of sdNVP alone. Likewise, data from S. Africa using 3TC-ZDV for 3-7 days following intrapartum sdNVP also reduced the rate of NVP resistance at 2-6 weeks postpartum from 60% with sdNVP without the 7 day tail to 10% with the tail (26). Data from Zambia indicate that combining an

intrapartum dose of TDF + FTC in the fixed dose formulation TRV with short course ZDV + sdNVP reduces NVP resistance from 25% to 12%, a 53% reduction (27).

Additional data are available from the TEmAA Study/ANRS 12109, which enrolled 38 pregnant women from Cote d'Ivoire, Vietnam, and S. Africa with median CD4 cell count at enrollment of 350 cells/mm^3 (intraquartile range 314-596) (28). In this study, all women received ZDV starting at 28 weeks gestation combined with sdNVP; TRV was given intrapartum and continued daily for 1 week postpartum. No ARV drug resistance to ZDV, NVP, TDF or FTC was observed at 4 weeks postpartum. This lack of resistance with the 7 day TRV "tail" was the primary reason for choosing the TRV regimen over single dose TRV or 7 days of a 3TC-ZDV "tail." Thus, all women in the PROMISE study who receive ZDV with intrapartum sdNVP will also receive an intrapartum dose of TRV, followed by 1 week of daily TRV or the date of the week 1 visit (up to 14 days), whichever is later (this regimen is subsequently referred to as ZDV + sdNVP + TRV tail). The risk of NVP resistance subsequent to receipt of the ZDV + sdNVP + TRV tail regimen will be examined in a subset of the women and their infants randomized in the AP Component to Step 1 Arm A.

Infant ARV Prophylaxis

All infants born to women enrolled in the study, regardless of maternal randomization arm, will be provided NVP through six weeks (42 days) of age, unless determined to be HIV-infected.

Antepartum ARV Prophylaxis Regimens Chosen for PMTCT

All women will be screened for hepatitis B virus (HBV) infection prior to study entry.

HIV-infected women without HBV co-infection

In PROMISE, HIV-infected women who are not co-infected with HBV (i.e., those who have a negative HBsAg at screening) will be randomized to one of three regimens: ZDV + sdNVP + TRV tail, 3TC-ZDV (Combivir, CBV) and Lopinavir (LPV)-Ritonavir (RTV) (Aluvia, Kaletra), or FTC-TDF (Truvada)/LPV-RTV.

The choices of the specific agents used for the antenatal triple ARV prophylaxis regimens for this protocol were decided by the 1077 study team based on known safety profile of the ARVs, potency and ease of administration.

While the use of protease inhibitor (PI) based triple ARV regimens are generally reserved for second line therapy in resource limited international settings, PI-based triple ARV prophylaxis regimens were chosen for use in PROMISE among women with high CD4 counts based on the following considerations:

- The use of nonnucleoside reverse transcriptase inhibitors (NNRTIs) is not an option due to safety concerns with use of NVP among women with higher CD4 counts, the group who will enroll in PROMISE, and teratogenicity concerns with efavirenz (EFV) use during pregnancy.

- A triple nucleoside reverse transcriptase inhibitor (NRTI), single-drug class regimen was not chosen given the lack of safety or efficacy data on use of NRTIs for PMTCT. Another concern regarding use of triple NRTIs is that randomized clinical trial data in non-pregnant HIV-infected adults have shown that the triple nucleoside regimen of 3TC-ZDV/abacavir (ABC) had significantly lower virologic efficacy than dual-class HAART (e.g., NNRTI or PI-based regimens), and therefore a triple NRTI regimen is neither a preferred nor alternative therapy choice for treatment of adults in resource-rich settings such as the US (29).

- Available safety, adherence and tolerability data with PI-based regimens among women with higher CD4 counts in several ongoing trials in Africa are favorable, as is ongoing clinical experience in the US.

HIV-infected Women Co-infected with HBV

HBV co-infection is common, affecting 10% of HIV-infected individuals in resource-limited settings. Although the impact of HIV disease on HBV co-infection has been studied in non-pregnant adults, little is known about the effects of HIV on HBV during pregnancy. In many resource-limited settings, HBV screening is not available and HIV/HBV co-infected pregnant women receive various regimens of HBV-active PMTCT regimens. ARV drugs with anti-HBV activity include 3TC, FTC and TDF. Thus, HIV/HBV co-infected pregnant women may receive regimens that do not include any HBV active drugs, regimens that contain only a single HBV active drug (e.g., 3TC), or regimens that contain two HBV active drugs (e.g., FTC-TDF). Despite the use of ARV regimens that may impact HBV disease, little is known about their impact on HBV-disease specific outcomes, such as the incidence of HBV resistance, the incidence of MTCT of HBV and the maternal safety of HAART regimens with a single HBV-active drug (3TC-ZDV) compared to two HBV-active drugs (TDF/3TC or FTC-TDF).

Although US and WHO guidelines recommend the use of two drugs active against HBV in co-infected patients starting ART, this standard has not been routinely applied to pregnant women. Specifically, uncertainty about the safety of TDF in pregnancy has limited the widespread use of dual HBV-active ARV therapy in this setting. However, because TDF is an HBV-active drug, it may be more beneficial for HIV/HBV co-infected pregnant women to receive TDF as well as 3TC or FTC during pregnancy.

The PROMISE study provides an unparalleled opportunity to examine drug safety and maternal and infant HBV outcomes with routinely administered PMTCT interventions in resource-limited settings. Women with HIV/HBV co-infection will be randomized to one of three regimens: ZDV + sdNVP + TRV tail, 3TC-ZDV/LPV-RTV or FTC-TDF/LPV-RTV. These are the same regimens to which women without HBV co-infection will be assigned. All follow-up evaluations will be identical for women with and without HBV co-infection. A brief overview of the substudy planned for these subjects may be found in Section 1.4 and additional detail can be found in Appendix VII.

2.11 Clinical Experience with and Safety of the PROMISE Study Drugs

Information regarding use of these drugs in pregnancy can also be found in the USPHS Task Force "Recommendations for Use of Antiretroviral Drugs in Pregnant HIV-Infected Women for Maternal Health and Interventions to Reduce Perinatal HIV Transmission in the United States," http://AIDSinfo.nih.gov. Additional information on each of these drugs is available in the most recent package inserts and/or investigator brochures.

Lamivudine-Zidovudine (3TC-ZDV, Combivir, CBV)

Note: Refer to the most recent package inserts for additional detail and updated information.

Lamivudine-Zidovudine (3TC-ZDV) as Combivir has been used extensively in pregnancy as part of a number of Phase I through Phase III perinatal trials in the U.S, Europe and Africa (PACTG 354, 386, 353, 358, 316, ANRS 075, PETRA, SAINT, KiBS) and in clinical practice (3, 8, 9, 14, 30-35). There has likewise been increasing experience with use of LPV-RTV during pregnancy in the US and Europe settings; as well as Phase I safety data; and also some experience now in an ongoing multisite trial, Kesho Bora, taking place in East, West and Southern Africa; and a trial in Botswana.

3TC-ZDV has been widely used for both treatment and as part of PMTCT regimens during pregnancy. The two NRTIs ZDV and 3TC are generally well tolerated with the anticipated and generally mild toxicities of anemia and neutropenia well described. Hepatic transaminase elevations may occur, and rarely life threatening hepatic steatosis and mitochondrial dysfunction have been described.

ZDV was shown to be safe and effective in the PACTG 076 trial with the most common side effect being reversible anemia. ZDV is the only drug approved by the US FDA for PMTCT and has been the backbone of antenatal regimens used for PMTCT both in resource rich as well as resource limited settings. High level resistance with ZDV is rare when used short term during pregnancy for PMTCT as multiple mutations are required before high level ZDV resistance occurs. Over 12,200 cases of use of ZDV in pregnancy have been reported to the Antiretroviral Pregnancy Registry (APR) with nearly 3,800 first trimester exposures without evidence of an increased risk of birth defects (36).

3TC is a potent and generally well tolerated NRTI used widely as part of HAART regimens. Although 3TC is an effective NRTI, virus with a resistance mutation at codon 184 rapidly emerges within 2 weeks of monotherapy and ~40% resistance is seen within 8 weeks (29). Resistance is also seen with dual nucleoside regimens within 4-8 weeks. Adverse events occur in less than 5% of patients. Side effects include headache, nausea and vomiting, malaise, fatigue and sleeplessness, anorexia, dizziness, rash, depression, anemia, neutropenia, and hyperamylasemia. Over 10,700 cases of use of 3TC in pregnancy have been reported to the Antiretroviral Pregnancy Registry with over 4,000 first trimester exposures without evidence of an increased risk of birth defects (36).

The pharmacokinetics, safety profile and activity of combination 3TC-ZDV used for PMTCT during pregnancy has been evaluated in a number of studies including the phase II ANRS 075 trial, as well as the phase I trials PACTG 353, 354, 358, 386, and has also been used in resource-limited countries as part of HAART regimens in pregnant women (3, 8, 9, 14, 30, 31, 34, 35). 3TC-ZDV was well tolerated in these trials. General side effects were those known to be related to ZDV and 3TC. Both ZDV and 3TC are FDA Pregnancy Class C.

3TC and HBV Infection

Exacerbations of HBV have been reported in patients after discontinuation of 3TC (37, 38). Patients who are co-infected with HBV may have increased values on liver function tests and exacerbation of hepatitis symptoms when 3TC is stopped. Usually these symptoms are self-limiting; however, serious complications have been reported. The causal relationship to 3TC discontinuation is unknown. Patients co-infected with HBV and HIV should be closely monitored with both clinical and laboratory assessments follow-up for several months after stopping 3TC treatment.

Lopinavir-Ritonavir (LPV-RTV, Kaletra, Aluvia)

Note: Refer to the most recent package inserts for additional detail and updated information.

Lopinavir (LPV, ABT-378) is a potent inhibitor of HIV protease. When co-formulated with LPV, ritonavir (RTV) inhibits the CYP3A-mediated metabolism of LPV, thereby providing increased plasma levels of LPV. LPV-RTV in a single fixed-dose combination capsule was evaluated and approved by the US Food and Drug Administration (FDA) in 2000 for use in combination with other ARVs for the treatment of HIV infection. A tablet formulation of LPV-RTV received FDA approval in October 2005. Kaletra and Aluvia are both forms of Lopinavir that are marketed in different areas of the world; the package inserts and safety information for one apply to both.

LPV-RTV has been studied in non-pregnant patients as combination therapy in Phase I/II and Phase III trials, and shown to be highly efficacious and potent with a favorable tolerability and safety profile.

A Phase III study (M98-863) evaluated the safety and efficacy of LPV-RTV plus stavudine (d4T) and 3TC versus nelfinavir (NFV) plus d4T and 3TC in treatment-naïve patients (39). The primary efficacy analyses included the proportion of participants with HIV RNA level < 400 copies/mL at week 24 and the duration of virologic response through week 48. Overall, 326 participants were assigned to the LPV-RTV group and 327 to the NFV group. Baseline HIV RNA level was 4.9 log_{10} copies/mL for each group. Baseline CD4 cell counts were approximately 260 cells/mm^3 for each group. At 48 weeks, the proportion of participants with HIV RNA levels < 400 (< 50) copies/mL by intent to treat (ITT) (missing value = failure, M = F) analysis were 75% (67%) for the LPV-RTV group compared with 63% (52%) for the NFV group (p<0.001) [proportion < 400 (< 50) copies/mL in the "on treatment" analysis was 93% (83%) versus 82% (68%), respectively]. Mean changes in CD4 cell counts were +207 cells/mm^3 for the LPV-RTV group and +195 cells/mm^3 for the NFV group. Durability of response has been demonstrated with LPV-RTV in ARV-naive patients in the above study with 79% of the 326 participants on the LPV-RTV arm maintaining virologic suppression (viral load of < 400 copies/mL) at 96 weeks, compared with 58% on the NFV arm.

LPV-RTV has been studied in combination with TDF and FTC. Study 418 is a randomized, open-label, multicenter trial comparing treatment with LPV-RTV 800 mg/200 mg once-daily plus FTC-TDF versus LPV-RTV 400 mg/100 mg twice-daily plus FTC-TDF in 190 antiretroviral treatment-naïve patients. Patients had a mean age of 39 years (range: 19 to 75), 54% were caucasian, and 78% were male. Mean baseline CD4 cell count was 260 cells/mm^3 and mean baseline plasma HIV RNA was 4.8 log_{10} copies/mL. Through 48 weeks of therapy, 71% in the LPV-RTV once-daily arm and 65% in the LPV-RTV twice-daily arm achieved and maintained HIV RNA < 50 copies/mL (95% confidence interval for the difference, -7.6% to 19.5%). Mean CD4 cell count increases at Week 48 were +185 cells/mm^3 for the LPV-RTV once-daily arm and +196 cells/mm^3 for the LPV-RTV twice-daily arm.

LPV-RTV has been studied as combination therapy in Phase I/II and Phase III trials. The most common AEs associated with LPV-RTV therapy were diarrhea and nausea, which were generally of mild-to-moderate severity. Rates of discontinuation of randomized therapy due to AEs were 5.8% in LPV-RTV-treated and 4.9% in NFV-treated patients in study M98-863. Pancreatitis has been reported in patients receiving LPV-RTV, although a causal relationship has not been established. The most common laboratory abnormalities in patients receiving LPV-RTV were elevations in triglycerides and cholesterol, which may be marked, and less commonly elevations in AST and ALT.

Recent information described effects on electrocardiogram. QTcF interval was evaluated in a randomized, placebo and active (moxifloxacin 400 mg once-daily) controlled crossover study in 39 healthy adults (M06-809), with 10 measurements over 12 hours on Day 3. The maximum mean time-matched (95% upper confidence bound) differences in QTcF interval from placebo after baseline-correction were 5.3 (8.1) and 15.2 (18.0) mseconds (msec) for 400 mg/100 mg twice-daily and supratherapeutic 800 mg/200 mg twice-daily lopinavir/ritonavir, respectively. Lopinavir/ritonavir 800 mg/200 mg twice daily resulted in a Day 3 mean Cmax approximately 2-fold higher than the mean Cmax observed with the approved once daily and twice daily lopinavir/ritonavir doses at steady state.

PR interval prolongation was also noted in subjects receiving lopinavir/ritonavir in the same study on Day 3. The maximum mean (95% upper confidence bound) difference from placebo in the PR interval after baseline-correction were 24.9 (21.5, 28.3) and 31.9 (28.5, 35.3) msec for 400 mg/100 mg twice-daily and supratherapeutic 800 mg/200 mg twice-daily lopinavir/ritonavir, respectively.

Additional information can be found in the most recent Kaletra or Aluvia package inserts, which state that lopinavir/ritonavir prolongs the PR interval in some patients and should be used with caution in patients who have preexisting structural heart disease, conduction system abnormalities, or other cardiac diseases. Lopinavir/ritonavir should be used with caution and with clinical monitoring in patients who are also using other drugs that prolong the PR interval, such as atazanavir, digoxin, beta blockers or calcium channel blockers. First-, second-, and third-degree atrioventricular block, QTc interval prolongation, and torsade de pointes have been observed in clinical trials and in postmarketing reports. The product label specifically recommends avoiding use in patients with congenital long QT syndrome, those with hypokalemia, and with other drugs that prolong the QT interval.

LPV-RTV in pregnancy

LPV-RTV is classified as FDA pregnancy category C. Placental passage of LPV and RTV is limited (40, 41). There has been no evidence of teratogenicity with administration of LPV-RTV to pregnant rats or rabbits. In rats treated with maternally toxic dosages (100 mg LPV-50 mg RTV/kg/day), embryonic and fetal developmental toxicities (early resorption, decreased fetal viability, decreased fetal body weight, increased incidence of skeletal variations and skeletal ossification delays) were observed. In rabbits, no embryonic or fetal developmental toxicities were observed with maternally toxic dosage, where drug exposure was 0.6-fold for LPV and 1.0-fold for RTV of the exposures in humans at recommended therapeutic dose. In the Antiretroviral Pregnancy Registry, sufficient numbers of first trimester exposures to LPV-RTV have been monitored to be able to detect at least a 2-fold increase in risk of overall birth defects. No such increase in birth defects has been observed with LPV-RTV. The prevalence of birth defects with first trimester LPV-RTV exposure was 2.4% (21 of 883; 95% CI, 1.5%–3.6%) compared with total prevalence of birth defects in the US population based on CDC surveillance of 2.7% (36).

LPV-RTV has been used in a multi-site efficacy trial, the Kesho Boro trial currently taking place in Africa with exposure from 28 weeks forward and postpartum up to six months of BF (42). It was also used in the Mma Bana PMTCT trial in Botswana (43).

The safety and pharmacokinetics of LPV-RTV in pregnancy have been evaluated in several studies; including studies of the capsule formulation and the new tablet formulation of LPV 200 mg-RTV 50 mg that is heat stable and does not have a food requirement. The pharmacokinetics of LPV-RTV capsules were evaluated in the second and third trimester of pregnancy in protocol P1026s. At standard adult capsule doses (3 LPV 133 mg-RTV 33 mg capsules twice daily), LPV levels during the third trimester were significantly lower compared to postpartum levels and those in nonpregnant adults (40). Only 3 (18%) of 17 women evaluated during the third trimester had LPV area under the curve (AUC) concentrations above the 10th percentile for non-pregnant adults, and none exceeded the 50th percentile; in contrast, 79% of these women evaluated postpartum had AUC values above the 10th percentile. As with RTV, placental passage of LPV was limited.

Increasing the dose of LPV-RTV in the third trimester to 4 capsules twice daily provided adequate LPV exposure during the third trimester, but resulted in higher levels by 2 weeks postpartum (44). However, a separate study in London of 16 pregnant HIV-infected primarily ARV-naïve women receiving standard dosing of LPV-RTV capsules throughout pregnancy found that the median trough level of LPV in the third trimester was 3,660 ng/mL and that 94% had trough levels >1,000 ng/mL (the minimum trough required to inhibit wild-type HIV); 14 (88%) of 16 women had virologic suppression (45). Data for AUC were not provided, so these data are not comparable with P1026s data. These investigators suggested therapeutic drug monitoring during the third trimester to determine if an increased dose would be required for the capsule formulation.

The tablet is the currently available formulation of LPV-RTV. Plasma concentrations of LPV and RTV after administration of two 200 mg/50 mg LPV-RTV tablets in nonpregnant patients are similar to those achieved with three LPV 133 mg-RTV 33 mg capsules given with food, but with less pharmacokinetic variability. In a study of 36 pregnant women, trough plasma LPV levels were measured during the second trimester in 23 women and third trimester in 19 women; trough levels were adequate with standard dosing (400 mg/100 mg twice daily) of the tablet formulation (46). Three women had trough levels below the target but were noted to have had adherence problems.

Data from P1026s evaluating standard dosing of the new LPV-RTV tablet formulation (2 tablets twice daily) until 30 weeks gestation, followed by an increase to 3 tablets twice daily until postpartum hospital discharge, when return to standard dosing occurs, showed that five of six women on standard dosing in the second trimester attained the target AUC, although the AUC was 50% lower than postpartum levels (47). The AUC target was attained in 19 of 21 women on the increased dose in the third trimester. All women met the AUC target on standard dosing in the early postpartum period. A study of standard doses of LPV-RTV (400 mg/100 mg twice daily as capsules) started during labor and continued postpartum demonstrated all women exceeding target AUC at 72 hours and 30 days postpartum, suggesting that standard LPV-RTV dosing is appropriate immediately postpartum (48). Based on these data, an increased dose of three tablets of LPV-RTV twice daily during the third trimester with reduction to the standard dose of two tablets twice daily immediately postpartum has been selected for use in this study.

Once daily dosing of LPV-RTV capsules or tablets is not recommended in pregnancy, as there are no data to address whether drug levels are adequate with such administration.

Emtricitabine and Tenofovir Disoproxil Fumarate (FTC and TDF)

Note: Refer to the most recent package inserts for additional detail and updated information.

TDF and FTC (as the combined formulation Truvada, TRV) will be used as one of the antenatal randomization arm regimens for pregnant women.

Emtricitabine (FTC, Emtriva™)

Emtricitabine (FTC) (5-fluoro-1-(2R,5S)-[2-(hydroxymethyl)-1,3-oxathiolan-5-yl]cytosine) is a synthetic nucleoside analogue with activity against HIV reverse transcriptase. FTC is the negative (-) enantiomer of a thio analogue of cytidine, which differs from cytidine analogues in that it has a fluorine in the 5-position. FTC is phosphorylated by cellular enzymes to form the active intracellular metabolite, emtricitabine 5'-triphosphate (FTC-TP), which is a competitive inhibitor of HIV RT and terminates the growing DNA chain.

Two Phase III controlled studies (FTC-301A, and FTC-303) provide the most information concerning the safety and efficacy of FTC in HIV-infected adults treated for extended periods with combinations of ART (49).

Study FTC-301A was a 48 week, double-blind, active-controlled, multicenter study comparing FTC (200 mg) once daily to d4T in combination with once daily open-label didanosine (ddI) and EFV in 571 ARV-naïve patients with plasma HIV RNA > 5,000 copies/mL. Patients had a mean age of 36 years (range 18 to 69), 85% were male, 52% Caucasian, 16% African American and 26% Hispanic. Patients had a mean baseline CD4 cell count of 318 cells/mm^3 (range 5-1317) and median baseline plasma HIV RNA of 4.9 $\log_{10}$ copies/mL (range 2.6-7.0). Thirty-eight percent of patients had baseline viral loads >100,000 copies/mL and 31% had CD4 cell counts < 200 cells/mm^3.

At week 48, FTC was statistically superior to d4T with 81% of the patients in the FTC treatment group achieving and maintaining plasma HIV RNA <400 copies/mL compared with 68% of the patients in the d4T treatment group. Likewise, the proportion of patients who achieved and maintained plasma HIV RNA <50 copies/mL was statistically significantly different with 78% of patients in the FTC treatment group compared with 59% of patients in the d4T treatment group. Additionally, FTC-treated patients had a statistically greater increase in CD4 cell count at Week 48 with a mean increase from baseline of +168 cells/mm^3 for the FTC group and +134 cells/mm^3 for the d4T group. The proportion of patients with virologic failure was 3% in the FTC group and 11% in the d4T group. A statistically greater proportion of patients in the d4T group experienced an adverse event (AE) that led to study drug discontinuation through Week 48 than in the FTC group (13% versus 7%).

Study FTC-303 was a 48 week, open-label, active-controlled, multicenter study comparing FTC to 3TC in combination with d4T or ZDV and a protease inhibitor (PI) or NNRTI in 440 patients who were on a 3TC-containing triple-ARV regimen for at least 12 weeks prior to study entry and had plasma HIV RNA $\leq$ 400 copies/mL (49). Patients were randomized 1:2 to continue therapy with 3TC (150 mg BID) or to switch to FTC (200 mg QD). All patients were maintained on their stable background regimen. Patients had a mean age of 42 years (range 22-80); 86% were male, 64% Caucasian, 21% African American, and 13% Hispanic. Patients had a mean baseline CD4 cell count of 527 cells/mm^3 (range 37-1,909) and median baseline plasma HIV RNA of 1.7 $\log_{10}$ copies/mL (range 1.7-4.0). The median duration of prior ART was 27.6 months.

Through 48 weeks of therapy, there was no statistically significant difference between treatment groups in efficacy outcomes. The proportion of patients with virologic failure was 7% in the FTC arm and 8% in the 3TC arm. Through 48 weeks of therapy, the proportion of patients who achieved and maintained plasma HIV RNA < 400 copies/mL was 77% in the FTC arm and 82% in the 3TC arm. The difference was largely attributed to attrition from the study and not loss of virological activity. Likewise, the proportion of patients who achieved and maintained plasma HIV RNA < 50 copies/mL was 67% in the FTC arm and 72% in the 3TC arm. The mean increase from baseline in CD4 cell counts was +29 cells/mm^3 in the FTC arm and +61 cells/mm^3 in the 3TC arm. These findings support equivalent efficacy of FTC 200 mg once-daily and 3TC 150 mg administered twice daily (50).

More than 2,000 adult patients with HIV infection have been treated with FTC alone or in combination with other ARVs for periods of 10 days to 200 weeks in Phase I-III clinical trials. Assessment of adverse events is based on data from studies FTC-301A and FTC-303 in which 571 treatment naïve (FTC-301A) and 440 treatment experienced (FTC-303) patients received FTC 200 mg (n=580) or comparator drug (n=431) for 48 weeks.

The most common adverse events that occurred in patients receiving FTC with other ARVs in clinical trials were headache, diarrhea, nausea and rash event, which were generally mild to moderate in severity. Approximately 1% of patients discontinued participation in the clinical studies due to these events. All adverse events were reported with similar frequency in FTC and control treatment groups with the exception of skin discoloration, which was reported with higher frequency in the FTC-treated group. Skin discoloration, manifested by hyperpigmentation on the palms and/or soles was generally mild and asymptomatic. Laboratory abnormalities in these studies occurred with similar frequency in the FTC and comparator groups.

FTC in Pregnancy

FTC is classified as FDA pregnancy category B. Fetal variations and malformations were not increased with FTC dosing in mice in systemic drug exposures that were 60 times higher than doses recommend in humans (51). In the Antiretroviral Pregnancy Registry, sufficient numbers of first-trimester exposures to

emtricitabine in humans have been monitored to be able to detect at least a 2-fold increase in risk of overall birth defects. No such increase in birth defects has been observed with emtricitabine. The prevalence of birth defects with first trimester FTC exposure was 2.3% (21 of 899 births; 95% CI, 1.4%–3.5%) compared with a 2.7% total prevalence in the U.S. population, based on CDC surveillance (36).

FTC crosses the placenta in mice and rabbits with average fetal/maternal drug concentration ratios of 0.4 in mice and 0.5 in rabbits. In a study of 35 pregnant women given a dose of 400 mg FTC at the onset of labor, median cord/maternal drug ratio was 0.73, indicating significant placental transfer. Median AUC after a 400 mg dose in labor was 15.5 mg*h/L, similar to levels in nonpregnant adults after a 200 mg dose. No data are currently available on levels of FTC in human breast milk. Among 18 women receiving standard FTC dosing (200 mg/day) during the third trimester, median AUC of 8.6 µg*h/mL was above the target of > 7 µg*h/mL, but only 12 of 18 women were above the target (47). Mean cord/maternal blood ratio at delivery was 1.17.

FTC and HBV Infection

Exacerbations of HBV have been reported in patients after discontinuation of FTC (52). Patients, who are co-infected with HBV, may have increased values on liver function tests and exacerbation of hepatitis symptoms when FTC is stopped. Usually these symptoms are self-limiting; however, serious complications have been reported. The causal relationship to FTC discontinuation is unknown. Patients co-infected with HBV and HIV should be closely monitored with both clinical and laboratory assessments follow-up for several months after stopping FTC treatment.

Tenofovir Disoproxil Fumarate (TDF, Viread®)

Tenofovir disproxil fumarate (TDF), (9-[(R)-2-[[bis[[(isopropoxycarbonyl)oxy]methoxy]phosphinyl] methoxy]propyl] adenine fumarate (1:1)) (formerly known as PMPA prodrug or GS-4331-05) is approved for the treatment of HIV infection. TDF is an orally bioavailable prodrug of tenofovir, an acyclic nucleotide analogue with activity in vitro against retroviruses, including HIV and HIV-2, and against hepadnaviruses. TDF is metabolized intracellularly to the active metabolite, tenofovir diphosphate (PMPApp), which is a competitive inhibitor of HIV reverse transcriptase that terminates the growing DNA chain. Although TDF is a nucleotide analogue, it has the same mechanism of action and resistance pattern as NRTIs. Therefore, for simplification of discussion, TDF will be referred to as an NRTI in this study.

Efficacy in Treatment Naïve Patients: Study 903 was a 144-week randomized, double-blind trial designed to compare the efficacy and safety of a treatment regimen of TDF, 3TC, and EFV to a regimen of d4T, 3TC and EFV in 600 ARV-naive subjects with HIV infection. Following the completion of the double blind portion of the trial, there was an additional 2 year single arm open-label portion of the trial in selected sites, wherein all patients received TDF, 3TC and EFV as once daily regimen. (Patients originally randomized to the d4T arm switched to receive TDF.)

In a 144-week analysis, when missing observations in the intent to treat (ITT) analysis were treated as having plasma HIV RNA concentrations greater than 400 copies/mL, 76% of subjects in the TDF group and 72% of subjects in the d4T active control group achieved plasma HIV RNA concentrations < 400 copies/mL. Plasma HIV RNA concentrations < 50 copies/mL at week 144 were seen in 73% and 69% of subjects in the TDF and d4T active control groups, respectively. The mean increases in CD4 cell count from baseline to week 144 were 263 cells/mm^3 and 283 cells/mm^3 for the TDF and d4T active control groups, respectively. The assessments of safety and tolerability indicate that the safety profile of TDF 300 mg/day was similar to that of the d4T active control (53).

FTC-TDF compared to 3TC-ZDV: Study 934 was a Phase III, randomized, open-label, multicenter study designed to compare a regimen of EFV with either TDF 300 mg/FTC 200 mg once daily or ZDV 300 mg/3TC 150 mg twice daily as fixed dose combination (FDC) Combivir (53). Interim analysis at 48 weeks revealed discontinuation occurred more frequently in the 3TC-ZDV group (9%) than FTC-TDF (4%), mostly because of adverse events such as anemia and nausea. The 48-week data demonstrated that using the time to loss of virologic failure as the primary analysis in which missing or switching is counted as a failure, the proportion of subjects with plasma HIV RNA levels less than 400 copies/mL in an ITT analysis (n=487) was 84% in the FTC-TDF group compared to 73% in the 3TC-ZDV-treated subjects (p=0.002). The proportion of subjects with plasma HIV RNA levels <50 copies/mL was 80% in the FTC-TDF group versus 70% in the 3TC-ZDV group (p=0.021). These results are supported by 96 week data (54).

Tenofovir and TDF administered in toxicology studies to rats, dogs, and monkeys at exposures (based on AUCs) between 6- and 12-fold higher than observed in humans caused bone toxicity. In monkeys, the bone toxicity was diagnosed as osteomalacia, and appeared to be reversible upon dose reduction or discontinuation of tenofovir. In rats and dogs, the bone toxicity manifested as reduced bone mineral density. The mechanism(s) underlying bone toxicity is unknown. Studies to assess loss of bone density among patients receiving tenofovir are described below.

More than 1,200 patients have received TDF 300 mg once daily alone or in combination with other ARVs in phase I-III clinical trials. Over 11,000 patients have received TDF in expanded access programs. The cumulative patient exposure to marketed TDF from first approval to 31 December 2003 is estimated to be approximately 200,000 patient-years of treatment.

In clinical trials in treatment-experienced patients (Studies 902 and 907), the safety profile of TDF 300 mg/day was similar to that of placebo. There were no clinically significant adverse events attributable to TDF 300 mg once daily other than a slightly higher incidence of mild to moderate gastrointestinal adverse events (nausea, diarrhea, vomiting and flatulence). Few adverse laboratory events were documented other than mild or moderate transient hypophosphatemia. Clinically significant events considered by the investigators to be related to TDF were uncommon and none suggested potential adverse drug reactions or drug-drug interactions (55, 56).

Study 910 was initiated to observe the long-term safety effects of TDF, in combination with other ARVs, in subjects who have completed prior TDF studies 901, 902 and 907. The long-term safety and tolerability of TDF were monitored using periodic assessments of concomitant medications, adverse events, serial laboratory tests and bone densitometry (in select subjects). A total of 687 subjects received TDF 300 mg either initially or through rollover. Long-term follow up shows that the incidence of adverse events or laboratory abnormalities leading to discontinuation of TDF remained low despite mean treatment duration of more than two years, and extending to nearly four years in some subjects. None of the adverse events or laboratory abnormalities that led to study drug discontinuation had a reported incidence of more than 1%. Furthermore, there was no indication of nephrotoxicity in this highly treatment-experienced population (57).

In Gilead study 903, TDF and d4T had comparable renal safety profiles with no patient in the TDF arm discontinuing the study for a renal-related abnormality and less than one percent of patients in each arm experiencing serum creatinine levels of more than 2 mg/dL. Toxicities that have been attributed to mitochondrial toxicity (peripheral neuropathy, lipodystrophy, and lactic acidosis) were reported in 100 patients, 83 (28%) of 301 in the d4T group and 17 (6%) of 299 in the TDF group (p<0.001). Neuropathy was observed in 31 (10%) of 301 and 9 (3%) of 299 patients in the d4T and TDF groups, respectively (p<0.001). Investigator-defined lipodystrophy was reported more often in patients receiving d4T than TDF (58 [19%] of 301 vs. 9 [3%] of 299, respectively; p<0.001).

Studies of TDF used in combination with lopinavir/ritonavir have shown varied results in terms of AUC concentration and creatinine clearance. Kearney and colleagues reported increased TDF exposure at steady state potentially related to increased TDF absorption but no clinical impact (58). In contrast, a study by Jullien revealed declines in TDF concentrations decreased for patients with no tubular dysfunction while they increased for those with dysfunction (59). In the CA Collaborative Treatment Group Study 578, patients on TDF + PI showed a greater decline in creatinine clearance compared to TDF + NNRTI regimen patients, but among TDF treated patients TDF plasma concentrations were not related to creatinine clearance.

Using whole body dual energy X-ray absorptiometry (DXA), significantly less total limb fat was observed in the d4T group at week 96 (7.9 kg TDF [n = 128] vs. 5.0 kg d4T [n = 134], $p<0.001$) and week 144 (8.6 kg TDF [n = 115] vs. 4.5 kg d4T [n = 117], $p<0.001$). Mean decreases in lumbar spine and hip bone mineral density after three years of treatment were less than three percent in both arms of the study. Bone mineral density reduction observed in Study 903 was non-progressive, with no substantial changes from the 24- and 48-week intervals to week 144. At 144 weeks, a total of five fractures were observed in the TDF arm compared to eleven fractures in d4T-treated patients.

TDF and HBV Infection

Exacerbations of HBV have been reported in patients after discontinuation of TDF (52). Patients who are co-infected with HBV may have increased values on liver function tests and exacerbation of hepatitis symptoms when TDF is stopped. Usually these symptoms are self-limiting; however, serious complications have been reported. The causal relationship to TDF discontinuation is unknown. Patients co-infected with HBV and HIV should be closely monitored with both clinical and laboratory assessments follow-up for several months after stopping TDF treatment.

TDF in Pregnancy

Chronic dosing of rats in pregnancy noted no growth or reproductive problems when TDF was administered at doses not associated with maternal toxicity. At high doses of exposure (25 times the AUC achieved with therapeutic dosing), no fetal structural changes were seen.

Chronic exposure of fetal monkeys to TDF at a high dose of 30 mg/kg (25 times the AUC levels achieved with therapeutic doses in humans) from days 20-150 of gestation did not result in gross structural abnormalities (60). However significantly lower fetal circulating insulin-like growth factor levels were reported and were associated with body weights 13% lower than untreated controls. A slight reduction in fetal bone porosity was also observed within 2 months of maternal treatment. However, a macaque treated for over 10 years with 10 mg/kg/day of TDF has given birth over several years to three infant macaques, all of whom were normal and had no bone abnormalities at birth (61).

TDF is designated as FDA pregnancy Category B based on animal and clinical data. In the Antiretroviral Pregnancy Registry, sufficient numbers of first trimester TDF exposures have been monitored to detect at least a two-fold increase in risk of overall birth defects but no such increase in birth defects has been observed. The prevalence of birth defects after first trimester TDF exposure was 2.3% (31 of 1,370 births; 95% CI, 1.5%-3.2%) which is within the range of congenital anomalies reported in the general US population (1).

Studies of intravenous TDF administration in pregnant cynomolgus monkeys reported a fetal/maternal concentration of 17% indicating some placental transfer (62). In three studies of pregnant women the cord-to-maternal blood ratio ranged from 0.60 to 0.99 indicating high placental transfer (63-65). A dose

of 600 mg of TDF in labor resulted in levels in the women similar to levels in non-pregnant adults after a 300 mg dose, suggesting higher doses are required for adequate levels during labor in term pregnant women (65). This was confirmed in PACTG 394 and HPTN 057, which showed adequate TDF concentrations with 600 mg intrapartum doses and a small increase in TDF concentrations when the intrapartum dose was increased to 900 mg (62, 66).

TDF pharmacokinetics during pregnancy among 19 pregnant women was assessed in P1026s in the last trimester between weeks 30-36 and also at 6-12 weeks post-delivery. The proportion of pregnant women with AUC exceeding the target of 2 μg hour/mL was slightly lower in the third trimester (74%) than postpartum (86%) but trough levels were comparable at both time points. A recent case series found TDF to be well tolerated among 76 pregnant women, with two stopping therapy, one for rash and one for nausea. All 78 infants were healthy with no signs of toxicity, and all were HIV-uninfected (67). No major toxicity signals have been reported despite increasing use of TDF in pregnant and lactating women worldwide. Limited studies have provided some reassuring data that TDF during pregnancy does not negatively impact human offspring bone or growth outcomes (68-70). However, the effects of prolonged maternal TDF use on pregnant/lactating women and their infants have not been adequately studied.

FTC and TDF Fixed Dose Combination Tablet (FTC-TDF, Truvada)

Gilead Sciences developed Truvada, a product containing FTC 200 mg and TDF 300 mg in a fixed-dose combination (FDC) tablet formulation that was approved by the US FDA on August 2, 2004. As a component of the New Drug Application, two Phase I studies evaluating the pharmacokinetics of co-administered FTC and TDF tablet formulation were completed.

Overall, Study GS-US-104-172 demonstrated bioequivalence between the FTC-TDF combination tablet and the FTC capsule and TDF tablet formulations when administered separately. Administration of the FTC-TDF combination tablet with either a high-fat meal or light meal increased tenofovir exposure by approximately 30% compared with fasted-state administration. Clinical experience with TDF indicates that the effect of food on tenofovir exposure is not of clinical relevance. FTC and TDF, either administered as a combination tablet (containing FTC 200 mg/ TDF 300 mg) or co-administered as FTC 200 mg capsule and TDF 300 mg tablet were well tolerated.

2.2 Study Objectives (Antepartum Component)

2.21 Primary Objectives

2.211 To evaluate the comparative efficacy of maternal antepartum triple ARV prophylaxis versus antepartum ZDV + sdNVP + TRV tail to reduce antepartum and intrapartum HIV transmission, as measured by the transmission rate through the Week 1 visit (6-14 days), when regimens are initiated $\geq$ 14 weeks gestation and prior to onset of labor

2.212 To assess and compare the safety and tolerability of the three ARV regimens, including adverse pregnancy outcomes (e.g., stillbirths, prematurity, low birth weight and congenital anomalies)

2.22 Secondary Objectives

2.221 To assess HIV transmission rates at birth by study arm

2.222 To assess 24-month HIV-free survival and overall survival in infants by maternal study arm (in conjunction with infants from the Postpartum Component)

2.223 To evaluate adherence to the maternal ARV regimens

2.224 To assess rates and patterns of maternal and infant resistance to the maternal and infant ARV strategies

2.225 To evaluate cost-effectiveness and feasibility of the trial ARV strategies

2.226 To assess rates of maternal suppression to HIV RNA < 400 copies/mL according to timing of ARV drug initiation before delivery

2.227 In HIV/HBV co-infected women, to compare the anti-HBV efficacy of two antepartum triple ARV prophylaxis regimens with single (3TC-ZDV) vs. dual (FTC-TDF) HBV-active drugs, assessed as change in hepatitis B viral load during the antepartum period, as well as several other HBV-specific outcomes (MTCT of HBV and HBV characteristics in babies, presence of HBV drug resistance, HBV virologic and biochemical changes after triple ARV prophylaxis cessation, and maternal anemia at delivery); see Appendix VII for additional details on the HBV substudy and its objectives.

2.3 Study Design (Antepartum Component)

This is a randomized, strategy trial to compare the efficacy and safety of triple ARV prophylaxis versus ZDV initiated at 14 weeks of pregnancy (or as soon as possible thereafter) plus sdNVP/Truvada (TRV) prophylaxis for the prevention of HIV *in utero* and intrapartum transmission in HIV-infected women with CD4 cell count $\geq$ 350 cells/mm^3 in BF (and FF) settings. HIV-infected women who do not need triple ARV therapy for their own health (and their unborn infants) will be randomized as outlined in Step 1 below. Should they subsequently need triple ARV therapy (HAART) for their own health, women will proceed to Step 2 (for first line therapy) and/or to Step 3 (for second line therapy).

1077BA Step 1: HIV-infected women who meet inclusion criteria (see Section 2.41), and their unborn infants, will be enrolled at $\geq$ 14 weeks gestation and prior to the onset of labor. Women will be randomized to one of three arms in a 1:1:1 ratio: ZDV + sdNVP + TRV tail (Step 1 Arm A), 3TC-ZDV/LPV-RTV (Step 1 Arm B) or FTC-TDF (TRV)/LPV-RTV (Step 1 Arm C).

1077BA Step 2: Mothers randomized to 1077BA Step 1 Arm A (ZDV + sdNVP + TRV tail), regardless of HBV status, who reach an indication for initiating HAART for their own health will be registered to 1077BA Step 2. Additionally, mothers randomized to 1077BA Step 1 Arm B or C (triple ARV prophylaxis) will be registered to 1077BA Step 2 if they reach an indication for triple ARV treatment for their own health while on the triple ARV prophylaxis regimen or after having stopped the triple ARV prophylaxis regimen. All mothers must complete a step change entry visit. For mothers not on a triple ARV regimen, the 1077BA Step 2 entry visit must be completed prior to initiation of HAART.

1077BA Step 3: Mothers from 1077BA Step 1 Arm B or Arm C (who are receiving the triple ARV regimen), or 1077BA Step 2 who are being followed on triple ARV therapy (HAART) will be registered to 1077BA Step 3 if they meet the criteria to switch to a second line regimen as specified in Section 2.613.2. The 1077BA Step 3 Entry visit must be completed prior to the first dose of the second regimen.

Infants in all study arms will receive NVP daily through six weeks (day 42) of life, regardless of whether they are subsequently enrolled in the Postpartum Component or continue to BF (see Section 2.6).

Note: Section 6.0 includes Statistical Considerations for this study component (and all others).

2.4 Selection and Enrollment of Subjects (Antepartum Component)

2.41 <u>1077BA Step 1</u>

2.411 Inclusion Criteria (1077BA Step 1)

2.411.1 Confirmed HIV-1 infection, defined as documented positive results from <u>two</u> samples collected at <u>different</u> timepoints prior to study entry:
Sample # 1 may be tested using any of the following:

- Two rapid antibody tests from two different manufacturers or based on different principles and epitopes
- One EIA OR Western Blot OR immunofluorescence OR chemiluminescence
- One HIV DNA PCR
- One quantitative HIV RNA PCR (> 5,000 copies/mL)
- One qualitative HIV RNA PCR
- One total HIV nucleic acid test

Sample #1 may be tested by non-study public or PEPFAR programs. However, both the result and the sample collection date must be recorded in the subject's chart. Source documentation (patient's medical record/chart, MOH register, laboratory results, etc.) must be available if requested.

Sample #2 may be tested using any of the following:

- One EIA confirmed by Western Blot OR immunofluorescence OR chemiluminescence
- One HIV DNA PCR
- One quantitative HIV RNA PCR (> 5,000 copies/mL)
- One qualitative HIV RNA PCR
- One total HIV nucleic acid test

Sample #2 must be tested in a laboratory that operates according to GCLP guidelines, participates in appropriate external quality assurance programs and is approved by the IMPAACT Central Laboratory.

2.411.2 Currently pregnant and $\geq$ 14 weeks gestation based on clinical or other obstetrical measurements

2.411.3 CD4 $\geq$ 350 cells/mm^3 or greater than or equal to the country-specific threshold for initiation of treatment, if that threshold is > 350 cells/mm^3, on specimen obtained within 30 days prior to study entry

2.411.4 Results of HBV screening (HBsAg testing) available from specimen obtained within 30 days prior to study entry

2.411.5 The following laboratory values from a specimen obtained within 30 days prior to study entry:

- Hemoglobin $\geq$ 7.5 g/dL
- WBC $\geq$ 1,500 cells/mm^3
- ANC $\geq$ 750 cells/mm^3
- Platelets $\geq$ 50,000 cells/mm^3
- ALT $\leq$ 2.5 x upper limit of normal (ULN)
- Estimated creatinine clearance of $\geq$ 60mL/min using the Cockroft-Gault equation for women: {([140 – age (years)] x [weight (kg)]) ÷ [72 x serum Cr (mg/dL)]} x 0.85

2.411.6 Plans to deliver in the study affiliated clinic or hospital

2.411.7 Has no plans to move outside of the study site area during the 24 months following delivery

2.411.8 Age of legal majority for the respective country and willing and able to provide written informed consent

2.411.9 Intends to breastfeed

2.412 Exclusion Criteria (1077BA Step 1)

2.412.1 Participation in PROMISE for a prior pregnancy

2.412.2 Ingestion of any antiretroviral regimen with three or more drugs (regardless of duration) or more than 30 days of a single or dual antiretroviral regimen during current pregnancy according to self report or available medical records

2.412.3 Requires triple ARV therapy (HAART) for own health based on local standard guidelines

2.412.4 WHO Stage 4 disease

2.412.5 Prior receipt of HAART for maternal treatment indications (e.g., CD4 < 350 cells/mm^3 or clinical indications); however, could have received ARVs for the sole purpose of PMTCT in previous pregnancies. (Prior PMTCT regimens could have included a triple ARV regimen, ZDV, 3TC-ZDV and/or sdNVP for PMTCT, as well as use of a short dual NRTI "tail" to reduce risk of NVP resistance.)

2.412.6 In labor – at onset or beyond (These women may be eligible for the Late Presenter registration)

2.412.7 Clinically significant illness or condition requiring systemic treatment and/or hospitalization within 30 days prior to study entry

2.412.8 Current or history of TB disease (positive PPD without TB disease is not exclusionary)

2.412.9 Use of prohibited medications within 14 days prior to study entry (refer to Section 2.63 for list of prohibited medications)

2.412.10 Fetus detected with serious congenital malformation (ultrasound not required to rule out this condition)

2.412.11 Current documented conduction heart defect (specialized assessments to rule out this condition are not required; a heart murmur alone and/or type 1 second-degree atrioventricular block (also known as Mobitz I or Wenckebach) is not considered exclusionary)

2.412.12 Known to meet the local standard criteria for treatment of HBV (Note: HBV DNA testing or other specialized assessments are not expected to be performed as part of 1077BF. A woman would be excluded only if this information is documented from other sources and she meets the local standard criteria for HBV treatment based on those assessments.)

2.412.13 Social or other circumstances which would hinder long-term follow-up, in the opinion of the site investigator

2.412.14 Currently incarcerated

2.42 <u>1077BA Step 2</u>

2.421 Inclusion Criteria (1077BA Step 2)

2.421.1
- On 1077BA Step 1 Arm A (ZDV + sdNVP + TRV tail); OR
- On 1077BA Step 1 Arm B or C (maternal triple ARV prophylaxis) and currently receiving triple ARV prophylaxis but does not meet the criteria for switching to a second line regimen and Step 3 entry; OR

- On Step 1 Arm B or C (maternal triple ARV prophylaxis) and not enrolled in the Postpartum Component or Maternal Health Component but remain in observational follow-up and are not currently receiving a triple ARV regimen (stopped the regimen)

2.421.2 Reached an indication for triple ARV therapy (HAART) for own health as specified in Section 2.613.1

2.421.3 Willing and able to initiate HAART

2.422 Exclusion Criteria (1077BA Step 2)

None.

NOTE: A participant should not move to a new step if she has a toxicity that would necessitate an interruption in therapy based on the Toxicity Management Guidelines (Appendix II); however, the participant may move to the new step once the toxicity has resolved to a grade that would allow therapy to begin.

2.43 1077BA Step 3 (Women from either 1077BA Step 1 Arms B or C, currently receiving triple ARV prophylaxis or 1077BA Step 2 who require a change in their triple ARV regimen)

2.431 Inclusion Criteria (1077BA Step 3)

2.431.1 On 1077BA Step 1 Arm B or C or on Step 2

2.431.2 Met the criteria for switching to a second line regimen, as specified in Section 2.613.2, while on a triple ARV regimen

2.431.3 Willing and able to initiate an alternate triple ARV regimen

2.432 Exclusion Criteria (1077BA Step 3)

2.432.1 Women on 1077BA Step 1 Arm B or C who were not enrolled in the Postpartum Component or Maternal Health Component but remain in observational follow-up and are not currently receiving a triple ARV regimen

NOTE: A participant should not move to a new step if she has a toxicity that would necessitate an interruption in therapy based on the Toxicity Management Guidelines (Appendix II); however, the participant may move to the new step once the toxicity has resolved to a grade that would allow resumption of therapy.

2.44 Enrollment Procedures

All sites must have a site implementation plan (SIP) that has been approved by the 1077BF protocol team. The SIP must include the site's plan for post-study HIV care and treatment for participating women and infants. Completion of DAIDS RSC protocol registration is one of the requirements for site-specific study activation. Each site's SIP must be approved prior to submission of protocol registration documents (described below).

Prior to implementation of this protocol, and any subsequent full version amendments, each site must have the protocol and the protocol informed consent form(s) approved, as appropriate, by their local institutional review board (IRB)/ethics committee (EC) and any other applicable regulatory entity (RE). Upon receiving final approval, sites will submit all required protocol registration documents to the DAIDS Protocol Registration Office (DAIDS PRO) at the Regulatory Support Center (RSC). The

DAIDS PRO will review the submitted protocol registration packet to ensure that all of the required documents have been received.

Site-specific informed consent forms (ICFs) will be reviewed and approved by the DAIDS PRO and sites will receive an Initial Registration Notification from the DAIDS PRO that indicates successful completion of the protocol registration process. A copy of the Initial Registration Notification should be retained in the site's regulatory files.

Upon receiving final IRB/EC and any other applicable RE approval(s) for an amendment, sites should implement the amendment immediately. Sites are required to submit an amendment registration packet to the DAIDS PRO at the RSC. The DAIDS PRO will review the submitted protocol registration packet to ensure that all the required documents have been received. Site-specific ICF(s) WILL NOT be reviewed and approved by the DAIDS PRO and sites will receive an Amendment Registration Notification when the DAIDS PRO receives a complete registration packet. A copy of the Amendment Registration Notification should be retained in the site's regulatory files.

For additional information on the protocol registration process and specific documents required for initial and amendment registrations, refer to the current version of the DAIDS Protocol Registration Manual.

Written informed consent must be obtained before any study-specific screening or enrollment procedures are performed. The woman will be asked to read and sign the consent forms. If the participant is unable to read, the process for consenting illiterate participants, as defined or approved by the local IRB/EC, should be followed. While all of the Components of PROMISE will be described in the Antepartum Component and Late Presenter consent forms, separate consent will be obtained before enrollment into each new component of the study.

After screening is completed and if eligibility criteria are met, the woman (and her unborn infant) will be enrolled and randomized into the Antepartum Component of PROMISE, according to her HBsAg status as described previously. For all subjects from whom a signed screening informed consent form has been obtained, a Screening Checklist must be entered through the DMC Subject Enrollment System. For subjects from whom informed consent has been obtained, but who are deemed ineligible or who do not enroll into the initial protocol component for any reason, a Screening Failure Results form must be completed and keyed into the database.

Because a large proportion of women are likely to be ineligible for study participation based on the required CD4 cell count, women will first be asked to provide consent for study screening. Those found potentially eligible based on initial screening will have the study carefully explained to and discussed with them in detail. They will then be asked to provide informed consent for study enrollment/participation.

Subject enrollment is done through the Data Management Center (DMC) Subject Enrollment System. The appropriate enrollment screen for this component is identified as 1077BA.

Screening laboratory tests can be performed as early as 10 weeks gestation; however, where noted above in the inclusion and exclusion criteria, the specimens/assessments on which eligibility determination is based must be obtained within 30 days prior to study entry (earliest study entry is 14 weeks gestation). Re-assessment may be required, for example, if too much time (> 30 days) passes after the initial assessments/specimens were obtained.

Note: Mothers and their infants are randomized at the same time, to the same study arm. In the case of a multiple birth, the additional infants will be manually assigned to the same study arm. All infants will be provided the same study drug regimen, regardless of maternal study arm assignment.

2.45 Co-Enrollment

Pregnant women enrolled in IMPAACT 1077BF will be encouraged to co-enroll in IMPAACT P1084s and P1026s, where available, to obtain pharmacokinetic data on the PROMISE drugs used during pregnancy and postpartum; no prior approval is required. Co-enrollment in PROMISE and other clinical trials may be considered on a case-by-case basis and requires the approval of the protocol chairs of both studies.

2.5 Study Treatment (Antepartum Component)

2.51 Drug Regimens, Formulation, Administration and Duration

2.511 Women (and their unborn infants) will be randomized in Step 1 to one of three arms:

2.511.1 Step 1 Arm A: ZDV + sdNVP + TRV tail

- Zidovudine 300 mg orally twice daily beginning at ≥ 14 weeks gestation (at study entry/randomization) through delivery
- Nevirapine 200 mg orally (one single dose) at onset of labor
- Emtricitabine-Tenofovir disoproxil fumarate fixed dose combination tablet 200 mg/300 mg x 2 tablets for a total dose of 400 mg/600 mg orally once ideally at onset of labor or as soon as possible thereafter
- Emtricitabine-Tenofovir disoproxil fumarate fixed dose combination tablet 200 mg/300 mg (1 tablet) orally each day after delivery for 7 days or the date of the week 1 visit (up to 14 days), whichever is later

Note: Women who do not receive the single dose of nevirapine as planned (for example, due to precipitous delivery) will not receive the Emtricitabine-Tenofovir disoproxil fumarate.

Note: Women who have false labor and started NVP and TRV should continue daily TRV until 7 days after their last NVP dose; the duration of the TRV tail will be dependent on whether it is false labor or progresses to delivery. Each subsequent episode of labor should be managed as per the Step 1 Arm A dosing regimen, specified above giving the accompanying TRV dose as 2 tablets because of decreased TRV absorption during labor. If women cannot be managed per these instructions (for example, TRV dose delayed or not initiated after NVP) consult the CMC for further management.

Note: Women with prolonged labor will receive a repeat dose of NVP along with 2 Truvada tablets if they have not yet delivered 48 hours after the initial NVP dose.

OR

2.511.2 Step 1 Arm B: 3TC-ZDV (Combivir)/LPV-RTV (triple ARV prophylaxis)

- Lamivudine-Zidovudine fixed dose combination tablet 150 mg /300 mg orally twice daily beginning at ≥ 14 weeks gestation (at study entry/randomization) through delivery and until 1 week postpartum visit (up to 14 days)*
- Lopinavir-Ritonavir fixed dose combination tablet 200 mg/50 mg x 2 tablets for a dose of 400 mg/100 mg orally twice daily beginning at ≥ 14 weeks gestation (at study entry/randomization) through 28 weeks gestation (through the second trimester): total daily dose of 800 mg Lopinavir and 200 mg Ritonavir
- Lopinavir-Ritonavir fixed dose combination tablet 200 mg/50 mg x 3 tablets for a dose of 600 mg/150 mg orally twice daily beginning ≥ 28 weeks gestation, or at the next visit (during the third trimester) through delivery: total daily dose of 1200 mg Lopinavir and 300 mg Ritonavir
- Lopinavir-Ritonavir fixed dose combination tablet 200 mg/50 mg x 2 tablets for a dose of 400 mg/100 mg orally twice daily after delivery until week 1 postpartum visit (up to 14 days)*: total daily dose of 800 mg Lopinavir and 200 mg Ritonavir

* Women randomized to Step 1 Arm B or C (triple ARV prophylaxis) who are found ineligible for or decline enrollment in the Postpartum Component, the triple ARV regimen should be continued after the week 1 visit for up to 28 days postpartum while eligibility for the Maternal Health Component is determined (until either enrolled or determined to be ineligible or unwilling).

OR

2.511.3 Step 1 Arm C: FTC-TDF (Truvada)/LPV-RTV (triple ARV prophylaxis)

- Emtricitabine-Tenofovir disoproxil fumarate fixed dose combination tablet 200 mg/300 mg orally once daily beginning at ≥ 14 weeks gestation (at study entry/randomization) until week 1 postpartum visit (up to 14 days)*
- Lopinavir-Ritonavir fixed dose combination tablet 200 mg/50 mg x 2 tablets for a dose of 400 mg/100 mg orally twice daily beginning at ≥ 14 weeks gestation (at study entry/randomization) through 28 weeks gestation (through the second trimester): total daily dose of 800 mg Lopinavir and 200 mg Ritonavir
- Lopinavir-Ritonavir fixed dose combination tablet 200 mg/50 mg x 3 tablets for a dose of 600 mg/150 mg orally twice daily beginning ≥ 28 weeks gestation, or at the next visit (during the third trimester) through delivery: total daily dose of 1200 mg Lopinavir and 300 mg Ritonavir
- Lopinavir-Ritonavir fixed dose combination tablet 200 mg/50 mg x 2 tablets for a dose of 400 mg/100 mg orally twice daily after delivery until week 1 postpartum visit (up to 14 days)*: total daily dose of 800 mg Lopinavir and 200 mg Ritonavir

* Women randomized to Step 1 Arm B or C (triple ARV prophylaxis) who are found ineligible for or decline enrollment in the Postpartum Component, the triple ARV regimen should be continued after the week 1 visit for up to 28 days postpartum while eligibility for the Maternal Health Component is determined (until either enrolled or determined to be ineligible or unwilling).

At enrollment/randomization, it is expected that the assigned maternal and infant regimens (listed above and below, respectively) will use study-supplied study drugs. Thereafter, if one or more of the assigned study-supplied study drugs cannot be tolerated, the regimen may be modified (in consultation with the

CMC if required per Appendix II) using study-supplied study drugs (see listing in Section 2.516) and/or drugs from other sources.

Regardless of source, all maternal triple ARV regimens must include three or more agents from two or more classes of antiretroviral drugs. All ARVs should be prescribed consistent with current package inserts. Fixed dose FTC-TDF-RPV may be used as an alternative first line regimen for mothers who are not able to tolerate or adhere to LPV-RTV or ATV-RTV. Given that FTC-TDF-RPV has thus far only been studied as a first line regimen, consultation with the CMC is required in advance of prescribing this regimen for any study participant.

Second-line regimens are not defined by this protocol and should be determined at the discretion of study site clinicians.

2.512 Infant ARV Prophylaxis Regimen (all study arms)

Infant Nevirapine Regimen			
**Birth Weight**	**Dose**	**Frequency**	**Time Frame**
≥ 2500 gm	1.5 mL NVP suspension	Once daily	As soon as possible after birth through 42 days of age or through the week 6 visit, whichever is later
2000 to 2499 gm	1.0 mL NVP suspension	Once daily	As soon as possible after birth through 42 days of age or through the week 6 visit, whichever is later
< 2000 gm	2 mg/kg based on birth weight	Once daily	As soon as possible after birth through 3 weeks of age
	4 mg/kg based on weight at 3 weeks of age	Once daily	3 weeks of age through 42 days of age or through the week 6 visit, whichever is later

See Section 2.6 for additional information on initiation and continuation of dosing.

In addition to study drug (NVP), cotrimoxazole (CTX) should be provided to all infants in this component as standard of care beginning at six weeks of age. Supplies of CTX should be obtained locally from non-study sources and, therefore, CTX is NOT considered a study drug for this component.

Similarly, all infants of HIV/HBV co-infected mothers should receive the HBV vaccine series starting at birth, regardless of study arm. Supplies of HBV vaccine should be obtained locally from non-study sources; study funds may be used to purchase vaccine supplies if necessary, but HBV vaccine is NOT considered a study drug for this component; see Section 2.6 regarding the provision of this vaccine.

2.513 Drug Administration

Atazanavir (ATV) and Emtricitabine-Tenofovir disoproxil fumarate-Rilpivirine (FTC-TDF-RPV, Complera) must be given with food; all other study drugs may be given with or without food.

2.514 Study Drug Supply

The study-supplied study drug available for infants in this component is Nevirapine (NVP) suspension (obtained from Boehringer-Ingelheim). The study-supplied study drugs available for mothers in this component are NVP tablets (obtained from Boehringer-Ingelheim); Zidovudine (ZDV), Lamivudine (3TC) and fixed dose combination Combivir (3TC-ZDV) (provided by

GlaxoSmithKline), Tenofovir disoproxil fumarate (TDF) , fixed dosed combination Emtricitabine-Tenofovir disoproxil fumarate (FTC-TDF, Truvada, TRV), and fixed dose combination Emtricitabine-Tenofovir disoproxil fumarate-Rilpivirine (FTC-TDF-RPV, Complera) (provided by Gilead Sciences); Lopinavir-Ritonavir (LPV-RTV) and Ritonavir (RTV) (provided by Abbott); Atazanavir (ATV) (obtained from Emcure Pharmaceuticals); and Didanosine (ddI) and Efavirenz (EFV), which will be obtained from a pharmaceutical supplier. However, all study-supplied drugs may not be available at all study sites; availability will be based on the status of drug regulatory approval for each ARV in each country.

2.515 Study Product Distribution and Accountability

The study products provided through this study will be distributed to the study sites by the NIAID Clinical Research Products Management Center (CRPMC), with the exception of Didanosine (ddI) and Efavirenz (EFV), which may be obtained directly by sites from a pharmaceutical supplier or other source with study resources as needed. The Clinical Research Site Pharmacist of Record can obtain the study products that are supplied through the CRPMC for this protocol by following the instructions provided in the latest version of the *Pharmacy Guidelines and Instructions for DAIDS Clinical Trials Networks*. Instructions for obtaining Didanosine (ddI) and Efavirenz (EFV) with study resources can be found in the study MOP.

The Clinical Research Site Pharmacist of Record is required to maintain records of all study products received, dispensed to study participants, and final disposition of all study products. The Clinical Research Site Pharmacist of Record must follow the instructions in the *Pharmacy Guidelines and Instructions for DAIDS Clinical Trials Networks* DAIDS Pharmaceutical Affairs for the destruction of unused study products.

Any dispensed study drug remaining after discontinuation must be collected.

2.516 Formulations of Study-Supplied Drugs

Generic Name<br>Abbreviation<br>Trade Name	Formulation	Appearance	Storage
Nevirapine<br>NVP<br>Viramune®	10 mg/mL suspension	White to off-white preserved suspension	25°C (77°F) - Excursions permitted between 15-30°C (59-86°F). See USP Controlled Room Temperature.
Nevirapine<br>NVP<br>Viramune®	200 mg tablets	White, oval, biconvex tablet	25°C (77°F) - Excursions permitted between 15-30°C (59-86°F). See USP Controlled Room Temperature.
Zidovudine<br>ZDV<br>Retrovir®	300 mg tablets	Biconvex, white, round, film-coated tablets	15-25°C (59-77°F)
Lamivudine<br>3TC<br>Epivir®	300 mg tablets	Gray, modified diamond-shaped, film-coated tablets	25°C (77°F) - Excursions permitted to 15-30°C (59-86°F). See USP Controlled Room Temperature.
Lamivudine-Zidovudine<br>3TC-ZDV<br>Combivir®	150 mg/300 mg tablets	White, modified capsule shaped, film-coated tablet	2-30°C (36-86°F)
Tenofovir Disoproxil Fumarate<br>TDF<br>Viread®	300 mg tablets	Almond-shaped, light blue, film-coated tablets	25°C (77°F) - Excursions permitted between 15-30°C (59-86°F). See USP Controlled Room Temperature.
Emtricitabine-Tenofovir Disoproxil Fumarate<br>FTC-TDF<br>Truvada®	200 mg/300 mg tablets	Blue, capsule shaped, film-coated tablet	25°C (77°F) - Excursions permitted between 15-30°C (59-86°F). See USP Controlled Room Temperature. Keep container tightly closed. Each bottle contains a silica gel desiccant canister that should remain in the original container to protect the product from humidity.
Lopinavir-Ritonavir<br>LPV-RTV<br>Kaletra®<br>Aluvia®	200 mg/50 mg tablets	Ovaloid, film-coated tablets that will be either red or yellow	20-25°C (68-77°F) - Excursions permitted between 15-30°C (59-86°F). See USP Controlled Room Temperature.
Ritonavir<br>RTV<br>Norvir®	100 mg tablets	White film-coated ovaloid tablets	20-25°C (68-77°F) - Excursions permitted to 15-30°C (59-86°F). See USP controlled room temperature.
Atazanavir<br>ATV	150 and 300 mg capsules	White body with a charcoal gray top with ATV 150 or 300 on both the body and the cap	25°C (77°F) - Excursions permitted between 15-30°C (59-86°F). See USP Controlled Room Temperature.
Didanosine<br>ddI	400 mg and 250 mg capsules	may vary	25°C (77°F) - Excursions permitted between 15-30°C (59-86°F). See USP Controlled Room Temperature. Store in tightly closed containers.
Efavirenz<br>EFV	may vary	may vary	25°C (77°F) - Excursions permitted between 15-30°C (59-86°F). See USP Controlled Room Temperature. Store in tightly closed containers.
Tenofovir Disoproxil Fumarate/Emtricitabine/ Rilpivirine<br>TDF/FTC/RPV<br>Complera®	300 mg/200 mg/25 mg tablets	Purplish-pink, capsule-shaped, film-coated, with "GSI" on one side	25°C (77°F) - Excursions permitted to 15-30°C (59-86°F). See USP Controlled Room Temperature. Store in tightly closed containers.

2.6 Subject Management (Antepartum Component)

Following randomization, women will follow the schedule of evaluations in Appendix IA. Women will remain on their assigned study drug regimen through the 1 week postpartum visit (6-14 days); modifications are allowed for toxicity, in consultation with the Clinical Management Committee* when required per Appendix II. Women will be screened for eligibility into the Postpartum Component prior to the 1 week postpartum visit (6-14 days); screening of women (and their infants) for the Postpartum Component is covered in the consent form for participation in the Antepartum Component (though separate informed consent must be obtained for enrollment into the next component).

Infants in all study arms will receive Nevirapine orally once a day beginning at birth (as soon as possible thereafter, and ideally within 3 days) through 6 weeks (42 days) of age, regardless of whether they are enrolled in the Postpartum Component or continue to BF, unless stopped for HIV-infection, toxicity or other medical reasons. See Section 2.512 for infant Nevirapine regimen from birth through 42 days of age. (Infants with birth weight < 2000 gm are ineligible for the Postpartum Component as specified in Section 4.512.) A negative HIV NAT result must be available at the Week 1 visit (day 6-14 postpartum) for continuation of NVP dosing or for initiation of dosing (if initiation was delayed), regardless of whether the infant is enrolled in the Postpartum Component. If initiation of NVP dosing is delayed beyond the Week 1 visit, a negative HIV NAT result is required before dosing can be initiated. For any gap in NVP dosing of 21 days or more following initiation during the first six weeks of life, a negative HIV NAT result on a specimen obtained within the previous 21 days must be available before dosing can be resumed (< 21days from the day the gap in dosing is identified). During the first six weeks, NVP dosing should be continued regardless of reported exposure to breast milk and at the dosage specified for the infant's birth weight category (< 2000, 2000 to 2499 gm, or ≥ 2500 gm). Infants will be screened for eligibility into the Postpartum Component prior to enrollment at the 1 week postpartum visit (6-14 days).

All enrolled infants will follow the schedule of evaluations in Appendix IB through age 104 weeks to determine the effect of the interventions on longer term HIV-free survival in the infants. This will also allow longer term assessment of the effect of *in utero* exposure to multiple ARVs compared to single drug (ZDV) during pregnancy on infant growth, development and survival at age 104 weeks.

All infants of HIV/HBV co-infected mothers are to receive the HBV vaccine series starting at birth or as soon thereafter as possible, regardless of maternal study arm. HBV vaccine will be provided locally as standard of care (outside of the study) for infants of mothers with HBV or, if necessary, purchased locally with study funds (although not to be considered a study-supplied study drug). Infants who are found to have confirmed HIV infection will be referred for treatment according to country guidelines and will continue to follow the modified schedule of evaluations in Appendix IB.

As described in Section 1.4, PROMISE includes a substudy (IMPAACT P1084s) to compare bone and renal outcomes in women and their infants exposed to TDF during pregnancy to a subset of women and infants who were not exposed to TDF during pregnancy. Eligible women (and their infants) will be encouraged to participate in this sub-study.

*The Clinical Management Committee (CMC) will be composed of the study chair and co-chairs or their designees, representatives from NIAID, NICHD, SDAC, the Data Management Center (DMC) and the PROMISE Operations Center.

2.61 Management Related to Mother-Infant Pairs Not Eligible for the Postpartum Component

At the 1 week (6-14 day) postpartum visit, willing, eligible BF mothers and their infants will be enrolled into the Postpartum Component of PROMISE (see Section 4.0).

2.611 Management of Women (and their Infants) who are Ineligible for the Postpartum Component or who Decline Participation

Women who do not meet eligibility criteria for the Postpartum Component due to a CD4 count < 350 cells/mm^3 or who have an indication for ARV treatment will enter 1077BA Step 2 (see Section 2.613.1).

Women who do not meet eligibility criteria for the Postpartum Component for reasons other than requiring treatment or who decline participation in the Postpartum Component but agree to continue follow-up, will be off study drug treatment, but will remain on study and continue to be followed according to the schedule of maternal evaluations in Appendix IA through the end of the study (96 weeks after the last woman enrolled in the Antepartum Component delivers) or, for women on Step 1, until they meet the criteria for entering 1077BA Step 2 (Section 2.42, Section 2.613.1).

All infants of these women will receive NVP through six weeks (42 days) of age as part of the Antepartum Component study drug regimen (as described in Sections 2.512 and 2.6) and will continue to be followed through age 104 weeks according to the schedule of evaluations in Appendix IB.

Women who were randomized to Step 1 Arm B or C (maternal triple ARV prophylaxis) during pregnancy but who are not eligible or decline participation in the Postpartum Component may be eligible for randomization in the Maternal Health Component (Section 5.31); the triple ARV regimen should be continued in these women after the week 1 visit for up to 28 days postpartum while eligibility for the Maternal Health Component is determined (until either enrolled or determined to be ineligible or unwilling). If such women need HAART for their own health (Section 2.613.1) either at the time that eligibility for the Maternal Health Component is being determined or any time later, they will enter Step 2 of the Antepartum Component if they meet the eligibility criteria specified in Section 2.42.

2.612 Management of Women (and their Infants) whose Infants are Ineligible for the Postpartum Component (or who experience fetal demise)

Women randomized to an antepartum triple ARV prophylaxis arm whose infants are not eligible for the Postpartum Component (see Section 4.51) or who experience fetal demise will be assessed for eligibility for the Maternal Health Component and, if eligible, offered randomization into the Maternal Health Component (see Section 5.0); the triple ARV regimen should be continued in these women after the week 1 visit for up to 28 days postpartum while eligibility for the Maternal Health Component is determined (until either enrolled or determined to be ineligible or unwilling). If they are not eligible for the Maternal Health randomization, they will continue to be followed off study drug but on study according to Appendix IA until 96 weeks after the last woman enrolled in the Antepartum Component has delivered or, for women on Step 1 until they meet the criteria for entering 1077BA Step 2 specified in Section 2.613.1.

Women randomized to Step 1 Arm A (ZDV + sdNVP + TRV tail) whose infants are not eligible for the Postpartum Component (see Section 4.51) will continue to be followed on study according

to Appendix IA until 96 weeks after the last woman enrolled in the Antepartum Component has delivered or until they meet criteria for entering 1077BA Step 2.

All infants of these women will receive NVP through six weeks (42 days) of age as part of the Antepartum Component study drug regimen (as described in Sections 2.512 and 2.6). After six weeks of age, infants should be provided NVP prophylaxis (or another ARV regimen if used locally) as standard of care (outside of the study) for the duration of breastfeeding, unless the mother is on a triple ARV regimen. If NVP is not available locally as standard of care, study-supplied NVP (or 3TC) may be provided to these HIV-exposed infants through cessation of breastfeeding (but not beyond 18 months of age). Infants will continue to be followed through age 104 weeks according to the schedule of evaluations in Appendix IB.

2.613 Management of Antiretroviral Therapy

2.613.1 1077BA Step 2: Management of Women Who are Found to Require Treatment for Their Own Health

A woman who otherwise meets the eligibility criteria in Section 2.42 will be considered to have reached an indication for triple ARV therapy (HAART) for her own health and will enter Step 2 if she:

- experiences clinical progression to an AIDS-defining/WHO Stage 4 illness (see Appendix IV); or
- meets country-specific clinical indications for initiation of ARV treatment; or
- has a confirmed CD4 count below 350 cells/mm^3 or below the country-specific threshold for initiation of treatment, if that threshold is > 350 cells/mm^3; or
- otherwise requires ARV treatment as determined in consultation with the CMC.

The woman may receive study-supplied antiretroviral medications, or she may receive triple ARV therapy of her choice from outside of the study, if the treatment regimen meets the protocol definition of HAART (three or more agents from two or more classes of ARVs) and is provided by prescription.

NOTE: A participant should not move to a new step if she has a toxicity that would necessitate an interruption in therapy based on the Toxicity Management Guidelines (Appendix II); however, the participant may move to the new step once the toxicity has resolved to a grade that would allow resumption of therapy.

1077BA STEP 2 FOLLOW-UP
Women who enter 1077BA Step 2 will continue to follow the schedule of evaluations in Appendix IA, and their infants will continue to follow the schedule of evaluations in Appendix IB.

2.613.2 1077BA Step 3: Management of Women Who Have Disease Progression While on a Triple ARV Regimen or Require a Complete Regimen Change Due to Toxicity

A woman receiving the triple ARV regimen either as prophylaxis through 1077BA Step 1 Arm B or C or through 1077BA Step 2 as therapy for her own health will have virologic as well as clinical and CD4 monitoring. A woman with clinical, immunologic or virologic failure or toxicity as defined below will be registered to the 1077BA Step 3 change in regimen.

The criteria for entering 1077BA Step 3 are:

- Clinical failure defined as development of an AIDS-defining/WHO Stage 4 condition; OR
- Immunologic failure defined as a confirmed decrease in CD4 count to less than any of the following:
  - pre-ARV initiation level (i.e., the baseline CD4 count at study entry), or
  - 50% of the participants peak levels, or
  - 350 cells/mm^3 or below the country-specific threshold for initiation of treatment, if that threshold is > 350 cells/mm^3; OR
- Virologic failure defined as confirmed RNA level > 1,000 copies/mL at or after 24 weeks on a triple ARV regimen; (see note below for counting weeks on a triple ARV regimen); OR
- Significant toxicity requiring a change in the backbone of the regimen, or otherwise requiring a change in more than one class of drug IF the CMC is consulted and approves the step change *in advance*; OR
- Meets country-specific standard indications for a complete change in regimen; OR
- Otherwise requires a change to an alternate triple ARV regimen as determined in consultation with the CMC.

NOTE: If a participant experiences one of the above conditions but the condition is judged by the study clinician as due to non-adherence, systemic illness or other explanatory circumstance, such that a change of regimen is not indicated, with approval from the CMC, entry into Step 3 is not required.

NOTE: For purposes of defining virologic failure, the 24 weeks referenced above refers to the number of continuous weeks on a triple ARV regimen. Please consult the CMC with any questions related to counting weeks on a triple ARV regimen and/or other aspects of defining failure.

While 1077BA Step 3 triple ARV regimens are not defined by this protocol, additional drugs available from the study are described above. 1077BA Step 3 regimens should be determined at the discretion of the study clinicians (consultation with the CMC available but not required). A regimen that is not provided by the study may be used if it meets the study definition of a triple ARV regimen (three or more agents from two or more classes of ARVs) and is provided by prescription.

NOTE: A participant should not move to a new step if she has a toxicity that would necessitate an interruption in therapy based on the Toxicity Management Guidelines (Appendix II); however, the participant may move to the new step once the toxicity has resolved to a grade that would allow resumption of therapy.

1077BA STEP 3 FOLLOW-UP

Women will continue to follow the schedule of evaluations in Appendix IA, and infants will continue to follow the schedule of evaluations in Appendix IB.

2.613.3 Women Who Develop Tuberculosis (TB)

Participants who develop TB and are not receiving a triple ARV treatment regimen should enter Step 2 or 3 as applicable and initiate ARV treatment for their own health.

Participants randomized to a triple ARV regimen who develop TB and need Rifampin-containing TB treatment during their index pregnancy may be offered Efavirenz (dose to be determined by site clinician) in place of LPV-RTV. All participants on TB treatment may continue to receive TDF, FTC, 3TC, ZDV, 3TC-ZDV (Combivir) and FTC-TDF (TRV).

EFV may also be offered to participants on LPV-RTV after delivery (for example, participants on HAART for their own health in Step 2) who need Rifampin-containing TB treatment. If such participants are participating in sexual activity that could lead to another pregnancy, they must agree to use two reliable methods of contraception, including a reliable barrier method of contraception together with another reliable form of contraception while receiving EFV and for 12 weeks after stopping EFV. These participants will have pregnancy testing at each study visit while receiving EFV and for 12 weeks after stopping EFV.

These study drug changes will be made available for the duration of the Rifampin-based TB treatment and for up to 30 days after stopping Rifampin. Thereafter, the participant will return to her assigned study drug regimen.

2.613.4 Virologic Monitoring of Women Receiving Triple ARV Treatment

Virologic failure is not an endpoint in this trial; however, monitoring viral load is used among individuals receiving antiretroviral treatment for their own health to maximize the benefits and to determine when treatment should be changed. Therefore, virologic monitoring will be provided for all women on triple ARV therapy for their own health in 1077BA Step 2 (Appendix IA) and those who require a change in their ARV regimen in 1077BA Step 3 (Appendix IA).

The US Department of Health and Human Services (DHHS) treatment guidelines state that the goal of ARV therapy is sustained suppression of HIV RNA to < 50 copies/mL (or below detectable limits of the available HIV RNA assay). However, the plasma HIV RNA threshold for switching therapy is not precisely defined.

Women receiving triple ARV therapy, who have a plasma HIV RNA level > 1,000 copies/mL at or after 24 weeks of therapy should return (if possible within 4 weeks) for confirmatory plasma HIV RNA. Women with confirmed HIV RNA levels > 1,000 copies/mL at or after 24 weeks of initial or second line therapy will be strongly encouraged to modify their regimen (1077BA Step 3). Women in whom virologic failure is believed to be due to non-adherence, systemic illness, vaccination or other circumstances determined by the study clinicians, will *not* be required to switch therapy unless the study clinician advises that therapy should be changed (consultation with the CMC available but not required). In such cases, the subject should continue scheduled study visits as outlined in Appendix IA.

Study-provided medications will be available to participants who meet 1077BA Step 3 criteria, or participants may access therapy not provided by the study. Therapy choice should include three or more agents from two or more classes of ARVs (the protocol definition of HAART). These regimens may include both study-provided ARVs and ARVs from outside the study, if necessary.

A participant who has reached a confirmed HIV RNA > 1,000 copies/mL but does not wish to change her assigned regimen due to clinical and immunologic stability may be continued on her current regimen and continue to be followed on study with clinical and laboratory monitoring (consultation with the CMC available but not required). If the CD4 cell count falls or the HIV RNA rises, participants will be strongly advised to change therapy.

2.613.5 Management of Second-Line ARV Therapy Failure

Participants who have a confirmed HIV RNA > 1,000 copies/mL on the second-line triple ARV regimen in 1077BA Step 3 or subsequent lines of HAART should be managed according to current standard of care and may continue to receive study provided ARV medications at the discretion of the local investigators, the participant and her primary care provider. Second-line failure due to non-adherence or intolerance may be able to be managed with use of the study-provided medications and decisions will need to be made on a case by case basis. If the participant has never had a CD4 cell count < 350 cells/mm^3, the CMC should be consulted and consideration may be given to careful observation off of a triple ARV regimen. Women who discontinue the triple ARV regimen will continue to be followed on study/off study drugs according to the schedule of evaluations in Appendix IA.

2.613.6 Management of HIV/HBV Co-Infected Women Who Received Triple ARV Prophylaxis in the Antepartum Component

HIV/HBV co-infected women who discontinue their triple ARV regimen may be at risk of rebound HBV viremia and subsequent transaminitis. Management of HIV/HBV co-infected women who discontinue triple ARV prophylaxis as part of the Postpartum Component or Maternal Health Component is specified in Sections 4.726 and 5.526, respectively. Likewise, HIV/HBV co-infected women who remain in observational follow-up in the Antepartum Component (those not eligible for the Postpartum Component or the Maternal Health Component) will have transaminases measured in real-time at 6 and 14 weeks and have plasma stored and tested retrospectively for HBV DNA, HBeAg and HBeAb at 6 and 26 weeks following ARV discontinuation. If, after triple ARV regimen cessation, liver function tests (transaminases or total bilirubin) are Grade 3 or above or if women are symptomatic (e.g., jaundice, severe fatigue), she should have careful clinical evaluation, and her management should be discussed with CMC.

2.613.7 Management of Infants with a Positive HIV Test (and their Mothers)

Infants who have a positive HIV test result should have a second test performed as soon as possible on a separate sample, collected on a different day. For infants on study drug at the time of the first positive HIV test result, the study drug should be held. For infants with confirmed HIV infection (or in whom infection cannot be ruled out following the initial positive test) study drug must be permanently

discontinued; however, infected infants should continue to be followed in 1077BA per Appendix IB through 104 weeks of age. Infants should be referred for care and treatment according to local standard procedures. Infected infants should receive CTX as standard of care (non-study supplied drug) through 52 weeks of age and thereafter based on WHO guidelines and local standards of care. Mothers of infants with confirmed HIV infection will be counseled to continue to BF their infant as per WHO recommendations. If these mothers have been receiving a triple ARV regimen, they should be considered for entry into the Maternal Health Component. See Section 2.612 to determine if mother is eligible for Maternal Health component.

2.613.8 Women Who Become Pregnant on Study

Women who become pregnant again during follow-up will be maintained in study follow-up, and outcomes will be analyzed based on their initial study randomizations. Women who are receiving a triple ARV regimen as part of the study when they become pregnant will continue to receive their study drugs with modification of the specific regimen as needed; they will also be required to provide separate consent to continue taking the study drugs while pregnant if study-supplied (Appendix V). Women who continue taking LPV-RTV will have a dose increase in the third trimester. Women who are not on a study triple ARV regimen when they become pregnant will be treated according to local standard of care.

Pregnancy outcomes should be ascertained and recorded on study CRFs. For participants who are pregnant at the end of the study or participants who are pregnant and decide to discontinue study participation while pregnant, additional post-study contacts should be completed to ascertain pregnancy outcomes. Outcomes may be ascertained based on participant report but medical records should be obtained whenever possible to supplement participant reports.

Sites are also encouraged to prospectively register pregnant subjects in the Antiretroviral Pregnancy Registry by calling the following number in the US: 910-679-1598 or by faxing: 910-256-0637 or by calling the following number in the United Kingdom: + 44-1628-789-666.

2.62 Concomitant Medications

All medications/preparations received by participants (both mothers and infants) during the period of study participation must be documented in the participant's source file, as this information may be needed for assessment of toxicities and adverse events.

- For infants, all medications/preparations (prescription and non-prescription) including alternative, complementary medications/preparations (i.e., traditional medicines) will also be recorded on applicable case report forms for entry into the study database.
- For mothers, all cardiac, diabetic, hepatic, renal drugs, oral antibiotics, OI medications and contraceptives (prescription and non-prescription), all other prescription medications and alternative, complementary medications/preparations (i.e., traditional medicines) will also be recorded on applicable case report forms for entry into the study database.
- For both mothers and infants, the names of alternative, complementary medications/preparations are not required – only whether or not such substances have been used since the last visit.

Sites must refer to the most recent study drug package insert to access additional current information on prohibited and precautionary medications. To avoid drug interaction and adverse events, the

manufacturer's package inserts of the ARV and concomitant agent should always be consulted when a concomitant medication is initiated or the dose is changed. ARV drug interactions can also be found at http://www.hiv-druginteractions.org/.

Concomitant use of traditional medicines is strongly discouraged while participants are on study.

Information on drugs without trade names, with many marketed forms, or those not available in the US may be found at http://www.nccc.ucsf.edu/.

2.63 Prohibited Medications

A participant who requires any medications considered prohibited while on a study drug must have the study drug held or permanently discontinued. A list of medications that are prohibited with study-supplied drugs will be included on the PSWP of the IMPAACT website.

2.64 Precautionary Medications

A list of medications that should be used with caution while on study-supplied drugs will be included on the PSWP of the IMPAACT website.

2.65 Toxicity Management, CRF Recording and Expedited Adverse Event Reporting

- Toxicity management is described in Appendix II.
- The DAIDS Table for Grading the Severity of Adult and Pediatric Adverse Events, Version 1.0, dated December 2004 (with Clarification dated August 2009), (which is available at the following website: http://rsc.tech-res.com) must be followed with the exception of axillary-measured fever and malnutrition/failure-to-thrive in infants, for which supplementary grading scales are included in Section 7.2.
- Case Report Form (CRF) recording requirements are included in Section 7.1.
- Requirements for expedited reporting of serious adverse events (SAEs) are included in Section 7.2.

2.66 Criteria for Study Drug Treatment Discontinuation

Women may be discontinued from ARV treatment temporarily or permanently primarily based on toxicity events and tolerability issues. Women (and infants) who discontinue study drugs for any reason will remain on study and complete the follow-up period including visits, clinical and laboratory evaluations and infant follow-up. The DAIDS Table for Grading the Severity of Adult and Pediatric Adverse Events, Version 1.0, dated December 2004 (with Clarification dated August 2009) and the Toxicity Management Guidelines (Appendix II) will be used to guide these decisions, in consultation with the CMC when required and/or when desired by the site investigator.

Reasons for study drug discontinuation include:
- Drug-related toxicity (see Toxicity Management Guidelines - Appendix II)
- Second virologic failure with CD4 $\geq$ 350 cells/mm^3
- Requirement for prohibited concomitant medications (see Section 2.63)
- Clinical reasons believed life threatening by the site investigator, even if not addressed in the toxicity management guidelines of the protocol
- Request of the primary care provider if s/he thinks the study treatment is no longer in the best interest of the participant
- Request of the participant

- If recommended by an Ethics Committee/Institutional Review Board or Data and Safety Monitoring Board
- Significant non-adherence thought by the site investigator to increase the risk of treatment failure
- Infants only: confirmed HIV infection or inability to rule out infection following one positive test

Any dispensed study drug remaining after discontinuation must be collected.

Note: Early discontinuation of study drug for any reason is not a reason for withdrawal from the study.

2.67 Criteria for Discontinuation from Study Participation

Participants will be discontinued from the study for the following reasons:

- Request by the participant to withdraw
- Request of the primary care provider if s/he thinks the study is no longer in the best interest of the participant, after consultation with the CMC
- Participant judged by the investigator to be a significant risk of failing to comply with the provisions of the protocol as to cause harm to self or seriously interfere with the validity of the study results, after consultation with the CMC
- At the discretion of the leadership of the IMPAACT Group, NIAID, NICHD, the Office for Human Research Protections (OHRP), the US FDA, the pharmaceutical supplier(s), an in-country national health or regulatory agency, or the IRB/EC
- Incarceration or involuntary confinement in a medical facility (e.g., for psychiatric illness or infectious disease) for a duration that interferes with timely completion of scheduled study visits

Evaluations in the Case of Early Withdrawal from the Study
If willing, women who decide to withdraw from participation early and their infants will have the clinical and laboratory evaluations specified on the Early Discontinuation study visit in Appendix IA for mothers and Appendix IB for infants.

2.7 References – Antepartum Component

(1) World Health Organization. Antiretroviral drugs for treating pregnant women and preventing HIV infection in infants – recommendations for a public health approach. July 2010. Available at: http://www.who.int/hiv/pub/mtct/antiretroviral2010/en/index.html.

(2) Panel on Antiretroviral Guidelines for Adults and Adolescents. Guidelines for the use of antiretroviral agents in HIV-1-infected adults and adolescents. US Department of Health and Human Services. March 2012. URL: http://www.aidsinfo.nih.gov/guidelines/html/1/adult-and-adolescent-treatment-guidelines/0/.

(3) Cooper ER, Charurat M, Mofenson LM, et al. Combination ARV strategies for treatment of pregnant HIV infected women and prevention of perinatal transmission. J AIDS 2002; 29 (5): 484-494.

(4) Dorenbaum A, Cunningham CK, Gelber RD, et al. Two-dose intrapartum newborn nevirapine and standard antiretroviral therapy to reduce perinatal HIV transmission: a randomized trial. JAMA 2002; 299(2): 189-198.

(5) Townsend CL, Cortina-Borja M, Peckham CS, et al. Low rates of MTCT of HIV following effective pregnancy interventions in the United Kingdom and Ireland, 2000-2006. AIDS 2008; 22:973-81.

(6) Lallemant M, Jourdain G, Le Cœur S, et al. Single-dose perinatal nevirapine plus standard zidovudine to prevent mother-to-child transmission of HIV-1 in Thailand. N Engl J Med 2004; 351(3):217-228.

(7) Plipat T, Naiwatanakul T, Rattanasuporn N, et al. Reduction in MTCT in Thailand, 2001-2003: Results from population-based surveillance in six provinces. AIDS 2007;21:145-51.

(8) Tonwe-Gold B, Ekouevi DK,Viho I, et al. ARV treatment and prevention of peripartum and postnatal HIV in West Africa: evaluation two–tiered approach. PLoS Med 2007; (Aug) 4: e257.

(9) Thomas T, Masaba R, Borkowf CB, et al. Triple-antiretroviral prophylaxis to prevent mother-to-child HIV transmission through breastfeeding--the Kisumu Breastfeeding Study, Kenya: a clinical trial. PLosMed 2011;8 (3):e1001015.
(10) Feiterna-Sperling NC, Weizsaecker K, Buhrer C, et al. Hematologic effects of maternal antiretroviral therapy and transmission prophylaxis in HIV-1 exposed uninfected newborn infants. JAIDS 2007; 45:43-51.
(11) Jamisse L, Balkus J, Hitti J, et al. Antiretroviral-associated toxicity among HIV-1 seropositive pregnant women in Mozambique receiving nevirapine-based regimens. JAIDS 2007: 44:371-6.
(12) Van Schalkwyk JE, Alimenti A, Khoo D, et al. Serious toxicity associated with continuous nevirapine-based HAART in pregnancy. BJOG 2008; 115: 1297-302
(13) Hitti J, Frenkel LM, Stek AM et al. Maternal toxicity with continuous nevirapine in pregnancy: results from PACTG 1022. JAIDS 2004; 36: 722-726.
(14) Thorne C, Newell ML. The safety of ARV drugs in pregnancy. Expert Opin Drug Safety 2005; 4:323-35.
(15) Ekouvi DK, Inwoley A, Tonwe-Gold B, et al. Variation of CD4 count and percentage during pregnancy and after delivery: implications for HAART initiation in resource-limited settings. AIDS Res Human Retroviruses 2007; 23:1409-1473.
(16) Chen JY, Ribaudo HJ, Souda S, et al. Highly active antiretroviral therapy and adverse birth outcomes among HIV-infected women in Botswana. J Infect Dis 2012 in press.
(17) The SMART Study Group. CD4+ count-guided interruption of anti-retroviral treatment. N Engl J Med 2006; 355:2283-96.
(18) Brinkman K, Ter Hofstede JH, Burger DM et al. Adverse effects of reverse transcriptase inhibitors: mitochondrial toxicity as common pathway. AIDS 1998; 12: 1735-44.
(19) Fleischer R, Boxwell D, Sherman KE. Nucleoside analogues and mitochondrial toxicity. Clin Infect Dis 2004; 38:e79-80.
(20) Ibdah JA, Yang Z, Bennettt MJ. Liver disease in pregnancy and fetal fatty acid oxidation defects. Mol Genet Metab 2000; 71:182-9.
(21) Barret B, Tardieu M, Rustin P, et al. Persistent mitochondrial dysfunction in HIV-1 exposed but uninfected infants; clinical screening in a large prospective cohort. AIDS 2003; 17: 1769-85.
(22) Le Chenadec J, Mayaux MJ, Guihenneuc-Joyaux C, et al. Perinatal antiretroviral treatment and hematopoiesis in HIV–uninfected infants. AIDS 2003; 17:2053-61.
(23) Shaffer N, Chuachoowong PA, Mock C, et al. Short-course zidovudine for perinatal HIV-1 transmission in Bangkok, Thailand: a randomized controlled trial. Bangkok Collaborative Perinatal HIV Transmission Study Group. Lancet 1999; 353 (9144): 773-780.
(24) Dabis F, Msellati P, Meda N, et al. 6-month efficacy, tolerance, and acceptability of a short regimen of zidovudine to reduce vertical transmission of HIV in breastfed children in Cote d'Ivoire and Burkina Faso: placebo-controlled multicentre trial. Lancet 1999; 353 (9155):786-792.
(25) Wiktor SZ, Ekpini E, Karon J, et al. Short-course oral zidovudine for prevention of mother-to-child transmission of HIV-1 in Cote d'Ivoire: a randomized trial. Lancet 1999; 353 (9155):781-785.
(26) McIntyre JA, Hopley M, Moodley D, et al. Efficacy of short-course AZT plus 3TC to reduce nevirapine resistnace in the prevention of mother to child HIV transmission: a randomized clinical trial. PlosMed 2009;6 (10):e1000172.
(27) Chi BH, Sinkala M, Cantrell RA, et al. Single-dose tenofovir and emtricitabine for reduction of viral resistance to non-nucleoside reverse transcriptase inhibitor drugs in women given intrapartum nevirapine for perinatal HIV prevention: a randomized trial. Lancet 2007; 370:1698-1705.
(28) Arrive E, Chaix ML, Nerrienet E, et al. Tolerance and viral resistance after single-dose nevirapine with tenofovir and emtricitabine to prevent vertical transmission of HIV-1. AIDS 2009;23:825-33.
(29) Gulick RM, Ribaudo JH, Shikuma CM et al. Triple nucleoside regiments versus efavirenz-containing regimens for the initial treatment of HIV infection. N Eng J Med 2004; 350:1850-61.
(30) Mandelbrot L, Landreau-Mascaro A, Rekacewica C, et al. Lamivudine-zidovudine combination for prevention of maternal-infant transmission of HIV-1. JAMA. 2001; 285(16):2083-93.

(31) Bryson YL, Mirochnick M, Stek A, et al. Pharmacokinetics and safety of nelfinavir when used in combination with zidovudine and lamivudine in HIV-infected pregnant women: Pediatric AIDS Clinical Trials Group (PACTG) Protocol 353. HIV Clin Trials2008; 9 (2):115-25.
(32) The Petra Study Team. Efficacy of three short-course regimens of zidovudine and lamivudine in preventing early and late transmission of HIV-1 from mother to child in Tanzania, South Africa, and Uganda: a randomized, double-blind, placebo-controlled trial. Lancet 2002; 359:1178-86.
(33) Moodley D, Moodley J, Coovadia H, et al. The South African intrapartum nevirapine trial (SAINT): a multicenter, randomized, controlled trial of nevirapine compared to a combination of zidovudine and lamivudine to reduce intrapartum and early postpartum mother to child transmission of human immunodeficiency virus type-1. J Infect Dis 2003;187:725-35.
(34) Palombi L, Marazzi MC, Voetberg A, et al. Treatment acceleration and the experience of the DREAM program in PMTCT of HIV. AIDS 2007;21 Suppl 4:S65-71.
(35) Peltier CA, Ndayisaba GF, Lepage P, et al. Breastfeeding with maternal antiretroviral therapy or formula feeding to prevent HIV postnatal mother to child transmission in Rwanda. AIDS 2009;23:2415-23.
(36) The Antiretroviral Registry Steering Committee. Antiretroviral Pregnancy Registry International Interim Report for 1 January 1989 through 31 January 2012. Registry Coordinating Center; 2012. Available at URL: www.APRegistry.com.Sellier P, Clevenbergh P, Mazeron MC, et al. Fatal interruption of a 3TC-containing regimen in a HIV-infected patient due to re-activation of chronic hepatitis B virus infection. Scand J Infect Dis 2004;36 (6-7):533-5.
(37) Sellier P, Clevenbergh P, Mazeron MC, et al. Fatal interruption of a 3TC-containing regimen in a HIV-infected patient due to re-activation of chronic hepatitis B virus infection. Scand J Infect Dis. 2004;36(6-7):533-5.
(38) Neau D, Schvoerer E, Robert D, et al. Hepatitis B exacerbation with a precore mutant virus following withdrawal of lamivudine in a human immunodeficiency virus-infected patient. J Infect 2000;41(2):192-4.
(39) Walmsley S, Bernstein B, King M, et al. Lopinavir-ritonavir versus nelfinavir for the initial treatment of HIV infection. N Engl J Med 2002; 346:2039-46.
(40) Stek AM, Mirochnick M, Capparelli E, et al. Reduced lopinavir exposure during pregnancy. AIDS 2006; 20:1931-9.
(41) Gingelmaier A, Kurowski M, Kastner R, et al. Placental transfer and pharmacokinetics of lopinavir and other protease inhibitors in combination with nevirapine at delivery. AIDS 2006; 20:1737-43.
(42) Kesho Bora Study Group. Triple antiretroviral compared with zidovudine and single-dose nevirapine prophylaxis during pregnancy and breastfeeding for prevention of mother-to-child transmission of HIV-1 (Kesho Bora study): a randomised controlled trial. Lancet Infect Dis 2011;11:171-80.
(43) Shapiro RL, Hughes MD, Ogwu A, et al. Antiretroviral regimens in pregnancy and breast-feeding in Botswana. N Engl J Med 2010;362:2282-94.
(44) Mirochnick M, Best BM, Stek A, et al. Lopinavir exposure with an increased dose during pregnancy. JAIDS 2008;49:485-91.
(45) Lyons F, Lechelt M, dE Ruiter A. Steady-state lopoinavir levels in third trimester of pregnancy. AIDS 2007;21:1053-4.
(46) Khoung-Josses M-A, Boussairi A, Palette C, et al. Lopinavir drug monitoring in 36 pregnant women. 14th CROI, Los Angeles, CA, February 2007; Abstract 743.
(47) Best B, Stek A, Mirochnick M, et al for the PACTG/IMPAACT P1026S team. Lopinavir tablet pharmacokinetics with an increased dose uring pregnancy. JAIDS 2010;54:381-8.
(48) Cressey TR, Van Dyke R, Jourdain G, et al, and the IMPAACT P1032 team. Early postpartum pharmacokinetics of lopinavir initiated intrapartum in Thai women. Antimicrob Agents Chemother 2009;53:2189-91.
(49) Saag MS, Cahn P, Raffi F, et al. Efficacy and safety of emtricitabine vs. stavudine in combination therapy in antiretroviral-naive patients. JAMA 2004;292:180-189.
(50) Benson CA, van der Horst C, Lamarca A, et al. A randomized study of emtricitabine and lamivudine in stably suppressed patients with HIV. AIDS 2004;18(17):2269-76.

(51) Szczech GM, Wang LH, Walsh JP, et al. Reproductive toxicology of emtricitabine in mice and rabbits. Reproductive Toxicol 2003; 17:95-108.
(52) Nuesch R, Anaworanich J, Srasuebkul P, et al. Interruptions of tenofovir/emtricitabine-based antiretroviral therapy in patients with HIV/hepatitis B virus. AIDS 2008; 22 (1):152-4.
(53) Gallant JE, Staszewski S, Pozniak AL, et al. For the 903 Study Group. Efficacy and safety of tenofovir DF vs. stavudine in combination therapy in antiretroviral-naive patients: a 3-year randomized trial. JAMA 2004; 292: 191-201.
(54) Arribas JR, Pozniak Al, Gallant JE, et al. Tenofovir disoproxial fumarate, emtricitabine and efavirenz compared with zidovudine/lamivudine and efavirenz in treatment–naive patients: 144-week analysis. JAIDS 2008; 47:74-8.
(55) Schooley RT, Ruane P, Myers RA, et al; Study 902 Team. Tenofovir DF in antiretroviral-experienced patients: a 48-week, randomized, double-blind study. AIDS 2002; 16:1257-63.
(56) Squires K, Pozniak AL, Pierone G Jr, et al; Study 907 Team. Tenofovir disoproxil fumarate in nucleoside-resistant HIV-1 infection: a randomized trial. Ann Intern Med 2004; 139 (5 Pt1):122
(57) Margot NA, Isaacson E, McGowan I, Cheng A, Miller MD. Extended treatment with tenofovir disoproxil fumarate in treatment-experienced HIV-1-infected patients: genotypic, phenotypic, and rebound analyses. JAIDS 2003: 33:15-21.
(58) Kearney BP, Mathias A, Mittan A, et al. Pharmacokinetics and safety of tenofovir disoproxil fumarate on coadministration with lopinavir/ritonavir. JAIDS 2006; 43:278-83.
(59) Jullien V, Treluyer JM, Rey E, et al. Population pharmacokinetics of tenofovir in HIV-infected patients taking active antiretroviral therapy. Antimicrob Agents Chemother 2005; 49:3361-6.
(60) Tarantal AF, Castillo A, Ekert JE, Bischofberger N, Martin RB. Fetal and maternal outcome after administration of tenofovir to gravid rhesus monkeys (Macaca mulatta). JAIDS 2002; 29(3):207-20.
(61) Van Rompay KK, Durand-Gasselin L, Brignolo LL, et al. Chronic administration of tenofovir to rhesus macaques from infancy through adulthood and pregnancy: summary of pharmacokinetics and biological and virological effects. Antimicrob Agents Chemother 2008; 52(9):3144-60.
(62) Tarantal AF, Marthas ML, Shaw J-P, et al. Administration of PMPA to gravid and infant rhesus macaques: safety and efficacy studies. J Acquir Immune Defic Syndr Hum Retrovirol 1999;20(4):323-33.
(63) Flynn PM, Mirochnick M, Shapiro DE, et al. Pharmacokinetics and safety of single-dose tenofovir disoproxil fumarate and emtricitabine in HIV-1-infected pregnant women and their infants. Antimicrob Agents Chemother 2011;55:5914-22.
(64) Burchett S, Best B, Mirochnick M, et al. Tenofovir pharmacokinetics during pregnancy, at delivery, and postpartum. 14th CROI, Los Angeles, CA, February 2007; Abstract 738b.
(65) Hirt D, Urien S, Rey, et al. Population pharmacokinetics of tenofovir in HIV-infected pregnant women and their neonates (anrs 12109). Clin Pharmacol Ther 2009;85:182-9.
(66) Mirochnick M, Kafulafula G, Kreitchmann R, et al. The pharmacokinetics (PK) of tenofovir disoproxil fumarate (TDF) after administration to HIV-1 infected pregnant women and their newborns. 16th CROI, Montreal, Canada, February, 2009.
(67) Habert A, Linde R, Reittner A, et al. Safety and efficacy of Tenofovir in pregnant women. 15th CROI, Boston, MA, February 3-6, 2008; Abstract 627a.
(68) Viganò A, Mora S, Giacomet V, Stucchi S, Manfredini V, Gabiano C, Salvini F, Cellini M, Tamburrini E, Puzzovio M, Zuccotti GV. In utero exposure to tenofovir disoproxil fumarate does not impair growth and bone health in HIV-uninfected children born to HIV-infected mothers. Antivir Ther. 2011;16(8):1259-66.
(69) Nurutdinova D, Onen NF, Hayes E, Mondy K, Overton ET. Adverse effects of tenofovir use in HIV-infected pregnant women and their infants. Ann Pharmacother. 2008;42(11):1581-5. Epub 2008 Oct 28.
(70) Siberry GK, Williams PL, Mendez H, Seage GR 3rd, Jacobson DL, Hazra R, Rich KC, Griner R, Tassiopoulos K, Kacanek D, Mofenson LM, Miller T, Dimeglio LA, Watts DH; for the Pediatric HIVAIDS Cohort Study (PHACS). Safety of tenofovir use during pregnancy: early growth outcomes in HIV-exposed uninfected infants. AIDS. 2012 26:1151-9.

2.8 Sample Informed Consent Form – Antepartum Component Screening

Informed Consent Form – Antenatal Component Screening
IMPAACT 1077BF
Breastfeeding Version of the PROMISE Study
(Promoting Maternal and Infant Survival Everywhere)
Protocol Version 3.0, Dated 15 October 2012

Note to Sites: The version number and date of the protocol should be on the first page and the version number and date of the consent form should be included in a header or footer on each page of the consent form along with page numbering in the following format: Page 1 of x, Page 2 of x, Page 3 of x.

INTRODUCTION

You are being asked to take part in screening tests to determine if you will be eligible to take part, with your baby, in the research study named above because:

- you are infected with human immunodeficiency virus (HIV), the virus that causes AIDS
- you are pregnant
- you are planning to breastfeed

This study is sponsored by the US National Institutes of Health (NIH). The doctor in charge of this study at this site is: *[insert name of site Principal Investigator]*. Before you decide if you want to participate in the screening tests, we would like to explain the purpose, the risks and benefits of participating, and what will be expected of you and your baby if you decide to participate. This informed consent form gives you information about the screening tests. You are free to ask any questions. After the screening has been fully explained to you and if you agree to participate, you will be asked to sign this consent form or make your mark (in front of a witness, if needed). You will be offered a copy of this form to keep.

WHAT SHOULD YOU KNOW ABOUT SCREENING FOR THE PROMISE STUDY?

- Your participation in the screening is entirely voluntary.
- You may decide not to participate in the screening tests or to withdraw from the screening at any time without losing the benefits of your standard medical care.
- Even if you agree to participate in the screening, it does not mean you have agreed to participate in the research study.
- If you decide not to participate in the screening, you cannot participate in this research study, but you can still join another research study later, if one is available and you qualify.

WHY IS THE PROMISE STUDY BEING DONE?

The PROMISE Study has been designed to look for the best way to prevent the transmission of HIV from a mother to her baby during pregnancy and labor and delivery and the best way to prevent HIV transmission during breastfeeding. It is also designed to look for ways to make sure that the HIV-infected mother stays as healthy as possible after breastfeeding. To achieve this, the PROMISE study has three parts.

The purpose of this screening is to see if you will be able to participate in the first part of the PROMISE Study, which is called the "Antepartum Part." The specific purpose of the Antepartum Part of the PROMISE Study is look at the safety and effectiveness of different anti-HIV drug combinations used to prevent the transmission of HIV from a mother to her baby during pregnancy and labor and delivery. We do not know which method will work better to reduce the chance of passing the HIV from mothers to

their babies during these times. We want to determine which of these anti-HIV drug combinations is the best. About 4400 pregnant HIV-infected women and their infants will take part in the Antepartum Part of the PROMISE Study around the world. We expect about *[sites: include local estimate here]* to participate here in this country.

Without any anti-HIV medications, about one in three babies born to HIV-infected pregnant women will become infected and, if infected, may progress to AIDS within a few years. To prevent that from happening, many countries have suggested that women and/or their babies take medications to keep the virus from infecting the baby during pregnancy and/or at the time of labor and following delivery. Different combinations of these medications are used in different places, depending on the National Guidelines.

The PROMISE Study and all of the parts have been approved by the Institutional Review Boards (IRBs)/ Ethics Committees. Institutional Review Boards (IRBs) and Ethics Committees are special groups that watch over the safety and rights of research participants in their community.

WHAT WILL HAPPEN IF YOU AGREE TO THE PROMISE STUDY SCREENING?

If you are interested in joining the PROMISE Study, we will first do some screening tests to see if you are eligible for the Antepartum Part.

The study staff will ask you some questions about your health and pregnancy, review your antenatal and other available health records, and do a physical examination. The study staff will draw about 1 tablespoon (15 mL) of blood from you.

- We may test you for HIV to confirm your status.
- We will test your blood to see how healthy you are.
- We will measure the number of CD4 cells, the cells that fight HIV in your body.
- We will test to see if you are infected with Hepatitis B virus.

You will be asked to return to the clinic to get the results of these blood tests. These blood tests are the first step in determining if you will be able to join the study. If the screening shows that you may be eligible, you will be provided more detailed information about the PROMISE Study and be asked to sign another consent form like this one to participate in the Antepartum Part of the study.

If you join the Antepartum Part of the PROMISE Study, you will be randomly assigned *[sites: insert locally relevant description here such as "flipping a coin"]* to one of three study groups, each receiving a different study drug regimen to help prevent transmission of HIV to the baby. You will be followed throughout your pregnancy and through labor and delivery and for 2-5 years after your baby is delivered. Your baby will be followed until he or she is two years old, even if you do not participate in any other part of the study.

After your baby is born, both of you will be screened to see if you are eligible to move on to one of the other PROMISE Study parts. Before you are asked to join another part of the study, it will be explained to you completely, and you will be encouraged to ask questions. If you are interested and willing to participate in the next part of the study, you will be asked to sign another consent form like this one at that time.

WHY MIGHT THE STUDY DOCTOR STOP MY SCREENING TESTS EARLY?

You will be withdrawn from the screening if at any time the screening tests show that you will not be able to participate in the study. You may also be withdrawn from the screening if the study is cancelled or stopped.

WHAT ARE THE RISKS OF STUDY SCREENING?

Taking blood from you may cause slight pain, swelling, and bruising at the place where the blood is taken. Drawing blood can also cause fainting or infection, but this is rare. If you are screened for this study, some hospital and study staff will know that you have HIV. The study doctors and staff will protect information about you and your participation in these screening tests to the best of their ability. On your screening records, a code will be used instead of your name. Only the study staff will know this code. Study staff will make every possible effort to be sure that others do not learn your HIV status. However, sometimes if you receive special treatments or attend a special clinic, it may make others wonder if you have HIV.

WHAT ARE THE POSSIBLE BENEFITS OF STUDY SCREENING?

These screening tests may or may not be of direct benefit to you. The results of the screening tests will be shared with you and with the medical staff providing your antenatal care, if you wish, as this may help them know more about what care you need. They may refer you for additional care if they find that your body's system for fighting infections is weak. If you do not know whether or not you are infected with hepatitis B, you will find out through the screening tests.

WHAT ARE THE CHOICES IF YOU DO NOT WANT TO BE SCREENED FOR THE STUDY?

You do not have to agree to be screened for this research study. If you do not agree to the screening, your care will not be affected. If you agree to take part in the screening, you can change your mind at any time without losing the benefits of your standard medical care.

You must be screened in order to participate in the first part of the study. If you are not interested in learning more about and possibly participating in other parts of the PROMISE study, you should not join the first part.

At this clinic, there is a special program for all pregnant women who are infected with HIV. *[Sites: please insert appropriate information here for referral to care and treatment of HIV-infected pregnant women at your site.]*

WHAT ABOUT CONFIDENTIALITY?

Every effort will be made to keep personal information confidential. This personal information may be disclosed, if required by law. Any publication of this study will not use your or your baby's name or identify you or your baby personally.

The outreach workers may contact you so we need to know the best way to reach you (such as home visit or phone call). Your records may be reviewed by the ethics committee overseeing the study at this site, the US Food and Drug Administration (FDA), the study sponsor (the US National Institutes of Health) or its agents, the US Office of Human Research Protections, IMPAACT leadership (e.g., staff from the operations center, data management center and network lab), local regulatory authorities, study staff, study monitors, and the drug companies supporting this study.

WILL THERE BE ANY COSTS OR PAYMENTS?

The screening procedures, physical examinations and blood tests will be done free - at no cost to you - but you will not receive any payment for having the screening tests done. *[Sites may add a statement that reimbursement may be provided.]*

WHAT HAPPENS IF I AM INJURED?

It is possible that you could experience a problem or injury that would not have occurred if you did not participate in the screening. If *[the study doctor]* determines that you have been injured as a direct result of being in the screening, you will be given immediate treatment for those injuries at no cost to you and then referred for further care if needed. *[Sites: add local information regarding treatment for injury]*.

However, *[the study doctor]* may determine that your illness or injury would have happened even if you did not participate in the screening. In that case, appropriate care and/or referral will likewise be provided for any illness or injury that occurs during screening *[Sites: Add local information regarding care/referral, and explain whether participants will bear the costs of treatment for non-study-related injury, or if there is some mechanism for covering these costs as well]*.

There are no plans to give you money if you experience a complication, whether or not the problem or injury was related to the screening. You will not be giving up any of your legal rights by signing this consent form.

WHAT IF I DO NOT ENROLL INTO THE STUDY?

If you decide not to take part in the first part of the study (the Antepartum Part) or if you do not meet the eligibility requirements for this part, you will not be able to participate in any other parts of the PROMISE Study. We will still use some of your information from the screening visits, some demographic (e.g., age, gender), clinical (e.g., disease condition, diagnosis), and laboratory information, so that the researchers may determine whether there are patterns or common reasons why people do not join the study. Only a code number will be used for this – not your name or other information that will identify you.

WHAT DO I DO IF I HAVE QUESTIONS OR PROBLEMS?

For questions about screening for this study or a screening-related injury, contact:

- *[insert name of the site investigator or other study staff]*
- *[insert telephone number and physical address of above]*

For questions about your rights as a research participant, contact:

- *[name or title of person on the Institutional Review Board (IRB), Ethics Committee (EC) or other organization appropriate for the site]*
- *[insert telephone number and physical address of above]*

SIGNATURE PAGE
Screening for Antepartum Part of the PROMISE Study (IMPAACT 1077BF)

If you have read this consent form (or had it explained to you), all your questions have been answered and you agree to take part in the screening for this research study, please sign your name below.

______________________________	______________________________
Participant's Name (print)	Participant's Signature and Date
______________________________	______________________________
Name of Study Staff Member Conducting Consent Discussion (print)	Study Staff Signature and Date
______________________________	______________________________
Witness's Name (print) (if needed)	Witness's Signature and Date

2.9 Sample Informed Consent Form – Antepartum Component Enrollment

Informed Consent Form – Antepartum Component Enrollment
IMPAACT 1077BF
Breastfeeding Version of the PROMISE Study
(Promoting Maternal and Infant Survival Everywhere)
Protocol Version 3.0, Dated 15 October 2012

Note to Sites: The version number and date of the protocol should be included on the first page and the version number and date of the consent form should be included in a header or footer on each page along with page numbering in the following format: Page 1 of x, Page 2 of x, Page 3 of x. Sites may omit tables or diagrams if not appropriate; however, the text must be adequate to convey the key messages.

INTRODUCTION

You and your baby are being asked to take part in this research study because:
- you are infected with human immunodeficiency virus (HIV), the virus that causes AIDS
- you are pregnant
- you are planning to breastfeed
- you agreed to participate in the screening for the study previously and the screening tests show that you are eligible to enroll in the study

This study is sponsored by the US National Institutes of Health (NIH). The doctor in charge of this study at this site is: *[insert name of site Principal Investigator]*. Before you decide if you want join this study with your baby, we want you to know about the study. We will explain the study to you. You are free to ask questions at any time. We will ask if you want to join the study with your baby as a pair. If you do want to join with your baby, we will ask you to sign or mark this consent form (in front of a witness, if needed). You will be offered a copy to keep.

WHY IS THE PROMISE STUDY BEING DONE?

The PROMISE Study has been designed to look for the best ways to prevent the transmission of HIV from a mother to her baby during pregnancy, labor and delivery and breastfeeding and ways to make sure that both the HIV-infected mother and HIV-exposed baby stay as healthy as possible from birth and beyond weaning. To achieve this, the PROMISE study has three parts – one for each of the main goals.

PROMISE Goals	Parts
Goal 1:<br>To determine the best combination of anti-HIV medications to give to HIV-infected pregnant women to prevent HIV infection in babies during pregnancy or at time of delivery.	Antepartum
Goal 2:<br>To determine the best way to protect the baby from HIV while being breastfed.	Postpartum
Goal 3:<br>To find the best way to take care of the mother's health during and after pregnancy.	Maternal Health

This is a consent form to join the Antepartum Part of the PROMISE Study. After your baby is born, we will discuss with you the other parts of the PROMISE Study and whether you and your baby qualify to participate. At that time, we will give you detailed information about the next parts of the study, but you are free to ask questions about them now if you would like.

You should not consider joining the Antepartum Part of the PROMISE Study if you are not interested in learning more about and possibly participating in the other parts of the PROMISE Study. If you do not participate in the Antepartum Part of the study, you will not be able to join the next parts of the study.

WHY IS THE ANTEPARTUM PART BEING DONE?

As explained when you agreed to participate in the screening, the specific purpose of the Antepartum Part of the PROMISE Study is to look at the safety and effectiveness of different combinations of anti-HIV medications used to prevent the transmission of HIV from a mother to her baby during pregnancy and during labor and delivery. We want to determine which of the anti-HIV drug combinations is the best to help women prevent transmission of HIV infection to their babies during this time.

Without any anti-HIV medicines, about one in three babies born to HIV-infected pregnant women will become infected and, if infected, may progress to AIDS within a few years. To prevent that from happening, many countries have suggested that women and/or their babies take medications to keep the virus from infecting the baby during pregnancy and/or at the time of labor and following delivery.

In some countries, the National Guidelines suggest a pregnant woman with HIV who is healthy and does not need treatment for her own health, take a regimen of anti-HIV drugs. One regimen includes an anti-HIV medicine called zidovudine (ZDV) during the last 6 months of pregnancy and during labor. ZDV helps decrease the amount of HIV in the blood, and decreases the chances of passing HIV to the baby during delivery. A second anti-HIV medicine called nevirapine (NVP) is also recommended to help decrease the chances of passing HIV to the baby during delivery. NVP is taken just once when labor begins. In some women who take a single dose of NVP (sdNVP), the HIV changes and becomes resistant to the NVP. This means that NVP may not help these women fight HIV if they need to take NVP in the future for their own health. To reduce the chance of this resistance happening, women are sometimes offered another anti-HIV medicine called Truvada (TRV) to take as well. Truvada, which is a combination of tenofovir plus emtricitabine, is continued for at least one week after delivery in order to keep HIV from becoming resistant to NVP.

In some other countries, women are advised to take a combination of three or more different types of anti-HIV drugs ("triple antiretroviral (ARV) prophylaxis") during pregnancy to help prevent transmission of HIV to their babies.

The clinical staff will describe the country-specific standard of care to prevent transmission of HIV from a mother to her baby during pregnancy and delivery and how this care is different than what you may receive in this part of the study.

We do not know which method will work better to reduce the chance of passing the HIV virus from the mom to her baby. For the Antepartum Part of PROMISE, we want look at three options for preventing HIV infection during pregnancy and at the time of delivery If you join the study, you will be assigned by chance, *[sites: insert locally relevant description here such as "like flipping a coin"]*, to one of three study groups. Each group will be given one of the three combinations of anti-HIV drugs that the study is looking at:

**Maternal Triple ARV Prophylaxis Study Group: 3TC-ZDV (Combivir) plus LPV-RTV** Combination anti-HIV medication for as much as the last 6 months of pregnancy, through delivery, and up to 14 days postpartum	OR	**Maternal Triple ARV Prophylaxis Group: FTC-TDF (Truvada) plus LPV-RTV** Combination anti-HIV medication for as much as the last 6 months of pregnancy, through delivery, and up to 14 days postpartum	OR	**ZDV plus single dose NVP plus Truvada Study Group** ZDV for as much as the last 6 months of pregnancy and through delivery, a single dose of NVP during labor, and Truvada at the time of labor for up to 14 days after delivery

You and the study staff will know which group you are in.

No matter what drugs you are given to reduce the risk of HIV transmission to your baby, your baby will be given NVP once a day beginning at birth through six weeks of age to help prevent HIV infection. In the event that you and your baby are not eligible to enter the next part of the study, you or your baby will continue on a regimen to prevent transmission of HIV for the duration of breastfeeding or until 18 months of age (whichever comes first) either through locally available resources or through the study.

The PROMISE Study and all of the parts have been approved by the [*sites: add local ethics committee or IRB information*]. Institutional Review Boards (IRBs) and Ethics Committees are special groups that watch over the safety and rights of research participants in their community.

WHAT WILL HAPPEN IF I NEED ANTIRETROVIRAL TREATMENT FOR MY OWN HEALTH?

If you need antiretroviral treatment for your own health, you will remain in the study. You will be provided counseling about your care and treatment options. You will be offered study drugs or you may take non-study drugs after talking with the study clinicians and your doctor.

WHAT WILL MY BABY AND I HAVE TO DO IF WE TAKE PART IN THIS STUDY?

You will be followed throughout your pregnancy and through labor and delivery. Once your baby is born, you will each be screened to see if you are eligible to continue on one of the other PROMISE Study parts. Whether or not you and your baby are eligible for the other parts, you and your baby will continue to be followed as part of the PROMISE Study.

You will be seen two weeks and four weeks after you join the study; thereafter, you will be seen every four weeks while you are still pregnant. Each visit will last about *[sites—if required by your IRB, insert local information on time required for study visits]*. You will have routine medical check-ups at the study clinic. It is important that you attend all of these Antepartum Part visits. If you do not come for a scheduled visit or if a test result comes back abnormal, the outreach worker will contact you to find out how you are doing. If at any time, you become sick you should let the study nurse or doctor know right away.

You will be seen at labor and delivery, and your baby will be examined after birth. You and your baby will return for a visit between 6 and 14 days after delivery. That visit is expected to last about *[sites—if required by your IRB, insert local information on time required for study visits]*. At that visit, we will discuss whether you and your baby will be able to enroll into other PROMISE parts. If you and/or your baby are not eligible for another PROMISE part, you will each continue to be followed as part of the PROMISE Study. If you are eligible for another part of the study, the specific details will be reviewed with you and, after all of your questions have been answered, you will be asked to sign another informed consent form like this one if you choose to join.

PROMISE Study follow up visits for you and your baby will be at 1, 6, 10 and 14 weeks. Thereafter, they will be about every month for your baby until your baby is 6 months old and about every three months for you. After your baby is 6 months old, follow-up visits will be about every 3 months. These visits are expected to last about *[sites—if required by your IRB, insert local information on time required for study visit]*.

<u>Tests and procedures at the study visits</u>

- <u>Medical history, questionnaire, interviews, and physical exam</u>
  We will ask you about your medical history and about any medications you have taken since the last visit and about how well you are taking the study drugs, if still on them. You and your baby will have a physical exam. We will update your contact information (for example, your address and telephone

number). We may ask questions about your home life and general well being. At some visits, we will also ask questions about infant feeding and nutrition.

- <u>Blood</u>
  Blood will be collected from you for various tests. Some tests measure how well study drugs are controlling the virus and other tests will check on your health. You will have approximately 10 to 32 mL (2-6 tsp *[sites include local relevant wording]*) of blood taken at most visits.

  We will collect about 5 ml (1 tsp *[sites include local relevant wording]*) from your baby at each of the visits. If you are Hepatitis B co-infected we will collect an additional 1 – 3 ml from your baby at some visits. If your baby becomes infected with HIV, we may need to collect some additional blood (about 1 ml *[sites include local relevant wording]*) at some of these visits. At most visits, we will test your baby for HIV and to make sure that the medications are not harming your baby.

  You will be given the results of blood tests that might affect your or your baby's health care as soon as possible, usually at the next study visit. Some of the tests will be used to help us know if you and your baby are eligible for one of the next parts of the PROMISE study. Some of your blood and your baby's blood will be tested immediately, and some of the blood may be kept and used later for study-specified tests.

  Later, we will ask you if you are willing to have some of your blood and your baby's blood saved even after the study is over for future tests not yet specified. This stored blood might be used later on to look for changes in the virus, how your body responds to HIV and/or other HIV diseases. You can still participate in the PROMISE study whether or not you agree to have your and your baby's blood stored after the study is completed. We will review the details with you and you will be asked to sign a separate consent form like this one if you agree to have your own and your baby's blood stored.

- <u>Breast milk</u>
  We will ask you to express up to 20 ml of breast milk (about 4 teaspoons) *[sites to include local relevant wording]*, which will be kept to look for the HIV virus and the presence of study medications.

OTHER INFORMATION

The information collected in this study may be used for other IMPAACT-approved research.

HOW MANY WOMEN AND CHILDREN WILL TAKE PART IN THE PROMISE STUDY?

About 4400 pregnant HIV-infected women and their infants will take part in the Antepartum Part of the PROMISE Study around the world.

HOW LONG WILL MY BABY AND I BE ON THE PROMISE STUDY?

You will be in the study from 2 to 5 years, depending on when you join the study. Most women will be in the study for about 3 years. Your baby will be followed until about 2 years of age.

WHY MIGHT THE DOCTOR TAKE ME OR MY BABY OFF THIS STUDY EARLY?

The study doctor may need to take you or your baby off the study early without your permission if the study is cancelled or stopped.

WHY MIGHT THE DOCTOR HAVE ME OR MY BABY STOP TAKING STUDY MEDICATIONS EARLY?

The study doctor may also need to take you or your baby off the study medications if:
- you and your baby are not able to attend the study visits
- you or your baby are not able to take the study medications
- continuing the study medications may be harmful to you or to your baby
- you or your baby need a treatment that you may not take while on the study
- you request to stop the study medications for you or your baby
- your baby is found to be HIV-infected

If you or your baby have the study medications stopped early for any reason, both you and your baby will remain in the PROMISE study and return for all of the study visits as scheduled.

AFTER THE PROMISE STUDY

After you and your baby have finished your PROMISE Study participation, the PROMISE Study will not be able to continue to provide you or your baby with study medications. If continuing to take these or similar medicines would be of benefit to you or your baby, the PROMISE Study staff will discuss how you may be able to obtain them *[sites insert local information here]*.

WHAT ARE THE RISKS OF THE STUDY?

Taking part in this study may involve some risks and discomforts. These include possible side effects of the anti-HIV medicines that you and your baby may take, possible risks and discomforts from the study tests, and possible risks to your privacy. More information is given on each of these types of risks below.

<u>Side Effects of Anti-HIV Medicines for Women</u>

Women in the Antepartum Part of the PROMISE Study will take at least 3 different anti-HIV medicines. Some of the medicines are combined together in one tablet, others come in separate tablets. Until you join the study, we will not know what specific medicines you will take. Therefore, this form gives information about all the anti-HIV medicines women may take. These are:

- Atazanavir (ATV)
- Didanosine (DDI)
- Efavirenz (EFV)
- Emtricitabine (FTC), taken with tenofovir disoproxil fumarate
- Lamivudine (3TC)
- Lopinavir (LPV), taken with ritonavir
- Nevirapine (NVP), taken as a single dose during delivery
- Rilpivirine (RPV)
- Ritonavir (RTV)
- Tenofovir disoproxil fumarate (TDF)
- Zidovudine (ZDV)

There are no known side effects of taking a single dose of nevirapine. Each of the other medicines can cause side effects, when taken alone and when taken in combination. No new or unexpected side effects are observed with drugs combined in one tablet than those observed when each drug is given separately. The combination drugs that may be used in this part of the study include *[sites: insert locally appropriate names of combination drugs – LPV/RTV; 3TC/ZDV; TDF/FTC; and TDF/FTC/RPV – used at your site]*. Some side

effects are minor, while others can be severe. Some are common, while others are rare. If you join the study, the study staff will tell you about the side effects of the specific medicines you will take. They will check for side effects during study visits and tell you what to do if you have any side effects.

First you should know about the possible severe side effects. These effects are rare, but they can cause serious health problems and can result in death:

- Severe rash. This can be caused by atazanavir, efavirenz and lopinavir/ritonavir, ritonavir

- Abnormal heart beat, which can result in lightheadedness, fainting and serious heart problems. This can be caused by atazanavir, lopinavir/ritonavir and ritonavir.

- Inflammation of the pancreas. The pancreas is an organ near the stomach. When the pancreas becomes inflamed, it can cause pain in the belly, nausea, vomiting and increased fats in the blood. This can be caused by didanosine, efavirenz, lamivudine, lopinavir/ritonavir, ritonavir and tenofovir.

- Inflammation of the liver. The liver is an organ near the stomach. When the liver becomes inflamed, it can cause pain and swelling in the belly, nausea and vomiting. This can be caused by efavirenz, lamivudine, lopinavir/ritonavir, ritonavir, tenofovir and zidovudine.

- Lactic acidosis, enlargement of the liver, and fatty liver, which can result in liver failure. Lactic acidosis is an imbalance in the blood that can cause weight loss, pain in the belly, nausea, vomiting, tiredness, weakness and difficulty breathing. When the liver is enlarged, it can cause pain especially on the right side of the belly, swelling in the belly, nausea, vomiting and loss of appetite. It can also cause bleeding problems that can result in vomiting blood or dark colored stools. Fatty liver is when healthy liver cells are replaced with fat. Sometimes it causes the liver to be enlarged, but doctors usually find out about it from tests of the blood. These effects can be caused by didanosine, emtricitabine, lamivudine, tenofovir and zidovudine. They occur more often in women, pregnant women, people who are overweight, and people who already have liver problems.

- Kidney damage or failure. The kidneys are organs near the middle of your back (one on each side). Doctors usually find out about kidney damage from tests of the blood. These effects can be caused by tenofovir.

- Severe depression, including suicidal thoughts or acts. This can be caused by efavirenz and rilpivirine.

- Other severe mental problems, including aggressive behavior and abnormal thinking. This can be caused by efavirenz.

- Efavirenz might also cause severe harm to unborn babies if taken during the first month of pregnancy.

You should also know about the more common side effects, which are not severe. There are many possible mild and moderate side effects. Some people who take anti-HIV medicines have some of these effects, other people have different effects. The more common mild and moderate side effects are listed below:

Overall Body Effects
- Overall weakness, tiredness, or feeling unwell
- Loss of appetite
- Loss of weight
- Changes in the placement of body fat, such as enlargement of the neck, stomach, and breasts and thinning of the arms, legs, and cheeks
- Numbness or tingling in the hands, arms, feet, legs, or around the mouth
- Pain in the hands or feet
- Allergic reaction
- Fever

Effects on Your Skin
- Rash, with or without itching
- Yellowing of the skin
- Darkening of the palms and soles of feet

Effects on Your Head
- Headache
- Runny nose
- Yellowing of the eyes
- Not seeing normally
- Changes in the sense of taste
- Swelling of the face, lips, or tongue

Effects on Your Chest
- Cough
- Shortness of breath
- Heartburn

Effects on Your Belly
- Pain or discomfort in the belly
- Nausea
- Vomiting
- Gas
- Loose or watery stools
- Inflammation of the gall bladder. The gall bladder is an organ near the stomach. If it becomes inflamed, it can cause severe pain.
- Stones in the gall bladder or kidneys. If these stones form, they can cause severe pain.

Effects on Your Muscles and Bones
- Aches or pains
- Loss of muscle
- Muscle weakness
- Bone thinning or softening (which could increase the chance of breaking a bone)

Effects on Your Blood
- Decreased blood cells
- White blood cells help fight infection.
- Red blood cells help store and transport energy through the body. Low red cells can cause weakness, tiredness and dizziness.
- Increased bleeding if you have hemophilia
- Increased blood sugar or development of diabetes
- Increased fats in the blood that may increase the risk of heart problems
- Other changes in blood test results that may indicate problems with the muscles, kidneys, liver, pancreas or gall bladder. The blood tests that may be affected include tests of how well these organs are working, tests of substances made by these organs, and tests of fats in the blood.

Effects on Your Mind or Mental Function
- Drowsiness
- Trouble sleeping
- Unusual dreams
- Difficulty concentrating
- Confusion
- Depression
- Agitation or anxiety
- Exaggerated feeling of well being
- Hallucinations
- Feeling of strangeness or losing touch with reality
- Dizziness

The list above is not a complete list of all side effects for all anti-HIV medicines. As a reminder, if you join the study, the study staff will tell you about the side effects of the specific medicines you will take.

Other Possible Risks of Anti-HIV Medicines for Women

Risk of Resistance: All anti-HIV medicines can cause resistance. Resistance has been seen in women taking one anti-HIV medicine during pregnancy and in women taking combinations of anti-HIV medicines during pregnancy. When resistance occurs, a medicine no longer works against HIV, which can limit the choices of medicines a person can take against HIV in the future. To avoid resistance, it is important to take anti-HIV medicines as instructed, and not miss doses.

Risk of Immune Reconstitution Syndrome: In some people with advanced HIV infection, symptoms from other infections or certain diseases may occur soon after anti-HIV medicines are started. Some of these symptoms may be life threatening. If you start having new symptoms, or notice that existing symptoms are getting worse after starting your anti-HIV medicines, tell your doctor right away.

Risks with Hepatitis B: Some anti-HIV medicines are active against Hepatitis B. For women who have Hepatitis B, and take anti-HIV medicines that are active against Hepatitis B, there are some risks. Usually, women with Hepatitis B are treated with at least 2 medicines that are active against Hepatitis B. In this study, women might get no, 1, or 2 anti-HIV medicines that are active against Hepatitis B. For women who get 1 anti-HIV medicine that is active against Hepatitis B, the Hepatitis B could become resistant and harder to treat. For women who get 2 anti-HIV medicines that are active against Hepatitis B, stopping the medicines later could cause the Hepatitis B to worsen. If this happens, most women get better quickly without treatment, but in rare cases this has resulted in death.

Side Effects of Anti-HIV Medicines for Babies

The anti-HIV medicines given in the Antepartum Part of the PROMISE Study could affect babies during pregnancy and after birth.

During Pregnancy: Several of the anti-HIV medicines that women in this study will take during pregnancy have been taken safely by thousands of other women during pregnancy, and the only side effect seen in babies has been mild anemia (low red blood cells), which got better on its own, with no treatment. For some medicines, including lopinavir and ritonavir, less information is available. Some studies have suggested higher rates of premature (early) births with the use of this type of medicine, while other studies have not. There also is less information available for tenofovir and emtricitabine, but studies giving these medicines to women at labor and their newborn babies have not found serious problems.

After Birth: Babies will take the anti-HIV medicine nevirapine for 6 weeks after birth. Nevirapine is recommended for all babies born to women who have HIV, and the risks of taking it are the same whether it is given in the study or given outside the study. Some serious side effects have been seen when nevirapine has been taken as treatment by adults and children who have HIV, but no serious side effects have been seen so far in studies of babies taking nevirapine for prevention for several weeks or months after birth. The most common side effects seen in babies have been rash and low red and white blood cells.

Babies may also receive some anti-HIV medicines taken by their mothers through breast milk. It is not known how much medicine is passed into breast milk, and what effects this may have.

The study staff will check for side effects in babies during study visits and tell you what to do if your baby has any side effects. Long term follow up is recommended for babies whose mothers take anti-HIV drugs during pregnancy. A study from France suggested that neurologic problems might occur rarely in babies whose mothers took anti-HIV medicines during pregnancy, but studies in the US did not find this. Other studies have found slight decreases in babies' blood cells. The study staff will talk to you about long term follow up that may be available when your baby's participation in the PROMISE Study ends.

Risks of the Study Tests

Blood drawing may cause some pain, bleeding or bruising where the needle enters. There is a small risk of skin infection at the puncture site.

It may be uncomfortable and awkward to express breast milk.

Possible Risks to Your Privacy

We will make every effort to protect your privacy while you are in this study. Your visits here will take place in private. However, it is possible that others may learn of your participation here and, because of this, may treat you unfairly. There also is a risk to your privacy if someone else taking part in this study knows you.

Other Risks

A recent study suggests that taking a combination of three anti-HIV medicines can make it much less likely for a person with HIV to pass HIV to a sexual partner. If you are assigned to the study group that takes only one anti-HIV drug during pregnancy, you may be more likely to pass HIV to a sexual partner than if you were taking three anti-HIV drugs.

There may be other risks to taking part in the Antepartum Part of the PROMISE Study that are not known at this time.

WHAT IF I BECOME PREGNANT AGAIN WHILE ON THE PROMISE STUDY?

If you wish to become pregnant again or think you may be pregnant again at any time during the study, please tell the study staff right away and we will test you using a blood or urine test. The study staff will talk to you about your choices.

If you get pregnant again during the PROMISE Study, you can continue on the study. You can continue the study ARV regimen if you were on study drugs when you got pregnant or receive other treatment according to your local guidelines. If you get pregnant while on study drugs, you will be asked to sign a separate consent to continue to receive study drugs while you are pregnant. Site staff will discuss with you what is known about using the study drugs in pregnancy and what risks there might be.

If you become pregnant again during the study, and are still pregnant at your last study visit, the study staff will contact you again to find out about the outcome of your pregnancy.

WHAT IF MY BABY IS OR BECOMES INFECTED WITH HIV?

If tests show that your baby is infected with HIV, your baby will be referred for HIV care and treatment *[sites: add local referral information as appropriate]*. HIV care and treatment of babies and children are not provided through the PROMISE Study.

ARE THERE BENEFITS TO ME OR MY BABY TAKING PART IN THIS STUDY?

The strategies used in the Antepartum Part to help prevent a mother from giving HIV to her baby may benefit you and your baby, but no guarantee can be made. Information learned from the PROMISE Study may help other HIV-infected mothers from giving HIV to their babies during pregnancy, at labor and delivery and/or during breastfeeding. A recent study suggests that, if you are assigned to one of the study groups that takes a combination of three anti-HIV drugs, you may be less likely to pass HIV to a sexual partner while taking those drugs. You also may get some satisfaction from knowing that you and your baby participated in this study.

WHAT OTHER CHOICES DO MY BABY AND I HAVE BESIDES PARTICIPATION IN THIS STUDY?

Joining or continuing in this is voluntary. Instead of being in the Antepartum Part of the PROMISE Study, you have the choice to receive the standard regimen of drugs for prevention of mother to infant HIV transmission provided at this location. Your doctor will discuss with you the available standard antepartum/intrapartum regimen for prevention of mother to infant HIV infection. Please talk to your doctor about the risks and benefits of these and other choices available to you and your baby.

You and your baby will continue to receive regular care whether or not you take part in the study.

WHAT ABOUT CONFIDENTIALITY?

Every effort will be made to keep personal information about you and your baby confidential. This personal information may be disclosed, if required by law. Any publication of this study will not use your or your baby's name or identify you or your baby personally.

The outreach workers may contact you so we need to know the best way to reach you (such as home visit or phone call). Your records and those of your baby may be reviewed by the ethics committee that oversees research at this site, the US Food and Drug Administration (FDA), the study sponsor (the US National Institutes of Health) or its agents, the US Office of Human Research Protections, IMPAACT leadership (e.g., staff from the operations center, data management center and network lab), local regulatory authorities, study staff, study monitors, and the drug companies supporting this study.

WHAT ARE THE COSTS TO ME?

There is no cost to you for your study visits, exams, or blood tests or those of your baby. There is no cost to you for the anti-HIV medications used in this study. If you take HIV medicines from another program or provider outside the study, you will need to pay for the medicines, unless the medicines are available free of charge. The study cannot pay for medicines obtained from other programs or providers.

WILL I RECEIVE ANY PAYMENT?

If you have to come to the hospital/clinic *[sites may add or replace with more accurate term]* because of your participation in the study, your transportation and time will be reimbursed to you. You will receive *[site to insert amount]* for each study visit.

WHAT HAPPENS IF EITHER MY BABY OR I ARE INJURED?

It is possible that either you or your baby could experience a problem or injury that would not have occurred if you did not participate in this study. If *[the study doctor]* determines that you or your baby has been injured as a direct result of being in this study, you and/or your baby will be given immediate treatment for those injuries at no cost to you and then referred for further care if needed. *[Sites: add local information regarding treatment for injury]*.

However, *[the study doctor]* may determine that your or your baby's illness or injury would have happened even if you did not participate in this study. In that case, appropriate care and/or referral will likewise be provided for any illness or injury that occurs during the study *[Sites: Add local information regarding care/referral, and explain whether participants will bear the costs of treatment for non-study-related injury, or if there is some mechanism for covering these costs as well]*.

There are no plans to give you money if you or your baby experiences a complication, whether or not the problem or injury was related to study participation. You will not be giving up any of your legal rights by signing this consent form.

WHAT ARE OUR RIGHTS AS RESEARCH PARTICIPANTS?

Taking part in the PROMISE Study is completely voluntary. You may choose not to participate in the study or leave this study at any time. If you decide not to participate or to leave the study early, you and your baby will not be penalized or lose any benefits to which you would otherwise have access outside the study.

We will tell you about new information from this or other studies that may affect you and your baby's health, welfare or willingness to stay in the PROMISE Study. If you want to be informed about the results of the PROMISE Study, the study staff *[will contact you when these are available--sites: include local information about how participants can find out about study results if applicable]*.

WHAT DO I DO IF I HAVE QUESTIONS OR PROBLEMS?

For questions about this study or a research-related injury, contact:

- *[insert name of the site investigator or other study staff]*
- *[insert telephone number and physical address of above]*

For questions about your rights as a research participant or those of your baby, contact:

- *[name or title of person on the Institutional Review Board (IRB), Ethic Committee (EC) or other organization appropriate for the site]*
- *[insert telephone number and physical address of above]*

SIGNATURE PAGE

If you have read this consent form (or had it explained to you), all your questions have been answered and you agree for you and your baby to take part in this study, please sign your name below.

______________________________	______________________________
Participant's Name (print)	Participant's Signature and Date
______________________________	______________________________
Infant's Father's Name (print)<br>(If reasonably available)	Father's Signature and Date
______________________________	______________________________
Name of Study Staff Member Conducting Consent Discussion (print)	Study Staff Signature and Date
______________________________	______________________________
Witness's Name (print)<br>(if needed)	Witness's Signature and Date

3.0 ADMINISTRATIVE MANAGEMENT OF LATE PRESENTERS
(DMC Enrollment Screen/CRF Identifier: 1077BL)

3.1 Rationale (Late Presenters)

Many HIV-infected pregnant women in international settings miss the opportunity for HIV testing, ARVs for prevention of MTCT, and ARV treatment for their own health due to late antenatal presentation and/or limited delivery of counseling and testing services. Despite the availability of effective regimens to reduce transmission of HIV from a pregnant woman to her infant, many infants do not benefit from these interventions due to lack of prenatal HIV testing or presentation very late in pregnancy or in labor. Studies have demonstrated that testing at labor/delivery is feasible (1) and that ARV prophylaxis can reduce the risk of peripartum transmission to the infant even when started in active labor or postpartum (2-3). Depending on the clinical center, up to 30% of HIV-infected women in resource-limited countries may present late in pregnancy or in active labor (4). Thus, it is of major public health importance to include “late presenters” in the PROMISE Study, as intrapartum and early postpartum interventions can successfully reduce MTCT and provide a platform for future comprehensive care for women identified as infected and for their HIV-exposed infants, including enrollment into the Postpartum Component of PROMISE during BF.

3.2 Study Design (Late Presenters)

Late presenters (LP) can be identified in early or active labor or in the immediate postpartum period (up to 5 days postpartum); those who are planning to breastfeed and are potentially interested in enrolling in the Postpartum Component will be considered for registration as a LP. Registration will occur after informed consent (for screening and registration) is obtained and eligibility is determined; this process may begin at the earliest possible time after the identification of HIV infection - ideally before the start of active labor - and must be completed before five days postpartum. Registration as a LP will provide the structure to administer intrapartum and/or immediate postpartum care as well as to complete the necessary screening evaluations for determination of eligibility of women and infants for randomization in the Postpartum Component of PROMISE. Registration of LP will continue until the target number has been randomized into the Postpartum Component of PROMISE (see Sections 6.23 and 6.24 for details).

Intrapartum and immediate postpartum management of LPs will mirror that of the women and infants randomized to the antepartum ZDV + sdNVP + TRV tail arm of the Antepartum Component (Section 2.511.1).

LP women and their infants who meet the eligibility criteria for the Postpartum Component will be enrolled and randomized at day 6-14 using the same procedures as women continuing from the Antepartum Component at the Week 1 (day 6-14) visit. If the woman or her infant does not meet these eligibility criteria, they will complete the study at the Week 6 visit and have no further study visits or procedures.

3.3 Selection and Registration (Late Presenters)

3.31 <u>Inclusion Criteria for Registration</u>

3.311 Age of legal majority for the respective country
3.312 HIV-1 infection, defined as documented positive results from tests performed on one sample at any time prior to Late Presenter Registration

Sample #1 may be tested using any of the following:

- Two rapid antibody tests from two different manufacturers or based on different principles and epitopes
- One EIA OR Western Blot OR immunofluorescence OR chemiluminescence
- One HIV DNA PCR
- One quantitative HIV RNA PCR (> 5,000 copies/mL)
- One qualitative HIV RNA PCR
- One total HIV nucleic acid test

Sample #1 may be tested by non-study public or PEPFAR programs. However, both the result and the sample collection date must be recorded in the subject's chart. Source documentation (patient's medical record/chart, MOH register, laboratory results, etc.) must be available if requested.

Note: Confirmatory testing on a second specimen collected at a different timepoint per the algorithm below should be performed as soon as possible and is required for final eligibility determination for and randomization into the Postpartum Component (1077BP) at the week 1 visit (6-14 days postpartum).

Sample #2 may be performed using any of the following:

- One EIA confirmed by Western Blot OR immunofluorescence OR chemiluminescence
- One HIV DNA PCR
- One quantitative HIV RNA PCR (> 5,000 copies/mL)
- One qualitative HIV RNA PCR
- One total HIV nucleic acid test

Sample #2 must be tested a laboratory that operates according to GCLP guidelines, participates in appropriate external quality assurance programs and is approved by the IMPAACT Central Laboratory.

3.313 In labor (from onset/early labor or beyond) or within 5 days after delivery (with day of delivery considered day 0)
3.314 Has provided written informed consent
3.315 Has no plans to move outside of the study site area during the 24 months following delivery
3.316 If delivered, infant alive and healthy*
3.317 Intends to breastfeed

*In the case of a multiple birth, a mother-infant pair will be included in the Late Presenter registration only if both/all infants and the mother meet the eligibility criteria. If only one infant of a multiple birth is alive, the M-I pair may be registered if the infant and the mother otherwise meet all of the eligibility criteria.

3.32 Exclusion Criteria for Registration

3.321 Participation in PROMISE in prior pregnancy
3.322 Ingestion of any antiretroviral regimen during current pregnancy (including for solely for PMTCT), according to self report and available medical records (Note: Use of ARVs provided as standard of care for PMTCT during labor/delivery or postpartum prior to Late Presenter registration is not exclusionary.)
3.323 If known: CD4 count < 350 cells/mm^3 or below the country-specific threshold for initiation of treatment, if that threshold is > 350 cells/mm^3, on specimen obtained within 30 days prior to study entry (result not required prior to registration)
3.324 Requires triple ARV therapy (HAART) for own health according to local standard guidelines
3.325 WHO Stage 4 disease

3.326 Prior receipt of HAART for maternal treatment indications (e.g., CD4 < 350 cells/mm^3 or clinical indications); however, could have received ARVs for the sole purpose of PMTCT in previous pregnancies. (Prior PMTCT regimens could have included a triple ARV regimen, ZDV, 3TC-ZDV and/or sdNVP for PMTCT, as well as use of a short dual NRTI "tail" to reduce risk of NVP resistance.)

3.327 Current or history of TB disease (positive PPD without TB disease is not exclusionary)

3.328 Known positive infant HIV nucleic acid test (NAT) result (result not required prior to registration)

3.329 Fetal demise or early neonatal death (prior to enrollment/registration)

3.3210 Fetus detected with serious congenital malformation (ultrasound not required to rule out this condition)

3.3211 Life threatening infant illness or birth condition incompatible with life

3.3212 If delivered, infant birth weight < 2.0 kg

3.3213 Social or other circumstances which would hinder long-term follow-up, in the opinion of the site investigator

3.3214 Current documented conduction heart defect (specialized assessments to rule out this condition are not required; a heart murmur alone and/or type 1 second-degree atrioventricular block (also known as Mobitz I or Wenckebach) is not considered exclusionary)

3.33 Registration Procedures

Late presenters (LP) can be identified in early or active labor or in the immediate postpartum period (up to 5 days postpartum); those who are planning to breastfeed and are potentially interested in enrolling in the Postpartum Component will be considered for registration as a LP. Interested HIV-infected pregnant women will be identified and be asked to provide informed consent for LP screening and registration (see Section 3.8, 1077BL – Late Presenter Sample Informed Consent). The initial consent process for "Late Presenter Registration" allows for administration of study drugs intrapartum and immediately postpartum and for screening of both the mother and baby for potential participation in the Postpartum Component. The LP consent process may begin at the earliest possible time before or after delivery - ideally before the start of active labor - and must be completed in time to confirm LP eligibility on or before Day 5 postpartum, when LP registration must be completed. If the consent process is begun during labor, it should be suspended if the woman is under any duress or in the final stages of labor and then resumed/completed after delivery.

If consent is obtained and the eligibility criteria are met, the woman will be registered as a LP through the Data Management Center (DMC) Subject Enrollment System. The appropriate enrollment screen for this LP registration is identified as 1077BL. There will be no randomization of these women at registration; consenting, eligible women and their infants are randomized as part of the Postpartum Component (see Section 4.0). Informed consent for participation in the Postpartum Component may be obtained any time before entry at the Week 1 visit (i.e., between the L/D visit and the Week 1 visit, as long as it is before entry into the PP Component). Women will follow the schedule of evaluations in Appendix IA beginning with the Labor/Delivery visit. Infants will follow the schedule of evaluations in Appendix IB.

3.4 Study Treatment (Late Presenters)

3.41 Drug Regimens, Formulation, Administration and Duration

3.411 Women who are registered in labor will receive:

- Zidovudine 300 mg orally twice daily through delivery
- Nevirapine 200 mg orally (one single dose) at onset of labor (unless woman has already received non-study stock of nevirapine)

- Emtricitabine-Tenofovir disoproxil fumarate fixed dose combination tablet 200 mg/300 mg x 2 tablets for a total dose of 400 mg/600 mg orally once ideally at onset of labor or as soon as possible thereafter
- Emtricitabine-Tenofovir disoproxil fumarate fixed dose combination tablet 200 mg/300 mg (1 tablet) orally each day after delivery for 7 days or the date of the week 1 visit (up to 14 days), whichever is later

Note: Women who do not receive the single dose of nevirapine as planned (for example, due to precipitous delivery) will not receive the Emtricitabine-Tenofovir disoproxil fumarate.

Note: Women who have false labor and received NVP and TRV should continue daily TRV until 7 days after their last NVP dose; the duration of the TRV tail will be dependent on whether it is false labor or progresses to delivery. Each subsequent episode of labor should be managed as per the women who registered in labor dosing regimen, specified above giving the accompanying TRV dose as 2 tablets because of decreased TRV absorption during labor. If women cannot be managed per these instructions (for example, TRV dose delayed or not initiated after NVP) consult the CMC for further management.

Note: Women with prolonged labor will receive a repeat dose of NVP along with 2 Truvada tablets if they have not yet delivered 48 hours after the initial NVP dose.

3.412 Women who are registered after delivery and received nevirapine outside of the study will receive:

- Emtricitabine-Tenofovir disoproxil fumarate fixed dose combination tablet 200 mg/300 mg (1 tablet) orally each day after delivery for 7 days or the date of the week 1 visit (up to 14 days), whichever is later

3.413 Women who are registered after delivery and did not receive nevirapine will not receive the study Emtricitabine-Tenofovir disoproxil fumarate for the one week postpartum.

3.414 Infant ARV Prophylaxis Regimen

**Infant Nevirapine Regimen**			
**Birth Weight**	**Dose**	**Frequency**	**Time Frame**
≥ 2500 gm	1.5 mL NVP suspension	Once daily	As soon as possible after birth through 42 days of age or through the week 6 visit, whichever is later
2000 to 2499 gm	1.0 mL NVP suspension	Once daily	As soon as possible after birth through 42 days of age or through the week 6 visit, whichever is later
< 2000 gm	2 mg/kg based on birth weight	Once daily	As soon as possible after birth through 3 weeks of age
	4 mg/kg based on weight at 3 weeks of age	Once daily	3 weeks of age through 42 days of age or through the week 6 visit, whichever is later

All infants of mothers known to be HIV/HBV co-infected should receive HBV vaccine series starting at birth, regardless of maternal study arm. HBV vaccine will be provided locally as standard of care (outside of the study) for infants of mothers with HBV or, if necessary, purchased locally with study funds (although not to be considered a study-supplied study drug).

See Section 2.6 for additional information on initiation and continuation of dosing.

3.415 Formulation of drugs, administration procedures and drug supply are described in Section 2.514-2.516.

3.5 Subject Management (Late Presenters)

LP registered mother-infant pairs will be screened for eligibility for randomization into the Postpartum Component prior to entry at the Week 1 visit. The infant blood sample for HIV NAT will be obtained within the first 5 days following delivery, along with a maternal CD4 cell count if not already available from a specimen obtained within the prior 30 days. Informed consent for participation in the Postpartum Component may be obtained any time prior to entry at the Week 1 Visit.

At the 1 week (6-14 day) postpartum visit, the maternal CD4 cell count and confirmatory HIV test result must be available for final eligibility determination prior to PP Component entry. Consenting, eligible BF mothers and their infants will be randomized into the Step 1 Postpartum Component at the Week 1 visit and subsequently evaluated and managed as described in Section 4.0. Regardless of whether they enroll in the Postpartum Component or continue to BF, infants will be provided nevirapine through 6 weeks (42 days) of age as described in Sections 2.512 and 2.6.

Women who do not meet eligibility criteria for the Postpartum Component due to CD4 count < 350 cells/mm^3 or below the country-specific threshold for initiation of treatment, if that threshold is > 350 cells/mm^3, or who have a clinical indication for HAART will be referred for locally available treatment. Mother-infant pairs in which the infant has positive on HIV NAT or if a sample was not obtained are also ineligible for the Postpartum Component. Mother-infant pairs who are ineligible for the Postpartum Component will continue to follow the schedules of evaluation in Appendix IA (mother) and Appendix IB (infant) through Week 6 when they will come off study.

Infants who have a positive HIV test result should have a second test performed as soon as possible on a separate sample, collected on a different day. Upon identification of the first positive test result, infant study drug (NVP) should be held. For infants with confirmed HIV infection (or in whom infection cannot be ruled out following the initial positive test) NVP must be permanently discontinued; however, both mother and infant should continue to be followed in 1077BL, per Appendix IA and Appendix IB, respectively, through six weeks postpartum. Infants should be referred for care and treatment according to local standard procedures. Mothers of infants with confirmed HIV infection will be counseled to continue to BF their infant as per WHO recommendations.

3.6 Additional Information (Late Presenters)

Toxicity management is described in Appendix II. Criteria for treatment and study discontinuation applicable to late presenters are described in Sections 2.66 and 2.67. Requirements for CRF recording and serious adverse event reporting are described in Section 7.0. Human subjects considerations are included in Section 8.0.

3.7 Late Presenters References

(1) Bulterys M, Jamieson DJ, O'Sullivan MJ et al. Rapid HIV testing during labor: a multicenter study. JAMA 2004 Jul 14;292(2):219-23.

(2) Wade NA, Birkhead GS, Warren BL, et al. Abbreviated regimens of zidovudine prophylaxis and perinatal transmission of the human immunodeficiency virus. N Engl J Med 1998:339:1409-14.

(3) Taha TE, Kumwenda NI, Gibbons A, et al. Short postexposure prophylaxis in newborn babies to reduce MTCT of HIV: NVAZ randomized clinical trial. Lancet 2003; 362:1171-7.

(4) Jackson JB, Musoke P, Fleming T, et al. Intrapartum and neonatal single-dose nevirapine compared with zidovudine for prevention of mother to child transmission of HIV in Kampala, Uganda: 18 month follow-up of the HIVNET 012 randomised trial. Lancet 2003; 362:859-68.

3.8 Sample Informed Consent Form – Late Presenter Registration

Informed Consent Form – Late Presenter Registration
IMPAACT 1077BF
Breastfeeding Version of the PROMISE Study
(Promoting Maternal and Infant Survival Everywhere)
Protocol Version 3.0, Dated 15 October 2012

Note to Sites: Version number and date of the protocol should be included on the first page and version number and date of the consent form should be included in a header or footer on each page of the consent form along with page numbering in the following format: Page 1 of x, Page 2 of x, Page 3 of x. Sites may omit tables or diagrams if not appropriate. However, the text must be adequate to convey the key messages

INTRODUCTION

You and your baby are being asked to take part in this research study because:
- you are infected with human immunodeficiency virus (HIV), the virus that causes AIDS
- you are about to have a baby or have just delivered
- you are going to breastfeed

This study is sponsored by the US National Institutes of Health (NIH). The doctor in charge of this study at this site is: *[insert name of site Principal Investigator]*. Before you decide if you want to join this study with your baby, we want you to know about the study. We will explain the study to you. You are free to ask questions at any time. We will ask if you want to join the study with your baby as a pair. If you do want to join with your baby, we will ask you to sign this consent form. You will be offered a copy to keep.

WHY IS THE PROMISE STUDY BEING DONE?

The PROMISE Study has been designed to look for the best ways to prevent the transmission of HIV from a mother to her baby during pregnancy, labor and delivery and breastfeeding and ways to make sure that both the HIV-infected mother and HIV-exposed baby stay as healthy as possible from birth and beyond weaning. To achieve these three goals, the PROMISE study has three parts as outlined below.

PROMISE Goals	Parts
Goal 1:<br>To determine the best combination of anti-HIV medications to give to HIV-infected pregnant women to prevent HIV infection in babies during pregnancy or at time of delivery.	Antepartum
Goal 2:<br>To determine the best way to protect the baby from HIV while being breastfed.	Postpartum
Goal 3:<br>To find the best way to take care of the mother's health during and after pregnancy.	Maternal Health

Because you are in labor or have just delivered your infant, you and your baby cannot join the Antepartum Part. This is a consent form to join the PROMISE Study through a Late Presenter Registration process. We will explain why the Late Presenter Registration is being done, what will happen during labor and delivery (if you have not delivered your baby yet) and during the first week of your baby's life. We will tell you about the additional medications that you may receive and the tests that will be done as part of the Late Presenter Registration process.

WHY IS THE LATE PRESENTER REGISTRATION BEING DONE AS PART OF THE PROMISE STUDY?

The Late Presenter Registration is being done so that we can provide study medications during labor and during the first week after delivery that may not be part of the standard care in this area for HIV-infected pregnant women and their infants to help prevent transmission of HIV from mother to baby. This is so that all of the women that enter the next part of the PROMISE Study (the Postpartum Part) will be treated the same during labor and the first few days after delivery. The Late Presenter Registration is also being done so that we can perform some screening tests and evaluations to determine if you and your baby qualify to participate in the Postpartum Part of the study.

In some countries, the National Guidelines recommend that an anti-HIV medicine called zidovudine (ZDV) be given to HIV-infected pregnant women during the last six months of pregnancy and during labor. ZDV helps decrease the amount of HIV in the blood, and decreases the chances of passing HIV to the baby during delivery. A second anti-HIV medicine called nevirapine (NVP) is also recommended to help decrease the chances of passing HIV to the baby during delivery. NVP is taken just once by the mother when labor begins.

But, in some women who take NVP, HIV changes and becomes <u>resistant</u> to the NVP. This means that NVP may not help these women fight HIV if they need to take NVP in the future for their own health. To reduce the chance of this resistance happening in the PROMISE Study, women will take another anti-HIV medicine called Truvada (TRV) as well. Truvada, which is a combination of tenofovir plus emtricitabine, will be continued for one week after delivery in order to keep HIV from becoming resistant to NVP.

In some countries, women are advised to take a combination of three or more different types of anti-HIV drugs (“triple antiretroviral (ARV) prophylaxis”) during pregnancy and during labor to help prevent transmission of HIV to their babies.

The clinical staff will describe the country-specific standard of care to prevent transmission of HIV from a mother to her baby during pregnancy and delivery and how this care is different than what you may receive in this part of the study.

Because you have not received any medicines during pregnancy to help reduce the chance of passing the HIV virus to your infant, we will begin to administer the medications that are recommended as soon as possible. So, for instance, if you are in labor you will receive the HIV drugs recommended to be given to you in labor and after delivery. However, if you have already delivered your infant, you will receive the HIV drugs recommended after your baby is born based on whether or not you received any HIV drugs during labor.

**If you register in labor**:<br>You will be given<br>ZDV through delivery and sdNVP + TRV for up to 14 days following delivery	OR	**If you register after delivery and have already received NVP:**<br>You will be given<br>TRV for up to 14 days following delivery	OR	**If you register after delivery and did not have any HIV drugs during labor**:<br>You will be given no HIV study medications

Your baby will receive a daily dose of NVP beginning soon after birth through six weeks of age to help prevent HIV infection, no matter when you register and even if you do not join the Postpartum Part of the study.

We will also begin to check if you and your baby qualify to participate in the Postpartum Part of the PROMISE Study as described below. You would not be able to participate if we find out that you need HIV medication for your own health or if your baby is already infected with HIV. If this happens, we will refer you to treatment and care for you and for your baby *[sites—add information here regarding referral if needed]*.

You should not consider joining the Late Presenter registration if you are not interested in learning more about and possibly participating in the Postpartum Part of PROMISE. To help you decide if you may be interested, we would like to tell you why the Postpartum Part of PROMISE is being done and how long you and your baby would be followed if you are eligible and decide to join.

The Postpartum Part of PROMISE will try to find out the best way to protect a baby from getting HIV infection during breastfeeding. So, we will compare these two methods in preventing HIV infection during breastfeeding:

**Baby Study Drug Group**		**Mother Study Drug Group**
The anti-HIV medication is given to the baby throughout the time of breastfeeding.	OR	The anti-HIV medications are given to the mother throughout the time of breastfeeding and to the baby through 6 weeks of age.

Depending on when you join the Postpartum Part, you may be in the study from 2 to 5 years. Most women will be in the study for about 3 years. Your baby will be followed until about 2 years of age.

You will have the opportunity to learn more details about the PROMISE Study within the next few days. If you choose to join the next part of study (the Postpartum Part), you will be asked to sign another informed consent form like this one to participate in that part of the study.

The PROMISE Study and all of the parts have been approved by the Institutional Review Boards (IRBs) and Ethics Committees responsible for overseeing research at this site. IRBs/ECs are special groups that watch over the safety and rights of research participants in their community.

WHAT WILL MY BABY AND I HAVE TO DO IF WE REGISTER FOR THE STUDY?

If you agree to join the Late Presenter Registration part of PROMISE, we will ask you some questions and do some tests. Some of the questions and tests will also help us know if you and your baby would be able to join the Postpartum Part of the PROMISE Study.

- <u>Medical history, questionnaire, and physical exam</u>
  We will ask you about any medications you may be taking or have taken in the past. We will ask you about your HIV status and the care you have received. We will ask about this current pregnancy. You and your baby will have a physical exam.

- <u>Blood collected</u>
  Blood will be collected from you for various tests, including HIV tests. You will have approximately 10 to 32 mL (about 2–6 tsp) of blood taken during labor or shortly after birth.

  Your baby will have about 5 mL (about 1 tsp) of blood taken. This blood will be used for various tests including testing to find out if your baby has HIV infection.

You will be given the results of the tests that might affect your or your baby's health care as soon as possible, usually at the next study visit. Some of your blood or your baby's blood will be tested immediately, and some of the blood may be kept and used later for study-specified tests.

OTHER INFORMATION

The information collected in this study may be used for other IMPAACT-approved research.

HOW MANY WOMEN AND CHILDREN WILL TAKE PART IN THE PROMISE STUDY?

Approximately 1,550 HIV-infected women from all over the world who intend to breastfeed and their infants will join the Postpartum Component of the PROMISE Study through the Late Presenter Registration Process during labor or soon after delivery.

HOW LONG WILL MY BABY AND I BE IN THE STUDY?

You and your baby will be in the Late Presenters Registration part until day 6–14 after you deliver if you enroll in the Postpartum Component. If you are not eligible to enroll in the Postpartum Component or if you decide not to enroll, you and your baby will be followed for 6 weeks on the study and referred for regular care.

WHY MIGHT THE DOCTOR TAKE ME OR MY BABY OFF THIS STUDY EARLY?

The study doctor may need to take you or your baby off the study early without your permission if the study is cancelled or stopped.

WHY MIGHT THE DOCTOR HAVE ME OR MY BABY STOP TAKING STUDY MEDICATIONS EARLY?

The study doctor may also need to take you or your baby off the study medications early if:
- you and your baby are not able to attend the study visits
- you or your baby are not able to take the study medications
- continuing the study medications may be harmful to you or to your baby
- you or your baby need a treatment that you may not take while on the study
- you request to stop the study medications for you or your baby
- your baby is found to be HIV-infected

If you or your baby has the study medications stopped early, the study staff will discuss with you how long you may stay on the study. How long you will be in the study depends on the reason that the study medication was stopped.

AFTER THE PROMISE STUDY

After you and your baby have finished your PROMISE Study participation, the study will not be able to continue to provide you or your baby with the study medications. If continuing to take these or similar medicines would be of benefit to you or your baby, the study staff will discuss how you may be able to obtain them *[sites insert local information here]*.

WHAT ARE THE RISKS OF THE STUDY?

Taking part in this study may involve some risks and discomforts. These include possible side effects of the anti-HIV medicines that you and your baby may take, possible risks and discomforts from the study tests, and possible risks to your privacy. More information is given on each of these types of risks below.

Side Effects of Anti-HIV Medicines for Women

Women in the Late Presenters Part of the PROMISE Study may take as many as 4 different anti-HIV medicines. Some of the medicines are combined together in one tablet, others come in separate tablets. This form gives information about the anti-HIV medicines women may take in the Late Presenters Part. These are:

- Emtricitabine (FTC)
- Lamivudine (3TC)
- Nevirapine, taken as a single dose during delivery (NVP)
- Tenofovir disoproxil fumarate (TDF)
- Zidovudine (ZDV)

There are no known side effects of taking a single dose of nevirapine. Each of the other medicines can cause side effects, when taken alone and when taken in combination. No new or unexpected side effects are observed with drugs combined in one tablet than those observed when each drug is given separately. The combination drugs that may be used in this part of the study include *[sites: insert locally appropriate names of combination drugs – 3TC/ZDV; TDF/FTC – used at your site]*. Some side effects are minor, while others can be severe. Some are common, while others are rare. If you join the study, the study staff will tell you about the side effects of the specific medicines you will take. They will check for side effects during study visits and tell you what to do if you have any side effects.

First you should know about the possible severe side effects. These effects are rare, but they can cause serious health problems and can result in death:

- Inflammation of the pancreas. The pancreas is an organ near the stomach. When the pancreas becomes inflamed, it can cause pain in the belly, nausea, vomiting and increased fats in the blood. This can be caused by lamivudine and tenofovir.

- Inflammation of the liver. The liver is an organ near the stomach. When the liver becomes inflamed, it can cause pain and swelling in the belly, nausea and vomiting. This can be caused by tenofovir, lamivudine and zidovudine.

- Lactic acidosis, enlargement of the liver, and fatty liver, which can result in liver failure. Lactic acidosis is an imbalance in the blood that can cause weight loss, pain in the belly, nausea, vomiting, tiredness, weakness and difficulty breathing. When the liver is enlarged, it can cause pain especially on the right side of the belly, swelling in the belly, nausea, vomiting and loss of appetite. It can also cause bleeding problems that can result in vomiting blood or dark colored stools. Fatty liver is when healthy liver cells are replaced with fat. Sometimes it causes the liver to be enlarged, but doctors usually find out about it from tests of the blood. These effects can be caused by emtricitabine, lamivudine, tenofovir and zidovudine. They occur more often in women, pregnant women, people who are overweight, and people who already have liver problems.

- Kidney damage or failure. The kidneys are organs near the middle of your back (one on each side). Doctors usually find out about kidney damage from tests of the blood. These effects can be caused by tenofovir.

You should also know about the more common side effects, which are not severe. There are many possible mild and moderate side effects. Some people who take anti-HIV medicines have some of these effects, other people have different effects.

The more common mild and moderate side effects are listed below:

**Overall Body Effects**<br>• Overall weakness, tiredness, or feeling unwell<br>• Loss of appetite<br>• Loss of weight<br>• Changes in the placement of body fat, such as enlargement of the neck, stomach, and breasts and thinning of the arms, legs, and cheeks<br>• Numbness or tingling in the hands, arms, feet, legs, or around the mouth<br>• Pain in the hands or feet<br>• Allergic reaction<br>• Fever	**Effects on Your Muscles and Bones**<br>• Aches or pains<br>• Muscle weakness<br>• Bone thinning or softening (which could increase the chance of breaking a bone)
**Effects on Your Skin**<br>• Rash, with or without itching<br>• Yellowing of the skin<br>• Darkening of the palms and soles of feet<br>**Effects on Your Head**<br>• Headache<br>• Runny nose<br>• Yellowing of the eyes<br>**Effects on Your Chest**<br>• Cough<br>• Shortness of breath<br>• Heartburn	**Effects on Your Blood**<br>• Decreased blood cells<br>• White blood cells help fight infection.<br>• Red blood cells help store and transport energy through the body. Low red cells can cause weakness, tiredness and dizziness.<br>• Increased fats in the blood that may increase the risk of heart problems<br>• Other changes in blood test results that may indicate problems with the muscles, kidneys, liver, pancreas or gall bladder. The blood tests that may be affected include tests of how well these organs are working, tests of substances made by these organs, and tests of fats in the blood.
**Effects on Your Belly**<br>• Pain or discomfort in the belly<br>• Nausea<br>• Vomiting<br>• Loose or watery stools	**Effects on Your Mind or Mental Function**<br>• Drowsiness<br>• Trouble sleeping<br>• Unusual dreams<br>• Depression<br>• Dizziness

The list above is not a complete list of all side effects for all anti-HIV medicines. As a reminder, if you join the study, the study staff will tell you about the side effects of the specific medicines you will take.

<u>Other Possible Risks of Anti-HIV Medicines for Women</u>

Risk of Resistance: All anti-HIV medicines can cause resistance. When resistance occurs, a medicine no longer works against HIV, which can limit the choices of medicines a person can take against HIV in the future. To avoid resistance, it is important to take anti-HIV medicines as instructed, and not miss doses.

Risk of Immune Reconstitution Syndrome: In some people with advanced HIV infection, symptoms from other infections or certain diseases may occur soon after anti-HIV medicines are started. Some of these symptoms may be life threatening. If you start having new symptoms, or notice that existing symptoms are getting worse after starting your anti-HIV medicines, tell your doctor right away.

Risks with Hepatitis B: Some anti-HIV medicines are active against Hepatitis B. For women who have Hepatitis B, and take anti-HIV medicines that are active against Hepatitis B, there are some risks. The Hepatitis B could become resistant and harder to treat. Also, if women later stop taking the medicines that are active against Hepatitis B, this could cause the Hepatitis B to worsen. If this happens, most women get better quickly without treatment, but in rare cases this has resulted in death.

<u>Side Effects of Anti-HIV Medicines for Babies</u>

The anti-HIV medicine nevirapine will be given to babies in the Late Presenter Part of the PROMISE Study. Babies will take nevirapine for 6 weeks after birth. Nevirapine is recommended for all babies born to women who have HIV, and the risks of taking it are the same whether it is given in the study or given outside the study. Some serious side effects have been seen when nevirapine has been taken as treatment by adults and children who have HIV, but no serious side effects have been seen so far in studies of babies taking nevirapine for prevention for several weeks or months after birth. The most common side effects seen in babies have been rash and low red and white blood cells.

Babies may also receive some anti-HIV medicines taken by their mothers through breast milk. It is not known how much medicine is passed into breast milk, and what effects this may have.

The study staff will check for side effects in babies during study visits and tell you what to do if your baby has any side effects.

<u>Risks of the Study Tests</u>

Blood drawing may cause some pain, bleeding or bruising where the needle enters. There is a small risk of skin infection at the puncture site.

<u>Possible Risks to Your Privacy</u>

We will make every effort to protect your privacy while you are in this study. Your visits here will take place in private. However, it is possible that others may learn of your participation here and, because of this, may treat you unfairly. There also is a risk to your privacy if someone else taking part in this study knows you.

<u>Other Risks</u>

There may be other risks to taking part in the Late Presenters Part of the PROMISE Study that are not known at this time.

WHAT IF MY BABY IS INFECTED WITH HIV?

If tests show that your baby is infected with HIV, your baby will be referred for HIV care and treatment *[sites: add local referral information as appropriate]*. HIV care and treatment of babies and children are not provided through the PROMISE Study.

ARE THERE BENEFITS TO ME OR MY BABY TAKING PART IN THIS STUDY?

There may be some benefit to you or your baby from the methods given to prevent HIV infection in the baby, but no guarantee can be made. Information learned from the PROMISE Study may help other HIV-infected mothers from giving HIV to their babies during pregnancy, at labor and delivery and/or during breastfeeding. You may get some satisfaction from knowing that you and your baby participated in this study.

WHAT OTHER CHOICES DO MY BABY AND I HAVE BESIDES PARTICIPATION IN THIS STUDY?

Joining or continuing in this is voluntary. Instead of being in this study, you have the choice to receive the standard regimen of drugs for prevention of mother to infant HIV transmission provided at this location to women who present for delivery. Your doctor will discuss with you the available standard regimens for prevention of mother to infant HIV infection for women who present late. Please talk to your doctor about the risks and benefits of these and other choices available to you and your baby.

You and your baby will continue to receive regular care whether or not you take part in the study.

WHAT ABOUT CONFIDENTIALITY?

Every effort will be made to keep personal information confidential. This personal information may be disclosed if required by law. Any publication of this study will not use your or your baby's name or identify you or your baby personally.

The outreach workers may contact you so we need to know the best way to reach you (such as home visit or phone call). Your records and those of your baby may be reviewed by the ethics committee overseeing research at this site, the US Food and Drug Administration (FDA), the study sponsor (the US National Institutes of Health) or its agents, the US Office of Human Research Protections, IMPAACT leadership (e.g., staff from the operations center, data management center and network lab), local regulatory authorities, study staff, study monitors, and the drug companies supporting this study.

WHAT ARE THE COSTS TO ME?

There is no cost to you for your study visits, exams, or blood tests or those of your baby. There is no cost to you for the anti-HIV medications used in this study. If you take HIV medicines from another program or provider outside the study, you will need to pay for the medicines, unless the medicines are available free of charge. The study cannot pay for medicines obtained from other programs or providers.

WILL I RECEIVE ANY PAYMENT?

If you have to come to the hospital/clinic *[sites may add or replace with more accurate term]* because of your participation in the study, your transportation and time will be reimbursed to you. You will receive *[site to insert amount]* for each study visit.

WHAT HAPPENS IF EITHER MY BABY OR I ARE INJURED?

It is possible that either you or your baby could experience a problem or injury that would not have occurred if you did not participate in this study. If *[the study doctor]* determines that you or your baby has been injured as a direct result of being in this study, you and/or your baby will be given immediate treatment for those injuries at no cost to you and then referred for further care if needed. *[Sites: add local information regarding treatment for injury]*.

However, *[the study doctor]* may determine that your or your baby's illness or injury would have happened even if you did not participate in this study. In that case, appropriate care and/or referral will

likewise be provided for any illness or injury that occurs during the study *[Sites: Add local information regarding care/referral, and explain whether participants will bear the costs of treatment for non-study-related injury, or if there is some mechanism for covering these costs as well]*.

There are no plans to give you money if you or your baby experiences a complication, whether or not the problem or injury was related to study participation. You will not be giving up any of your legal rights by signing this consent form.

WHAT ARE OUR RIGHTS AS RESEARCH PARTICIPANTS?

Taking part in study is completely voluntary. You may choose not to participate in the study or leave this study at any time. If you decide not to participate or to leave the study early, you and your baby will not be penalized or lose any benefits that you would otherwise have access to outside the study.

We will tell you about new information from this or other studies that may affect you and your baby's health, welfare or willingness to stay in this study. If you want to be informed about the results of the PROMISE Study, the study staff *[will contact you when these are available--sites: include local information about how participants can find out about study results if applicable]*.

WHAT DO I DO IF I HAVE QUESTIONS OR PROBLEMS?

For questions about this study or a research-related injury, contact:
- *[insert name of the site investigator or other study staff]*
- *[insert telephone number of above]*

For questions about you or your baby's rights as a research participant, contact:
- *[name or title of person on the Institutional Review Board (IRB), Ethics Committee (EC) or other organization appropriate for the site]*
- *[telephone number of above]*

SIGNATURE PAGE
If you have read this consent form (or had it explained to you), all your questions have been answered and you agree for you and your baby to take part in this study, please sign your name below.

______________________________	______________________________
Participant's Name (print)	Participant's Signature and Date
______________________________	______________________________
Infant's Father's Name (print)<br>(If reasonably available)	Father's Signature and Date<br>(If reasonably available)
______________________________	______________________________
Name of Study Staff Conducting<br>Consent Discussion (print)	Study Staff Signature and Date
______________________________	______________________________
Witness's Name (print)<br>(if needed)	Witness Signature and Date

4.0 POSTPARTUM COMPONENT: PREVENTION OF BREAST MILK MOTHER TO CHILD TRANSMISSION

SCHEMA: POSTPARTUM COMPONENT
(DMC Enrollment Screen/CRF Identifier: 1077BP)

DESIGN: A strategy trial that will enroll and randomize consenting, eligible postpartum women who have a CD4 count $\geq$ 350 cells/mm^3 and their uninfected infants within 1 week (6-14 days) following delivery to one of two study arms:

Arm A: Maternal triple ARV prophylaxis through BF cessation or through 18 months postpartum, whichever comes first (with infant NVP prophylaxis through six weeks of age)

Arm B: Infant NVP prophylaxis through BF cessation or through 18 months postpartum, whichever comes first (with no maternal prophylaxis)

Women and infants on Step 1 Arms A and B will be followed on the same schedules. For a woman randomized to the infant NVP prophylaxis arm (Step 1 Arm B), a triple ARV therapy (HAART) will be started if she reaches an indication for initiating HAART for her own health as specified in Section 4.721.

POPULATION: Consenting, eligible mother-infant pairs from the Antepartum Component who plan to BF; and consenting, eligible mother-infant pairs from the Late Presenter Registration who plan to BF (women not randomized in the Antepartum Component, who did not receive any ARVs prior to labor and delivery and were identified during labor or immediately postpartum).

SAMPLE SIZE: The accrual target is approximately, 4,650 mother-infant pairs (all eligible BF mother-infant pairs who were randomized in the Antepartum Component plus up to approximately 1,550 BF late presenters)

STRATIFICATION: Receipt of a triple ARV prophylaxis regimen during antepartum period vs. ZDV + sdNVP + TRV tail vs. only intrapartum/postpartum ZDV + sdNVP + TRV tail [late presenters] vs. none [late presenters] and by country

TREATMENT REGIMEN: 1077BP Step 1: At entry, participants will be randomized to one of two arms:

Arm A (maternal prophylaxis): Maternal triple ARV prophylaxis given from 6 (up to 14) days postpartum through BF cessation or through 18 months postpartum, whichever comes first unless stopped for toxicity, other medical reasons or confirmed infant HIV infection (of all the woman's study infants, if multiple births) plus infant NVP prophylaxis through six weeks (42 days) of age (continued from the Antepartum Component study drug regimen). The study-supplied maternal triple ARV regimen is LPV-RTV plus TRV (fixed dose combination FTC-TDF).

Arm B (infant prophylaxis): Infant NVP prophylaxis given once daily given from 6 (up to 14) days of age (continued from the Antepartum Component study drug regimen) through BF cessation or through 18 months postpartum,

whichever comes first unless stopped for infant HIV infection, toxicity, or other medical reasons.

1077BP Step 2: A mother who reaches an indication for initiating triple ARV treatment for her own health (as specified in Section 4.721) while on 1077BP Step 1 Arm B (infant prophylaxis) or on 1077BP Step 1 Arm A while still on triple ARV prophylaxis but before meeting the criteria for switching to a second line regimen or after having stopped triple ARV prophylaxis regimen will be registered to this step. All mothers entering Step 2 must complete a step change entry visit. For a mother not on a triple ARV regimen, the 1077BP Step 2 Entry visit must be completed prior to initiation/re-initiation of HAART.

1077BP Step 3: A mother from 1077BP Step 1 Arm A (who is receiving triple ARV prophylaxis) or 1077BP Step 2 (who is being followed on a triple ARV regimen for treatment) will register to this step if she meets the criteria for switching to a second line regimen (as specified in Section 4.722). The Step 3 Entry visit must be completed prior to the first dose of the second line regimen.

STUDY DURATION: Women will be followed until 96 weeks after the last woman on the Antepartum Component delivers (approximately 2-5 years, depending on the rate of accrual); infants will be followed through age 104 weeks.

OBJECTIVES

Primary Objectives

1. To evaluate the comparative efficacy of giving daily maternal triple ARV prophylaxis versus daily infant NVP prophylaxis during BF to reduce cumulative HIV transmission from BF.
2. To assess the safety and tolerability of these ARV regimens for mother and infant.

Secondary Objectives

1. To assess the distribution of time to postnatal HIV transmission according to allocated study arms.
2. To assess whether the relative efficacy of maternal triple ARV prophylaxis and infant NVP in preventing postpartum MTCT differs in early-presenting (Antepartum Component enrollees) versus late-presenting mothers or in early-presenting mothers who received triple ARV prophylaxis versus ZDV + sdNVP + TRV tail during pregnancy.
3. To compare cumulative 24-month HIV-free survival among infants by postpartum study arm and by combined antepartum/postpartum strategy.
4. To assess and compare overall infant mortality rates through 12 and 24 months post-delivery by postpartum study arm and by combined antepartum/postpartum strategy.
5. To evaluate adherence to the maternal and/or infant ARV regimens.
6. To assess rates and patterns of maternal and infant resistance according to the maternal and infant ARV strategies.
7. To evaluate cost effectiveness and feasibility of the trial ARV strategies.
8. To assess MTCT according to mode of infant feeding and other risk factors.
9. To describe ARV pharmacokinetics in breastfeeding mothers and their infants.
10. To identify and assess immune correlates of protection against postnatal HIV transmission.

Figure 2 – Postpartum Component Randomization (Step 1) in IMPAACT 1077BF (1077BP)

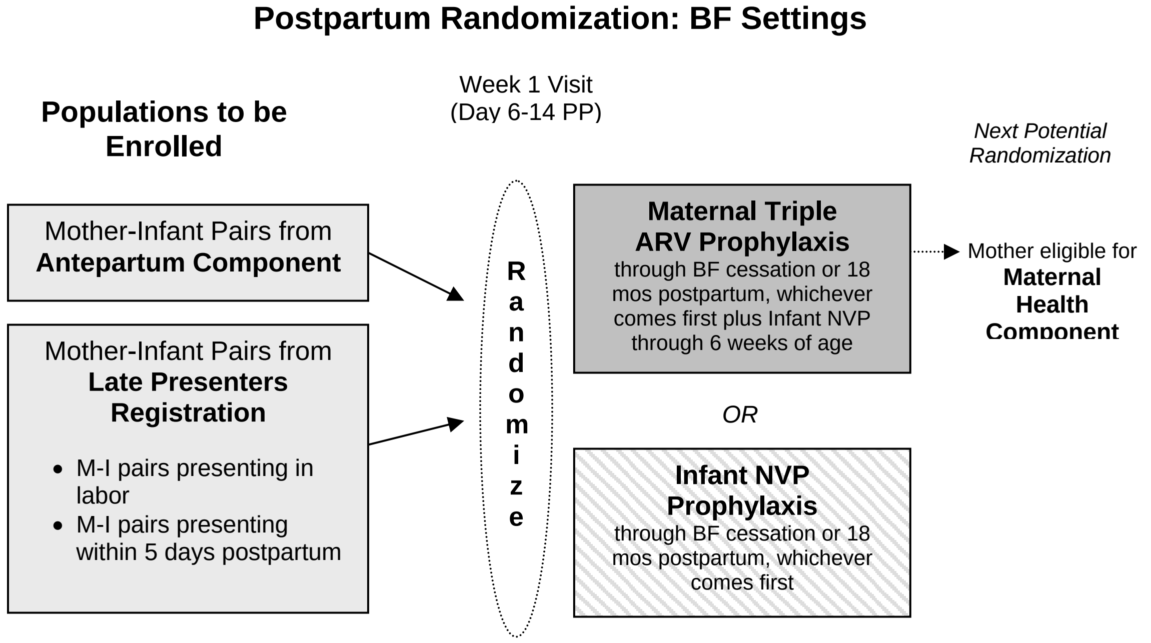

4.1 Rationale (Postpartum Component)

4.11 Rationale for Studying Infant ARV Prophylaxis and Maternal ARV Prophylaxis Regimen as Two Alternative Strategies to Reduce the Risk of MTCT during BF

In resource-limited settings, BF is the cultural norm, including among HIV-infected women. BF is also critical to infant survival and protects the infant against common infectious causes of infant mortality such as diarrhea and pneumonia. In a meta-analysis by the WHO Collaborative Study Team on the Role of Breastfeeding on the Prevention of Infant Mortality, BF was associated with a 6-fold (95% confidence interval [CI] 6-10) decrease in mortality due to infectious diseases for infants < 2 months of age. Protection persisted but declined with age during infancy with a 4-fold (95% CI 3-6) decrease for ages 2 to 3 months, a 3-fold (95% CI 2-4) decrease for ages 4 to 5 months, a 2-fold (95% CI 1-3) decrease for ages 6 to 8 months and 1.4-fold (95% CI 1-3) decrease for ages 9 to 11 months (1). Breast milk contains carbohydrates, fats, amino acids, minerals and vitamins as well as various growth-promoting factors, and provides optimal nutrition to infants, particularly during the first 12 months of life.

BF also provides benefits to the mother. BF delays resumption of ovulation resulting in increased child spacing. There are also psychosocial benefits of BF through promotion of maternal-infant bonding. In addition to individual health benefits, there are economic and social benefits due to savings from not needing to purchase breast milk alternatives for the child and decreased health care costs due to lowered rates of infant disease.

However, for an HIV-infected woman in resource-limited settings, BF also carries the risk of HIV transmission to her infant. This risk is about 0.6% to 0.9% transmission risk per month of late transmission (transmission after age 1 month) with early mixed feeding and somewhat lower with early exclusive BF (2). With prolonged BF into the second year, acquisition of infant HIV infection through breast milk contributes an estimated 30-50% of all MTCT. Thus, the optimal length of BF among HIV-

infected women is a major public health dilemma, and has led researchers to develop strategies to make breastfeeding safer.

Trial data from Zambia demonstrated that the overall benefit of extended breastfeeding into the second year afforded by protecting the HIV-exposed infant against diarrheal and other infectious causes of infant mortality led to similar 24-month HIV-free survival compared to early abrupt BF cessation by 4 months (3). Among the subgroup of women with higher CD4 counts ($\geq$ 350 cells/mm^3), BF into the second year was associated with better 24-month infant HIV-free survival than abrupt early BF cessation.

For many HIV-infected women in resource-limited settings, safe feeding alternatives to BF are neither feasible nor culturally acceptable. Currently, WHO recommends exclusive BF for six months unless AFASS criteria (availability of breast milk substitutes that are affordable, feasible, acceptable, sustainable and safe) for breast milk alternatives are met and continued BF through 12 months with introduction of appropriate complementary foods beginning at 6 months (3). Finding effective interventions to reduce the MTCT risk from BF is an urgent public health priority. Development of such interventions should also improve overall infant survival, since BF has been shown to confer protection against infant morbidity and mortality (4, 5).

Two general strategies have been proposed to reduce the risk of postpartum BF HIV transmission: 1) use of infant ARV prophylaxis during BF and 2) use of maternal triple ARV prophylaxis during BF. The infant ARV prophylaxis strategy aims to protect the infant during the period of HIV exposure during BF while maternal prophylaxis aims to reduce the risk of transmission primarily by lowering viral load in breast milk, although transfer of ARVs to the infant through breast milk ingestion also occurs, which could provide some indirect ARV prophylaxis to the infant (but also the potential for infant ARV toxicity).

Clinical Trial Data Supporting the Efficacy of Infant NVP Prophylaxis to Prevent Breast Milk HIV Transmission

A phase I/II study of infant NVP prophylaxis through age 6 months (HIVNET 023) established the dosing and safety of the daily NVP dosing of HIV-exposed infants through age 6 months (6).

Provision of infant NVP through the first 6-14 weeks of BF has been shown to reduce postnatal HIV transmission in the Six Week Extended-Dose Nevirapine (SWEN) and Post-Exposure Prophylaxis of the Infant (PEPI)-Malawi trials (7, 8) compared to the control arm interventions of sdNVP or sdNVP plus one week of infant ZDV, respectively, used in the two trials. The SWEN study carried out in Uganda, Ethiopia, and India reported a significant 50% reduction in postnatal HIV transmission at age 6 weeks for infants in the study arm who received 6 weeks of daily infant NVP compared to the sdNVP regimen (6 week postpartum transmission rate of 2.5% versus 5.3% respectively, p=.009), although this benefit was no longer statistically significant by 6 months (however, 6-month HIV-free survival remained significantly better in the extended infant NVP study arm). About 70% of women ceased BF by infant age 6 months. The PEPI-Malawi study reported a 67% reduction in postpartum transmission at 6 and 14 weeks for infants who were randomized to receive 14 weeks of either daily infant NVP or daily infant NVP/ZDV compared to sdNVP plus 1 week of daily ZDV, and a persistent significant 50% reduction in postnatal transmission at age 9 months (most infants ceased BF between ages 6-9 months). There were no significant differences in efficacy between the daily NVP and combination NVP/ZDV arms, but infants in the combination arm were more likely to have adverse events that were possibly associated with study drug, predominantly neutropenia.

In the SWEN study, infants who became infected despite extended NVP prophylaxis and were infected during the period of prophylaxis had an increased risk of selection for and persistence of NVP resistance mutations. Resistance data are not yet available for the PEPI-Malawi study.

Two earlier trials also provide background rationale for the use of infant ARV prophylaxis for up to 6 months of BF (the SIMBA and MITRA trials) (9, 10), but these trials did not have control arms as in the PEPI and SWEN studies. The SIMBA study from Rwanda gave mothers dual antenatal treatment with ZDV plus didanosine (ddI) from 34-36 weeks gestation through 1 week postpartum, and their breastfed infants received either NVP or 3TC daily prophylaxis for up to 6 months (average duration BF was only 3.5 months). The investigators reported a postpartum transmission rate at age 6 months of 2.4% in infants who were uninfected at age 4 days, with no difference between infant NVP vs. 3TC; median duration of BF was < 4 months.

The MITRA study was a one-armed, open-label study from Tanzania, which gave mothers antenatal dual treatment with 3TC-ZDV from 34-36 weeks through 1 week postpartum, and their breastfed infants received 3TC once daily. The overall transmission rate at 6 months was 4.9%; the postnatal transmission rate in BF infants who were HIV-uninfected at age 6 weeks was 1.1% at age 6 months; median duration of BF was 18 weeks.

Thus, there are data from several separate trials, including two randomized, controlled trials that support the use of infant NVP prophylaxis during the first months of life to reduce the risk of BF transmission. Recent results from HPTN 046, a study in which all infants received daily infant NVP through age 6 weeks and were then randomized to receive either daily infant NVP or placebo through 6 months of age, demonstrated that 6 months of infant NVP was significantly more effective at 6 months in reducing BF transmission than 6 weeks and adverse events did not differ between the nevirapine and placebo groups (11).

Clinical Studies Supporting the Efficacy of Maternal Triple ARV Regimens to Prevent Breast Milk HIV Transmission

There are also recent encouraging observational data suggesting a beneficial role for maternal triple ARV prophylaxis given in the third trimester up to 6 months postpartum for prevention of BF transmission; however, there are not yet randomized clinical trial data. This includes 4 open-label observational studies of maternal triple ARV regimens given during the third trimester through cessation of BF (up to 6 months) from in Kenya, Tanzania, Rwanda and Mozambique. Duration of BF was not provided in these reports.

The KiBs Study from Kisumu, Kenya, was an open-label observational study of 500 HIV-infected pregnant women who received HAART containing 3TC-ZDV and either nelfinavir (if CD4 count $\geq$ 250 cells/mm^3) or NVP (if CD4 count < 250 cells/mm^3) from 34 weeks gestational age through 6 months of BF; infants received sdNVP (12). The overall transmission at 6 and 12 months was 5.0% and 5.9%, respectively. The postnatal transmission rate between birth and age 6 months was 2.6% and 12 months was 3.5%; actual duration of BF was not specified.

The MITRA-PLUS study in Tanzania was an open-label observational study in which 501 HIV-infected pregnant women who received 3TC-ZDV/NVP from 34 weeks gestation through 6 months of BF; infants received 3TC-ZDV for a week (13). The overall transmission rate at 6 months was 5.0%. The postnatal transmission rate in BF infants who were HIV-uninfected at age 6 weeks was 0.9% at age 6 months; duration of BF was not specified. The overall and postpartum transmission rates are very similar to the results reported in the infant ARV prophylaxis MITRA study discussed above.

The AMATA study from Kigali, Rwanda was an open-label observational study in 316 FF women and 238 BF women who received HAART (d4T/3TC/NVP if CD4 < 350 cells/mm^3 or 3TC-ZDV/EFV if CD4 $\geq$ 350 cells/mm^3) from 26 weeks gestation through delivery if FF or 6 months postpartum if BF; infants received sdNVP and one week of ZDV (14). Infection status was available on 77% of infants; the 6 month overall transmission rate was 1.6% (undifferentiated by mode of infant feeding). The investigators report a postnatal transmission rate in BF infants who were uninfected at age 4 weeks of 0.6% at 6 months; however, the duration of BF was not provided.

Finally, in Mozambique in the DREAM study, pregnant women received NVP-based HAART from 25 weeks gestation through 6 months of BF; infant prophylaxis was not specified (15). Infection status was available on 74% of infants; the 6 month overall transmission rate was 2.2%. The investigators report a postnatal transmission rate in BF infants between 1 and 6 months of 0.8%, but the duration of BF was not provided.

It is important to note that BF infants who become infected despite maternal HAART may have virus with drug resistance mutations. In the KiBs study, ARV drug resistance (both NRTI and NNRTI mutations) emerged during the breastfeeding period among 67% of the 24 infants who became infected despite maternal HAART; in general, resistance was not detected on the initial positive DNA PCR test but at subsequent testing, suggesting resistance was likely due to transfer of ARV drug from mother to the infant via breast milk in the presence of actively replicating virus in the infant (16). Resistance mutations to NRTI drugs were detected in 4 of 6 (66%) infants and to NNRTI drugs in 6 of 6 (100%) infants whose mothers took NVP-based HAART; resistance mutations to NRTI drugs were detected in 10 of 10 (100%) infants and to PI drugs in 0 of 10 (0%) infants whose mothers took nelfinavir-based HAART.

Further data are available that support the use of extended antiretroviral prophylaxis through 6 months given either to the mother as HAART or to the infant as daily NVP during up to 6 months of breastfeeding. The BAN study was a large 2 x 3 factorial design study that compared a control regimen of intrapartum sdNVP plus 7 days of ZDV/3TC which all women and infants in the trial received to either extended maternal HAART (ZDV/3TC + either NVP or LPV/RTV) or infant daily NVP prophylaxis given for up to 7 months of exclusive breast feeding (17). Additional nutritional supplements were also part of the study design. Findings indicated that both extended maternal HAART and extended infant NVP prophylaxis were superior to the one-week control arm: at 28 weeks post-delivery, the postnatal transmission rates among infants uninfected at birth were 1.7% for the extended infant NVP arm and 2.9% for the extended maternal HAART arm, compared to 5.7% for the control arm. At 28 weeks, HIV-free survival was also significantly better for both extended maternal HAART arm (4.1%) and extended infant prophylaxis arms (2.3%) when compared to the control arm (7.0%).

Likewise, the Mama Bana study also suggests that maternal HAART is a highly effective intervention to reduce transmission among breastfeeding women (18). This Botswana trial, which compared a PI-based HAART regimen (AZT/3TC/LPV/RTV) and a triple nucleoside reverse transcriptase inhibitor HAART regimen started between 26-34 weeks and given through up to 6 months of exclusive breastfeeding, found overall transmission rates of 0.4% for the PI-based HAART and 1.8% for NRTI based HAART with an overall transmission rate of 1.1%.

While direct comparison between studies is difficult due to differing populations and possibly different durations of breastfeeding, based on the above data, both maternal HAART and infant NVP prophylaxis appear promising as public health approaches to reducing the risk of HIV transmission among HIV-infected BF women who do not require treatment for their own health while preserving the beneficial effects of breast milk on overall infant survival in resource limited settings, and WHO now recommends use of one of the two strategies. However to date, the relative risks and benefits of these two strategies (i.e., maternal HAART versus infant ARV prophylaxis) have not been directly compared. Additionally,

the safety of continuing either maternal or infant prophylaxis to allow longer duration of BF (e.g., past age 6 months) has not been evaluated. The PROMISE trial has been designed to compare the relative efficacy of daily maternal triple ARV prophylaxis (with six weeks of infant prophylaxis as recommended by WHO) to daily infant NVP prophylaxis given through BF cessation or through 18 months postpartum, whichever comes first unless stopped for toxicity, other medical reasons or confirmed infant HIV infection.

4.12 Safety and Pharmacokinetic Data on Infant NVP Prophylaxis

Infant NVP Prophylaxis: Pharmacokinetic and Safety Data from the HIVNET 023, SWEN and PEPI Trials

HIVNET 023: HIVNET 023 was a Phase I/II randomized open label clinical trial conducted in Zimbabwe and S. Africa to assess the safety and pharmacokinetics of 3 different NVP dosing regimens from birth to 6 months in BF infants born to HIV-infected women (6). The primary objective was to determine a NVP dosing regimen that was safe and maintained through NVP plasma concentration > 100 ng/mL (10 times the *in vitro* IC_{50} against HIV) continuously from birth to 6 months of life. Women received an oral dose of 200 mg of NVP to administer to themselves at the onset of labor. Seventy-five infants (including 1 set of twins) were randomized within 48 hours of birth to 1 of 3 study arms and received either weekly, twice weekly or daily regimens of NVP from birth through 6 months of age. Infants in the weekly and twice weekly arms received 4 mg/kg/dose for the first 2 weeks of life, then 8 mg/kg/dose through 24 weeks. In the daily arm, infants received 2 mg/kg/dose for the first 2 weeks of life, followed by 4 mg/kg/dose through 24 weeks. Pre-dose (trough) concentrations were determined at 2, 8, 16, 20 and 24 weeks of age using pharmacokinetic data from the Zimbabwe site.

Concentrations are available for between 10 and 17 infants in each treatment arm at each time point. Overall, none of the pre-dose samples from the daily dosing arm infants were below the therapeutic target of 100 ng/mL, while 3 of 65 (4.6%) of the twice-weekly samples and 48 of 75 (64%) of the weekly samples fell below the target. When all concentrations from all time points are combined for each treatment arm, median pre-dose NVP concentration was 1348 ng/mL (range: 108-4843 ng/mL) with daily dosing compared to 459 ng/mL (range: < 25-1386 ng/mL) with twice-weekly dosing and 64 ng/mL (range: < 25-1519 ng/mL) with weekly dosing.

No severe (Grade 3 or higher) skin rash, hepatic or renal toxicity related to NVP were observed. Three infants died (2 during follow-up, 1 after the 32-week visit). The causes of death included early-onset neonatal sepsis (1 infant, HIV-uninfected) and pneumonia (2, both HIV-infected with clinical evidence of AIDS). None of the deaths were attributable to study drug. The most frequent serious laboratory abnormality was neutropenia (Grade 3 or higher) noted in 8 infants (24%). Grade 3 or higher neutropenia was reported in 4 infants in the once-weekly arm, 4 in the twice-weekly arm, and none in the daily arm. The neutropenia resolved in all 8 infants; in seven infants, the neutropenia was transient, and infants continued on NVP dosing through 24 weeks. In one infant with persistent neutropenia, the abnormality resolved after NVP was permanently discontinued. Grade 3 or higher anemia was observed in two infants (6%). In both infants, anemia was judged to be possibly, but unlikely related to study drug. Two infants (6%) experienced Grade 3 or higher thrombocytopenia, unrelated to study drug; both infants were HIV-infected and the etiology was felt to be secondary to HIV infection. None of the enrolled infants had Grade 3 or higher elevations in serum ALT values. Thus, based on findings in HIVNET 023, NVP was deemed safe and well tolerated by HIV-exposed infants enrolled in the study.

SWEN Trial: The SWEN Study, conducted in Ethiopia, India and Uganda, was a randomized masked trial comparing the safety and efficacy of giving 6 weeks of extended daily infant NVP compared to sdNVP. The NVP doses used in the SWEN study were 2 mg/kg at birth followed by 5 mg once daily from days 8

to 42. There were no major safety concerns noted with extended use of NVP (7). The SWEN study reported a Grade 3 neutropenia rate of 10.4% in the extended infant NVP arm, compared to 8.9% the sdNVP anchor arm (p = 0.28); and a Grade 4 neutropenia rate of 3.3% in the extended NVP arm compared to 5% in the sdNVP arm (p = 0.08). There were no Grade 3 hepatic elevated ALT in either arm, and no Grade 4 hepatic ALT events in the SWEN arm compared to 0.1% Grade 4 ALT events in the sdNVP arm. Grade 3 rashes were rare in both arms, 0.7% in the extended 6 week arm and 0.5% in the sdNVP arm, and there were no Grade 4 rashes. The background rate of overall serious adverse events (SAEs) was high (generally due to unrelated infectious events requiring hospitalization) but was comparable across both study arms: 38.4% in the extended NVP arm compared to 39.9% in the sdNVP arm (p = 0.54)

PEPI-Malawi Trial: The PEPI-Malawi randomized trial compared a control arm of sdNVP plus 1 week of ZDV to the control arm plus either extended 14 weeks of daily infant NVP or 14 weeks of daily infant NVP + ZDV (8). The NVP doses used in the PEPI-Malawi study were 2 mg/kg at birth followed by 2 mg/kg once daily for days 8-14 (2nd week) and 4 mg/kg once daily for days 15-98 (3-14 weeks). In the study, the vast majority (87.3%) of SAEs were judged unrelated to study product. The most common SAEs judged to be possibly or probably associated with extended NVP or ZDV/ NVP were neutropenia, anemia and rash. The rates of SAE for infants in the control arm was 14.2% compared to 16.8% in the 14 week extended NVP arm and 22.4% in the ZDV/NVP 14 week extended arm. The most common SAE seen in the dual ZDV/NVP arm was neutropenia.

4.13 Age-based Infant NVP Dosing Regimen for Prevention of Breast Milk HIV Transmission

Several trials involving administration of NVP using age band dosing regimens to prevent breast milk HIV transmission through age 6 months are either underway or have recently been completed. HPTN 046 was a phase III trial comparing the efficacy and safety of 6 weeks vs. 6 months of NVP administered to breast feeding infants born to HIV-infected women for the prevention of vertical HIV transmission, which found 6 months to be more effective than 6 weeks in reducing postnatal infection (11). HPTN 046 used an age-based NVP dosing regimen with eight changes in dosing between the first week of life and age 6 months. The BAN and Kesha Bora studies are recently completed studies that compared no postnatal treatment, maternal HAART and infant NVP during breastfeeding over the first 6 months of life (18, 19). The infant NVP regimen used in both of these studies was a modified version of the HPTN 046 age-based regimen with doses rounded off so that there were only three dose changes during the first 6 months of life.

The WHO Pediatric Antiretroviral Working Group evaluated the age-based and weight-based NVP regimens. The Working Group concluded that they are not practical for widespread implementation in resource limited settings, because they are either too complicated and/or the timing of dose changes does not match the limited number of visits infants currently receive as part of standard well child care. The dosing regimen recommended by the Working Group and used in PROMISE is a simplified NVP regimen with dose changes that parallel the typical infant well-child care visit schedule:

- Birth to 6 weeks: 15 mg once daily if birth weight $\geq$ 2500 gm; 10 mg once daily if birth weight 2000 to 2499 gm
- Greater than Week 6 to 6 months: 20 mg once daily
- Greater than 6 months to 9 months: 30 mg once daily
- Greater than 9 months to weaning: 40 mg once daily

Note: For infants with birth weight less than 2000 gm, see Sections 2.512 and 3.414. (These infants are ineligible for the Postpartum Component as specified in Section 4.512.)

Figure 3 below shows the distribution of per kg doses that will be administered with the NVP dosing regimen in infants with weight between the 5th and 95th percentiles.

Figure 4 below shows the distribution of NVP pre-dose concentrations over the first 18 months of life from a simulation using the PROMISE dosing regimen and the DACS 095 population PK model, which incorporates changes in clearance from maturation associated with increasing age and from NVP autoinduction. IMPAACT DACS 095 is a population analysis of NVP PK data from five HIVNET and PACTG studies (20).

Figure 3 - Distribution of per kilogram NVP doses in infants

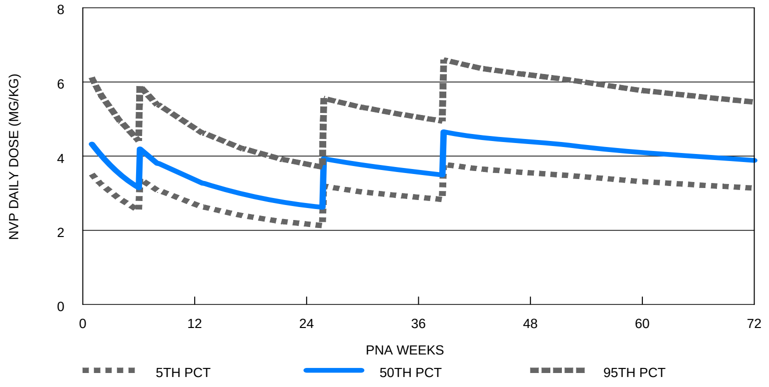

Figure 4 - Distribution of NVP pre-dose concentrations over the first 18 month of life

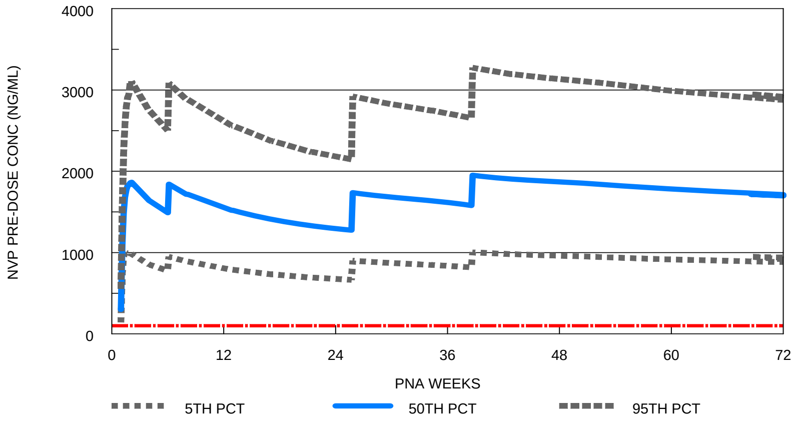

4.14 Infant NVP Prophylaxis and NVP Resistance

It is anticipated with the Antepartum Component and Postpartum Component regimens in PROMISE that very few of infants in the PROMISE trial will become infected (< 5%) given results from the above described studies using maternal HAART or infant NVP prophylaxis in the first 6 months postpartum. However, the risk of development of NVP resistance is well established if an infant becomes infected

despite receiving sdNVP. Similarly, in the SWEN study, infants who became infected while receiving the extended 6 week infant NVP prophylaxis regimen had an increased risk of selection for and persistence of NVP resistance mutations (21). However, infants who became infected after the period of NVP prophylaxis ended (after age 6 weeks) were less likely to have NVP resistance than infants who were infected *in utero*, peripartum or during the first 6 weeks of breastfeeding (22).

To ensure that the length of exposure to NVP will be reduced if an infant does become infected, frequent HIV nucleic acid testing (NAT) is scheduled for infants in the Postpartum Component of the PROMISE Protocol. Based on current WHO pediatric treatment guidelines (23), infants < 12 months of age who are identified as HIV-infected based on NAT should begin ARV treatment as soon as possible based on clinical trial findings, which showed significantly improved survival for infants who began therapy before age 3 months irrespective of CD4 count or symptoms. These WHO Guidelines recommend use of a PI-based HAART regimen (LPV-RTV) for initial therapy of infants with known exposure to sdNVP; similarly, infant who become infected despite infant NVP prophylaxis should be started on PI-based HAART when identified. LPV-RTV for children is available at all sites participating in PROMISE (outside of the study). HIV-infected children will be referred to local ART clinics for treatment.

4.15 Maternal Postpartum Triple ARV Prophylaxis Regimen

ZDV is a preferred nucleoside analogue drug for use as part of a triple ARV regimen when used solely for prevention of MTCT during pregnancy because of extensive safety experience. However, in the Postpartum Component of PROMISE, women randomized to receive the triple ARV regimen to prevent postnatal transmission could receive as long as 18 months of the study drugs. 3TC-ZDV is associated with bone marrow suppression, manifested by macrocytic anemia and neutropenia, and this may be exacerbated in resource-limited settings where baseline levels of maternal anemia may be high.

In HIV-infected non-pregnant adults in resource-rich countries, the fixed-dose combination of FTC-TDF (TRV) is a preferred dual NRTI combination for HAART regimens for individuals who require treatment for their own health (24). In the Gilead 934 randomized, controlled clinical trial, TDF, when used with either 3TC or FTC as part of an EFV-based regimen in treatment naïve patients demonstrated potent virologic suppression through 144 weeks and was superior to 3TC-ZDV in virologic and immunologic efficacy with these differences persisting to 144 weeks (25). More subjects in the 3TC-ZDV arm developed loss of limb fat at 96 and 144 weeks compared with the TRV arm. Also, emergence of the M184V 3TC/FTC resistance mutation was less frequent in patients receiving TRV than 3TC-ZDV; no patient developed the K65R mutation after 144 weeks of therapy in the TRV arm. While TRV has not been compared head-to-head with 3TC-ZDV as a dual NRTI backbone in a PI-based regimen, in a study comparing once and twice-daily LPV-RTV using TRV as the dual NRTI backbone, the 48 week virologic efficacy was similar to that seen in trials of LPV-RTV using 3TC-ZDV as the dual NRTI backbone in other treatment naïve patients, with 70% of patients having HIV RNA < 50 copies/mL at 48 weeks (26). Recent data from the Octane study also supports the potent viral suppression using FTC-TDF and Lopinavir/ritonavir with 93% of patients on that regimen showing undetectable viral loads.

Thus, based on these data, in the US, FTC-TDF (TRV) is a preferred dual NRTI backbone for a HAART regimen. 3TC-ZDV is viewed as an alternative dual NRTI backbone because of the superior virologic efficacy of TRV and the greater toxicity of 3TC-ZDV compared to the preferred regimen. Additionally, the WHO guidelines for treatment in pregnant or non-pregnant women include use of TRV as a preferred dual NRTI backbone for HAART (27). Therefore, because the duration of ARV exposure will be more prolonged when the postpartum triple ARV prophylaxis regimen is given (e.g., at least 6 months) than with an antepartum triple ARV prophylaxis regimen alone (or if an antepartum ARV prophylaxis regimen is combined with a postpartum triple ARV prophylaxis regimen) as well as the reasons above, the

PROMISE team has opted to use the TRV-based regimen as the preferred regimen for women randomized postpartum to triple ARV prophylaxis for prevention of breast milk MTCT.

However, for some women randomized to 3TC-ZDV/LPV-RTV in the Antepartum Component, the study clinician, after discussion with the study participant, may decide that switching the dual NRTI backbone from 3TC-ZDV to TRV is not in the best interest of the woman, and in such cases the woman may continue on 3TC-ZDV (consultation with the CMC available but not required).

The maternal triple ARV prophylaxis arm (Arm A) will be compared for efficacy, tolerability, safety and cost effectiveness to the infant NVP prophylaxis arm (Arm B) during the postpartum period of breastfeeding.

4.16 Safety

General information on the safety and efficacy of LPV-RTV and TRV are provided in Section 2.11. Studies in rats have demonstrated drug transfer into breast milk for TDF, FTC and LPV-RTV; data in humans are minimal. Preliminary data from HPTN 057 suggest very low levels of TDF in the breast milk of women following single dose TDF in labor (28).

TDF is the water-soluble diester prodrug of the active ingredient, tenofovir. The oral bioavailability of tenofovir by itself is poor; however, as the diester prodrug combined with disoproxil fumarate, the drug becomes water soluble and oral bioavailability is about 25% in fasting patients. Drug that would be excreted into milk would be in the tenofovir form of the drug (and not the prodrug TDF), and therefore is expected to have very little bioavailability to infants who ingest the breast milk.

Because of limited information about the secretion of TDF, FTC, and LPV-RTV (and other second-line ARVs that may be received in women with virologic failure) into breast milk, PROMISE will include a safety evaluation of the pharmacokinetics and drug exposure for breastfeeding mothers receiving a triple ARV regimen, transfer to breast milk and a determination of infant ARV exposure from breast milk during maternal ARV use in the first 50 mother-infant pairs receiving each drug of interest. Pharmacology studies are described more fully in Section 4.8.

The main toxicities of concern with TDF are renal and bone density changes based on animal and human studies. Because of limited data on maternal outcome with use of TDF during lactation and on infant outcome of infants potentially exposed to TDF in breast milk, PROMISE will also include a substudy that will compare bone and renal outcomes in women and their infants exposed to TDF during pregnancy to a subset of women and infants who were not exposed to TDF during pregnancy as described in Section 1.4.

4.2 Study Objectives (Postpartum Component)

4.21 Primary Objectives

4.211 To evaluate the comparative efficacy of giving daily maternal triple ARV prophylaxis versus daily infant NVP prophylaxis during BF to reduce cumulative HIV transmission from BF

4.212 To assess the safety and tolerability of these ARV regimens for mother and infant

4.22 Secondary Objectives

4.221 To assess the distribution of time to postnatal HIV transmission according to allocated study arms

4.222 To assess whether the relative efficacy of maternal triple ARV prophylaxis and infant NVP prophylaxis in preventing postpartum MTCT differs in early- (Antepartum Component enrollees)

versus late-presenting mothers or in early-presenting mothers who received triple ARV prophylaxis versus ZDV + sdNVP + TRV tail during pregnancy

4.223 To compare cumulative 24-month HIV-free survival among infants by postpartum study arm and by combined antepartum/postpartum strategy

4.224 To assess and compare overall infant mortality rates through 12 and 24 months post-delivery by postpartum study arm and by combined antepartum/postpartum strategy

4.225 To evaluate adherence to the maternal and/or infant ARV regimens

4.226 To assess rates and patterns of maternal and infant resistance according to the maternal and infant ARV strategies

4.227 To evaluate cost effectiveness and feasibility of the trial ARV strategies

4.228 To assess MTCT according to mode of infant feeding and other risk factors

4.229 To describe ARV pharmacokinetics in breastfeeding mothers and their infants

4.230 To identify and assess immune correlates of protection against postnatal HIV transmission

4.3 Study Design (Postpartum Component)

4.31 Mother-Infant Pairs from Antepartum Component and Late Presenters Registration

Participants for this study component will be recruited from two source populations: the Antepartum Component (see Section 2.0) and the Late Presenters Registration (see Section 3.0). Overall, approximately 3,100 BF mothers and their infants from the PROMISE Antepartum Component and approximately 1,550 from the Late Presenters Registration are anticipated to be eligible for the Postpartum Component.

The screening assessments for the Postpartum Component are included in Antepartum Component and Late Presenters informed consent forms so that eligibility assessments can be done as soon as possible; however, separate informed consent must be obtained for participation in the Postpartum Component before study entry. Randomization should occur at the week 1 (day 6-14) visit, after the mother and infant have met eligibility criteria (see Section 4.5).

Women who were randomized to receive the triple ARV prophylaxis regimen in the Antepartum Component will continue to receive triple ARV prophylaxis until the week 1 (day 6-14) postpartum visit, and then will be randomized to either receive FTC-TDF/LPV-RTV triple ARV prophylaxis regimen (with continuation of infant NVP prophylaxis (from the Antepartum Component) through six weeks of age) or discontinue their antepartum triple ARV prophylaxis regimen and continue daily infant NVP prophylaxis (from the Antepartum Component) based on the postpartum randomization.

Note: For some women randomized to 3TC-ZDV/LPV-RTV triple ARV prophylaxis regimen in the Antepartum Component, the study clinician, after discussion with the study participant, may decide that switching the dual NRTI backbone from 3TC-ZDV to FTC–TDF is not in the best interest of the woman, and in such cases the woman may continue on 3TC-ZDV (consultation with the CMC available but not required).

Women who were randomized to ZDV + sdNVP + TRV tail in the Antepartum Component will receive TRV postpartum after delivery for 7 days or the date of the week 1 visit (up to 14 days), whichever is later and their infants will receive NVP through six weeks (42 days) of life, regardless of whether they are enrolled in the Postpartum Component. Eligible mother-infant pairs will be randomized in the Postpartum Component at the week 1 visit (day 6-14) to either initiate the maternal FTC-TDF/LPV-RTV ARV regimen or to continue infant daily NVP prophylaxis beyond 6 weeks.

4.32 Following Mothers Enrolled in the Postpartum Component and Steps of Triple ARV Use

Mother-infant pairs eligible for the Postpartum Component will be randomized to one of the arms described below in 1077BP Step 1. For women who give birth to multiple infants (e.g., twins), all live born infants will be randomized to the same arm in 1077BP Step 1. Women who are randomized to the infant NVP prophylaxis arm (and thus are not receiving maternal triple ARV prophylaxis) who develop indications for ARV treatment for their own health will move to 1077BP Step 2. Additionally, women who are randomized to the maternal prophylaxis arm who reach an indication for triple ARV treatment for their own health while on triple ARV prophylaxis (but before meeting the criteria to switch to a second line regimen) or after having stopped triple ARV prophylaxis will move to 1077BP Step 2. Women receiving a triple ARV regimen, either as prophylaxis through 1077BP Step 1 Arm A or as therapy for their own health through 1077BP Step 2 who require a change in the triple ARV regimen because of clinical, immunologic and/or virologic failure or toxicity (see Section 4.722) will move to 1077BP Step 3.

If a woman randomized to Arm A (maternal prophylaxis) cannot continue a triple ARV regimen due to toxicity, and no alternative triple ARV regimen is available or recommended after consultation with the CMC, her infant(s) should continue or resume daily NVP dosing (as outlined in Section 4.612).

1077BP Step 1 - Randomization (Maternal visits follow Appendix IA):

Arm A: Maternal triple ARV prophylaxis (with infant NVP prophylaxis through six weeks of age) given through BF cessation or through 18 months postpartum, whichever comes first unless stopped for toxicity, other medical reasons or confirmed infant HIV infection (of all of the woman's study infants). The study-supplied triple ARV prophylaxis regimen is LPV-RTV plus TRV (fixed dose combination FTC-TDF).

Arm B: Infant NVP prophylaxis (no maternal prophylaxis) given once daily through BF cessation or through 18 months postpartum, whichever comes first, unless stopped for toxicity, other medical reasons or HIV infection. (See Section 4.612 for additional information on dosing continuation.)

1077BP Step 2 (Maternal visits follow Appendix IA):
A mother who reaches an indication for triple ARV therapy (HAART) for her own health as specified in Section 4.721 will be registered to this step. All mothers must complete a step change entry visit. If not on a triple ARV regimen, the 1077BP Step 2 Entry visit must be completed prior to initiation of HAART.

1077BP Step 3 (Maternal visits follow Appendix IA):
Participants who are being followed on a triple ARV regimen, either as prophylaxis through 1077BP Step 1 Arm A or as therapy for their own health through 1077BP Step 2 will register to this step if they meet the criteria for switching to a second line regimen as specified in Section 4.722. The 1077BP Step 3 Entry visit must be completed prior to the first dose of the second line regimen.

4.33 Following Infants Enrolled in the Postpartum Component (Appendix IB)

If an infant randomized to the infant prophylaxis arm (Arm B) must permanently discontinue the study NVP due to toxicity/intolerability, he/she will be provided an alternative regimen of 3TC through the study. (See Section 4.612 for regimen specifics.)

As noted above, breastfeeding infants of mothers randomized to Arm A (maternal prophylaxis) should continue or resume daily NVP dosing (as outlined in Section 4.612) through cessation of breastfeeding (unless HIV-infected) if the mother cannot continue a triple ARV regimen due to toxicity, and no alternative triple ARV regimen is available or recommended after consultation with the CMC.

CTX will be administered to all infants from 6 weeks of age through cessation of BF as defined above. CTX will be provided as standard of care by the site, outside of the PROMISE study. Infants found to be HIV-infected will receive CTX through 52 weeks of age as standard of care; after this point, CTX treatment will be based on WHO guidelines CD4 criteria.

Note: Section 6.0 includes Statistical Considerations for this study component (and all others).

4.4 Counseling on HIV and Infant Feeding (Postpartum Component)

The PROMISE trial will follow the current general WHO guidelines for HIV-infected mothers concerning infant feeding. Enrolled women will be counseled to exclusively breastfeed their HIV uninfected infants from birth for six months and to then introduce appropriate complementary foods thereafter and continue breastfeeding for the first 12 months of life. Breastfeeding should then only stop once a nutritionally adequate and safe diet without breast milk can be provided.

Exclusive BF (EBF) is defined as an infant's consumption of human milk without supplementation of any type (no water, no juice, no non-human milk and no foods) except for vitamins, minerals and medications. It is anticipated that almost all mothers in the study will have stopped breastfeeding by 18 months and also that the benefits of breastfeeding in terms of child survival are minimized by that age. Unless they are assured access to ARV prophylaxis outside of the study as standard of care, study mothers who are still breastfeeding at 18 months postpartum will be advised to stop breastfeeding at that time given that the postpartum study interventions (triple ARV maternal prophylaxis or infant NVP prophylaxis) will not be provided after 18 months. Women will also be counseled against pre-mastication (pre-chewing food by the mother or caregiver) of any foods to be given to infants in view of hygiene and reports of HIV transmission through premastication.

Counselors will receive training and guidance to provide uniform messages outlining the benefits of EBF and maintenance of breast health. BF counseling will begin antepartum. Mothers will receive additional counseling support at delivery (BF will be encouraged within the first hour of birth) and postnatal counseling. Counseling on infant feeding will also be continued at the scheduled follow up visits. Counseling regarding optimal duration of the cessation period, age-appropriate complementary and replacement foods as well as necessary conditions for their safe provision - will be based on materials from the World Health Organization and the local Ministry of Health (29).

Exclusive BF Compliance
A structured questionnaire of infant dietary consumption will be given at each clinic visit to determine infant feeding patterns including EBF. At the first visit, mothers will be asked how long they intend to BF. At subsequent visits, they will be asked about infant feeding practices including current BF status and total number of breast feeds over the last 24 hours. Infant consumption of other liquids or foods will also be assessed.

BF Problems
Women who experience BF problems will be encouraged to return to clinic for evaluation and care, even if not at a scheduled study visit.

Continued Reassessment of AFASS Criteria during PROMISE Visits
Each individual situation for BF will be reassessed at the week 22 and week 50 visits using the AFASS screening tool. In addition, as part of the infant feeding practices questionnaire, this will be assessed at 4 weekly intervals to determine whether breastfeeding has been stopped.

It is acknowledged that counseling guidance on when to cease BF for most HIV-infected BF women may vary by site depending on availability of other foods, agricultural cultural norms and country guidelines. However, it is expected that at a given PROMISE site, the majority of women participating in the study will choose to stop BF at similar times given the local context and cultural norms while also taking into account their own situation and preferences.

Women whose infants become HIV-infected will be encouraged to continue to breastfeed their infected infants according to WHO guidelines.

Participating sites will be queried annually regarding the usual and minimum duration of BF at that particular site, for use in data analyses (see Section 6.21).

Assessment of Maternal Food Security
Mothers' food security will also be assessed by means of the Revised Household Food Insecurity Access Scale (HFIAS). This is a tool developed by the Food and Nutrition Technical Assistance (FANTA) program and the Food and Agriculture Organization (FAO) and it consists of five questions which allows one to categorize the mother into one of three categories: relatively food secure; moderately food insecure; and severely food insecure. This food insecurity questionnaire will be administered to the mothers at the 6 and 26 week visit and thereafter every 12 weeks until week 98. Where there is evidence of food insecurity, as collected in this questionnaire, mothers will be referred to appropriate, site available food assistance programs.

4.5 Selection and Enrollment of Subjects (Postpartum Component)

4.51 1077BP Step 1 (Randomization; occurs at the week 1 visit (6-14 days post birth))

4.511 Inclusion Criteria (1077BP Step 1)

4.511.1 Participation in the Antepartum Component of PROMISE (see Section 2.0) or registered as a Late Presenter (see Section 3.0)
4.511.2 Intent to BF
4.511.3 Provided written informed consent
4.511.4 Has no plans to move outside of the study site area during the 24 months following delivery
4.511.5 Maternal CD4+ count ≥ 350 cells/mm^3 or greater than or equal to the country-specific threshold for initiation of treatment, if that threshold is > 350 cells/mm^3, from a specimen obtained within 30 days prior to entry in 1077BP.

NOTE: Specimen collection for CD4 and CD8 counts is not recommended during labor or within the first 24 hours postpartum. When more than one CD4 cell count from a specimen obtained within 30 days prior to entry into 1077BP is available, the count with the latest date should be used to determine eligibility for 1077BP.

4.511.6 Maternal laboratory values on a specimen obtained within 30 days prior to entry in 1077BP

- Hemoglobin ≥ 7.0 g/dL
- WBC ≥ 1,500 cells/mm^3
- ANC ≥ 750 cells/mm^3
- Platelets ≥ 50,000 cells/mm^3
- ALT ≤ 2.5x ULN
- Estimated creatinine clearance of ≥ 60mL/min using the Cockroft-Gault equation for women (See 2.411.5)

4.511.7 Infant alive, healthy, ≤ 14 days of age, and uninfected (negative HIV NAT result on specimen drawn prior to entry in 1077BP)

4.511.8 Infant lab values on specimens obtained prior to entry in 1077BP (within 14 days of birth)*

- Hemoglobin ≥ 10 g/dL
- WBC ≥ 1,500 cells/mm^3
- ANC ≥ 750 cells/mm^3
- Platelets ≥ 50,000 cells/mm^3
- ALT ≤ 2.5x ULN

4.511.9 *For Registered Late Presenters*: Confirmed maternal HIV-1 infection, defined as documented positive results from two samples collected at different time points at any time prior to entry in 1077BP. (Note: Results of testing performed on sample #1 were required prior to LP registration; results of the testing performed on the second sample are required prior to entry into the PP Component.)

Sample #1 may be tested using any of the following:

- Two rapid antibody tests from different manufacturers or based on different principles and epitopes
- One EIA OR Western Blot OR immunofluorescence OR chemiluminescence
- One HIV DNA PCR
- One quantitative HIV RNA PCR (> 5,000 copies/mL)
- One qualitative HIV RNA PCR
- One total HIV nucleic acid test

Sample #1 may be tested by non-study public or PEPFAR programs. However, both the result and the sample collection date must be recorded in the subject's chart. Source documentation (patient's medical record/chart, MOH register, laboratory results, etc.) must be available if requested.

Sample #2 may be tested using any of the following:

- One EIA confirmed by Western Blot OR immunofluorescence OR chemiluminescence
- One HIV DNA PCR
- One quantitative HIV RNA PCR (> 5,000 copies/mL)
- One qualitative HIV RNA PCR
- One total HIV nucleic acid test

Sample #2 must be tested in a laboratory that operates according to GCLP guidelines, participates in appropriate external quality assurance programs and is approved by the IMPAACT Central Laboratory.

*In the case of a multiple birth, a mother-infant pair will be included in the postpartum component only if both/all infants and their mother meet the eligibility criteria. If only one infant of a multiple birth is alive, the mother-infant pair may be enrolled if the infant and the mother otherwise meet all of the eligibility criteria.

4.512 Exclusion Criteria (1077BP Step 1)

4.512.1 Positive infant HIV NAT result on specimen drawn prior to entry in 1077BP or no infant HIV NAT result on specimen drawn prior to entry in 1077BP

4.512.2 Life threatening infant illness or birth condition incompatible with life

4.512.3 Infant birth weight < 2.0 kg

4.512.4 Social or other circumstances which would hinder long-term follow-up as judged by the site investigator

4.512.5 Current or history of TB disease (positive PPD without TB disease is not exclusionary)

4.512.6 Current documented conduction heart defect (specialized assessments to rule out this condition are not required; a heart murmur alone and/or type 1 second-degree atrioventricular block (also known as Mobitz I or Wenckebach) is not considered exclusionary)

4.512.7 Requires triple ARV therapy (HAART) for own health (includes women who are on Step 2 of the Antepartum Component and women who are on Step 3 of the Antepartum Component for immunologic/clinical disease progression requiring a change in their triple ARV regimen).

Note: Women on Step 3 of the Antepartum Component who were never on Step 2 and who entered Step 3 for toxicity or virologic failure without clinical or immunologic disease progression requiring a complete change in the triple ARV regimen are eligible for the Postpartum Component.

4.52 <u>1077BP Step 2</u>

4.521 Inclusion Criteria (1077BP Step 2)

4.521.1
- On 1077BP Step 1 Arm B (infant prophylaxis); OR
- On 1077BP Step 1 Arm A (maternal prophylaxis) and currently receiving triple ARV prophylaxis but does not meet the criteria for switching to a second line regimen and entry into 1077BP Step 3; OR
- On 1077BP Step 1 Arm A (maternal prophylaxis) and not enrolled in the Maternal Health Component but remain in observational follow-up and are not currently receiving a triple ARV regimen (stopped the regimen)

4.521.2 Reached an indication for triple ARV therapy (HAART) as specified in Section 4.721

4.521.3 Willing and able to initiate HAART

4.522 Exclusion Criteria (1077BP Step 2)

None.

NOTE: A participant should not move to a new step if she has a toxicity that, based on the Toxicity Management Guidelines (Appendix II), would necessitate an interruption in therapy; however, the participant may move to the new step once the toxicity has resolved to a grade that would allow therapy to begin.

4.53 <u>1077BP Step 3</u> (Mothers from either 1077BP Step 1 Arm A, currently receiving triple ARV prophylaxis or 1077BP Step 2 currently receiving triple ARV regimen for treatment who require a change in their triple ARV regimen)

4.531 Inclusion Criteria (1077BP Step 3)

4.531.1 On 1077BP Step 1 Arm A or on Step 2

4.531.2 Met the criteria for switching to a second line regimen, as specified in Section 4.722, while on a triple ARV regimen

4.531.3 Willing and able to initiate an alternate triple ARV regimen

4.532 Exclusion Criteria (1077BP Step 3)

4.532.1 Women on 1077BP Step 1 Arm A who were not enrolled in the Maternal Health Component but remain in observational follow-up and are not currently receiving a triple ARV regimen

NOTE: A participant should not move to a new step if she has a toxicity that, based on the Toxicity Management Guidelines (Appendix II), would necessitate an interruption in therapy; however, the participant may move to the new step once the toxicity has resolved to a grade that would allow resumption of therapy.

4.54 Enrollment Procedures

Requirements for sites to initiate IMPAACT 1077BF, beginning with the two points of entry - the Antenatal Component and the Late Presenters Registration - are outlined in Section 2.44 and will be detailed in the study Manual of Procedures.

As noted previously, subject enrollment is done through the Data Management Center (DMC) Subject Enrollment System. The appropriate enrollment screen for this component is identified as 1077BP. As with each individual component, written informed consent for participation in the Postpartum Component must be obtained before entry.

4.55 Maternal and Infant Co-Enrollment Procedures

PROMISE participants will be encouraged to take part in IMPAACT P1084s and P1026s, where available; no prior approval is required. Participants are discouraged from participating in other clinical trials of investigational agents during the time of the trial. Co-enrollment in PROMISE and other clinical trials may be considered on a case-by-case basis and requires the approval of the protocol chairs of both trials.

Infants who become infected during follow up in PROMISE may co-enroll in treatment protocols if they meet eligibility criteria. Each case will be reviewed and requires the approval of the protocol chairs of both trials.

4.6 Study Treatment (Postpartum Component)

The postpartum intervention will start at the week 1 (day 6-14) visit; infant blood for HIV NAT will be obtained at this visit. For women continuing the triple ARV regimen from the Antepartum Component (1077BA), study drugs should be started immediately, and there should be no gap between the two regimens. For all women, study medications are to be started within 72 hours of entry to 1077BP Step 1. Likewise, for HIV-uninfected infants continuing daily NVP prophylaxis from the Antepartum Component (1077BA), the next dose of study drug should be given immediately.

At entry to 1077BP Step 1 subjects will be randomized to one of two arms:

Arm A:

Mothers:

- Emtricitabine-Tenofovir disoproxil fumarate fixed dose combination tablet 200 mg/300 mg orally daily from the week 1 postpartum visit until up to two weeks after complete BF cessation is achieved (defined as completely stopping all exposure to breast milk for ≥ 28 days), i.e., up to 42 days after last exposure to breast milk, 18 months postpartum, or entry into the Maternal Health component, whichever comes first unless stopped for toxicity, other medical reasons or confirmed infant HIV infection (of all the mother's infants). Note that a mother with multiple infants (e.g., twins) will not discontinue her study treatment for the criterion of infant HIV infection if at least one of her study infants remains uninfected.

- Lopinavir/ritonavir fixed dose combination tablet 200 mg/50 mg x 2 tablets for a single dose of 400 mg/100 mg and a total daily dose of 800 mg/200 mg orally twice daily from week 1 postpartum visit until up to two weeks after complete BF cessation is achieved (defined as completely stopping all exposure to breast milk for ≥ 28 days), i.e., up to 42 days after last exposure to breast milk, 18 months postpartum, or entry into the Maternal Health component, whichever comes first unless stopped for toxicity, other medical reasons or confirmed infant HIV infection (of all the mother's infants). Note that a mother with multiple infants (e.g., twins) will not discontinue her study treatment for the criterion of infant HIV infection if at least one of her study infants remains uninfected.

Infants:

- Nevirapine age-based daily dosing (see Section 4.612, Table 1) starting from the week 1 postpartum visit (day 6-14 postpartum) (as a continuation of the daily dosing regimen begun within 5 days of birth in the Antepartum Component or Late Presenters Registration) and continuing through 42 days of age or through the Week 6 visit, whichever is later. See Section 4.612 for additional information on continuation of dosing.

****OR****

Arm B:

Mothers:

- Mothers will not receive any study drug during the Postpartum Component.

Infants:

- Nevirapine age-based daily dosing (see Section 4.612, Table 1) starting from the week 1 postpartum visit (day 6-14) (as a continuation of the daily dosing regimen begun in the AP Component or LP Registration) and continuing for up to two weeks after complete BF cessation is achieved (defined as completely stopping all exposure to breast milk for ≥ 28 days; i.e., for up to 42 days after last exposure to breast milk) or 18 months postpartum, whichever comes first, unless stopped for infant HIV infection, toxicity or other medical reasons. If a mother has multiple infants, such as twins, an infant with HIV infection must stop study NVP, but the other infant(s) can continue study drug. Infants who discontinue NVP due to toxicity/intolerability will be provided an alternative regimen of lamivudine (3TC). See Section 4.612 for additional information on continuation of study drug dosing and the alternative regimen.

At enrollment/randomization, it is expected that the assigned maternal or infant regimens (listed above) will use study-supplied study drugs. Thereafter, if one or more of the assigned study-supplied study drugs cannot be tolerated, the regimen may be modified (in consultation with the CMC if required per Appendix II) using study-supplied study drugs (see listing in Section 4.61) and/or drugs from other

sources. In addition, for mothers who may have experienced intolerance or toxicity to one or more of the ARVs included in the preferred maternal regimen while pregnant, consideration may be given to use of alternative regimens from the time of randomization into 1077BP, in consultation with the CMC. Study site clinicians are advised to consult the CMC regarding such participants in advance of the Week 1 postpartum visit, at which randomization into 1077BP takes place.

Note: Mothers who have a medical indication for HAART in 1077BP Step 2 or Step 3 may take a regimen agreed upon by their health care provider and themselves and may use any study-provided antiretroviral medication to construct a regimen or any other locally available antiretroviral medication provided that it meets the protocol definition of HAART and is provided by prescription.

Regardless of source, all maternal triple ARV regimens must include three or more agents from two or more classes of antiretroviral drugs. All ARVs should be prescribed consistent with current package inserts. Fixed dose FTC-TDF-RPV may be used as an alternative first line regimen for mothers who are not able to tolerate or adhere to LPV-RTV or ATV-RTV. Given that FTC-TDF-RPV has thus far only been studied as a first line regimen, consultation with the CMC is required in advance of prescribing this regimen for any study participant.

Second-line maternal regimens are not defined by this protocol and should be determined at the discretion of study site clinicians.

4.61 Drug Formulations, Duration, and Regimens

4.611 Formulations of Study-Supplied Drugs

Generic name<br>Abbreviation<br>Trade name	Formulation	Appearance	Storage
Zidovudine<br>ZDV<br>Retrovir®	300 mg tablets	Biconvex, white, round, film-coated tablets	15-25°C (59-77°F)
Lamivudine<br>3TC<br>Epivir®	300 mg tablets	Gray, modified diamond-shaped, film-coated tablets	25°C (77°F) - Excursions permitted to 15-30°C (59-86°F). See USP Controlled Room Temperature.
Lamivudine<br>3TC<br>Epivir®	10 mg/mL oral solution	A clear, colorless to pale yellow, strawberry-banana flavored liquid	Store in tightly closed bottles at 25°C (77°F). See USP Controlled Room Temperature.
Lamivudine-Zidovudine<br>3TC/ZDV<br>Combivir®	150 mg/300 mg tablets	White, modified capsule shaped, film-coated tablet	2-30°C (36-86°F)
Tenofovir Disoproxil Fumarate<br>TDF<br>Viread®	300 mg tablets	Almond-shaped, light blue, film-coated tablets	25°C (77°F) – Excursions permitted between 15-30°C (59-86°F). See USP Controlled Room Temperature.
Emtricitabine/Tenofovir Disoproxil Fumarate<br>FTC/TDV<br>Truvada®	200 mg/300 mg tablets	Blue, capsule shaped, film-coated tablet	25°C (77°F) - Excursions permitted between 15-30°C (59-86°F). See USP Controlled Room Temperature. Keep container tightly closed. Each bottle contains a silica gel desiccant canister that should remain in the original container to protect the product from humidity.
Lopinavir/Ritonavir<br>LPV-RTV<br>Kaletra®<br>Aluvia®	200 mg/50 mg tablets	Ovaloid, film-coated tablets that will be either red or yellow	20-25°C (68-77°F) - Excursions permitted between 15-30°C (59-86°F). See USP Controlled Room Temperature.
Ritonavir<br>RTV<br>Norvir®	100 mg tablets	White film-coated ovaloid tablets	20-25°C (68-77°F) - Excursions permitted to 15-30°C (59-86°F). See USP Controlled Room Temperature.
Atazanavir<br>ATV	150 and 300 mg capsules	White body with a charcoal gray top with ATV 150 or 300 on both the body and the cap	25°C (77°F) - Excursions permitted between 15-30°C (59-86°F). See USP Controlled Room Temperature.
Didanosine<br>ddI	400 mg and 250 mg capsules	may vary	25°C (77°F) - Excursions permitted between 15-30°C (59-86°F). See USP Controlled Room Temperature. Store in tightly closed containers.
Efavirenz<br>EFV	may vary	may vary	25°C (77°F) - Excursions permitted between 15-30°C (59-86°F). See USP Controlled Room Temperature. Store in tightly closed containers.

Tenofovir Disoproxil Fumarate/Emtricitabine/Rilpivirine<br>TDF/FTC/RPV<br>Complera®	300 mg/200 mg/25 mg tablets	Purplish-pink, capsule-shaped, film-coated, with "GSI" on one side	25^{0}C (77^{0}F) - Excursions permitted to 15-30^{0}C (59-86^{0}F). See USP Controlled Room Temperature. Store in tightly closed containers.
Nevirapine<br>NVP<br>Viramune®	10 mg/mL oral suspension	White to off-white preserved suspension	25°C (77°F) - Excursions permitted to 15–30°C (59–86°F). See USP Controlled Room Temperature.

4.612 Duration

Arm A (Maternal Prophylaxis Arm): Cessation of BF is defined as completely stopping all exposure to breast milk for ≥ 28 days, based on maternal report. The study drugs for women will be continued until up to two weeks after complete BF cessation as defined above is achieved (i.e., up to 42 days after last exposure to breast milk) or 18 months postpartum (week 72-76 postpartum*), whichever comes first, unless stopped for confirmed infant HIV infection (of all the woman's study infants), toxicity, or other medical reasons. Note that a mother with multiple infants (e.g., twins) will not discontinue her study treatment for the criterion of confirmed infant HIV infection if at least one of her study infants remains uninfected.

*Note: for women who are still breastfeeding at 18 months postpartum and potentially eligible for the Maternal Health Component, the triple ARV regimen should be continued while eligibility for the Maternal Health Component is determined (until either enrolled or determined to be ineligible or unwilling), with continued dosing permitted through week 78 postpartum.

Arm B (Infant Prophylaxis Arm): After the first six weeks of age, the study drug for infants (NVP) will be continued for up to two weeks after complete BF cessation as defined above is achieved (i.e., up to 42 days after last exposure to breast milk) or to 18 months postpartum (week 72-76), whichever comes first, unless stopped for infant HIV infection (of all the woman's study infants), toxicity, or other medical reasons. If an infant in Arm B must discontinue NVP due to toxicity/intolerability, he or she will be provided lamivudine (3TC) as an alternative study drug (see regimen below).

Study drug dosing for infants in either study arm:

- Any infant who vomits within 60 minutes of ingesting study drug may be re-dosed one time following vomiting.
- Infants will continue daily NVP prophylaxis through six weeks of age even if BF is ceased (unless stopped due to HIV infection, toxicity, or other medical reasons).
- Infants with a positive HIV test result should have the study drug held; if HIV infection is subsequently confirmed or cannot be ruled out, the study drug should be permanently discontinued. See instructions below for delayed initiation of NVP dosing and gaps in dosing.
- Regardless of HIV infection status and/or whether study drug dosing is discontinued, infants will remain in study follow-up through age 104 weeks.
- If a mother has multiple infants, such as twins, any infant with HIV infection must stop study drug; however, the other infant(s) can continue the study drug.

Infant NVP will be administered according to age as described in Section 4.13 and shown in the table below.

Table 1: Once Daily Nevirapine Prophylaxis Doses for Infants

Infant Age	Total Daily Dose (mg)	Volume of Nevirapine Suspension to Administer (mL)
Birth to 6 Weeks*	If birth weight ≥ 2500 gm: 15mg	1.5 mL
	If birth weight 2000–2499 gm: 10mg	1.0 mL
> 6 Weeks to 6 Months	20mg	2.0 mL
> 6 Months to 9 Months	30mg	3.0 mL
> 9 Months to Cessation of BF or 18 months, whichever is first	40mg	4.0 mL

*Infant dosing will begin as part of the AP Component study drug regimen; for those enrolled in the PP Component, continuation of the regimen from entry (Visit 1) at 6-14 days after delivery will be considered part of the PP Component study drug regimen. (Infants not enrolled in the PP Component will continue dosing as part of the AP Component or LP Registration regimen through six weeks of age.)

Infants who must discontinue study NVP due to toxicity/intolerability will be provided the following alternative regimen of lamivudine (3TC):

< 4 weeks of age: 2 mg/kg every 12 hours
≥ 4 weeks of age: 4 mg/kg every 12 hours

Mothers will receive oral syringes of the appropriate size and calibration increments, and instructions for dosing the oral suspension to their infants. Sites will be responsible for providing the oral syringes for the study. Ordering information for oral syringes will be provided in the study Manual of Operations (MOP) or by contacting the protocol pharmacist. Mothers will receive at least enough study drug and syringes to dose their infant until the next scheduled visit. Clinic staff may request additional drug and syringes in the event that the mother cannot return to the clinic on the infant's scheduled visit date but can return within the infant's visit window.

If a mother informs the site staff that they will not be able to return for a follow-up visit during the entire next visit window, the mother may be provided study drug sufficient until the next scheduled visit (i.e., 1 month additional drug if being seen every 4 weeks, 3 months additional drug if being seen every 12 weeks). Site staff must determine whether the infant will pass an age-related dose modification in the interim between visits and provide adequate drug supplies and instructions to the mother to ensure appropriate dosing.

Delayed Initiation and Gaps in Infant Study Drug Dosing

For delays in initiation of infant NVP dosing or gaps in NVP dosing during the first six weeks of life, the instructions provided in Section 2.6 should be followed.

After the first six weeks of life, infants with a gap in NVP dosing of 21 days or more after initiation must have a negative HIV NAT result on a specimen obtained at the study visit when the gap was identified or within the previous 21 days and must also have been exposed to breast

milk within the last 28 days or dosing cannot be resumed. Dosing should be resumed at the level appropriate for the infant's age as shown in Table 1.

4.613 Infant Cotrimoxazole Prophylaxis

Cotrimoxazole (CTX) should be provided to all infants in this component as standard of care beginning at 6 weeks of age and continuing up to 42 days after last exposure to breast milk (up to two weeks after complete BF cessation as defined above is achieved). Supplies of CTX should be obtained from non-study sources and, therefore, CTX is NOT considered a study drug for this component. The dose to be given will be determined by the site clinician. Infants identified as HIV-infected will receive cotrimoxazole through 52 weeks of age and thereafter based on WHO or local ministry of health guidelines.

4.62 Administration

Atazanavir and Tenofovir disoproxil fumarate-Emtricitabine-Rilpivirine (FTC-TDF-RPV, Complera) must be given with food; all other study drugs may be given with or without food.

4.63 Study Drug Supply

The study-supplied study drugs available for infants in this component are Nevirapine (NVP) suspension (obtained from Boehringer-Ingelheim) and Lamivudine (3TC) suspension (provided by GlaxoSmithKline). The study-supplied study drugs available for mothers in this component are; Zidovudine (ZDV), Lamivudine (3TC) tablets and fixed dose combination Combivir (3TC-ZDV) (provided by GlaxoSmithKline); Tenofovir disoproxil fumarate (TDF), fixed dose combination Emtricitabine-Tenofovir disoproxil fumarate (FTC-TDF, Truvada, TRV), and fixed dose combination Emtricitabine-Tenofovir disoproxil fumarate-Rilpivirine (FTC-TDF-RPV, Complera) (provided by Gilead Sciences); Lopinavir-Ritonavir (LPV-RTV) and Ritonavir (RTV) (provided by Abbott); Atazanavir (ATV) (obtained from Emcure Pharmaceuticals); and Didanosine (ddI) and Efavirenz (EFV) which will be obtained from a pharmaceutical supplier. However, all study-supplied drugs may not be available at all study sites; availability will be based on the status of drug regulatory approval for each ARV in each country.

4.64 Drug Distribution and Accountability

See Section 2.515.

4.7 Subject Management (Postpartum Component)

4.71 Management of Mother-Infant Pairs Randomized into 1077BP Step 1 following the Antepartum Component and Late Presenters

HIV-infected women who took part in the Antepartum Component or the Late Presenter Registration who intend to BF will have provided consent for the screening assessments for the Postpartum Component as part of the Antepartum Component or LP Registration informed consent process; however, they will need to provide separate consent for participation in the Postpartum Component (for themselves and their infants) prior to entry.

4.711 1077BP STEP 1: Randomization into the Postpartum Component (1077BP Step 1 schedule of evaluations: Appendix IA for Mothers; Appendix IB for Infants)

Entry and randomization into the Postpartum Component will occur at the week 1 (day 6-14) visit, after the mother and infant have met the eligibility criteria in Section 4.51.

1077BP STEP 1: Randomization

	Mothers	Infants
Arm A	Maternal triple ARV prophylaxis	Infant prophylaxis through 6 weeks of age
Arm B	No Maternal prophylaxis	Infant NVP prophylaxis for up to 18 months while BF

4.712 Maternal Triple ARV Prophylaxis Randomization (with Infant Prophylaxis for 6 Weeks)

Mothers of mother-infant pairs randomized to the maternal triple ARV prophylaxis arm in 1077BP Step 1 will be advised to initiate the triple ARV prophylaxis regimen on the day of randomization (day 6-14 postpartum). All women (regardless of antepartum randomization and HIV/HBV co-infected status) will be advised to initiate TRV/LPV-RTV. Likewise, for infants continuing daily NVP prophylaxis from the Antepartum Component or Late Presenter Registration, the next dose of study drug should be given on the day of randomization; continuation of the regimen from Postpartum Component entry (Week 1 visit) at 6-14 days after delivery will be considered part of the PP Component study drug regimen.

Women randomized to triple ARV prophylaxis in the Postpartum Component will continue study drug dosing through complete cessation of BF (as defined above) or through 18 months postpartum (week 72-76 postpartum), whichever comes first, and until randomization into the Maternal Health Component (if eligible and willing; see Section 5.3) unless study drug is stopped early for toxicity, other medical reasons or confirmed infant HIV infection (of all the mother's infants). The window for the Maternal Health randomization is up to two weeks after complete BF cessation as defined above is achieved with no plans to re-initiate BF with current infant. See Section 4.715 for management of infants with positive HIV NAT after randomization (and their mothers).

<u>1077BP STEP 1 FOLLOW-UP</u>

Women will follow the schedule of evaluations listed in Appendix IA, and infants will follow the schedule of evaluations in Appendix IB. Monitoring and ARV management of women is described in Section 4.72.

4.713 Infant NVP Prophylaxis Randomization (no Maternal Prophylaxis)

Infants of mother-infant pairs randomized to the infant NVP prophylaxis arm in 1077BP Step 1 will continue the daily infant NVP regimen begun as part of the AP Component or Later Presenter Registration; continuation of the regimen from Postpartum Component entry (Week 1 visit) at 6-14 days after delivery will be considered part of the PP Component study drug regimen. Randomized infants will follow the infant schedule of evaluations listed in Appendix IB. Infants should continue on their study drug until up to two weeks after complete BF cessation as defined above is achieved or through 18 months of age (week 72-76), whichever comes first, unless NVP is stopped for toxicity, other medical reasons or for HIV infection.

Mothers whose infants were randomized to the infant NVP prophylaxis arm in the Postpartum Component (and who therefore are not receiving triple ARV prophylaxis) will be followed on the same study visit schedule as women who were randomized to the maternal triple ARV prophylaxis arm (Appendix IA); visits will include regular clinical and CD4 count monitoring.

These women will initiate a triple ARV regimen for treatment (HAART) in Step 2 if they experience clinical progression to an AIDS-defining illness or have a confirmed CD4 count below 350 cells/mm^3 or below the country-specific threshold for initiation of treatment, if that threshold is > 350 cells/mm^3 (see Section 4.721).

Infants randomized to infant NVP prophylaxis in the Postpartum Component whose mothers require HAART for their own health (enter 1077BP Step 2) should continue their study NVP as per the protocol. See Section 4.715 for management of infants with positive HIV tests after randomization (and their mothers).

<u>1077BP STEP 1 FOLLOW-UP</u>
Mothers will follow the schedule of evaluations in Appendix IA and infants will continue to follow the schedule of evaluations in Appendix IB. Monitoring and ARV management of women is described in Section 4.72.

4.714 Management of Mother-Infant Pairs from the Antepartum Component who are Ineligible for 1077BP Step 1 Randomization

Women who do not meet eligibility criteria for the Postpartum Component and require a triple ARV regimen for their own health (see Section 2.613.1) will enter 1077BA Step 2 if they meet the criteria in Section 2.42.

Women who do not meet eligibility criteria for the Postpartum Component for reasons other than requiring treatment or who decline participation in the Postpartum Component but agree to continue follow-up, will be off study drug treatment, but will remain on study and continue to be followed observationally according to the schedule of maternal evaluations in Appendix IA through the end of the study (96 weeks after the last woman enrolled in the Antepartum Component delivers) or, for women on Step 1, until they meet criteria for entering 1077BA Step 2 (Section 2.42, Section 2.613.1).

Women who were randomized to Step 1 Arm A (maternal triple ARV prophylaxis) during pregnancy but who are not eligible or decline participation in the Postpartum Component may be eligible for randomization in the Maternal Health Component (Section 5.31). If such women need HAART for their own health (see Section 2.613.1), either at the time that eligibility for the Maternal Health Component is being determined or any time later, they will enter Step 2 of the Antepartum Component (1077BA) if they meet the eligibility criteria specified in Section 2.42.

All infants will continue to be followed through age 104 weeks according to the schedule of infant evaluations in Appendix IB and will receive NVP through six weeks of age.

4.715 Management of Infants with a Positive HIV Test after Randomization (and their Mothers)

Infants who have a positive HIV test result should have study drug held and a second test performed as soon as possible on a separate sample, collected on a different day. For infants in either arm who were on study drug at the time when of their first positive HIV test result, study drug should be held. For infants with confirmed HIV infection (or in whom infection cannot be ruled out following the initial positive test) study drug must be permanently discontinued; however, infected infants should continue to be followed in 1077BP per Appendix IB through 104 weeks of age. Infants should be referred for care and treatment according to local standard procedures. Infected infants should receive CTX as standard of care (non-study drug) through 52 weeks of age and thereafter based on WHO guidelines and local standards of care

Note that if a mother has multiple infants, such as twins, the infant with HIV infection must stop study drug, but the other infant(s) can continue the study drug. If the mother of multiple infants had been randomized to maternal triple ARV prophylaxis in Postpartum Component 1077BP Step 1, and one infant has HIV infection but the other infant(s) is uninfected, the maternal triple ARV prophylaxis regimen should be continued until cessation of breastfeeding of her uninfected infant(s) or 18 months, whichever comes first.

Mothers of infants with confirmed HIV infection who were randomized to the maternal triple ARV prophylaxis arm are eligible for randomization into the Maternal Health Component following confirmation of the positive infant HIV test (of all their infants if multiple births), and should continue their triple ARV prophylaxis regimen until randomization into the Maternal Health Component to continue or stop their triple ARV prophylaxis regimen (see Section 5.4). Mothers who are ineligible for the Maternal Health Component or refuse participation will discontinue their triple ARV prophylaxis regimen and continue follow-up as per Appendix IA.

Infants identified as HIV-infected will receive CTX through 52 weeks of age, and then based on WHO guidelines through study completion at 104 weeks of age.

4.72 Management including ARV Management, of Women and Infants in Postpartum Component

Mothers of mother-infant pairs randomized to the maternal triple ARV prophylaxis arm (Step 1 Arm A) will receive the study-supplied ARV regimen for the Postpartum Component of PROMISE, fixed dose combination FTC-TDF (TRV) plus LPV-RTV. Mothers in both Step 1 Arms A and B will follow the schedule of evaluations in Appendix IA. Drugs may be switched for toxicity, or for clinical, immunologic or virologic failure according to the criteria specified below; additional drugs provided for use in this study include fixed dose combination 3TC-ZDV, ZDV, 3TC, TDF, efavirenz (EFV), atazanavir (ATV), and ritonavir (RTV).

Infants of mother-infant pairs randomized to either arm in the Postpartum Component whose mothers require a triple ARV regimen for their own health (enter 1077BP Step 2 or Step 3) should continue their study NVP as long as they meet the protocol-specified criteria (breastfeeding, HIV-uninfected and < 18 months of age).

Infants in both Step 1 study arms (Arms A and B) will be followed according to Appendix IB, regardless of infant study drug treatment status or HIV infection status and regardless of maternal study drug treatment status or subsequent pregnancy. If an infant randomized to the infant prophylaxis arm (Arm B) must permanently discontinue the study NVP due to toxicity/intolerability, he/she will be provided an alternative regimen of 3TC through the study (see Section 4.612). Breastfeeding infants of mothers randomized to Arm A (maternal prophylaxis) should continue or resume daily NVP dosing (as outlined in Section 4.612) through cessation of breastfeeding (unless HIV-infected) if the mother cannot continue a triple ARV regimen due to toxicity, and no alternative triple ARV regimen is available or recommended after consultation with the CMC.

4.721 1077BP STEP 2 (Management of Women who Require Treatment - Appendix IA Mothers)

Women who otherwise meet the eligibility criteria in Section 4.52 will be considered to have reached an indication for triple ARV therapy (HAART) for their own health and will enter Step 2 if they:

- experience clinical progression to an AIDS-defining/WHO Stage 4 illness (see Appendix IV); or
- meet country-specific clinical indication(s) for initiation of ARV treatment; or

- have a confirmed CD4 count below 350 cells/mm^3 or below the country-specific threshold for initiation of treatment, if that threshold is > 350 cells/mm^3; or
- otherwise require ARV treatment as determined in consultation with the CMC.

These women may receive study supplied antiretroviral medications or they may receive triple ARV therapy of their choice from outside the study if the treatment regimen meets the protocol definition of HAART (three or more agents from two or more classes of antiretroviral drugs) and is provided by prescription.

NOTE: A participant should not move to a new step if she has a toxicity that, based on the Toxicity Management Guidelines (Appendix II), would necessitate an interruption in therapy; however, the participant may move to the new step once the toxicity has resolved to a grade that would allow resumption of therapy.

<u>1077BP STEP 2 FOLLOW-UP</u>
Women who enter 1077BP Step 2 will follow the schedule of evaluations in Appendix IA, and infants will continue to follow the schedule of evaluations in Appendix IB.

4.722 1077BP STEP 3 (Women Receiving a Triple ARV Regimen in Step 1 Arm A or Step 2 who Require a Change in their Regimen - Appendix IA Mothers)

Women receiving a triple ARV regimen either through Step 1 randomization to Arm A (maternal triple ARV prophylaxis) or through 1077BP Step 2 as therapy for their own health (see Section 4.52) will have virologic as well as clinical and CD4 monitoring. Women with clinical, immunologic or virologic failure or toxicity as defined below are eligible for the 1077BP Step 3 change in regimen. Women with clinical, immunologic or virologic failure or toxicity as defined below will be registered to the 1077BP Step 3 change in regimen.

The criteria for entering 1077BP Step 3 are:

- Clinical failure defined as development of an AIDS-defining/WHO Stage 4 condition; OR
- Immunologic failure defined as a confirmed decrease in CD4 count to less than any of the following:
  - pre-ARV initiation level (i.e., the baseline CD4 count at study entry), or
  - 50% of the participants' peak levels, or
  - 350 cells/mm^3 or below the country-specific threshold for initiation of treatment, if that threshold is > 350 cells/mm^3; OR
- Virologic failure defined as confirmed RNA level > 1,000 copies/mL at or after 24 weeks on a triple ARV regimen; (see note below for counting weeks on a triple ARV regimen); OR
- Significant toxicity requiring a change in the backbone of the regimen, or otherwise requiring a change in more than one class of study drug IF the CMC is consulted and approves the step change *in advance*; OR
- Meets country-specific standard indications for a complete change in regimen; OR
- Otherwise requires a change to an alternate triple ARV regimen as determined in consultation with the CMC.

NOTE: If a participant experiences one of the above conditions but the condition is judged by the study clinician as due to non-adherence, systemic illness, or other explanatory circumstance, such that a change of regimen is not indicated, with approval from the CMC, entry into Step 3 is not required.

NOTE: For purposes of defining virologic failure, the 24 weeks referenced above refers to the number of continuous weeks on a triple ARV regimen and includes time on a triple ARV regimen during the antepartum component even if a different triple ARV regimen was taken in the antepartum component. Please consult the CMC with any questions related to counting weeks on a triple ARV regimen and/or other aspects of defining failure.

While 1077BP Step 3 triple ARV regimens are not defined by this protocol, additional drugs available through the study are specified above. 1077BP Step 3 regimens should be determined at the discretion of the study clinicians (consultation with the CMC available but not required). An ARV regimen that is not provided by the study may be used if it meets the study definition of a triple ARV regimen (three or more agents from two or more classes of ARVs) and is provided by prescription.

NOTE: A participant should not move to a new step if she has a toxicity that, based on the Toxicity Management Guidelines (Appendix II), would necessitate an interruption in the study drug regimen; however, the participant may move to the new step once the toxicity has resolved to a grade that would allow resumption of dosing.

1077BP STEP 3 FOLLOW-UP
Women will follow the schedule of evaluations in Appendix IA, and infants will continue to follow the schedule of evaluations in Appendix IB.

4.723 Women Who Develop Tuberculosis (TB)

Participants who develop TB and are not receiving a triple ARV treatment regimen should enter Step 2 or 3 as applicable and initiate ARV treatment for their own health.

Participants randomized to continue the triple ARV regimen who develop TB and need Rifampin-containing TB treatment while on study may be offered Efavirenz (dose to be determined by site clinician) in place of LPV-RTV if they can use appropriate contraception (as outlined below). All participants on TB treatment may continue to receive TDF and FTC or FTC-TDF (TRV) or 3TC-ZDV (Combivir). These study drug changes will be made available for the duration of the Rifampin-based TB treatment, and for 30 days after stopping Rifampin. Thereafter, the participant will return to her assigned study drug regimen.

NOTE: Participants who are participating in sexual activity that could lead to pregnancy and who are receiving EFV must agree to use two reliable methods of contraception, including a reliable barrier method of contraception together with another reliable form of contraception while receiving EFV and for 12 weeks after stopping EFV. These participants will have pregnancy testing at each study visit while receiving EFV and for 12 weeks after stopping EFV.

4.724 Virologic Monitoring of Women Receiving a Triple ARV Regimen

Virologic failure is not an endpoint in this trial; however, monitoring viral load is used among individuals receiving the study triple ARV regimen for their own health to maximize the benefits of the triple ARV regimen and to determine when treatment should be changed. Therefore, virologic monitoring (Appendix IA) will be provided for women on a triple ARV regimen whether for prophylaxis or treatment in 1077BP Step 1 (Arm A), Step 2 or Step 3.

DHHS treatment guidelines state that the goal of ARV therapy is sustained suppression of HIV RNA to < 50 copies/mL (or below detectable limits of the available HIV RNA assay). However, the plasma HIV RNA threshold for switching therapy is not precisely defined.

Women receiving the study triple ARV regimen, who have a plasma HIV RNA level > 1,000 copies/mL at or after 24 weeks of the triple ARV regimen should return (if possible within 4 weeks) for confirmatory plasma HIV RNA. Women with confirmed HIV RNA levels > 1,000 copies/mL at or after 24 weeks of initial or second line therapy are strongly encouraged to modify their triple ARV regimen (1077BP Step 3). Women in whom virologic failure is believed to be due to non-adherence, systemic illness, vaccination, or other circumstances determined by the study clinicians, will *not* be required to switch regimens unless the study clinician advises that the regimen should be changed (consultation with the CMC available but not required). In such cases, the subject should continue scheduled study visits as outlined in Appendix IA.

Study-provided medications will be available to participants who meet 1077BP Step 3 criteria or participants may access ARVs not provided by the study. Regimen choice should meet the protocol definition of a triple ARV regimen. These regimens may include both study-provided ARVs and ARVs from outside the study if necessary.

In the event that a participant has reached a confirmed HIV RNA > 1,000 copies/mL but does not wish to change her assigned regimen due to clinical and immunologic stability, she may remain on her current regimen and continue to be followed on study with clinical and laboratory monitoring (consultation with the CMC available but not required). If the participant's CD4 cell count falls or the HIV RNA rises, she will be strongly advised to change regimens.

Women randomized to Step 1 Arm A (maternal triple ARV prophylaxis) who enter 1077BP Step 3 for clinical or immunologic failure are not eligible for randomization into the Maternal Health Component but should continue the triple ARV regimen because the criteria were met for regimen failure. Women randomized to Step 1 Arm A who enter 1077BP Step 3 because they needed to change medications due to toxicity are eligible for randomization into the Maternal Health Component.

Women who develop virologic failure on the study triple ARV regimen and move to Step 3 are still eligible for randomization to the Maternal Health Component, as long as they do not have a clinical or immunologic indication to continue their triple ARV regimen. Women who have viral load > 1,000 and who report recent non-adherence or who have been off of their triple ARV regimen for toxicity and resumed are still eligible for enrollment into the Maternal Health Component as long as they meet all of the other eligibility criteria. Questions regarding the eligibility of women with virologic failure should be addressed to the CMC.

4.725 Management of Second-Line ARV Therapy Failure

Participants who experience a confirmed HIV RNA > 1,000 copies/mL on the second-line triple ARV regimen in 1077BP Step 3 or subsequent lines should be managed according to current standard of care and may continue to receive study-provided ARV medications at the discretion of the local investigators, participant and primary care provider. Second-line failure due to non-adherence or intolerance may be able to be managed with use of the study-provided medications, and decisions will need to be made on a case by case basis. If the participant has never had a CD4 cell count < 350 cells/mm^3, the CMC should be consulted and consideration may be given to careful observation off the triple ARV regimen. Women who discontinue the triple ARV

regimen will be followed on study/off study drugs at regular study visits as per their schedule of evaluations when the regimen is stopped.

4.726 Management of HIV/HBV Co-Infected Women Who Received Triple ARV Prophylaxis in the Postpartum Component

HIV/HBV co-infected women who discontinue their triple ARV regimen may be at risk of rebound HBV viremia and subsequent transaminitis. In the Staccato HIV Treatment Interruption Trial, 5/6 HIV/HBV co-infected patients who stopped their triple ARV regimen developed HBV viremia and transaminitis and 1/6 had a severe hepatic flare (30). Additional case reports have also documented transaminitis after HBV-active ART cessation, one resulting in severe hepatitis (31, 32). HIV/HBV co-infected women who discontinue triple ARV prophylaxis as part of the Postpartum Component (e.g., are randomized to the infant NVP arm of the Postpartum Component) will have transaminases measured in real-time at 6 and 14 weeks and have plasma stored and tested retrospectively for HBV DNA, HBeAg and HBeAb at 6 and 26 weeks following ARV discontinuation. If, after triple ARV regimen cessation, liver function tests (transaminases or total bilirubin) are Grade 3 or above or if women are symptomatic (e.g., jaundice, severe fatigue), she should have careful clinical evaluation and her management should be discussed with CMC.

4.727 Women Who Become Pregnant on Study

Women who become pregnant again during follow-up will be maintained in study follow-up, and outcomes will be analyzed based on their initial study randomizations. Women who are receiving a triple ARV regimen as part of the study when they become pregnant will continue to receive their study drugs with modification of the specific regimen as needed; they will also be required to provide separate consent to continue taking the study drugs while pregnant if study-supplied (Appendix V). Women who continue taking LPV-RTV will have a dose increase in the third trimester. Women who are not on a study triple ARV regimen when they become pregnant will be treated according to local standard of care.

Pregnancy outcomes should be ascertained and recorded on study CRFs. For participants who are pregnant at the end of the study or participants who are pregnant and decide to discontinue study participation while pregnant, additional post-study contacts should be completed to ascertain pregnancy outcomes. Outcomes may be ascertained based on participant report but medical records should be obtained whenever possible to supplement participant reports.

Sites are also encouraged to prospectively register pregnant subjects in the Antiretroviral Pregnancy Registry by calling the following number in the US: 910-679-1598 or by faxing: 910-256-0637 or by calling the following number in the United Kingdom: + 44-1628-789-666.

4.73 <u>Concomitant Medication Guidelines</u>

All medications/preparations received by participants (both mothers and infants) during the period of study participation must be documented in the participant's source file, as this information may be needed for assessment of toxicities and adverse events.

- For infants, all medications/preparations (prescription and non-prescription) including alternative, complementary medications/preparations (i.e., traditional medicines) will also be recorded on applicable case report forms for entry into the study database.
- For mothers, all cardiac, diabetic, hepatic, renal drugs, oral antibiotics, OI medications and contraceptives (prescription and non-prescription), all other prescription medications and alternative,

complementary medications/preparations (i.e., traditional medicines) will also be recorded on applicable case report forms for entry into the study database.
- For both mothers and infants, the names of alternative, complementary medications/preparations are not required – only whether or not such substances have been used since the last visit.

Sites must refer to the most recent study medication's package insert or investigator's brochure to access additional current information on prohibited and precautionary medications. To avoid drug interaction and adverse events, the manufacturer's package inserts of the ARV and concomitant agent should always be referred to whenever a concomitant medication is initiated or dose changed.

Concomitant use of ingested traditional medicines is strongly discouraged while participants are on study.

Information on drugs without trade names, with many marketed forms, or those not available in the US may be found at:

http://www.hiv-druginteractions.org/
http://www.nccc.ucsf.edu/

4.731 Prohibited Medications

A participant who requires any medications considered prohibited while on a study drug must have the study drug held or permanently discontinued. A list of medications that are prohibited with study-supplied drugs will be included on the PSWP of the IMPAACT website.

4.732 Precautionary Medications

A list of medications that should be used with caution while on study-supplied drugs will be included on the PSWP of the IMPAACT website.

4.74 Toxicity Management and Adverse Event Reporting (Postpartum Component)

- Toxicity management is described in Appendix II.
- The DAIDS Table for Grading the Severity of Adult and Pediatric Adverse Events, Version 1.0, dated December 2004 (with Clarification dated August 2009) (which is available at the following website: http://rsc.tech-res.com), must be followed with the exception of maxillary-measured fever and malnutrition/failure-to-thrive in infants, for which supplementary grading scales are included in Section 7.2.
- Case Report Form (CRF) recording requirements are included in Section 7.1.
- Requirements for expedited reporting of serious adverse events (SAEs) are included in Section 7.2.

4.75 Criteria for Study Drug Discontinuation

Women or infants may be discontinued from study treatment temporarily or permanently primarily based on toxicity events and tolerability issues. The DAIDS Table for Grading the Severity of Adult and Pediatric Adverse Events, Version 1.0, dated December 2004 (with Clarification dated August 2009) and the Toxicity Management Guidelines (Appendix II) will be used to guide these decisions, in consultation with the CMC when required and/or when desired by the site investigator). Women and infants who are removed from treatment will be continued to be followed on study (off study drug/on study) and follow the relevant maternal or infant schedule of evaluations. The randomized treatment regimen is given through BF cessation or through 18 months postpartum, whichever comes first**,** unless stopped for toxicity, other medical reasons or confirmed infant HIV infection (of all the woman's infants).

Study drugs may be discontinued for any of the following reasons:

- Drug-related toxicity (see Toxicity Management - Appendix II)
- Requirement for prohibited concomitant medications
- Clinical reasons believed life threatening by the site investigator, even if not addressed in the Toxicity Management Guidelines (Appendix II)
- Second virologic failure with CD4 $\geq$ 350 cells/mm^3
- Request of the primary care provider if s/he thinks the study treatment is no longer in the best interest of the participant
- Request of the participant
- Documented HIV infection in the infant (if randomized to maternal triple ARV prophylaxis during breastfeeding, mother may remain eligible for randomization into Maternal Health Component, see Section 5.3)*

Any dispensed study drug remaining after discontinuation must be collected.

* Note: In the case of a multiple birth (e.g., twins), a mother assigned to the maternal the triple ARV prophylaxis arm will not discontinue her study treatment for the criterion of confirmed HIV infant infection if at least one of her study infants remains uninfected; if infants were assigned to infant NVP arm, an infant with confirmed HIV infection must stop study NVP, but if the other infant(s) is uninfected, he/she can continue the study NVP. If infection of an infant cannot be ruled out following an initial positive HIV test, study drug should be permanently discontinued.

Note: Early discontinuation of study product for any reason is <u>not</u> a reason for withdrawal from the study.

4.76 <u>Criteria for Discontinuation of Study Participation</u>

Participants will be withdrawn from study participations for the following reasons:

- Request by the participant to withdraw.
- Request of the primary care provider if s/he thinks the study is no longer in the best
- interest of the participant, after consultation with the CMC.
- Participant judged by the investigator to be a significant risk of failing to comply with the provisions of the protocol as to cause harm to self or seriously interfere with the validity of the study results, after consultation with the CMC.
- At the discretion of the IMPAACT leadership, NIAID, NICHD, the Office for Human Research Protections (OHRP), the US FDA, the pharmaceutical supplier(s), an in-country national health or regulatory agency, or the IRB/EC
- Imprisonment or involuntary confinement in a medical facility (e.g., for psychiatric illness or infectious disease) for a duration that interferes with timely completion of scheduled study visits

4.8 Clinical Pharmacology Plan (Postpartum Component)

4.81 <u>Rationale</u>

Whenever any drug is used in a lactating woman, a major consideration has to be the kinetics of drug transfer into the infant via breast milk. Previous studies with ZDV, NVP, nelfinavir and 3TC have shown different patterns of transfer for each drug. Transfer of ZDV and nelfinavir from mother to infant is negligible (33). In contrast, transfer of 3TC and NVP from mother to infant via breast milk results in clinically significant but subtherapeutic concentrations (34). These concentrations are too low to result in complete viral suppression, making them likely to lead to the development of NVP and 3TC viral

resistance in infected infants (16). Preliminary studies using hair measures in infants born of HIV-infected mothers have demonstrated significant transfer of LPV and RTV during pregnancy, but negligible transfer of both of these agents during breastfeeding (35). Many of the women enrolled in the PROMISE study will be receiving LVP-RTV, TDF and FTC for which there are limited or no data describing mother to child breast milk drug transfer. Samples will be collected from these women and their infants in order to determine the concentration of these ARVs in maternal plasma, breast milk and hair and infant plasma and hair in order to model the kinetics of transfer of these ARVs from mother to infant via breast milk.

4.82 Pharmacology Objectives

4.821 To estimate maternal pharmacokinetic parameters and drug exposure for LPV-RTV, TDF, and FTC (and other second line ARVs that women may receive) in breastfeeding mothers receiving a triple ARV regimen.
4.822 To characterize the kinetics of ARV transfer from maternal plasma to breast milk in these women and estimate infant breast milk drug dose.
4.823 To determine infant ARV exposure from breast milk during maternal receipt of LPV-RTV, TDF, and FTC (and other second-line ARVs that may be received in women) therapy using the estimates of infant breast milk drug dose from Objective 2, measured infant drug concentrations and existing population models of the pharmacokinetics of these drugs in infants, where available.
4.824 To assess the relationship between hair drug concentrations and plasma ARV drug concentrations in selected mother-infant pairs during breastfeeding.

4.83 Study Design, Modeling and Data Analysis

4.831 Pharmacology Study Design

Samples of maternal plasma, hair, breast milk, and infant blood (plasma or dried blood spot) will be collected at birth, weeks 1, 6, 14, 26, and subsequent visits during breastfeeding. Sets of samples from 50 mother-infant pairs receiving each drug of interest and will be assayed for ARV concentrations. Assays for individual ARVs in plasma, breast milk and dried blood spots will be assigned to one of the IMPAACT Pharmacology Laboratories, depending on laboratory expertise and work load. Demographic and clinical data will be compiled from the PROMISE data base. Data describing the timing of recent ARV doses will be collected. Assays for ARVs in hair samples will be performed at the University of California San Francisco (UCSF) Drug Studies Unit.

4.832 Modeling and Data Analysis

Objective 1: *To estimate maternal pharmacokinetic parameters and drug exposure for LPV-RTV, TDF, and FTC (and other second-line ARVs that women may receive) in breastfeeding mothers receiving a triple ARV regimen.* A two-stage approach will be used. First, descriptive pharmacologic analyses will be performed for each drug by generating summed maternal concentration time plots for the entire population. These plots will be compared graphically with existing data describing the distribution of drug concentrations over time in non-pregnant adults, using reference ranges derived from postpartum pharmacokinetic results from P1026s. Monte Carlo simulations will be used to construct confidence interval bands for expected concentrations encompassing the following intervals: $<10^{th}$, 10th -$<25^{th}$, 25th-$<50^{th}$, 50th-$<75^{th}$, 75th -$<90^{th}$ and $>90^{th}$ percentiles. The measured maternal plasma drug concentrations in PROMISE will be compared to those postpartum from P1026s values and the ratio to the non-BF maternal concentration calculated. The percentile band will be determined for each PROMISE maternal pharmacokinetic sample for each drug. These comparisons will provide an initial indication of whether differences exist in ARV exposure between the breastfeeding mothers and the reference populations. The

second stage of the analysis will use pharmacokinetic modeling techniques to provide estimates of pharmacokinetic parameters and drug exposure for the breastfeeding women and to make statistical comparisons between the breastfeeding women and the reference population. Among the modeling techniques used will be least squares mixed effects modeling. In this approach, pharmacokinetic parameters describing antiretroviral kinetics in maternal blood, including intra- and inter-patient variability, will be estimated using nonlinear hierarchical models and an extended least squares method using the software program NONMEM. The data will be nested with the maternal pharmacokinetic data from P1026s.

Objective 2: *To characterize the kinetics of transfer of LPV-RTV, TDF, and FTC (and other second-line ARVs that may be received in women) from maternal plasma to breast milk in these women and estimate infant breast milk drug dose.* A similar two-stage approach will be used to analyze the breast milk data. First, descriptive pharmacologic analyses will be performed for each drug comparing absolute breast milk concentrations and milk to plasma ratios to temporal and demographic variables, including time from maternal dosing, time from last feeding and time since delivery. The second stage will use pharmacokinetic parameters determined in Objective 1 and a similar modeling approach to the breast milk concentration data. The kinetics of drug transfer from plasma to breast milk and an estimated drug dose delivered to the infant in breast milk will be modeled. The model will incorporate breast milk as a pharmacokinetic compartment and model the plasma and breast milk drug concentration concomitantly using a semi-physiologic approach. Other components of the PROMISE study will involve analyzing the breast milk and maternal blood samples for HIV viral load and resistance profiles. We will perform exploratory analyses looking for relationships between these virologic parameters (breast milk viral load, resistance profile of breast milk virus and discrepancies between breast milk and blood resistance profiles) and the ARV exposure parameters. While the sample size will probably be too small for definitive conclusions, we hope to be able to develop preliminary hypotheses relating breast milk viral load and resistance to breast milk drug exposure.

Objective 3: *To estimate infant antiretroviral exposure from breast milk during maternal LPV-RTV, TDF and FTC (and other second-line ARVs that may be received in women) dosing using the estimates of infant breast milk drug dose from Objective 2, measured infant plasma drug concentrations and existing models of these ARV pharmacokinetics in infants.* Infant ARV exposure will be estimated using modeling techniques. The breast milk drug doses described above and an assumed consumption of 150 mL/kg/day of breast milk will initially be used for infant drug input (36). The infant drug exposure will be modeled as a function of maternal plasma pharmacokinetics, time of maternal dosing, timing of infant feedings and maturational state of the infant. Analyses performed for Objectives 1 and 2 will provide will the maternal information necessary for the assessment of infant pharmacokinetics in drugs where appreciable concentrations are found. Models of maternal plasma and breast milk kinetics will be linked and modeled simultaneously with the infant PK models. The infant models may be nested with existing developmental models of drug disposition where data exist or from more general models of drug metabolizing enzyme ontogeny (37). Simultaneous modeling should provide a more accurate description of the pharmacokinetic parameter space since mothers with higher drug concentrations are likely to give their infants larger drug doses through breast milk. The final models will be used to estimate the range of drug exposures likely to be seen in BF infants over the entire period of breastfeeding. Monte Carlo simulations will be performed in two thousand individuals at each visit age using the Simulation subroutine of NONMEM to provide the infant drug exposure pattern expected during breastfeeding.

Objective 4: *To assess the relationship between hair drug concentrations and plasma ARV drug concentrations in selected mother-infant pairs at the end of the study.* As above, fifty mother-infant pairs in each study arm will be enrolled into the pharmacology sub-study and samples of maternal plasma, hair, breast milk, infant blood (plasma or dried blood spot) and hair will be collected at birth and subsequent visits during breastfeeding. The ratios of the infant hair concentrations to maternal hair concentrations at

delivery will help determine the kinetics of drug transfer from mother to infant *in utero*. The ratios of infant to maternal concentrations during breastfeeding in both plasma and hair will help determine the kinetics of ARV transfer during this period. The correlation between hair ARV measures and concentrations of ARVs in plasma and breast milk will be assessed via scatterplots. Correlations between maternal and infant hair LPV/RTV concentrations and maternal and infant PK parameters from the modeling described above will be investigated and expansion of the PK models to include drug transfer into maternal and infant hair will be investigated.

4.9 References – Postpartum Component

(1) WHO Collaborative Study Team on the Role of Breastfeeding on the Prevention of Infant Mortality. Effect of breastfeeding on infant and child mortality due to infectious diseases in less developed countries: a pooled analysis. Lancet 2000; 355:451-5.

(2) The Breastfeeding and HIV International Transmission Study Group. Late postnatal transmission of HIV in breast-fed children: an independent patient data meta-analysis. J Infect Dis 2004; 189:2154-66.

(3) World Health Organization. Guidelines on HIV and infant feeding 2010 – principles and recommendations for infant feeding in the context of HIV and a summary of the evidence. 2010 Geneva, Switzerland. URL http://www.who.int/maternal_child_adolescent/documents/9789241599535/ en/index.html.

(4) Kuhn L, Aldrovandi GM, Sinkala M, et al for the Zambia Exclusive Breastfeeding Study. Effects of early, abrupt weaning on HIV-free survival of children in Zambia. N Engl J Med. 2008 Jul 10;359(2):130-41.

(5) Thior I, Lockman S, Smeaton LM, et al. Breastfeeding plus infant zidovudine prophylaxis for 6 months vs. formula feeding plus infant zidovudine for 1 month to reduce mother-to-child HIV transmission in Botswana: a randomized trial: tMashi Study. JAMA 2006 Aug 16; 296(7):794-805.

(6) Shetty AK, Coovadia HM, Mirochnick MM, et al for the HIVNET 023 Study Team. Safety and trough concentrations of nevirapine prophylaxis given daily, twice weekly, or weekly in breastfeeding infants from birth to 6 months. JAIDS 2003; 34:482-90.

(7) Six Week Extended-Dose Nevirapine (SWEN) Study Team. Extended-dose nevirapine to 6 weeks of age for infants to prevent HIV transmission via breastfeeding in Ethiopia, India, and Uganda: an analysis of three randomised controlled trials. Lancet 2008;372 (9635):300-12.

(8) Kumwenda NI, Hoover DR, Mofenson LM, et al. Extended antiretroviral prophylaxis to reduce breast-milk HIV transmission. N Engl J Med 2008;359(2):119-29.

(9) Vyankandondera J, Luchters S, Hassink E, et al. Reducing risk of HIV transmission from mother to infant through breastfeeding using antiretroviral prophylaxis in infants (SIMBA). 2nd IAS Conference on HIV Pathogenesis and Treatment, Paris, France, 2003; Abstract LB7.

(10) Kilewo C, Karlsson K, Massawe A, et al. Prevention of mother-to-child transmission of HIV-1 through breast-feeding by treating infants prophylactically with lamivudine in Dar es Salaam, Tanzania: the Mitra Study. JAIDS 2008;48 (3):237-40.

(11) Coovadia HM, Brown ER, Fowler MG, et al. Efficacy and safety of an extended nevirapine regimen in infant children of breastfeeding mothers with HIV-1 infection for prevention of postnatal HIV-1 transmission (HPTN 046): a randomized, double-blind, placebo-controlled trial. Lancet 2012;379:221-8.

(12) Thomas T, Masaba R, Borkowf CB, et al. Triple-antiretroviral prophylaxis to prevent mother-to-child HIV transmission through breastfeeding--the Kisumu Breastfeeding Study, Kenya: a clinical trial. PLosMed 2011;8 (3):e1001015.

(13) Kilewo C, Karlsson K, Ngarina M, et al. Prevention of mother to child transmission of HIV-1 through breastfeeding by treating mothers with triple antiretroviral therapy in Dar es Salaam, Tanzania – the MITRA-PLUS study. JAIDS 2009;52:406-16.

(14) Peltier CA, Ndayisaba GF, Lepage P, et al. Breastfeeding with maternal antiretroviral therapy or formula feeding to prevent HIV postnatal mother to child transmission in Rwanda. AIDS 2009;23:2415-23.

(15) Palombi L, Marazzi MC, Voetberg A, et al. Treatment acceleration and the experience of the DREAM program in PMTCT of HIV. AIDS 2007;21 Suppl 4:S65-71.

(16) Zeh C, Weidle P, Nafisa L, et al. HIV-1 drug resistance emergence among breastfeeding infants born to HIV-infected mothers during a single-arm trial of triple antiretroviral prophylaxis for prevention of mother to child transmission: a secondary analysis. PLosMed 2011;8 (3):e1000430.

(17) Chasela CS, Hudgens MG, Jamieson DJ et al. Maternal or infant antiretroviral drugs to reduce HIV-1 transmission. N Engl J Med 2010;362:2271-81.

(18) Shapiro RL, Hughes MD, Ogwu A, et al. Antiretroviral regimens in pregnancy and breastfeeding in Botswana. N Engl J Med 2010;362:2282-94.

(19) Kesho Bora Study Group. Triple antiretroviral compared with zidovudine and single-dose nevirapine prophylaxis during pregnancy and breastfeeding for prevention of mother-to-child transmission of HIV-1 (Kesho Bora study): a randomised controlled trial. Lancet Infect Dis 2011;11:171-80.

(20) Mirochnick M, Capparelli E, Nielsen K, Pilotto JH, Musoke P, Shetty A, Luzuriaga K. Nevirapine pharmacokinetics during the first year of life: A population analysis across studies. Pediatric Academic Societies Meeting, San Francisco, CA, May, 2006.

(21) Church JD, Omer SB, Guay LA, et al. Analysis of Nevirapine (NVP) Resistance in Ugandan Infants Who Were HIV Infected Despite Receiving Single-Dose (SD) NVP versus SD NVP Plus Daily NVP Up to 6 Weeks of Age to Prevent HIV Vertical Transmission. J Infect Dis 2008; 198 (7):1075-1082.

(22) Moorthy A, Gupta A, Bhosale R, et al. Nevirapine resistance and breast milk HIV transmission: effects of single- and extended-dose nevirapine prophylaxis in subtype C HIV-infected infants. PLosOne 2009;4 91):e4096.

(23) Report of the WHO Technical Reference Group, Paediatric HIV/ART Care Guideline Group meeting. WHO Headquarters, Geneva, Switzerland. 10-11 April 2008.

(24) Panel on Antiretroviral Guidelines for Adult and Adolescents. Guidelines for the use of antiretroviral agents in HIV-infected adults and adolescents. Department of Health and Human Services. March 27, 2012. URL:http://www.aidsinfo.nih.gov/guidelines/html/1/adult-and-adolescent-treatment-guidelines/0/.

(25) Gallant JE, DeJesus E, Arribas JR, et al. Tenofovir DF, emtricitabine, and efavirenz vs zidovudine, lamivudine and efavirenz for HIV. N Engl J Med 2006;354 (3):251-60.

(26) Johnson MA, Gathe JC Jr, Podzamczer D, et al. A once daily lopinavir/ritonavir-based regimen provides noninferior antiviral activity compared with a twice-daily regimen. JAIDS 2006; 43 (2):153-60.

(27) Nuesch R, Anaworanich J, Srasuebkul P, et al. Interruptions of tenofovir/emtricitabine-based antiretroviral therapy in patients with HIV/hepatitis B virus co-infection. AIDS 2008; 22 (1):152-4.

(28) Nielsen-Saines K, Mirochnick M, Boechat MI, et al. Bone Age and Mineral Density Assessments Using Plain Roentgenograms in Tenofovir (TDF) exposed infants in Malawi and Brazil Enrolled in HPTN 057. Pediatric Academic Societies Meeting, Vancouver, Canada, May, 2010.

(29) World Health Organization. Antiretroviral therapy for HIV infection in adults and adolescents – recommendations for a public health approach. 2010 revision. 2010 Geneva, Switzerland. URL: http://www.who.int/hiv/pub/arv/adult2010/en/index.html.

(30) WHO/UNICEF/UNAIDS. HIV and infant feeding counseling: a training course. Trainers’ Guide. World Health Organization, Geneva; 2000.

(31) Sellier P, Clevenbergh P, Mazeron MC, et al. Fatal interruption of a 3TC-containing regimen in a HIV-infected patient due to re-activation of chronic hepatitis B virus infection. Scand J Infect Dis 2004;36(6-7):533-5.

(32) Neau D, Schvoerer E, Robert D, et al. Hepatitis B exacerbation with a precore mutant virus following withdrawal of LMV in a human immunodeficiency virus-infected patient. J Infect 2000; 41(2):192-4.

(33) Corbett A KA, Rezk N, Jamieson D, et al., BAN Study Team. Antiretroviral drug concentrations in breast milk and breastfeeding infants. 15th CROI, Boston, MA, February 2008; Abstract 648.

(34) Mirochnick M, Thomas T, Capparelli E, et al. Antiretroviral concentrations in breastfeeding infants of mothers receiving highly active antiretroviral therapy. Antimicrob Agents Chemother 2009 ;53 :1170-6.

(35) Achan J Cohan D, Aweeka F, Mwesigwa J, Huang Y, Plenty A, Kamya M, Havlir DV, Gandhi M. Lopinavir and Efavirenz Concentrations in Hair Samples as a Marker of Cumulative Exposure among Postpartum Women and Breastfeeding Infants in Uganda; O-14; 4th International Workshop on HIV and Pediatrics, Washington DC, July 2012.
(36) Kent JC, Mitoulas L, Cox DB, et al. Breast volume and milk production during extended lactation in women. Exp Physiol 1999;84(2):435-44.
(37) Edginton AN, Schmitt W, Voith B, Willmann S. A mechanistic approach for the scaling of clearance in children. Clin Pharmacokinet 2006;45(7):683-704.

4.10 Sample Informed Consent Form – Postpartum Component

Informed Consent Form – Postpartum Component
IMPAACT 1077BF
Breastfeeding Version of the PROMISE Study
(Promoting Maternal and Infant Survival Everywhere)
Protocol Version 3.0, Dated 15 October 2012

Note: Version number and date of the protocol should be included on the first page of the consent form and the version number and date of the consent form should be included in a header or footer on each page of the consent form along with page numbering in the following format: Page 1 of x, Page 2 of x, Page 3 of x. Sites may omit the tables and diagrams if not appropriate; however the information contained therein must be conveyed in the text.

INTRODUCTION

You and your baby are being asked to take part in this research study because:
- you are infected with human immunodeficiency virus (HIV), the virus that causes AIDS
- you are pregnant or have just had a baby
- you are going to breastfeed

This study is sponsored by the US National Institutes of Health (NIH). The doctor in charge of this study at this site is: *[insert name of site Principal Investigator]*. Before you decide if you want you to join this study with your baby, we want you to know about the study. We will explain the study to you and you are free to ask questions at any time. We will ask if you want to join the study with your baby as a pair. If you do want to join with your baby, we will ask you to sign this consent form or make your mark (in front of a witness, if needed). You will be offered a copy to keep.

WHY IS THE PROMISE STUDY BEING DONE?

The PROMISE Study has been designed to look for the best ways to prevent the transmission of HIV from a mother to her baby during pregnancy, labor and delivery and breastfeeding and ways to make sure that both the HIV-infected mother and HIV-exposed baby stay as healthy as possible from birth and beyond weaning. The PROMISE study has three goals and is divided into three parts to reach those goals.

PROMISE Goals	Parts
Goal 1:<br>To determine the best combination of anti-HIV medications to give to HIV-infected pregnant women to prevent HIV infection in babies during pregnancy or at time of delivery.	Antepartum
Goal 2:<br>To determine the best way to protect the baby from HIV while being breastfed.	Postpartum
Goal 3:<br>To find the best way to take care of the mother's health during and after pregnancy.	Maternal Health

This is a consent form to join the Postpartum Part of the PROMISE Study. We will give you detailed information about the Maternal Health Part at a later time, but you are free to ask questions now if you would like.

WHY IS THE POSTPARTUM PART BEING DONE?

The goal of this part of the PROMISE Study is to compare the safety and effectiveness of different medicines given to protect a baby from getting HIV while being breastfed by an HIV-infected mother.

Without any anti-HIV medicines, about one in three babies born to HIV-infected mothers will become infected. Some of these infections occur during the delivery of the baby and some of these infections can occur while the baby is breastfeeding.

Studies have been done that suggest giving the baby an anti-HIV medicine for 6 weeks, 14 weeks or up to 6 months of breastfeeding may help decrease the chance of the baby getting HIV from the breast milk. Other studies have suggested that giving the mother a combination of anti-HIV drugs during the first 6 months while she is breastfeeding is another way to decrease the chance of her baby from getting HIV through the breast milk.

The clinical staff will describe the country-specific standard of care to prevent transmission of HIV from a mother to her baby during breastfeeding and how this care is different than what you may receive in this part of the study.

We do not know which way is better long term: to give the medication to the baby throughout breastfeeding or to give the medications to the mother throughout breastfeeding (with medication to the baby for the first six weeks of life). We want to compare these two methods in preventing HIV infection during breastfeeding – giving anti-HIV drugs to the baby or giving anti-HIV drugs to the mother

**Baby Study Drug Group**		**Mother Study Drug Group**
The anti-HIV medication is given to the baby throughout the time of breastfeeding or for 18 months after birth, whichever comes first	OR	The anti-HIV medications are given to the mother throughout the time of breastfeeding or for 18 months after delivery, whichever comes first with anti-HIV medication given to the baby through 6 weeks of age

The PROMISE Study and all of its parts have been approved by the *[sites: add local ethics committee or IRB information]*. Institutional Review Boards (IRBs) and Ethics Committees are special groups that watch over the safety and rights of research participants in their community.

WHAT WILL MY BABY AND I HAVE TO DO IF WE JOIN THIS STUDY?

<u>Screening/First Visit</u>

If you decide that you want to join the Postpartum Part with your baby, we will need to confirm that you and your baby are eligible. If you or your baby is not eligible for the Postpartum Part, we will talk about whether you may be eligible for the Maternal Health Part of the PROMISE Study.

All or most of the laboratory tests and other assessments needed to determine if you and your baby are eligible for the Postpartum Part were done through your participation in the Antepartum Part or Late Presenter Registration part of the PROMISE Study. Depending on the results and/or when the tests were done, we may need to repeat some of those tests. Any other necessary screening tests will be done before you and your infant can be enrolled in the Postpartum Part of the study.

The first Postpartum Part study visit happens 6 to 14 days after your baby is born. You and your baby will be randomly assigned *[sites—insert locally relevant description here, such as, “like flipping a coin”]* to one of the study groups; either the Baby Study Drug Group or the Mother Study Drug Group. You and the study staff will know which group you are in.

We will ask how you and your baby are doing, about any medications you or your baby are taking and about how well you and your baby are taking the study drugs, if still on them. The specific tests and procedures to be done at this visit are described in the next section.

Study Visits

Until your baby is about 6 months of age, the study visits will be one time each month. After that, the study visits will change to 1 time every 3 months for you and your baby. Each study visit will last about *[sites—if required by your IRB, insert local information on time required for study visits]*. You and your baby will have routine medical check-ups at the study clinic. It is important that you and your baby return for all of these study visits. If you do not come for a study visit or if a test result comes back abnormal, the outreach worker will contact you by home visit or phone call to find out how you and your baby are doing. If at any time, you or your baby becomes sick you should let the study nurse or doctor know right away.

Tests and procedures at the study visits

- Medical history, questionnaire, and physical exam
  We will be ask you about any medications you or your baby have taken in the past and about how well you are taking the study drugs if still on them. We will ask about breastfeeding and any foods or liquids you are feeding your baby. You and your baby will have a physical exam. We will also ask you questions about how you live and work.

- Blood collected
  Blood will be collected from you for various tests. Some tests measure how well study drugs are controlling the virus, and other tests will check on your health. You will have approximately 10 to 32 mL (2-6 tsp) of blood taken at most visits *[sites include local relevant wording]*).

  We will collect about 5 ml *[sites include local relevant wording]* from your baby at each of the visits. If you are Hepatitis B co-infected we will collect an additional 1 – 3 ml from your baby at some visits. At most visits, we will test your baby for HIV and to make sure that the medications are not harming your baby. If your baby becomes infected with HIV, we may need to collect some additional blood (about 1 ml *[sites include local relevant wording]*) at some of these visits.

  You will be given the results of blood tests that might affect your or your baby's health care as soon as possible, usually at the next study visit. Some of the tests will be used to help us know if you and your baby are eligible for one of the next PROMISE parts. Some of your blood and your baby's blood will be tested immediately, and some of the blood may be kept for a while and used later for study-related tests.

- Pregnancy test
  If pregnancy is suspected, you may be asked to give an additional 1mL of blood or a urine sample to test for pregnancy, unless you are taking Efavirenz, in which case, you will be tested for pregnancy at each study visit and for three months thereafter. You will be given the results of the pregnancy test as soon as possible.

- Breast milk
  We will ask you to express up to 20 ml of breast milk (about 4 teaspoons) *[Sites to include relevant wording]*, which will be kept to look for the HIV virus, your body's response to the virus, and the presence of study medications later.

[For sites that are participating in the Hair Substudy:]

- *[Hair*
  [We ask that you allow us to collect about 20 strands of hair from you and/or your infant at each visit while you or your infant are receiving anti-HIV medications and while you are breastfeeding. The hair will be checked to see how much of the anti-HIV medicine is in the hair strands. Medicines go into many places in the body, including the hair. The amount of medicine found inside the hair may provide a better idea of how much medicine could be in the body after a long period of time. This cutting of the small hair sample from you and your baby is voluntary. You will be asked to indicate whether you agree to have hair collected when you sign this consent form. If you do not agree to have you and your baby's hair collected, or you change your mind later, you can still participate in this part of the PROMISE study.]

WHAT HAPPENS IF I DEVELOP AIDS OR MY T-CELLS FALL DURING THE POSTPARTUM PART?

In the event that your disease progresses and you are advised to start treatment for your own health, you will have the option to receive ARV medications from the study for therapy. If you prefer to take locally available HIV medicines that is also an option. We would like you to remain in follow-up on the PROMISE Study while taking medications. You will continue to be followed up until the study is completed.

OTHER INFORMATION

The information collected in this study may be used for other IMPAACT-approved research.

HOW MANY WOMEN AND CHILDREN WILL TAKE PART IN THE PROMISE STUDY?

About 4650 women and their babies will take part in this Postpartum Part of the PROMISE Study around the world.

HOW LONG WILL MY BABY AND I BE ON THE PROMISE STUDY?

You will be in the study from 2 to 5 years, depending on when you join the study. Most women will be in the study for less than 3 years. Your baby will be followed until about 2 years of age.

WHY MIGHT THE DOCTOR TAKE ME OR MY BABY OFF THIS STUDY EARLY?

The study doctor may need to take you or your baby off the study early without your permission if the study is cancelled or stopped.

WHY MIGHT THE DOCTOR HAVE ME OR MY BABY STOP TAKING STUDY MEDICATIONS EARLY?

The study doctor may also need to take you or your baby off the study medications early if:

- you and your baby are not able to attend the study visits
- you or your baby are not able to take the study medications
- continuing the study medications may be harmful to you or to your baby
- you or your baby need a treatment that you may not take while on the study
- your baby is found to have HIV infection
- you request to stop the study medication for you or your baby

If you or your baby has the study medications stopped early for any reason, you will remain in the PROMISE study and return for all of the study visits as scheduled.

AFTER THE PROMISE STUDY

After you and your baby have finished your PROMISE Study participation, the PROMISE Study will not be able to continue to provide you or your baby with the study medications. If continuing to take these or similar medicines would be of benefit to you or your baby, the PROMISE Study staff will discuss how you may be able to obtain them *[sites insert local information here]*.

WHAT ARE THE RISKS OF THE STUDY?

Taking part in this study may involve some risks and discomforts. These include possible side effects of the anti-HIV medicines that you and your baby may take, possible risks and discomforts from the study tests, and possible risks to your privacy. More information is given on each of these types of risks below.

<u>Side Effects of Anti-HIV Medicines for Women</u>

Women in the Postpartum Part of the PROMISE Study will take at least 3 different anti-HIV medicines. Some of the medicines are combined together in one tablet, others come in separate tablets. Until you join the study, we will not know what specific medicines you will take. Therefore, this form gives information about all the anti-HIV medicines women may take. These are:

- Atazanavir (ATV)
- Didanosine (DDI)
- Efavirenz (EFV)
- Emtricitabine (FTC), taken with tenofovir disoproxil fumarate
- Lamivudine (3TC)
- Lopinavir (LPV), taken with ritonavir
- Rilpivirine (RPV)
- Ritonavir (RTV)
- Tenofovir disoproxil fumarate (TDF)
- Zidovudine (ZDV)

Each of the other medicines can cause side effects, when taken alone and when taken in combination. No new or unexpected side effects are observed with drugs combined in one tablet than those observed when each drug is given separately. The combination drugs that may be used in this part of the study include *[sites: insert locally appropriate names of combination drugs – LPV/RTV; 3TC/ZDV; TDF/FTC; and TDF/FTC/RPV – used at your site]*. Some side effects are minor, while others can be severe. Some are common, while others are rare. If you join the study, the study staff will tell you about the side effects of the specific medicines you will take. They will check for side effects during study visits and tell you what to do if you have any side effects.

First, you should know about the possible severe side effects. These effects are rare, but they can cause serious health problems and can result in death:

- Severe rash. This can be caused by atazanavir, efavirenz and lopinavir/ritonavir, ritonavir.

- Abnormal heart beat, which can result in lightheadedness, fainting, and serious heart problems. This can be caused by atazanavir, lopinavir/ritonavir and ritonavir.

- Inflammation of the pancreas. The pancreas is an organ near the stomach. When the pancreas becomes inflamed, it can cause pain in the belly, nausea, vomiting, and increased fats in the blood. This can be caused by didanosine, efavirenz, lamivudine, lopinavir/ritonavir, ritonavir and tenofovir.

- Inflammation of the liver. The liver is an organ near the stomach. When the liver becomes inflamed, it can cause pain and swelling in the belly, nausea, and vomiting. This can be caused by efavirenz, lamivudine, lopinavir/ritonavir, ritonavir, tenofovir and zidovudine.

- Lactic acidosis, enlargement of the liver, and fatty liver, which can result in liver failure. Lactic acidosis is an imbalance in the blood that can cause weight loss, pain in the belly, nausea, vomiting, tiredness, weakness and difficulty breathing. When the liver is enlarged, it can cause pain especially on the right side of the belly, swelling in the belly, nausea, vomiting and loss of appetite. It can also cause bleeding problems that can result in vomiting blood or dark colored stools. Fatty liver is when healthy liver cells are replaced with fat. Sometimes it causes the liver to be enlarged, but doctors usually find out about it from tests of the blood. These effects can be caused by didanosine, emtricitabine, lamivudine, tenofovir and zidovudine. They occur more often in women, pregnant women, people who are overweight, and people who already have liver problems.

- Kidney damage or failure. The kidneys are organs near the middle of your back (one on each side). Doctors usually find out about kidney damage from tests of the blood. These effects can be caused by tenofovir.

- Severe depression, including suicidal thoughts or acts. This can be caused by efavirenz and rilpivirine.

- Other severe mental problems, including aggressive behavior and abnormal thinking. This can be caused by efavirenz.

- Efavirenz might also cause severe harm to unborn babies if taken during the first month of pregnancy.

You should also know about the more common side effects, which are not severe. There are many possible mild and moderate side effects. Some people who take anti-HIV medicines have some of these effects, other people have different effects. The more common mild and moderate side effects are listed below:

**Overall Body Effects**<br>• Overall weakness, tiredness, or feeling unwell<br>• Loss of appetite<br>• Loss of weight<br>• Changes in the placement of body fat, such as enlargement of the neck, stomach, and breasts and thinning of the arms, legs, and cheeks<br>• Numbness or tingling in the hands, arms, feet, legs, or around the mouth<br>• Pain in the hands or feet<br>• Allergic reaction<br>• Fever	**Effects on Your Muscles and Bones**<br>• Aches or pains<br>• Loss of muscle<br>• Muscle weakness<br>• Bone thinning or softening (which could increase the chance of breaking a bone)
**Effects on Your Skin**<br>• Rash, with or without itching<br>• Yellowing of the skin<br>• Darkening of the palms and soles of feet<br>**Effects on Your Head**<br>• Headache<br>• Runny nose<br>• Yellowing of the eyes<br>• Not seeing normally<br>• Changes in the sense of taste<br>• Swelling of the face, lips, or tongue	**Effects on Your Blood**<br>• Decreased blood cells<br>• White blood cells help fight infection.<br>• Red blood cells help store and transport energy through the body. Low red cells can cause weakness, tiredness and dizziness.<br>• Increased bleeding if you have hemophilia<br>• Increased blood sugar or development of diabetes<br>• Increased fats in the blood that may increase the risk of heart problems<br>• Other changes in blood test results that may indicate problems with the muscles, kidneys, liver, pancreas or gall bladder. The blood tests that may be affected include tests of how well these organs are working, tests of substances made by these organs, and tests of fats in the blood.
**Effects on Your Chest**<br>• Cough<br>• Shortness of breath<br>• Heartburn<br>**Effects on Your Belly**<br>• Pain or discomfort in the belly<br>• Nausea<br>• Vomiting<br>• Gas<br>• Loose or watery stools<br>• Inflammation of the gall bladder. The gall bladder is an organ near the stomach. If it becomes inflamed, it can cause severe pain.<br>• Stones in the gall bladder or kidneys. If these stones form, they can cause severe pain.	**Effects on Your Mind or Mental Function**<br>• Drowsiness<br>• Trouble sleeping<br>• Unusual dreams<br>• Difficulty concentrating<br>• Confusion<br>• Depression<br>• Agitation or anxiety<br>• Exaggerated feeling of well being<br>• Hallucinations<br>• Feeling of strangeness or losing touch with reality<br>• Dizziness

The list above is not a complete list of all side effects for all anti-HIV medicines. As a reminder, if you join the study, the study staff will tell you about the side effects of the specific medicines you will take.

Other Possible Risks of Anti-HIV Medicines for Women

Risk of Resistance: All anti-HIV medicines can cause resistance. When resistance occurs, a medicine no longer works against HIV, which can limit the choices of medicines a person can take against HIV in the future. To avoid resistance, it is important to take anti-HIV medicines as instructed, and not miss doses.

Risk of Immune Reconstitution Syndrome: In some people with advanced HIV infection, symptoms from other infections or certain diseases may occur soon after anti-HIV medicines are started. Some of these symptoms may be life threatening. If you start having new symptoms, or notice that existing symptoms are getting worse after starting your anti-HIV medicines, tell your doctor right away.

Risks with Hepatitis B: Some anti-HIV medicines are active against Hepatitis B. For women who have Hepatitis B, and take anti-HIV medicines that are active against Hepatitis B, there are some risks. The Hepatitis B could become resistant and harder to treat. Also, if women later stop taking the medicines that are active against Hepatitis B, this could cause the Hepatitis B to worsen. If this happens, most women get better quickly without treatment, but in rare cases this has resulted in death.

Risks with Contraception: Some anti-HIV medications can interfere with some contraceptive methods, including pills, injections (shots), and implants (placed under the skin). Because of this, it may be necessary to use different or additional contraceptive methods while taking anti-HIV medicines. The study staff will tell you about the effects of the specific anti-HIV medicines you will take and discuss reliable contraceptive methods with you.

Side Effects of Anti-HIV Medicines for Babies

All babies will take the anti-HIV medicine nevirapine for 6 weeks after birth. This is recommended for all babies born to women who have HIV, and the risks of taking nevirapine are the same whether it is given in the study or given outside the study.

Some babies will continue taking nevirapine for up to 18 months while breastfeeding. Some serious side effects have been seen when nevirapine has been taken as treatment by adults and children who have HIV, but no serious side effects have been seen so far in studies of babies taking nevirapine for prevention for several weeks or months after birth. The most common side effects seen in babies have been rash and low red and white blood cells. Babies that cannot take nevirapine while breastfeeding because of side effects will be given another anti-HIV medicine, lamivudine, instead. Some serious side effects have been seen when lamivudine has been taken as treatment by adults and children who have HIV, but no serious side effects have been seen so far in studies of babies taking lamivudine for prevention for several months after birth.

Babies may also receive some anti-HIV medicines taken by their mothers through breast milk. It is not known how much medicine is passed into breast milk, and what effects this may have. For babies that become infected with HIV, it is possible that this may cause resistance (explained above).

The study staff will check for side effects in babies during study visits and tell you what to do if your baby has any side effects.

Other Possible Risks of Anti-HIV Medicines for Babies

If your baby becomes infected with HIV while he or she is taking nevirapine or lamivudine, continuing to take these medicines could cause resistance. When resistance occurs, a medicine no longer works against HIV, which can limit the choices of medicines a person can take against HIV in the future. To avoid resistance, babies that become infected with HIV must stop taking nevirapine or lamivudine as soon as possible after infection. The study staff will test your baby for HIV and will give you the test results as soon as possible. However, it is possible that some days or weeks could pass before finding out your baby is infected. To avoid this, it is important to give updated contact information to the study staff and to attend study visits as scheduled.

Risks of the Study Tests

Blood drawing may cause some pain, bleeding or bruising where the needle enters. There is a small risk of skin infection at the puncture site.

It may be uncomfortable and awkward to express breast milk.

[For sites that are participating in the Hair Substudy: There is a very small chance that you or your infant might be cut during collection of the hair strands with the scissors.*]*

Possible Risks to Your Privacy

We will make every effort to protect your privacy while you are in this study. Your visits here will take place in private. However, it is possible that others may learn of your participation here and, because of this, may treat you unfairly. There also is a risk to your privacy if someone else taking part in this study knows you.

Other Risks

A recent study suggests that taking three anti-HIV medicines can make it much less likely for a person with HIV to pass HIV to a sexual partner. If you are assigned to stop taking anti-HIV medicines after delivery, you may be more likely to pass HIV to a sexual partner than if you continued taking anti-HIV medicines.

There may be other risks to taking part in the Postpartum Part of the PROMISE Study that are not known at this time.

WHAT IF I BECOME PREGNANT AGAIN WHILE ON THE PROMISE STUDY?

If you wish to become pregnant or think you may be pregnant at any time during the study, please tell the study staff right away and we will test you using a blood or urine test. The study staff will talk to you about your choices.

If you get pregnant during the PROMISE Study, you can continue on the study. You can continue the study ARV regimen if you were on study drugs when you got pregnant or receive other treatment according to your local guidelines. If you get pregnant while on study drugs, you will be asked to sign a separate consent to continue to receive study drugs while you are pregnant. Site staff will discuss with you what is known about using the study drugs in pregnancy and what risks there might be.

If you become pregnant again during the study, and are still pregnant at your last study visit, the study staff will contact you again to find out about the outcome of your pregnancy.

WHAT IF MY BABY IS OR BECOMES INFECTED WITH HIV?

If tests show that your baby is infected with HIV, your baby will be referred for HIV care and treatment *[sites: add local referral information as appropriate]*. HIV care and treatment of babies and children are not provided through the PROMISE Study.

ARE THERE BENEFTIS TO ME OR MY BABY TAKING PART IN THIS STUDY?

The strategies used in the Postpartum Part to help prevent a mother from giving HIV to her baby may benefit you and your baby, but no guarantee can be made. Information learned from the PROMISE Study may help other HIV-infected mothers from giving HIV to their babies during breastfeeding. A recent study suggests that taking triple anti-HIV medicines can make it much less likely to pass HIV to a sexual partner. If you are assigned to take three anti-HIV medicines in the Postpartum Part, you may have this benefit. You may get some satisfaction from knowing that you and your baby participated in this study.

WHAT OTHER CHOICES DO MY BABY AND I HAVE BESIDES THIS STUDY?

Joining or continuing in this is voluntary. Instead of being in this study, you have the choice to receive the standard regimen of drugs for prevention of mother to infant HIV transmission during breastfeeding provided at this location. Your doctor will discuss with you the available standard regimen for prevention of mother to infant HIV infection during breastfeeding. Please talk to your doctor about the risks and benefits of these and other choices available to you and your baby.

You will continue to receive regular care whether or not you take part in the study.

WHAT ABOUT CONFIDENTIALITY?

Every effort will be made to keep personal information about you and your baby confidential. This personal information may be disclosed if required by law. Any publication of this study will not use your or your baby's name or identify you or your baby personally.

The outreach workers may contact you so we need to know the best way to reach you (such as home visit or phone call). Your records and those of your baby may be reviewed by the ethics committee overseeing research at this site, the US Food and Drug Administration (FDA), the study sponsor (the US National Institutes of Health) or its agents, the US Office of Human Research Protections, IMPAACT leadership (e.g., staff from the operations center, data management center and network lab), local regulatory authorities, study staff, study monitors, and the drug companies supporting this study.

WHAT ARE THE COSTS TO ME?

There is no cost to you for your study visits, exams, or blood tests or those of your baby. There is no cost to you for the anti-HIV medications used in this study. If you take HIV medicines from another program or provider outside the study, you will need to pay for the medicines, unless the medicines are available free of charge. The study cannot pay for medicines obtained from other programs or providers.

WILL I RECEIVE ANY PAYMENT?

If you have to come to the hospital/clinic *[sites may add or replace with more accurate term]* because of your participation in the study, your transportation and time will be reimbursed to you. You will receive *[site to insert amount]* for each study visit.

WHAT HAPPENS IF EITHER MY BABY OR I ARE INJURED?

It is possible that either you or your baby could experience a problem or injury that would not have occurred if you did not participate in this study. If [the study doctor] determines that you or your baby has been injured as a direct result of being in this study, you and/or your baby will be given immediate treatment for those injuries at no cost to you and then referred for further care if needed. *[Sites: add local information regarding treatment for injury]*.

However, *[the study doctor]* may determine that your or your baby's illness or injury would have happened even if you did not participate in this study. In that case, appropriate care and/or referral will likewise be provided for any illness or injury that occurs during the study *[Sites: Add local information regarding care/referral, and explain whether participants will bear the costs of treatment for non-study-related injury, or if there is some mechanism for covering these costs as well]*.

There are no plans to give you money if you or your baby experiences a complication, whether or not the problem or injury was related to study participation. You will not be giving up any of your legal rights by signing this consent form.

WHAT ARE OUR RIGHTS AS RESEARCH PARTICIPANTS?

Taking part in the PROMISE Study is completely voluntary. You may choose not to participate in the PROMISE study or leave this study at any time. If you decide not to participate or to leave the study early, you and your baby will not be penalized or lose any benefits to which you would otherwise have access outside of the study. If you and your baby do not join the Postpartum Part, you still may qualify to join other parts of the PROMISE study.

We will tell you about new information from this or other studies that may affect you and your baby's health, welfare or willingness to stay in the PROMISE Study. If you want to be informed about the results of the PROMISE study, the study staff *[will contact you when these are available--sites: include local information about how participants can find out about study results if applicable]*.

WHAT DO I DO IF I HAVE QUESTIONS OR PROBLEMS?

For questions about this study or a research-related injury, contact:

- *[insert name of the site investigator or other study staff]*
- *[insert telephone number and physical address of above]*

For questions about you or your baby's rights as a research participant, contact:

- *[name or title of person on the Institutional Review Board (IRB) or other organization appropriate for the site]*
- *[insert telephone number and physical address of above]*

SIGNATURE PAGE

If you have read this consent form (or had it read and explained to you), if all your questions have been answered, and if you agree for you and your baby to take part in this study, please sign your name below.

[For sites participating in the Hair and Adherence sub study: Please indicate whether or not you agree to have your and your baby's hair collected for this part of the study.*]*

[I agree to allow my hair and my infant's to be collected for testing how much anti HIV medicine is found in the hair, as discussed in this consent form]

No ____ Yes ____

______________________________	______________________________
Participant's Name (print)	Participant's Signature and Date
______________________________	______________________________
Legal Guardian's Name (print)<br>(if needed)	Legal Guardian's Signature and Date<br>(if needed)
______________________________	______________________________
Study Staff Conducting<br>Consent Discussion (print)	Study Staff Signature and Date
______________________________	______________________________
Witness Name (print)	Witness Signature and Date (if needed)

5.0 MATERNAL HEALTH COMPONENT OF PROMISE

SCHEMA: Maternal Health Component
(DMC Enrollment Screen/CRF Identifier: 1077BM)

DESIGN: A strategy trial that will enroll and randomize consenting, eligible women with a CD4 count of ≥ 350 cells/mm^3 who were either randomized to receive triple ARV prophylaxis in the Postpartum Component of PROMISE or randomized to receive triple ARV prophylaxis in the Antepartum Component and ineligible for the Postpartum Component. Participants will be randomized to one of two study arms:

Arm A: Continue the study triple ARV regimen

Arm B: Discontinue the study triple ARV regimen

Women on both study arms will follow the same schedule of evaluations. The triple ARV regimen will be resumed if a participant in Arm B reaches an indication for initiation of treatment for her own health.

Women randomized to triple ARV therapy who cease BF but are not eligible for randomization (and women who were randomized to the infant NVP prophylaxis arm in the Postpartum Component of PROMISE) will also be followed as a comparison group.

POPULATION: The study population will include consenting, eligible HIV-infected women with a CD4 count of ≥ 350 cells/mm^3 who were either a) randomized to triple ARV prophylaxis in the Postpartum Component and have ceased BF, reached 18 months postpartum or have confirmed infant HIV infection (in all infants if multiple births) - whichever comes first, or b) randomized to a triple ARV prophylaxis arm in the Antepartum Component but ineligible for the Postpartum Component.

Another comparison group will include women who did not receive triple ARV prophylaxis during either the AP Component or the PP Component (i.e., women randomized to ZDV + sdNVP + TRV tail in the Antepartum Component and/or randomized to infant NVP prophylaxis in the Postpartum Component).

SAMPLE SIZE: There is no specific accrual target for the Maternal Health randomization, because the sample size will depend on the number of women enrolled to the Postpartum Component and the number of women in the Antepartum Component who are ineligible for the Postpartum Component. It is projected that approximately 2,100 BF women from the Postpartum Component will meet eligibility criteria at cessation of BF, 18 months postpartum, or confirmation of infant HIV infection (of all the woman's study infants if multiple births), whichever comes first, and be randomized. It is projected that approximately 100 BF women who are ineligible for the Postpartum Component will meet the eligibility criteria for the Maternal Health Component after delivery and be randomized.

STRATIFICATION: By country, infant age at randomization (< 9 months, 9-12 months, > 12 months) and antepartum ARV intervention (triple ARV prophylaxis vs. ZDV + sdNVP + TRV tail vs. IP/PP ZDV + sdNVP/TVD [late presenters] vs. none [late presenters])

STUDY DRUG REGIMEN:

1077BM Step 1:
Arm A: Continuation of the study triple ARV regimen (as treatment)
Arm B: Discontinuation of the study triple ARV regimen

1077BM Step 2: Step 1 Arm B participants who reach an indication for initiation of triple ARV therapy for their own health as specified in Section 5.521 will be registered to this step. In addition, Step 1 Arm A participants, who reach an indication for triple ARV treatment for their own health while on a triple ARV regimen (but do not require switching to a second line regimen), will be registered to this step. All women entering Step 2 will have a step change entry visit. For women not on a triple ARV regimen, the Step 2 entry visit must be completed prior to initiation of triple ARV therapy.

1077BM Step 3: Participants who are being followed on triple ARV therapy in Step 1 Arm A or Step 2 will be registered to this step if they meet criteria for switching to a second line regimen as specified in Section 5.522. The Step 3 Entry visit must be completed prior to the first dose of the second line regimen.

STUDY DURATION: All women will be followed until 96 weeks after the last woman in the Antepartum Component of IMPAACT 1077BF delivers (approximately 2-5 years, depending on the rate of accrual).

OBJECTIVES:

Primary Objective

1. To compare the rate of progression to AIDS-defining illness or death between study arms.

Secondary Objectives

1. To determine, characterize, and compare the rates of AIDS-defining and HIV-related illnesses, opportunistic infections, immune reconstitution syndromes, and other targeted medical conditions with regard to outcomes and survival.
2. To assess toxicities, including selected Grade 2 laboratory abnormalities (renal, hepatic and hematologic) and all Grade 3 or higher laboratory values and signs and symptoms.
3. To compare emergence of HIV resistance to ARV drugs during the 1st, 2nd and 3rd years following randomization and at end of study
4. To evaluate rates of self-reported adherence to triple ARV therapy and its association with the primary endpoint and with CD4 cell count, HIV viral load, and HIV resistance patterns at 1, 2 and 3 years following randomization
5. To compare quality of life measurements between the study arms at 1, 2 and 3 years following randomization.
6. To investigate changes in plasma concentrations of inflammatory and thrombogenic markers (IL-6, d-dimer, hs-CRP) between arms and to correlate these markers to clinical events.
7. To evaluate cost effectiveness and feasibility of the trial maternal triple ARV therapy strategies.

Figure 5 – Maternal Health Component Randomization (Step 1) in IMPAACT 1077BF (1077BM)

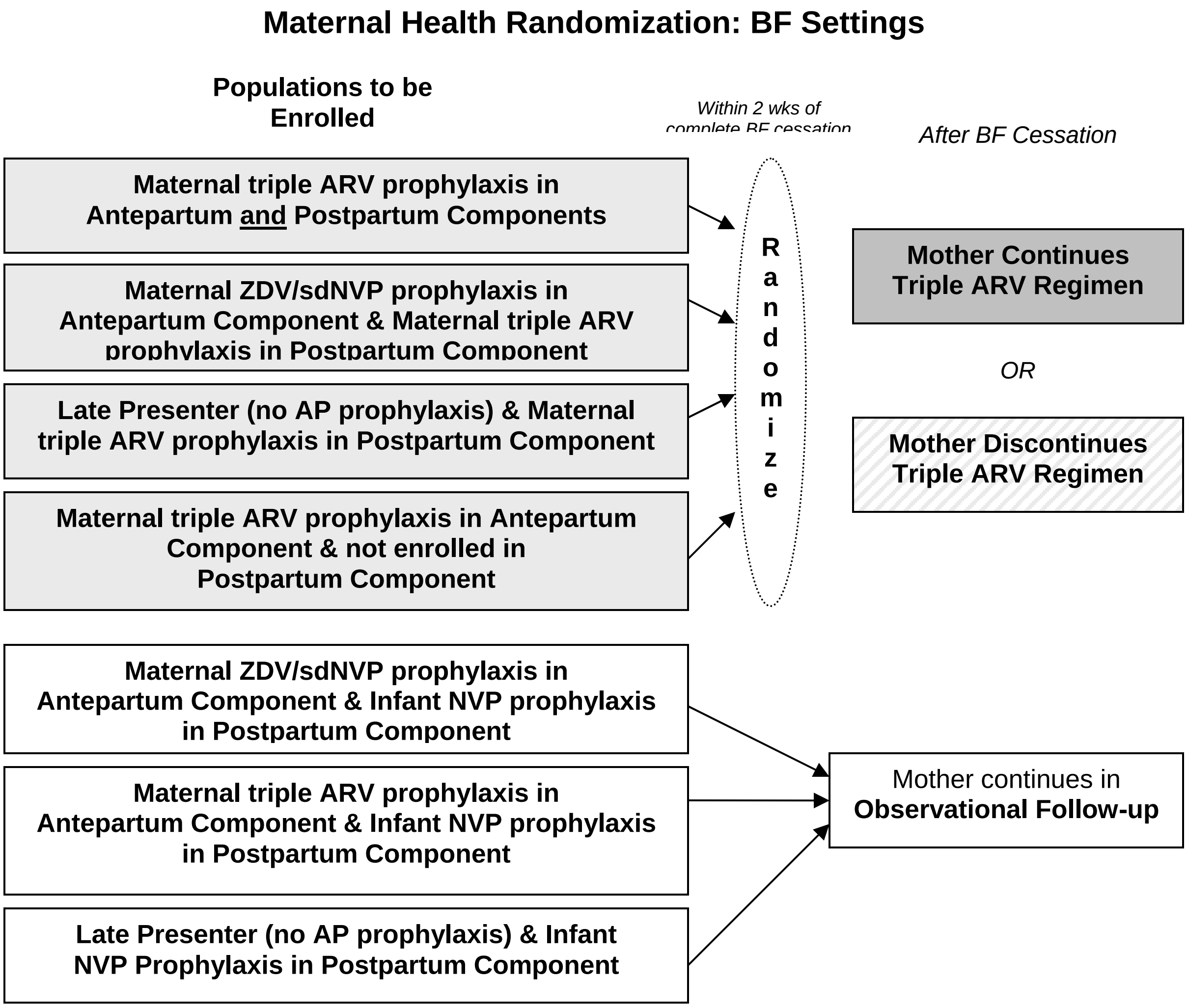

5.1 Overall Design and Rationale (Maternal Health Component)

5.11 Overview

One of the major issues related to the use of various ARV combinations for the prevention of MTCT (PMTCT) of HIV and the length of their use is the effect of these preventive interventions on the health of the mother. The goal of this component of PROMISE is to address the effects on maternal health of use of triple ARV regimens in a PMTCT setting, with two general types of comparisons 1) triple ARV prophylaxis vs. ZDV + sdNVP + TRV tail for PMTCT of HIV and 2) the effects of extending the maternal triple ARV regimen beyond the time needed for PMTCT (e.g., provision of triple ARVs for an indeterminate duration regardless of CD4 cell count, as in “Option B+”). Each comparison will be examined in the setting of an antepartum triple ARV regimen and a postpartum triple ARV regimen leading to four specific scientific questions:

Questions will be addressed by using the comparisons outlined below.

1. Effects of maternal triple ARV prophylaxis versus ZDV + sdNVP + TRV tail interventions for PMTCT:
   a. What is the effect on women of using maternal triple ARV prophylaxis to prevent antepartum [AP]/intrapartum [IP] MTCT, relative to using ZDV + sdNVP + TRV tail to prevent AP/IP MTCT?
   b. What is the effect on women of using maternal triple ARV prophylaxis to prevent postpartum MTCT, relative to using infant NVP to prevent postpartum MTCT?

2. Effects of extending the maternal triple ARV regimen beyond the time needed for PMTCT:
   a. What is the effect on women of extending the AP/IP maternal triple ARV regimen postnatally versus discontinuing the regimen at the time of birth?
   b. What is the effect on women of extending the postpartum maternal triple ARV regimen after the cessation of BF versus discontinuing the regimen with the cessation of BF?

The design of PROMISE, including long-term follow-up of women beyond the time their infants are at risk of MTCT, allows these questions to be answered directly using randomized comparison groups.

These questions will be addressed by comparing maternal outcomes in women randomized to the Antepartum, Postpartum and/or Maternal Health components of PROMISE; with each question addressed using randomized comparison groups. In a secondary analysis, the three sequential PROMISE randomizations will be used to form three comparison groups which correspond to the three WHO PMTCT options: Option A= antepartum ZDV + sdNVP + TRV tail and postpartum infant NVP prophylaxis; Option B= antepartum and postpartum maternal triple ARV prophylaxis; and Option B+ = maternal triple ARV prophylaxis for life, regardless of CD4+ cell count). All three pairwise comparisons of these three groups will be conducted. (see Section 6.3 for details).

5.12 Background and Rationale

In industrialized countries, use of triple ARV regimens during pregnancy for PMTCT, along with scheduled cesarean delivery and avoidance of BF, has reduced rates of transmission to < 2% (1). For women with CD4 lymphocyte counts $\geq$ 350 cells/mm^3 at initiation of a triple ARV regimen for PMTCT prophylaxis, discontinuation of the ARV regimen after delivery has been recommended (2) but the safety of this approach has not been evaluated. A version of PROMISE, 1077HS, will be conducted in resource-rich countries (e.g., US, Brazil) to evaluate this issue in women with higher CD4 counts who received a triple ARV regimen during pregnancy solely for PMTCT, with randomization to continue or stop the ARV regimen postpartum. In resource-limited settings, the potential benefits and risks to maternal health of prolonged maternal triple ARV regimens for PMTCT without maternal health indications and cessation of the ARV regimen after the intervention is completed should be an important part of the consideration related to policies on the use of triple ARV regimens solely for PMTCT. The main question to be addressed in this component of the PROMISE study is the risk and benefits of stopping the triple ARV regimen after completion of pregnancy in women who received this strategy solely for PMTCT and who FF their infant, and after completion of BF for women who received this strategy solely for PMTCT of breast milk transmission, compared to women receiving an antepartum regimen of ZDV + sdNVP + TRV tail and compared to women who continue the triple ARV regimen postpartum.

Data comparing women stopping ZDV at delivery to untreated women in PACTG 076 and women stopping or continuing ZDV monotherapy at delivery in PACTG 185 did not suggest harm from short-term ZDV use for PMTCT (3, 4). Although no increase in disease progression has been seen so far in studies of pregnant women with relatively high CD4 cell counts who stop triple ARV regimens after

delivery (5-7), the available data remain limited and the consequences in terms of safety and toxicity of stopping triple ARV regimens used solely for PMTCT among women with high CD4 cell counts is not known, nor is the benefit of continuing triple ARV regimens indefinitely following initiation during pregnancy or BF given risks of poor adherence and loss to follow-up.

Data from studies comparing scheduled treatment interruptions to continuous therapy in non-pregnant adults have raised concerns that stopping triple ARV regimens, as opposed to continuing them may be detrimental. Several small studies, using various treatment schedules, have not suggested harm from scheduled treatment interruptions, although all have shown lower CD4 lymphocyte counts at the end of the study in treatment interruption groups (8-10). The CD4-guided therapy arm of the Trivacan trial in Africa was stopped early because of a significantly increased rate of serious morbidity in the interruption arm (15.2/100 person years) compared to the continuous therapy arm (6.7/100 person-years, RR 2.27, 95% CI 1.15-4.76) (11). At enrollment, all subjects had CD4 cell counts > 350 cells/mm^3 and HIV RNA below 300 copies/mL. Therapy was re-instituted for a CD4 count < 250 cells/mm^3. The largest trial reported to date, the Strategies for Management of Antiretroviral Therapy (SMART) study, used similar inclusion and therapy interruption/reinstitution guidelines and included 5472 subjects (12). In SMART, the rate of opportunistic disease or death was 3.3/100 person-years in the therapy interruption group and 1.3/100 person-years in the continuous therapy group (HR 2.6, 95% CI 1.9-3.7 for interruption compared to continuous group). In a subset of SMART participants who were either ART naïve at enrollment or off therapy for several months, populations similar to women likely to be initiated on a triple ART regimen during pregnancy, similar inferior results were noted in terms of clinical outcomes among those who interrupted HAART (13). Of note, the hazard ratio for major cardiovascular, renal and hepatic disease was 1.7 (95% CI 1.1-2.5) for the interruption compared to the continuous group, despite less overall ARV drug exposure in the interruption group, an unexpected result. Updated results from the long-term follow-up of the SMART study suggest that re-initiation of therapy after the interruption was associated with a blunted CD4 T lymphocyte response with failure of mean CD4 cell count to reach the baseline value in the interruption arm by end of the study (14). Other key findings from SMART suggest that interruption of HAART is associated with surprisingly rapid changes in inflammatory and coagulation markers (d-dimer, IL-6 and hs-CRP); factors that may influence the risk of various end organ damage (15).

Scheduled treatment interruption studies vary widely in inclusion criteria, interruption schedules and threshold for restarting, thus making comparisons between studies and extrapolation to women receiving triple ARV regimens for PMTCT difficult. In addition, the risk versus benefit considerations for initiation of a triple ARV regimen in women with a CD4 cell count $\geq$ 350 cells/mm^3 with continuation of the ARV regimen indefinitely are unclear. The short-term risk of AIDS and death at CD4 counts $\geq$ 350 cells/mm^3 is low, and the potential absolute risk reductions associated with treatment in such patients are therefore small. Within the ART Cohort Collaboration, the absolute 3-year risk differences between those with CD4 counts 200 to 349 cells/mm^3 and those with CD4 counts $\geq$ 350 cells/mm^3 were only 1.3% (for those with HIV-RNA < 100,000 copies/mL) and 1.7% (for those with HIV-RNA $\geq$ 100,000 copies/mL) (16). These differences were similar through 5 years of observation (17).

Data from the AIDS Therapy Evaluation Project, Netherlands (ATHENA), have demonstrated that patients who start therapy with CD4 counts > 350 cells/mm^3 were significantly more likely to achieve CD4 counts > 800 cells/mm^3 after seven years of HAART than those who initiated therapy at lesser CD4 counts (18). A long-term study based on the Johns Hopkins Clinical Cohort demonstrated that patients who initiated ART with a CD4 count < 350 cells/mm^3 were significantly less likely to achieve a CD4 count > 500 cells/mm^3 after six years of HAART compared to those who started therapy at higher CD4 counts (19).

Factors that might support initiating therapy as early as possible include the possible negative impact of uncontrolled replication on renal, hepatic, neurologic, cognitive and immunological functions (20).

Earlier treatment of HIV infection may also have positive public health implications, as it may reduce HIV transmission (21). This may have significant implication in individuals in discordant relationships (i.e., HIV-infected individuals with HIV-uninfected sexual partners) as was recently demonstrated in the HPTN 052 study (22). HPTN 052 also demonstrated a lower rate of clinical illness when treatment was initiated at CD4+ cell counts between 350 and 550 cells/mm^3, compared to CD4+ cell counts below 250 cells/mm^3; however there was no difference observed in mortality in this study.

Despite possible benefits of treatment of persons with CD4 counts > 350 cells/mm^3, there are also considerations that argue against earlier therapy. First, the potential relative reduction in risk of non-AIDS events/morbidity with antiretroviral therapy as a result of CD4 count increase and viral load suppression is not large. Second, although there are now several reasonably safe and well-tolerated options for first-line regimens, the long-term toxicities remain unknown. Third, ARV treatment requires life-long adherence to therapy. Some patients may find that the need to take daily medications decreases quality of life, even without side effects. Fourth, regimens are expensive and often unavailable to all who require them based on an AIDS-defining illness or low CD4 lymphocyte count in some settings. Additionally, some data from African countries in discordant couples suggest that there may be some reluctance of HIV-infected individuals to initiate life-long treatment solely for prevention of sexual transmission, including concerns related to side effects, inconvenience, adherence requirements, stigma, psychological issues, among others (23). In a study in Kenya, nearly 40% of 181 Kenyan HIV-infected individuals with CD4 count > 350 cells/mm^3 in known HIV serodiscordant partnerships reported reservations about early initiation of treatment solely for HIV prevention (24).

As the use of triple ARV prophylaxis during pregnancy and BF for PMTCT continues to increase worldwide, the risks and benefits of continuing versus stopping these regimens must be evaluated. A critical issue in the management of HIV infection among women is to determine how interventions to reduce perinatal transmission impact maternal health in the short- and long-term. If we find that women who currently do not meet guidelines for initiating a triple ARV therapy for their own health derive a significant benefit from triple ARV regimens for PMTCT, then programs will need to reassess standards of care in many parts of the world where these women currently do not receive a triple ARV regimen for PMTCT. Alternatively, if women who receive a triple ARV regimen for PMTCT incur some penalty in terms of their own health, then this may offset any benefits of a maternal triple ARV strategy for PMTCT. Furthermore, if continuing a triple ARV regimen at the conclusion of the PMTCT intervention is associated with reduced morbidity, these data will add to the growing body of evidence suggesting that earlier initiation of triple ARV therapy has benefits. The design of the PROMISE study provides an opportunity to address several of these crucial questions regarding optimal use of triple ARV regimens for prophylaxis antenatally and during BF and for treatment postpartum and after breastfeeding among childbearing HIV-infected women.

5.13 Study Drugs

While this is a strategy trial rather than an evaluation of specific drug regimens, selected drugs will be available through the study to assure access for all women. The first line regimen for women randomized to continue their triple ARV regimen postpartum is TRV/LPV-RTV. Summary information for drugs in the first line regimen is provided in Sections 2.11 and 4.15.

5.2 Study Design (Maternal Health Component)

5.21 Randomization

Entry and randomization in the Maternal Health Component will occur (i) within two weeks of complete cessation of BF, at 18 months postpartum (72-78 weeks postpartum), or after confirmation of infant HIV infection (of all the woman's study infants), whichever comes first, among women randomized to maternal triple ARV prophylaxis in the Postpartum Component, regardless of the woman's antepartum assignment, or (ii) as soon as the week 1 visit (6-14 days postpartum) but within 28 days after delivery among women randomized to maternal triple ARV prophylaxis during pregnancy in the Antepartum Component who are ineligible (themselves or their infants) for the Postpartum Component. Women will be randomized to continue the triple ARV regimen or to stop the triple ARV regimen. Women who report that they have stopped exposure to breast milk can be screened for eligibility for the Maternal Health Component during the 28 days after last reported breast milk exposure and should be enrolled during the first two weeks (14 days) after reaching complete breastfeeding cessation (defined as completely stopping all exposure to breast milk for $\geq$ 28 days). The triple ARV regimen will be continued during this period until randomization. Women who are randomized to discontinue the triple ARV regimen will do so within 72 hours of randomization.

1077BM Step 1:
Arm A: Continuation of the study triple ARV regimen
Arm B: Discontinuation of the study triple ARV regimen

Women in both arms will follow the same schedule of evaluations. Women in Step 1 Arm A may receive study-supplied ARV medications or they may receive a triple ARV regimen of their choice from outside the study if the regimen meets the protocol definition of triple ARV therapy (HAART) (three or more agents from two or more classes of antiretroviral drugs) and is provided by prescription.

1077BM Step 2: Participants in Step 1 Arm B may resume the triple ARV regimen if they develop the need for treatment for their own health according to the criteria specified in Section 5.521. Additionally, Step 1 Arm A participants will enter Step 2 if they reach an indication for HAART for their own health while on the triple ARV regimen (but do not meet the criteria for switching to a second line regimen). Participants must be registered to Step 2 of the study and all women will have a step change entry visit. For those not on a triple ARV regimen the Step 2 entry evaluations must be completed prior to the first dose of the treatment regimen. Participants in Step 2 may receive study-supplied ARV medications or they may receive ARV therapy of their choice from outside the study if the ARV therapy meets the protocol definition of triple ARV treatment (HAART) (three or more agents from two or more classes of antiretroviral drugs) and is provided by prescription.

1077BM Step 3: Participants from either arm who are being followed on HAART (Step 1 Arm A or Step 2) will be registered to Step 3 if they meet the criteria specified in Section 5.522 for switching to a second line regimen. Step 3 entry evaluations must be completed prior to the first dose of the second-line HAART regimen. The women may receive study-supplied antiretroviral medications or they may receive ARV therapy of their choice from outside the study if the therapy meets the protocol definition of HAART (three or more agents from two or more classes of antiretroviral drugs) and is provided by prescription.

Note: Section 6.0 includes Statistical Considerations for this study component (and all others).

5.22 Study Follow-Up

Women will be followed until 96 weeks after the last delivery occurs in PROMISE (approximately 2-5 years). Women assigned to discontinue their triple ARV regimen should remain off the regimen unless they develop an indication for treatment for their own health (see Section 5.521); women assigned to continue their triple ARV regimen should stay on the regimen without interruption.

5.3 Selection and Enrollment of Subjects (Maternal Health Component)

5.31 1077BM Step 1 (Randomization into Arm A or Arm B)

5.311 Inclusion Criteria (1077BM Step 1)

5.311.1 Randomized to triple ARV prophylaxis as part of the Postpartum Component and has continued triple ARV prophylaxis until the current randomization without treatment interruption (defined as more than seven consecutive days of missed dosing) within the previous 30 days; *OR*
randomized to triple ARV prophylaxis in the Antepartum Component but ineligible for the Postpartum Component and has continued triple ARV prophylaxis until the current randomization without treatment interruption (defined as more than seven consecutive days of missed dosing) within the previous 30 days.

5.311.2 Within two weeks after complete BF cessation is achieved (defined as completely stopping all exposure to breast milk for $\geq$ 28 days); i.e., within 29-42 days of last breast milk exposure, or reached 18 months postpartum (whichever comes first). Women who reach 18 months postpartum while still breastfeeding will be eligible for entry within two weeks before and four weeks after the week 74 visit (week 72-78);

- or if the woman was randomized to triple ARV prophylaxis in the Postpartum Component and her infant is infected and still breastfeeding, she will be eligible for the Maternal Health Component within 42 days of specimen collection for the confirmatory infant HIV NAT;
- or if the woman was randomized to triple ARV prophylaxis in the Antepartum Component but mother-infant pair was ineligible for the Postpartum Component, she will be eligible for the Maternal Health Component beginning at the week 1 visit (6-14 days postpartum) through 28 days after delivery; these women should be randomized as soon as possible - ideally within 6-14 days after delivery;
- or if the woman was randomized to triple ARV prophylaxis in the Postpartum Component and breastfeeding risk for MTCT ceases for other reasons (e.g., infant death or permanent removal from home through legal services or adoption) within 28 days of event.

Note: A mother with multiple births who is assigned to triple ARV prophylaxis in the Postpartum Component will continue her treatment assignment if at least one of her study infants remains uninfected; she may be eligible for the MH Component after cessation of BF of the last uninfected infant or at 18 months postpartum (weeks 72-78 postpartum), whichever comes first; if all of her infants are infected and still breastfeeding, she may be eligible for the Maternal Health Component within 42 days of specimen collection for the confirmatory NAT for the last infected infant.

5.311.3 Provided written informed consent

5.311.4 CD4 cell count $\geq$ 350 cells/mm^3 or greater than or equal to the country-specific threshold for initiation of treatment, if that threshold is > 350 cells/mm^3, on specimen obtained within 30 days prior to entry in 1077BM

5.311.5 The following laboratory values on a specimen obtained within 30 days prior to entry in 1077BM:

- Absolute neutrophil count (ANC) $\geq$ 750 cells/ mm^3
- Hemoglobin $\geq$ 7.0 gm/dL
- Platelet count $\geq$ 50,000 cells/mm^3
- ALT (SGPT) $\leq$ 2.5 x ULN
- Estimated creatinine clearance of $\geq$ 60 mL/min using the Cockroft-Gault equation for women (See 2.411.5)

5.311.6 Intend to remain in current geographical area of residence for the duration of study

5.312 Exclusion Criteria (1077BM Step 1)

5.312.1 WHO Stage 4 disease

5.312.2 Clinically significant illness or condition requiring systemic treatment and/or hospitalization within 30 days prior to entry in 1077BM

5.312.3 Current or history of TB disease (positive PPD without TB disease is not exclusionary)

5.312.4 Use of prohibited medications within 14 days prior to entry in 1077BM

5.312.5 Social or other circumstances which would hinder long-term follow-up as judged by the site investigator

5.312.6 Current documented conduction heart defect (specialized assessments to rule out this condition are not required; a heart murmur alone and/or type 1 second-degree atrioventricular block (also known as Mobitz I or Wenckebach) is not considered exclusionary)

5.312.7 Requires triple ARV therapy for own health (includes women who are on Step 2 of 1077BA or 1077BP and women who are on Step 3 of 1077BA or 1077BP who entered Step 3 for immunologic/clinical disease progression requiring a change in their triple ARV regimen (HAART))

Note: Women on Step 3 of 1077BA or 1077BP who were never on Step 2 and who entered Step 3 for toxicity or virologic failure without clinical or immunologic disease progression requiring a complete change in their triple ARV regimen are eligible for the Maternal Health Component.

5.32 <u>1077BM Step 2</u>

5.321 Inclusion Criteria (1077BM Step 2)

5.321.1
- On Step 1 Arm B (discontinue the study triple ARV regimen arm); OR
- On Step 1 Arm A (triple ARV regimen) and currently on the triple ARV regimen but does not meet the criteria for switching to a second line regimen and entry into Step 3

5.321.2 Reached an indication for triple ARV treatment for her own health as specified in Section 5.521

5.321.3 Willing and able to re-initiate or continue triple ARV therapy

5.322 Exclusion Criteria (1077BM Step 2)

None.

NOTE: A participant should not move to a new step if she has a toxicity that based on the Toxicity Management Guidelines (Appendix II), would necessitate an interruption in therapy;

however, the participant may move to the new step once the toxicity has resolved to a grade that would allow therapy to begin.

5.33 1077BM Step 3 (Women on Step 1 Arm A or Step 2 who require a change in HAART)

5.331 Inclusion Criteria (1077BM Step 3)

5.331.1 On Step 1 Arm A or Step 2
5.331.2 Meets the criteria for switching to a second line regimen, as specified in Section 5.522, while on a triple ARV regimen
5.331.3 Willing and able to initiate an alternate triple ARV regimen (HAART)

5.332 Exclusion Criteria (1077BM Step 3)

5.332.1 On Step 1 Arm B

NOTE: A participant should not move to a new step if she has a toxicity that based on the Toxicity Management Guidelines (Appendix II), would necessitate an interruption in therapy; however, the participant may move to the new step once the toxicity has resolved to a grade that would allow resumption of therapy.

5.34 Enrollment Procedures

Requirements for sites to initiate the PROMISE 1077BF trial, beginning with screening and enrollment of participants in the Antenatal Component (and Late Presenters), are outlined in Section 2.44 and will be detailed in the study Manual of Procedures.

Assessments done as part of the Antepartum Component or the Postpartum Component may serve as screening evaluations for the Maternal Health Component, provided they are performed within timeframes specified in the eligibility criteria. As with each individual PROMISE study component, separate written informed consent for participation in the Maternal Health Component must be obtained before entry. As noted previously, subject enrollment is done through the Data Management Center (DMC) Subject Enrollment System. The appropriate enrollment screen for the Maternal Health Component is identified as 1077BM.

Women randomized to receive triple ARV prophylaxis in the Postpartum Component of 1077BF (who have completely ceased BF or reached 18 months postpartum (week 72-78), whichever comes first, or who have an infant with confirmed HIV infection (all infants if a multiple birth), or MTCT breastfeeding risk ceases for other reasons (e.g., infant death (all infants if multiple birth)) and women randomized to receive triple ARV prophylaxis in the Antepartum Component who are ineligible for or decline participation in the Postpartum Component may be eligible for the Maternal Health Component. Note that a mother with multiple births, such as twins, who is assigned to triple ARV prophylaxis in the Postpartum Component will continue her treatment assignment if at least one of her study infants remains uninfected; she may be eligible for the Maternal Health Component after cessation of BF of the uninfected infant or at 18 months (week 72-78) postpartum, whichever comes first, at which time she may elect to continue to BF her HIV-infected infant. If the infant is breastfeeding but was not eligible for the Postpartum Component based on inability to take NVP (e.g., intolerance or allergy), then the infant will be prophylaxed for at least six weeks (or longer based on current country/WHO guidelines) with another antiretroviral (e.g., ZDV or 3TC) according to the site clinician's judgment. These drugs will be dispensed as prescription non-strategy drugs, and the infant will continue to be followed observationally.

If the mother was on triple ARV prophylaxis antepartum and meets the other eligibility criteria, she will be enrolled in the Maternal Health Component.

The study triple ARV regimen (initiated in the Antepartum or Postpartum Component) will be continued after cessation of BF or until 18 months (week 72-78) postpartum, whichever comes first, while eligibility for the Maternal Health Component is being determined (until either enrolled or determined to be ineligible or unwilling); or for a mother whose infant is HIV-infected (or if multiple births, if all infants are HIV-infected), within 42 days of positive confirmatory HIV test (women of infected infants will be counseled to continue BF their infected infant as per WHO guidelines); or for otherwise eligible mothers who were not eligible for or declined participation in the Postpartum Component the study triple ARV regimen will be continued while eligibility for the Maternal Health Component is being determined, with dosing permitted through day 28 postpartum or for mothers whose infant has died (all infants if a multiple birth), with dosing permitted through day 28 after cessation of risk. Women who meet the eligibility criteria and provide informed consent will be enrolled and randomized in the Maternal Heath Component. Women randomized to stop their triple ARV regimen will be instructed to do so immediately (within 72 hours) and return any remaining drug supplies.

Women who do not meet the eligibility criteria for the Maternal Health Component because of an indication for HAART for their own health (see Section 5.521) will not be randomized but will continue to be followed according to Appendix IA, if willing.

Women who otherwise are ineligible or refuse to participate in the Maternal Health Component will have the study triple ARV regimen discontinued but will continue to be followed observationally on study (as per Appendix IA), if willing.

Women whose infants were randomized to the infant NVP prophylaxis arm in the Postpartum Component will continue follow-up on study after cessation of BF following Appendix IA, as these women provide a comparison group for the women randomized in the Maternal Health Component.

Women whose infants are found to be HIV-infected will be encouraged to continue to BF according to WHO guidelines.

5.35 Co-enrollment Guidelines

Women enrolled in the PROMISE study may be enrolled into observational studies, with no study treatment. Co-enrollment into treatment studies would be on a case-by-case basis and requires the approval of the protocol chairs of both PROMISE and the other trial.

5.4 Study Treatment (Maternal Health Component)

5.41 Drug Regimens, Formulation, Administration and Duration

At entry into the Maternal Health Component (1077BM), women will be randomized in Step 1 to one of two arms:

- ***Arm A* - Continue the study triple ARV regimen**

Regardless of Hepatitis B antigen status, women randomized to continue triple ARV therapy will be provided with Lopinavir-Ritonavir plus fixed dose combination Emtricitabine-Tenofovir disoproxil fumarate (Truvada) as the preferred regimen for this component. While study-supplied LPV-RTV + FTC-TDF is the preferred regimen, study site clinicians in conjunction with participants should determine

the optimal drug combination for each participant. For example, women who may have experienced intolerance or toxicity to one or more of the ARVs in the preferred regimen in a previous study component may continue an alternate regimen in this component. Regimens may also be modified (in consultation with the CMC if required per Appendix II) using study-supplied study drugs (see listing in Section 5.414) and/or non-study drugs. Regardless of source, all maternal triple ARV regimens must include three or more agents from two or more classes of antiretroviral drugs. All ARVs should be prescribed consistent with current package inserts. Fixed dose FTC-TDF-RPV may be used as an alternative first line regimen for mothers who are not able to tolerate or adhere to LPV-RTV or ATV-RTV. Given that FTC-TDF-RPV has thus far only been studied as a first line regimen, consultation with the CMC is required in advance of prescribing this regimen for any study participant.

- ***Arm B* - Discontinue the study triple ARV regimen**

Note: There is no infant study drug dosing as part of the Maternal Health Component; however, infants of participating mothers may be continuing the six week NVP prophylaxis regimen from the AP Component when their mothers are enrolled in the Maternal Health Component.

5.411 Study Drug Supply

The study-supplied study drugs available for this component are Zidovudine (ZDV), Lamivudine (3TC), and fixed dose combination Combivir (3TC-ZDV) (provided by GlaxoSmithKline); Tenofovir disoproxil fumarate (TDF), fixed dosed combination Emtricitabine-Tenofovir disoproxil fumarate (FTC-TDF, Truvada, TRV), and fixed dose combination Emtricitabine-Tenofovir disoproxil fumarate-Rilpivirine (FTC-TDF-RPV, Complera) (provided by Gilead Sciences); Lopinavir-Ritonavir (LPV-RTV) and Ritonavir (RTV) (provided by Abbott); Atazanavir (ATV) (obtained from Emcure Pharmaceuticals); and Didanosine (ddI) and Efavirenz (EFV), which will be obtained from a pharmaceutical supplier. However, all study-supplied drugs may not be available at all study sites; availability will be based on the status of drug regulatory approval for each ARV in each country.

5.412 Study Drug Administration

Atazanavir and Tenofovir disoproxil fumarate-Emtricitabine-Rilpivirine (FTC-TDF-RPV, Complera) must be given with food; all other study drugs may be given with or without food.

5.413 Study Drug Distribution and Accountability

See Section 2.515.

5.414 Formulations of Study-Provided Drugs

Generic name<br>Abbreviation<br>Trade name	Formulation	Appearance	Storage
Zidovudine<br>ZDV<br>Retrovir®	300 mg tablets	Biconvex, white, round, film-coated tablets	15-25°C (59-77°F)
Lamivudine<br>3TC<br>Epivir®	300 mg tablets	Gray, modified diamond-shaped, film-coated tablets	25°C (77°F) - Excursions permitted to 15-30°C (59-86°F). See USP Controlled Room Temperature.
Lamivudine-Zidovudine<br>3TC-ZDV<br>Combivir®	150 mg/300 mg tablets	White, modified capsule shaped, film-coated tablet	2-30°C (36-86°F)
Tenofovir Disoproxil Fumarate<br>TDF<br>Viread®	300 mg tablets	Almond-shaped, light blue, film-coated tablets	25°C (77°F)
Emtricitabine-Tenofovir Disoproxil Fumarate<br>FTC-TDF<br>Truvada®	200 mg/300 mg tablets	Blue, capsule shaped, film-coated tablet	25°C (77°F) - Excursions permitted between 15-30°C (59-86°F). See USP Controlled Room Temperature. Keep container tightly closed. Each bottle contains a silica gel desiccant canister that should remain in the original container to protect the product from humidity.
Lopinavir/Ritonavir<br>LPV-RTV<br>Kaletra®<br>Aluvia®	200 mg/50 mg tablets	Ovaloid, film-coated tablets that will be either red or yellow	20-25°C (68-77°F) - Excursions permitted between 15-30°C (59-86°F). See USP Controlled Room Temperature.
Ritonavir<br>RTV<br>Norvir®	100 mg tablets	White film-coated ovaloid tablets	20-25°C (68-77°F) - Excursions permitted to 15-30°C (59-86°F). See USP controlled room temperature.
Atazanavir<br>ATV	150 and 300 mg capsules	White body with a charcoal gray top with ATV 150 or 300 on both the body and the cap	25°C (77°F) - Excursions permitted between 15-30°C (59-86°F).
Didanosine<br>ddI	400 mg and 250 mg capsules	may vary	25°C (77°F) - Excursions permitted between 15-30°C (59-86°F). See USP Controlled Room Temperature. Store in tightly closed containers.
Efavirenz<br>EFV	may vary	may vary	25°C (77°F) - Excursions permitted between 15-30°C (59-86°F). See USP Controlled Room Temperature. Store in tightly closed containers.
Tenofovir Disoproxil Fumarate/ Emtricitabine/ Rilpivirine<br>TDF/FTC/RPV<br>Complera®	300 mg/200 mg/25 mg tablets	Purplish-pink, capsule-shaped, film-coated, with "GSI" on one side	25°C (77°F) - Excursions permitted to 15-30°C (59-86°F). See USP Controlled Room Temperature. Sore in tightly closed containers.

5.5 Subject Management (Maternal Health Component)

5.51 <u>Management of Mothers Randomized in Step 1 of the Maternal Health Component (1077BM) Following the Postpartum Component (or following the Antepartum Component if Ineligible for Postpartum Component)</u>

Women who provide written informed consent and meet the eligibility criteria for the Maternal Health Component (1077BM) will be randomized to one of two study arms in Step 1. Women in both study arms will follow the schedule of evaluations in Appendix IC. Women are followed until 96 weeks after

the last woman delivers in the Antepartum Component of 1077BF (approximately 2-5 years, depending on the rate of accrual).

5.511 Randomization into Step 1 of the Maternal Health Component (1077BM)

Women who meet the eligibility criteria specified in Section 5.31 will be enrolled and randomized in Step 1 of the Maternal Health Component:

- within 29-42 days of last exposure to breast milk in women who report that they have ceased BF (i.e. up to two weeks after BF cessation as defined above) or at 18 months postpartum while still breastfeeding, whichever comes first (women who reach 18 months postpartum while still breastfeeding may be randomized within 72-78 weeks postpartum); or
- within 42 days of specimen collection for the positive confirmatory HIV test if infant is infected (or if multiple births, if all infants are confirmed to be HIV-infected); or
- at the Week 1 visit (6-14 days postpartum) or within 28 days after delivery if the mother-infant pair was ineligible for or declined participation in the Postpartum Component. (Note: These women should be randomized as soon as possible – ideally within 6-14 days after delivery.); or
- cessation of breastfeeding MTCT risk (infant death); these women should be randomized as soon as possible and within 28 days of cessation of risk.

1077BM STEP 1: Randomization (Mothers)

Arm A	Continue the Study Triple ARV Regimen
Arm B	Discontinue the Study Triple ARV Regimen

5.512 Arm A - Women Randomized to <u>Continue</u> the Study Triple ARV Regimen

Women randomized to continue the triple ARV regimen (for treatment) in Step 1 should remain on the study drug regimen without interruption (unless required for toxicity management) for the duration of the study. A triple ARV regimen (HAART) is defined as three or more drugs from two or more classes of ARVs. The preferred study regimen is LPV-RTV plus fixed dose combination FTC-TDF (Truvada).

<u>1077BM STEP 1 FOLLOW-UP</u>
Women randomized to continue the study triple ARV regimen will have clinical, immunologic and virologic monitoring and follow the schedule of evaluations in Appendix IC. Monitoring and ARV management of women is described in Section 5.52.

5.513 Arm B - Women Randomized to <u>Discontinue</u> the Study Triple ARV Regimen

Women randomized to discontinue the triple ARV regimen in Step 1 will be instructed to stop the regimen immediately (within 72 hours) and return any remaining drug supplies.

These women will follow the schedule of evaluations in Appendix IC, which includes careful clinical and CD4 monitoring, but not routine virologic monitoring. They will start HAART in Step 2 if indicated for their own health (see Section 5.521). They may receive study supplied antiretroviral medications or they may receive ARV therapy of their choice from outside the study if the therapy meets the protocol definition of HAART (three or more drugs from two or more classes of ARVs) and provided by prescription.

1077BM STEP 1 FOLLOW-UP
Women randomized to discontinue their triple ARV regimen will have clinical and immunologic monitoring and follow the schedule of evaluations in Appendix IC. Monitoring and ARV management of women is described in Section 5.52.

5.514 Management of Women Found Ineligible for or Decline to be Enrolled to Maternal Health Component

Women in the Postpartum Component who are found to be ineligible for Step 1 randomization (see Section 5.31) or who refuse randomization will be asked to continue follow-up as per Appendix IA. If such women are ineligible because they meet the criteria for initiation of HAART for their own health (see Section 4.721) either at the time that eligibility for the Maternal Health Component is being determined or any time later, they will enter Step 2 of the Postpartum Component if they meet the eligibility criteria specified in Section 4.52.

5.52 Management of Women in the Maternal Health Component (including ARV management)

For women randomized to continue maternal triple ARV regimen in 1077BM Step 1, the preferred regimen is fixed dose combination FTC-TDF (TRV) plus LPV-RTV. Drugs may be switched as specified in Section 5.522 below (e.g., for failure or toxicity). Subsequent regimens are not defined by the protocol but rather are to be at the discretion of the study clinicians (consultation with the CMC available but not required). Women in Step 1 will follow the schedule of evaluations in Appendix IC.

5.521 1077BM STEP 2 (Women who are found to require treatment)

Women who otherwise meet the eligibility criteria in Section 5.32 will be considered to have reached an indication for triple ARV treatment for their own health and will enter Step 2 if during follow-up they:

- experience clinical progression to an AIDS-defining illness/WHO Stage 4 illness (see Appendix IV); or
- meet country-specific clinical indication(s) for initiation of ARV treatment; or
- have a confirmed CD4 cell count below 350 cells/mm^3 or below the country-specific threshold for initiation of treatment, if that threshold is > 350 cells/mm^3; or
- otherwise require ARV treatment as determined in consultation with the CMC.

NOTE: A participant should not move to a new step if she has a toxicity that, based on the Toxicity Management Guidelines (Appendix II), would necessitate an interruption in therapy; however, the participant may move to the new step once the toxicity has resolved to a grade that would allow resumption of therapy.

Women may receive study-supplied antiretroviral medications or they may receive ARV therapy of their choice from outside the study if the therapy meets the protocol definition of HAART (three or more agents from two or more classes of antiretroviral drugs) and is provided by prescription.

1077BM STEP 2 FOLLOW-UP
Women who enter Step 2 will follow the schedule of evaluations in Appendix IC.

5.522 1077BM STEP 3 (women randomized to continue the triple ARV regimen in Step 1 Arm A or on 1077BM Step 2, who require a change in their regimen; Appendix IC)

Women receiving HAART, either in Step 1 (Arm A) or in Step 2, will have virologic as well as clinical and CD4 monitoring. Women who meet the criteria specified are eligible for the 1077BM Step 3 change in regimen.

The criteria for entering 1077BM Step 3 include:

- Clinical failure defined as development of an AIDS-defining/WHO Stage 4 condition; or any other clinical condition that is considered an indication for HAART by country-specific guidelines OR
- Immunologic failure defined as a confirmed decrease in CD4 cell count to less than any of the following:
  - pre-ARV regimen initiation level (i.e., the baseline CD4 count at study entry), or
  - 50% of the participants peak levels, or
  - 350 cells/mm^3 or the country-specific threshold for initiation of treatment, if that threshold is > 350 cells/mm^3; OR
- Virologic failure defined as confirmed RNA level > 1,000 copies/mL at or after 24 weeks on a triple ARV regimen; see note below for more information on counting weeks on triple ARV regimen); OR
- Significant toxicity requiring a change in the backbone of the regimen, or otherwise requiring a change in more than one class of study drug IF the CMC is consulted and approves the step change *in advance*; OR
- Meets country-specific standard indications for a complete change in regimen; OR
- Otherwise requires a change to an alternate triple ARV regimen as determined in consultation with the CMC.

NOTE: For purposes of defining virologic failure, the 24 weeks referenced above refers to the number of continuous weeks on a triple ARV regimen and includes time on a triple ARV regimen during previous component(s) even if a different triple ARV regimen was taken in previous component(s). Please consult the CMC with any questions related to counting weeks on a triple ARV regimen and/or other aspects of defining failure.

NOTE: If a participant experiences one of the above conditions but the condition is judged by the study clinician as due to non-adherence, systemic illness, or other explanatory circumstance, such that a change of regimen is not indicated, with approval from the CMC, entry into Step 3 is not required.

NOTE: A participant should not move to a new step if she has a toxicity that based on the Toxicity Management Guidelines (Appendix II), would necessitate an interruption in therapy; however, the participant may move to the new step once the toxicity has resolved to a grade that would allow resumption of therapy.

While 1077BM Step 3 triple ARV regimens (HAART) are not defined by this protocol, additional drugs available from the study are described above. 1077BM Step 3 regimens should be determined at the discretion of the study clinicians (consultation with the CMC available but not required). HAART that is not provided by the study may be used if it meets the study definition of HAART (three or more agents from two or more classes of ARVs) and is provided by prescription.

1077BM STEP 3 FOLLOW-UP

Women entered in Step 3 will follow the schedule of evaluations in Appendix IC.

5.523 Women Who Develop TB

Participants who develop TB and are not receiving a triple ARV treatment regimen should enter Step 2 or 3 as applicable and initiate ARV treatment for their own health.

Participants randomized to continue the triple ARV regimen who develop TB and need Rifampin-containing TB treatment while on study may be offered EFV (dose to be determined by site clinician) in place of LPV-RTV if they can use appropriate contraception (as outlined below). All participants on TB treatment may continue to receive TDF and FTC or FTC-TDF (TRV) or 3TC-ZDV (Combivir). These study drug changes will be made available for the duration of the Rifampin-based TB treatment, and for 30 days after stopping Rifampin. Thereafter, the participant will return to her assigned study drug regimen.

NOTE: Participants who are participating in sexual activity that could lead to pregnancy and who are receiving EFV must agree to use two reliable methods of contraception, including a reliable barrier method of contraception together with another reliable form of contraception while receiving EFV and for 12 weeks after stopping EFV. These participants will have pregnancy testing at each study visit while receiving EFV and for 12 weeks after stopping EFV.

5.524 Virologic Monitoring of Women Receiving HAART

Virologic failure is not an endpoint in this trial; however, monitoring viral load is used among individuals receiving HAART treatment for their own health to maximize the benefits of HAART and to determine when treatment should be changed. Therefore, virologic monitoring (Appendix IC) will be provided for women on a triple ARV regimen in Step 1 (Arm A), Step 2 or Step 3.

US DHHS treatment guidelines state that the goal of ARV therapy is sustained suppression of HIV RNA to < 50 copies/mL (or below detectable limits of the available HIV RNA assay). However, the plasma HIV RNA threshold for switching therapy is not precisely defined.

Women receiving HAART, who have a plasma HIV RNA level > 1,000 copies/mL at or after 24 weeks of antiretroviral therapy should return (if possible, within 4 weeks) for confirmatory plasma HIV RNA. Women with confirmed HIV RNA levels > 1,000 copies/mL at or after 24 weeks of initial or second line therapy are strongly encouraged to modify their regimen (Step 3). Women in whom virologic failure is believed to be due to non-adherence, systemic illness, vaccination, or other circumstances determined by the study clinicians, will not be required to switch therapy unless the study clinician advises that therapy should be changed (consultation with the CMC available but not required). Subject should continue scheduled study visits as outlined in Appendix IC.

Study-provided medications will be available to participants who meet Step 3 criteria or participants may access therapy not provided by the study. Therapy choice should meet the protocol definition of HAART and be provided by prescription.

In the event that a participant has reached a confirmed HIV RNA > 1,000 copies/mL but does not wish to change her assigned regimen due to clinical and immunologic stability, she may, remain on her current regimen and continue to be followed on study with clinical and laboratory

monitoring (consultation with the CMC available but not required). If the CD4 cell count falls or the HIV RNA rises, the participant should be strongly advised to change therapy.

Women who develop virologic failure on a triple ARV regimen and move to Step 3 in the Antepartum or Postpartum Component are still eligible for randomization to the Maternal Health Component, as long as they do not have a clinical or immunologic indication for HAART. Women who have viral load > 1000 copies/mL and who report recent non-adherence or who have been off of their triple ARV regimen for toxicity and resumed are still eligible for enrollment into the Maternal Health Component as long as they meet all of the other eligibility criteria. Questions regarding the eligibility of women with virologic failure should be addressed to the CMC.

5.525 Management of Second Line Failure

Participants who experience a confirmed HIV RNA >1,000 copies/mL on second-line HAART in Step 3 or subsequent lines of therapy should be managed according to current standard of care and may continue to receive study provided antiretroviral medications at the discretion of the local investigators, participant and primary care provider. Second line failure due to non-adherence or intolerance may be able to be managed with use of the study provided medications, and decisions will need to be made on a case by case basis. If the participant has never had a CD4 cell count < 350 cells/mm^3, the CMC should be consulted and consideration may be given to careful observation off antiretroviral therapy. Participants who discontinue HAART will be followed on study, off study drug at regular study visits.

5.526 Management of HIV/HBV Co-Infected Women Who Discontinue the Triple ARV Regimen in the Maternal Health Component

HIV/HBV co-infected women who discontinue the triple ARV regimen may be at risk of rebound HBV viremia and subsequent transaminitis. In the Staccato HIV Treatment Interruption Trial, 5/6 HIV/HBV co-infected patients who stopped HAART developed HBV viremia and transaminitis and 1/6 had a severe hepatic flare (25). Additional case reports have also documented transaminitis after HBV-active ART cessation, one resulting in severe hepatitis (26, 27). HIV/HBV co-infected women who discontinue their triple ARV regimen as part of the Maternal Health Component will have transaminases measured in real-time at 4, 8, and 12 weeks and have plasma stored and tested retrospectively for HBV DNA, HBeAg and HBeAb at 8 and 24 weeks following discontinuation. If, after study drug cessation, liver function tests (transaminases or total bilirubin) are Grade 3 or above or if women are symptomatic (e.g., jaundice, severe fatigue), should have careful clinical evaluation and be discussed with CMC.

5.53 <u>Concomitant Medications</u>

All medications/preparations received by mothers during the period of study participation must be documented in the participant's source file, as this information may be needed for assessment of toxicities and adverse events. In addition, all cardiac, diabetic, hepatic, renal drugs, oral antibiotics, OI medications and contraceptives (prescription and non-prescription), all other prescription medications and alternative, complementary medications/preparations (i.e., traditional medicines) will also be recorded on applicable case report forms for entry into the study database. The names of alternative, complementary medications/preparations are not required – only whether or not such substances have been used since the last visit.

Sites must refer to the most recent study drug package insert or investigator's brochure to access additional current information on prohibited and precautionary medications. To avoid drug interaction and adverse events, the manufacturer's package inserts of the antiretroviral and concomitant agent should be referred to whenever a concomitant medication is initiated or dose changed.

Concomitant use of traditional medicines is strongly discouraged while participants are on study.

Information on drugs without trade names, with many marketed forms, or those not available in the US may be found at:

http://www.hiv-druginteractions.org/
http://www.nccc.ucsf.edu/

5.531 Prohibited Medications

A participant who requires any medications considered prohibited while on a study drug must have the study drug held or permanently discontinued. A list of medications that are prohibited with study-supplied drugs will be included on the PSWP of the IMPAACT website.

5.532 Precautionary Medications

Medications which should be used with caution with study-supplied drugs will be included on the PSWP of the IMPAACT website.

5.54 Toxicity Management and Adverse Event Reporting (Maternal Health Component)

- Toxicity management is described in Appendix II.
- The DAIDS Table for Grading the Severity of Adult and Pediatric Adverse Events, Version 1.0, dated December 2004 (with Clarification dated August 2009), (which is available at the following website: http://rsc.tech-res.com) must be followed.
- Case Report Form (CRF) recording requirements are included in Section 7.1.
- Requirements for expedited reporting of serious adverse events (SAEs) are included in Section 7.2.

5.55 Criteria for Treatment Discontinuation

Women may be discontinued from study treatment temporarily or permanently primarily based on toxicity events and tolerability issues. The DAIDS Table for Grading the Severity of Adult and Pediatric Adverse Events, Version 1.0, dated December 2004 (with Clarification dated August 2009) and the Toxicity Management Guidelines (Appendix II) will be used to guide these decisions, in consultation with the CMC when required and/or when desired by the site investigator. Women who are removed from treatment will remain in the study (off study drug/on study) and follow the relevant maternal schedule of evaluations.

Subjects may be discontinued from study drug treatment for any of the following reasons:
- Drug-related toxicity (see Appendix II)
- Second virologic failure with CD4 $\geq$ 350 cells/mm^3
- Requirement for prohibited concomitant medications
- Clinical reasons believed life threatening by the site investigator, even if not addressed in the Toxicity Management Guidelines (Appendix II)

- Request of the primary care provider if she or he thinks that the study treatment is no longer in the best interest of the participant
- Request of the participant
- If recommended by an Ethics Committee/IRB or Data and Safety Monitoring Board
- Significant non-adherence thought by the site investigator to increase the risk of treatment failure

Any dispensed study drug remaining after discontinuation must be collected.

Note: Early discontinuation of study product for any reason is not a reason for withdrawal from the study.

5.56 Criteria for Discontinuation of Study Participation

- Participants will be discontinued from the study for the following reasons:
- Request by the participant to withdraw
- Request of the primary care provider if s/he thinks the study is no longer in the best interest of the participant, after consultation with the CMC
- Participant judged by the investigator to be at significant risk of failing to comply with the provisions of the protocol as to cause harm to self or seriously interfere with the validity of the study results, after consultation with the CMC
- At the discretion of the IMPAACT leadership, NIAID, NICHD, the Office for Human Research Protections (OHRP), the US FDA, the pharmaceutical supplier(s), an in-country national health or regulatory agency, or the IRB/EC
- Imprisonment or involuntary confinement in a medical facility (e.g., for psychiatric illness or infectious disease) for a duration that interferes with timely completion of scheduled study visits

5.57 Women Who Become Pregnant on Study

Women who become pregnant again during study participation will be maintained in study follow-up, and pregnancy outcomes will be analyzed based on the initial study randomizations. Women who are receiving a triple ARV regimen as part of the study when they become pregnant will continue to receive the regimen with modifications as needed; they will also be required to provide separate consent to continue taking the study drugs while pregnant if study-supplied (Appendix V). Women who continue taking LPV-RTV will have a dose increase in the third trimester. Women who are not on a triple ARV regimen when they become pregnant will be treated according to local standard of care.

Pregnancy outcomes should be ascertained and recorded on study CRFs. For participants who are pregnant at the end of the study or participants who are pregnant and decide to discontinue study participation while pregnant, additional post-study contacts should be completed to ascertain pregnancy outcomes. Outcomes may be ascertained based on participant report but medical records should be obtained whenever possible to supplement participant reports.

Sites are also encouraged to prospectively register pregnant subjects in the Antiretroviral Pregnancy Registry by calling the following number in the US: 910-679-1598 or by faxing: 910-256-0637 or by calling the following number in the United Kingdom: + 44-1628-789-666.

5.6 Maternal Health References

(1) Cooper ER, Charurat M, Mofenson L, et al. Combination antiretroviral strategies for the treatment of pregnant HIV-1-infected women and prevention of perinatal HIV-1 transmission. J Acquir Immune Defic Syndr 2002;29:484-94.

(2) US Public Health Service task force recommendations for the use of antiretroviral drugs in pregnant women infected with HIV-1 for maternal health and for reducing perinatal HIV-1 transmission in the United States. July 31, 2012. URL: http://www.aidsinfo.nih.gov/guidelines/html/3/perinatal-guidelines/0/.

(3) Bardeguez AD, Shapiro DE, Mofenson LM, et al for the Pediatric AIDS Clinical Trials Group 288 Protocol Team. Effect of cessation of zidovudine prophylaxis to reduce vertical transmission on maternal HIV disease progression and survival. J Acquir Immune Defic Syndr 2003;32:170-81.

(4) Watts DH, Lambert J, Stiehm ER, Harris DR, Bethel J, Mofenson L, Meyer WA III, Mathieson B, Fowler MG, Nemo G, for the PACTG 185 Study Team. Progression of HIV disease among women following delivery. J Acquir Immune Defic Syndr 2003;33:585-93.

(5) Watts DH, Lu M, Thompson B, et al. Treatment interruption after pregnancy: effects on disease progression and laboratory findings. *Infect Dis Obstet Gynecol.* 2009;2009:456717.

(6) Palacios R, Senise J, Vaz M, Diaz R, Castelo A. Short-term antiretroviral therapy to prevent mother-to-child transmission is safe and results in a sustained increase in CD4 T-cell counts in HIV-1-infected mothers. *HIV Med.* Mar 2009;10(3):157-162.

(7) Tai JH, Udoji MA, Barkanic G, et al. Pregnancy and HIV disease progression during the era of highly active antiretroviral therapy. *J Infect Dis.* Oct 1 2007;196(7):1044-1052.

(8) Krolewiecki AJ, Zala C, Vanzulli C, et al. Safe treatment interruptions in patients with nadir CD4 counts of more than 350 cells/uL: randomized trial. J Acquir Immune Defic Syndr 2006;41:425-9.

(9) Ananworanich J, Gayet-Ageron A, LeBraz M, et al. CD4-guided scheduled treatment interruptions compared with continuous therapy for patients infected with HV-1: results of the Staccato randomized trial. Lancet 2006;368:459-65.

(10) Marchou B, Tangre P, Charreau I, et al, ANRS 106 Study Team. Intermittent antiretroviral therapy in patients with controlled HIV infection. AIDS 2007;21:457-66.

(11) Danel C, Moh R, Minga A, Anzian A,, et al. CD4-guided structured antiretroviral treatment interruption strategy in HIV-infected adults in West Africa (Trivacan ANRS 1269 trial): a randomized trial. Lancet 2006;367:1981-9.

(12) Strategies for Management of Antiretroviral Therapy (SMART) Study Group. CD4+ count-guided interruption of antiretroviral treatment. N Engl J Med 2006;355:2283-96.

(13) Emery S, Neuhaus JA, Phillips AN, and the SMART Study Group. Major clinical outcomes in antiretroviral therapy (ART)-naive participants and in those not receiving ART at baseline in the SMART study. J Infect Dis 2008; 197:1084-6.

(14) El-Sadr W and the SMART Study Group. Risk for opportunistic disease and death after reinitiating continuous antiretroviral therapy in patients with HIV previously receiving episodic therapy: a randomized trial. Ann Intern Med 2008;149:289-99.

(15) Kuller LH, Tracy R, Waldo Belloso W, et al. SMART Study Group. Inflammatory and coagulation biomarkers and mortality and patients with HIV. PLoS Medicine 2008;5:e203-15.

(16) Egger M, May M, Chene G, et al. Prognosis of HIV-1-infected patients starting highly active antiretroviral therapy: a collaborative analysis of prospective studies. Lancet 2002;360:119-29.

(17) May M, Sterne JA, Sabin C, et al. Prognosis of HIV-1-infected patients up to 5 years after initiation of HAART: collaborative analysis of prospective studies. AIDS 2007; 21:1185-97.

(18) Gras L, Kesselring AM, Griffin JT, et al. CD4 cell counts of 800 cells/mm^3 or greater after 7 years of highly active antiretroviral therapy are feasible in most patients starting with 350 cells/mm^3 or greater. J Acquir Immune Defic Syndr 2007; 45:183-92.

(19) Moore RD, Keryly JC. CE4+ cell count 6 years after commencement of HAART in persons with sustained virologic suppression. Clin Infect Dis 2007; 44: 441-6.

(20) McCutchan JA, Wu JW, Robertson K, et al. HIV suppression by HAART preserves cognitive function in advanced, immune-reconstituted AIDS patients. AIDS 2007;21:1109-1117

(21) Porco TC, Martin JN, Page-Shafer KA, et al. Decline in HIV infectivity following the introduction of highly active antiretroviral therapy. AIDS 2004;18:81-8
(22) Cohen MS, Chen YQ, McCauley M et al. Prevention of HIV-1 infection with early antiretroviral therapy. N Engl J Med 2011; 365:493-505.
(23) Talia R. Kahn, Michelle Desmond, Deepa Rao, et al. Delayed initiation of antiretroviral therapy among HIV-discordant couples in Kenya, AIDS Care: Psychological and Socio-medical Aspects of AIDS/HIV, 2012 DOI:10.1080/09540121.2012.712660
(24) Heffron R, Ngure K, Mugo N, Celum C, Kurth A, Curran K, Baeten JM. Willingness of Kenyan HIV-1 serodiscordant couples to use antiretroviral based HIV-1 prevention strategies. J Acquir Immune Defic Syndr. 2012 May 16. [Epub ahead of print]
(25) Nuesch R, Anaworanich J, Srasuebkul P, et al. Interruptions of tenofovir/emtricitabine-based ART in patients with HIV/hepatitis B virus co-infection. AIDS 2008 Jan 2;22(1):152-4.
(26) Sellier P, Clevenbergh P, Mazeron MC, et al. Fatal interruption of a 3TC-containing regimen in a HIV+ patient due to re-activation of chronic hep B virus. Scand J Infect Dis 2004;36(6-7):533-5.
(27) Neau D, Schvoerer E, Robert D, et al. Hep B exacerbation with a precore mutant virus following withdrawal of lamivudine in an HIV- infected patient. J Infect Dis 2000.Sep; 41(2):192-4.

5.7 Sample Informed Consent Form – Maternal Health Component

Informed Consent Form – Maternal Health Component
IMPAACT 1077BF
Breastfeeding Version of the PROMISE Study
(Promoting Maternal and Infant Survival Everywhere)
Protocol Version 3.0, Dated 15 October 2012

Note to Sites: Version number and date of the protocol should be included on the first page of the consent form and the version number and date of the consent form should be included in a header or footer on each page of the consent form along with page numbering in the following format: Page 1 of x, Page 2 of x, Page 3 of x. Sites may omit the tables and diagrams if not appropriate; however, the information contained therein must be adequately conveyed to the participant in text.

INTRODUCTION

You are being asked to take part in this research study because:

- you are infected with human immunodeficiency virus (HIV), the virus that causes AIDS
- you have been receiving medicines called highly active antiretroviral therapy (HAART) to try to reduce the risk of your baby getting HIV.

This study is sponsored by the US National Institutes of Health (NIH). The doctor in charge of this study at this site is: *[insert name of site Principal Investigator]*. Before you decide if you want to join this study, we want you to know about the study. We will explain the study to you and you are free to ask questions at any time. We will ask if you want to join the study. If you do want to join, we will ask you to sign this consent form or make your mark (in front of a witness if needed). You will be offered a copy to keep.

WHY IS THE PROMISE STUDY BEING DONE?

As explained to you previously, the PROMISE Study has been designed to look for the best ways to prevent the transmission of HIV from a mother to her baby during pregnancy, labor and delivery and breastfeeding and ways to make sure that the HIV-infected mother stays as healthy as possible from birth and beyond weaning. The PROMISE study has three goals and is divided into three parts to reach those goals.

PROMISE Goals	Part
Goal 1:<br>To determine the best combination of anti-HIV medications to give to HIV-infected pregnant women to prevent HIV infection in babies during pregnancy or at time of delivery.	Antepartum
Goal 2:<br>To determine the best way to protect the baby from HIV while being breastfed.	Postpartum
Goal 3:<br>To find the best way to take care of the mother's health during and after pregnancy.	Maternal Health

This is a consent form to join the Maternal Health Part of the PROMISE Study.

WHY IS THE MATERNAL HEALTH PART OF THE STUDY BEING DONE?

The goal of this part is to find the best way to take care of an HIV-infected mother's health after her infant is born. To reach this goal, the Maternal Heath Part will:

- Determine if women who received triple anti-HIV medicines during pregnancy and/or during breastfeeding and who continue to receive the triple anti-HIV medications have less chance of getting sick than women who stop the medications. Stopping the anti-HIV medications after use for prevention of transmission to the baby in women who would not be on the medications for their own health is often done in the US and other countries.
- Evaluate the chance of developing HIV that is resistant to HIV medicines or of developing clinical or laboratory abnormalities in women who continue taking triple anti-HIV medications compared to those who stop their anti-HIV medicines.
- Determine whether and how well women will be able to take anti-HIV medicines after delivery or after breastfeeding and how that relates to remaining healthy and having low amount of the HIV in their blood.

Some pregnant women with HIV infection who would not otherwise need HIV treatment for their own health are given treatment with three drugs active against HIV to try to keep the baby from being HIV-infected. We do not know if it is better for the mother's health in the long term if she stops the drugs after delivery (what is usually done now) or continues the drugs indefinitely once started. Some studies in people who are not pregnant have shown that it is better to continue the drugs once started rather than stopping, but people in these studies often were less healthy and had been on treatment longer than you have been during pregnancy. Other studies have not shown that stopping ARV medications leads to more complications than continuing it. The clinical staff will describe the country-specific standard of care for treatment and how this care is different than what you may receive in this part of the study. To see if stopping the drugs is better, worse, or the same in the long term compared to continuing the drugs, the Maternal Health Part of the PROMISE Study will compare the health of women who stop the drugs soon after the baby is born or after breastfeeding is stopped to the health of women who keep taking the drugs.

Only HIV medicines that are approved by the US Food and Drug Administration or local authorities will be used in this study.

WHAT WILL I HAVE TO DO IF I AM IN THIS STUDY?

<u>Screening/First Visit</u>
If you decide that you want you to join the Maternal Health Part of the study, we will need to confirm that you are eligible. All or most of the screening tests will have been done through your participation in the Antepartum or Postpartum parts of the PROMISE Study. Depending on the results of the tests or when they were done, we may need to repeat some of the tests.

If you are not eligible for the Maternal Health Part, you are asked to continue follow up in the PROMISE Study as originally agreed, along with your infant. Study staff will discuss options with you for continuing or discontinuing the triple anti-HIV medications. You would still continue to come for visits every three months like before.

If you are eligible for the Maternal Health Part of the PROMISE Study, the first study visit will occur after delivery if you only participated in the Antepartum part of the study. The first study visit will occur after you completely stop breastfeeding for at least four weeks, you reach 18 months postpartum or your infant is found to have HIV infection if you participated in the Postpartum Part of the study. We will ask how you and your baby are doing, about any non-study medications you may be taking and about how well you are taking your study drugs, if still on them. Depending on the results, we may need to repeat

some of those tests. The specific tests and procedures to be done at this visit are described in the next section.

You will be randomly assigned *[sites—insert locally relevant description here, such as, "like flipping a coin"]* to one of two study groups: either the Stop ARV medications Group or the Continue ARV medications Group.

**Stop Triple Anti-HIV Medications Group**<br>The anti-HIV medications are stopped within six weeks of stopping breastfeeding or diagnosis of infant HIV infection.	OR	**Continue Triple Anti-HIV Medications Group**<br>The anti-HIV medications are continued for the rest of the study.

You and the study staff will know which group you are in. If you are assigned to stop the drugs, the study staff will explain how to stop the drugs. If you are assigned to continue the triple anti-HIV medications, the preferred drugs for treatment after delivery are Emtricitabine-Tenofovir and Lopinavir-Ritonavir. Information on these drugs is provided in this consent form. The study staff will discuss with you switching from zidovudine/lamivudine if you were taking these drugs during pregnancy.

<u>Study Visits</u>
You will have visits at week 4, week 12 and after that every 3 months. Each study visit will last about *[sites—if required by your IRB, insert local information on time required for study visits]*. You will have routine medical check-ups at the study clinic. It is important that you return for all of these study visits. If you do not come for a study visit or if a test result comes back abnormal, the outreach worker will contact you to find out how you and your baby are doing. If at any time, you become sick you should let the study nurse or doctor know right away.

<u>Tests and procedures at the study visits</u>

- <u>Medical history, questionnaire, and physical exam</u>
  We will ask you about any medications you have taken in the past and about how well you are taking the study drugs. You will have a physical exam.

- <u>Blood</u>
  Blood will be collected from you for various tests. Some tests measure how well study drugs are controlling the virus, and other tests will check on your health. The amount of blood taken will vary by visit but at most visits you will have approximately 25-33 mL of blood *[sites include local relevant wording]* taken. You will be given the results of tests that might affect your health care as soon as possible, usually at the next study visit. Some of your blood will be tested immediately, and some of the blood may be kept for a while and used later for study-specified tests.

- <u>Pregnancy test</u>
  If you or the study staff think that you may be pregnant, you will be asked to give an additional 5mL of blood or a urine sample to test for pregnancy. If you are taking a specific anti-HIV drug called Efavirenz, you will have a pregnancy test at each study visit and for three months thereafter. You will be given the results of the pregnancy test as soon as possible.

WHAT HAPPENS IF I DEVELOP AIDS OR MY T-CELLS FALL DURING THE MATERNAL HEALTH PART?

In the event that your disease progresses and you are advised to start treatment for your own health, you will have the option to receive anti-HIV medications from the study. If you prefer to take locally available HIV medicines that is also an option. We would like you to remain in follow-up on the PROMISE Study while taking medications. You will continue to be followed up until the study is completed.

OTHER INFORMATION

The information collected in this study may be used for other IMPAACT-approved research.

HOW MANY WOMEN WILL TAKE PART IN THE PROMISE STUDY?

About 4,700 women will take part in the Maternal Health Part of the PROMISE Study around the world.

HOW LONG WILL I BE ON THE PROMISE STUDY?

You will be in the study from 2 to 5 years, depending on when you join the study. Most women will be in the study for less than 3 years.

WHY MIGHT THE DOCTOR TAKE ME OFF THIS STUDY EARLY?

The study doctor may need to take you off the study early without your permission if the study is cancelled or stopped.

WHY MIGHT THE DOCTOR HAVE ME STOP TAKING THE STUDY MEDICATIONS EARLY?

The study doctor may also need to take you off the study medications early if:

- you are not able to attend the study visits
- you are not able to take the study medications as instructed
- continuing the study medications may be harmful to you
- you need a treatment that you may not take while on the study
- you request to stop the study medications

If you have the study medications stopped early for any reason, you will remain in the PROMISE Study and return for all of your study visits as scheduled.

AFTER THE PROMISE STUDY

After you have finished your PROMISE Study participation, the PROMISE Study will not be able to continue to provide you with the study medications. If continuing to take these or similar medicines would be of benefit to you, the study staff will discuss how you may be able to obtain them *[sites insert local information here]*.

WHAT ARE THE RISKS OF THE STUDY?

Taking part in this study may involve some risks and discomforts. These include possible side effects of the anti-HIV medicines that you and your baby may take, possible risks and discomforts from the study tests, and possible risks to your privacy. More information is given on each of these types of risks below.

<u>Side Effects of Anti-HIV Medicines for Women</u>
Women in the Maternal Health Part of the PROMISE Study may take at least 3 different anti-HIV medicines. Some of the medicines are combined together in one tablet, others come in separate tablets. Until you join the study, we will not know what specific medicines you may take or if your anti-HIV medicines are stopped. Therefore, this form gives information about all the anti-HIV medicines women may take. These are:

- Atazanavir (ATV)
- Didanosine (DDI)
- Efavirenz (EFV)
- Emtricitabine (FTC), taken with tenofovir disoproxil fumarate
- Lamivudine (3TC)
- Lopinavir (LPV), taken with ritonavir
- Rilpivirine (RPV)
- Ritonavir (RTV)
- Tenofovir disoproxil fumarate (TDF)
- Zidovudine (ZDV)

Each of the other medicines can cause side effects, when taken alone and when taken in combination. No new or unexpected side effects are observed with drugs combined in one tablet than those observed when each drug is given separately. The combination drugs that may be used in this part of the study include *[sites: insert locally appropriate names of combination drugs – LPV/RTV; 3TC/ZDV; TDF/FTC; and TDF/FTC/RPV – used at your site]*. Some side effects are minor, while others can be severe. Some are common, while others are rare. If you join the study, the study staff will tell you about the side effects of the specific medicines you will take. They will check for side effects during study visits and tell you what to do if you have any side effects.

First, you should know about the possible severe side effects. These effects are rare, but they can cause serious health problems and can result in death:

- Severe rash. This can be caused by atazanavir, efavirenz or lopinavir/ritonavir, ritonavir.

- Abnormal heart beat, which can result in lightheadedness, fainting, and serious heart problems. This can be caused by atazanavir, lopinavir/ritonavir and ritonavir.

- Inflammation of the pancreas. The pancreas is an organ near the stomach. When the pancreas becomes inflamed, it can cause pain in the belly, nausea, vomiting, and increased fats in the blood. This can be caused by didanosine, efavirenz, lamivudine, lopinavir/ritonavir, ritonavir and tenofovir.

- Inflammation of the liver. The liver is an organ near the stomach. When the liver becomes inflamed, it can cause pain and swelling in the belly, nausea, and vomiting. This can be caused by efavirenz, lamivudine, lopinavir/ritonavir, ritonavir, tenofovir and zidovudine.

- Lactic acidosis, enlargement of the liver, and fatty liver, which can result in liver failure. Lactic acidosis is an imbalance in the blood that can cause weight loss, pain in the belly, nausea, vomiting, tiredness, weakness and difficulty breathing. When the liver is enlarged, it can cause pain especially on the right side of the belly, swelling in the belly, nausea, vomiting and loss of appetite. It can also cause bleeding problems that can result in vomiting blood or dark colored stools. Fatty liver is when healthy liver cells are replaced with fat. Sometimes it causes the liver to be enlarged, but doctors usually find out about it from tests of the blood. These effects can be caused by didanosine, emtricitabine, lamivudine, tenofovir and zidovudine. They occur more often in women, pregnant women, people who are overweight, and people who already have liver problems.

- Kidney damage or failure. The kidneys are organs near the middle of your back (one on each side). Doctors usually find out about kidney damage from tests of the blood. These effects can be caused by tenofovir.

- Severe depression, including suicidal thoughts or acts. This can be caused by efavirenz and rilpivirine.

- Other severe mental problems, including aggressive behavior and abnormal thinking. This can be caused by efavirenz.

- Efavirenz might also cause severe harm to unborn babies if taken during the first month of pregnancy.

You should also know about the more common side effects, which are not severe. There are many possible mild and moderate side effects. Some people who take anti-HIV medicines have some of these effects, other people have different effects.

The more common mild and moderate side effects are listed below:

Overall Body Effects
- Overall weakness, tiredness, or feeling unwell
- Loss of appetite
- Loss of weight
- Changes in the placement of body fat, such as enlargement of the neck, stomach, and breasts and thinning of the arms, legs, and cheeks
- Numbness or tingling in the hands, arms, feet, legs, or around the mouth
- Pain in the hands or feet
- Allergic reaction
- Fever

Effects on Your Skin
- Rash, with or without itching
- Yellowing of the skin
- Darkening of the palms and soles of feet

Effects on Your Head
- Headache
- Runny nose
- Yellowing of the eyes
- Not seeing normally
- Changes in the sense of taste
- Swelling of the face, lips, or tongue

Effects on Your Chest
- Cough
- Shortness of breath
- Heartburn

Effects on Your Belly
- Pain or discomfort in the belly
- Nausea
- Vomiting
- Gas
- Loose or watery stools
- Inflammation of the gall bladder. The gall bladder is an organ near the stomach. If it becomes inflamed, it can cause severe pain.
- Stones in the gall bladder or kidneys. If these stones form, they can cause severe pain.

Effects on Your Muscles and Bones
- Aches or pains
- Loss of muscle
- Muscle weakness
- Bone thinning or softening (which could increase the chance of breaking a bone)

Effects on Your Blood
- Decreased blood cells
- White blood cells help fight infection.
- Red blood cells help store and transport energy through the body. Low red cells can cause weakness, tiredness and dizziness.
- Increased bleeding if you have hemophilia
- Increased blood sugar or development of diabetes
- Increased fats in the blood that may increase the risk of heart problems
- Other changes in blood test results that may indicate problems with the muscles, kidneys, liver, pancreas or gall bladder. The blood tests that may be affected include tests of how well these organs are working, tests of substances made by these organs, and tests of fats in the blood.

Effects on Your Mind or Mental Function
- Drowsiness
- Trouble sleeping
- Unusual dreams
- Difficulty concentrating
- Confusion
- Depression
- Agitation or anxiety
- Exaggerated feeling of well being
- Hallucinations
- Feeling of strangeness or losing touch with reality
- Dizziness

The list above is not a complete list of all side effects for all anti-HIV medicines. As a reminder, if you join the study, the study staff will tell you about the side effects of the specific medicines you will take.

Other Possible Risks of Anti-HIV Medicines for Women

Risk of Resistance: All anti-HIV medicines can cause resistance. When resistance occurs, a medicine no longer works against HIV, which can limit the choices of medicines a person can take against HIV in the future. To avoid resistance, it is important to take anti-HIV medicines as instructed, and not miss doses.

Risk of Immune Reconstitution Syndrome: In some people with advanced HIV infection, symptoms from other infections or certain diseases may occur soon after anti-HIV medicines are started. Some of these symptoms may be life threatening. If you start having new symptoms, or notice that existing symptoms are getting worse after starting your anti-HIV medicines, tell your doctor right away.

Risks with Hepatitis B: Some anti-HIV medicines are active against Hepatitis B. For women who have Hepatitis B, and take anti-HIV medicines that are active against Hepatitis B, there are some risks. The Hepatitis B could become resistant and harder to treat. Also, if women later stop taking the medicines that are active against Hepatitis B, this could cause the Hepatitis B to worsen. If this happens, most women get better quickly without treatment, but in rare cases this has resulted in death.

Risks with Contraception: Some anti-HIV medications can interfere with some contraceptive methods, including pills, injections (shots), and implants (placed under the skin). Because of this, it may be necessary to use different or additional contraceptive methods while taking anti-HIV medicines. The study staff will tell you about the effects of the specific anti-HIV medicines you will take and discuss reliable contraceptive methods with you.

Risks of the Study Tests

Blood drawing may cause some pain, bleeding or bruising where the needle enters. There is a small risk of skin infection at the puncture site.

Possible Risks to Your Privacy

We will make every effort to protect your privacy while you are in this study. Your visits here will take place in private. However, it is possible that others may learn of your participation here and, because of this, may treat you unfairly. There also is a risk to your privacy if someone else taking part in this study knows you.

Other Risks

A recent study suggests that taking HAART can make it much less likely for a person with HIV to pass HIV to a sexual partner. If you are assigned to stop HAART, you may be more likely to pass HIV to a sexual partner than if you continued HAART.

There may be other risks to taking part in the Maternal Health Part and the PROMISE Study that are not known at this time.

WHAT IF I BECOME PREGNANT AGAIN WHILE ON THE PROMISE STUDY?

If you wish to become pregnant or think you may be pregnant at any time during the study, please tell the study staff right away and we will test you using a blood or urine test. The study staff will also talk to you about your choices.

If you get pregnant during the PROMISE Study you can continue on the study. You can continue the study anti-HIV medications if you were taking them when you got pregnant or you can receive other treatment according to your local guidelines. Study staff will discuss with you what is known about using the study drugs in pregnancy and what risks there might be. If you get pregnant while on study drugs, you will be asked to sign a separate consent to continue to receive study drugs while you are pregnant.

If you were assigned to stop taking anti-HIV medications after delivery and are not on them when you get pregnant, you will be advised to restart medication usually given to pregnant women in your area.

If you become pregnant again during the study, and are still pregnant at your last study visit, the study staff will contact you again to find out about the outcome of your pregnancy.

ARE THERE BENEFITS TO ME TAKING PART IN THIS STUDY?

There may be benefit to you from receiving study drugs, but we do not know for sure. There may be no benefit to you from being in the study or your health can worsen if you don’t take the medications as prescribed or develop resistance to the HIV drugs. A recent study suggests that taking triple anti-HIV medicines can make it much less likely to pass HIV to a sexual partner. If you are assigned to continue on the anti-HIV medications, you may have that benefit. Information learned from the PROMISE Study may help other HIV-infected mothers keep from passing HIV to their infants and keep themselves and their babies as healthy as possible. You may get some satisfaction from knowing that you participated in this study.

WHAT OTHER CHOICES DO I HAVE BESIDES THIS STUDY?

Joining or continuing in this is voluntary. Your doctor will discuss with you the available standard ARV regimens for HIV-infected mothers who are no longer breastfeeding and do not meet the requirements for HIV treatment for their own health. Please talk to your doctor about the risks and benefits of these and other choices available to you.

You will continue to receive regular care whether or not you take part in the study.

WHAT ABOUT CONFIDENTIALITY?

Every effort will be made to keep your personal information confidential. This personal information may be disclosed if required by law. Any publication of this study will not use your name or identify you or your baby personally.

The outreach workers may contact you so we need to know the best way to reach you (such as home visit or phone call). Your records may be reviewed by the ethics committee overseeing research at this site, the US Food and Drug Administration (FDA), the study sponsor (the US National Institutes of Health) or its agents, the US Office of Human Research Protections, IMPAACT leadership (e.g., staff from the operations center, data management center and network lab), local regulatory authorities, study staff, study monitors, and the drug companies supporting this study.

WHAT ARE THE COSTS TO ME?

There is no cost to you for your study visits, exams or blood tests. There is no cost to you for the anti-HIV medications used in this study.

WILL I RECEIVE ANY PAYMENT?

If you have to come to the hospital/clinic *[sites may add or replace with more accurate term]* because of your participation in the study, your transportation and time will be reimbursed to you. You will receive about *[Site to insert amount]* for each study visit.

WHAT HAPPENS IF I AM INJURED?

It is possible that you could experience a problem or injury that would not have occurred if you did not participate in this study. If [the study doctor] determines that you have been injured as a direct result of being in this study, you will be given immediate treatment for those injuries at no cost to you and then referred for further care if needed. *[Sites: add local information regarding treatment for injury]*.

However, the study doctor may determine that your illness or injury would have happened even if you did not participate in this study. In that case, appropriate care and/or referral will likewise be provided for any illness or injury that occurs during the study *[Sites: Add local information regarding care/referral, and explain whether participants will bear the costs of treatment for non-study-related injury, or if there is some mechanism for covering these costs as well]*.

There are no plans to give you money if you experience a complication, whether or not the problem or injury was related to study participation. You will not be giving up any of your legal rights by signing this consent form.

WHAT ARE MY RIGHTS AS A RESEARCH PARTICIPANT?

Taking part in the PROMISE Study is completely voluntary. You may choose not to participate in the PROMISE Study or leave this study at any time. If you decide not to participate or to leave the, you will not be penalized or lose any benefits that you would otherwise have access to outside of the study.

We will tell you about new information from this or other studies that may affect your welfare or willingness to stay in the PROMISE Study. If you want to be informed about the results of the PROMISE Study, the study staff will contact you when these are available *[Sites: include local information about how participants can find out about study results, if applicable]*.

WHAT DO I DO IF I HAVE QUESTIONS OR PROBLEMS?

For questions about this study or a research-related injury, contact:

- *[insert name of the site investigator or other study staff]*
- *[insert telephone number and physical address of above]*

For questions about your rights as a research participant, contact:

- *[name or title of person on the Ethics Committee or other organization appropriate for the site]*
- *[insert telephone number and physical address of above]*

SIGNATURE PAGE

If you have read this consent form (and had it explained to you), and if all your questions have been answered and you agree to take part in this part of the PROMISE study, please sign your name below.

______________________________	______________________________
Participant’s Name (print)	Participant’s Signature and Date
______________________________	______________________________
Study Staff Conducting Consent Discussion (print)	Study Staff Signature and Date
______________________________	______________________________
Witness’s Name (print) (if needed)	Witness’s Signature and Date

6.0 STATISTICAL CONSIDERATIONS FOR PROMISE

This section contains a complete statistical considerations section for each of the three components of PROMISE. Sections 6.1, 6.2 and 6.3 describe the statistical considerations for the Antepartum, Postpartum and Maternal Health Components, respectively.

6.1 Statistical Considerations for Antepartum Component of PROMISE

6.11 General Design Issues

As noted earlier, for ease of use by clinical sites, this version of the PROMISE protocol (IMPAACT 1077BF) is specifically for BF settings and includes all three components: the Antepartum, Postpartum, and Maternal Health Components and their respective randomizations; a separate version of the PROMISE protocol has been developed for FF settings (1077FF), which includes only the Antepartum and Maternal Health Components. The analysis of the Antepartum and Maternal Health Components of PROMISE will combine mother-infant pairs from both FF and BF settings. Therefore, this statistical section for the Antepartum Component describes the statistical considerations for the combined analyses of the FF and BF setting mother-infant pairs.

The Antepartum Component of PROMISE is an open label randomized trial in resource-limited settings. HIV-infected pregnant women (whether BF or FF), who are at least 14 weeks gestation and are not yet in labor, and who have CD4 cell count $\geq$ 350 cells/mm^3 will be screened for HBV and randomized in a 1:1:1 ratio to one of three arms: ZDV + sdNVP + TRV tail, 3TC-ZDV/LPV-RTV, or FTC-TDF/LPV-RTV. The primary objectives of the Antepartum Component are to compare the efficacy of ZDV + sdNVP + TRV tail and triple ARV prophylaxis to reduce antepartum/intrapartum MTCT of HIV, and to assess the safety and tolerability of these ARV regimens. Both the 3TC-ZDV/LPV-RTV and the FTC-TDF/LPV-RTV regimens represent the strategy of using triple ARV prophylaxis to reduce transmission, and it is expected that the efficacy of these two triple ARV regimens will be the same. Thus, results from the two triple ARV arms will be combined in the efficacy analyses, and the two triple ARV arms will not be compared with one another with respect to efficacy. However, because these triple ARV arms may differ with respect to the frequency and specific types of serious adverse events, as well as adverse pregnancy outcomes, all three arms will be compared with respect to these safety issues.

The primary efficacy comparison of the Antepartum Component interventions will be based on HIV nucleic acid test (NAT) positivity rates from infant specimens dawn at or prior to the week 1 (day 6 - 14) visit. The choice of the best time point for the primary MTCT outcome measure for the Antepartum Component of the PROMISE study is complex. On the one hand, HIV diagnostic tests have lower sensitivity when given within 2 weeks following HIV infection, and thus HIV infections which occurred just before or during delivery may not be detected until one or two weeks after birth (1). Several studies indicate that the sensitivity of HIV NAT (DNA PCR) exceeds 90% by 14 days of age (1), although more recent unpublished data on MTCT with HIV subtype C virus suggest that the sensitivity reaches 90% by 7 days of age (2). Thus, assessing antepartum/intrapartum HIV transmission by an HIV NAT taken at birth and between days 6-14 of age should capture most, but not all, antepartum/intrapartum transmissions. However, if the primary outcome measure for the Antepartum PMTCT Component is evaluated after the postnatal PMTCT interventions have started (i.e., after day 6-14 of age), the Antepartum MTCT comparisons may be biased by differences between postpartum interventions that vary by antepartum treatment arm.

A simulation project (details available upon request) was conducted to explore the extent of these biases and their effects on the planned analyses of the Antepartum Component of PROMISE under models for the timing of pre- and post-natal HIV infection in infants and on the sensitivity of DNA-PCR. Also

explored was the impact of drawing the specimen for DNA-PCR on day 12 compared with day 7, as well as biases associated with basing the Antepartum Component analysis on an additional DNA-PCR test after the postnatal PMTCT interventions have started (at either day 14, 21, 28 or 42). The results suggest that the extent to which infant infections occurring prior to the week 1 visit are not captured by the birth and week 1 DNA-PCR is generally small, and that with the planned sample size, PROMISE should have adequate power to detect the anticipated differences in Antepartum Component MTCT rates assumed in the sample size calculation. In contrast, comparative analyses of the Antepartum Component intervention arms using a DNA-PCR examination after the postnatal PMTCT interventions have started will distort the validity of the Antepartum Component comparisons when PP efficacies depend on which Antepartum Component intervention was used. In the specific example studied, the actual Type I error increased to over 8%, well over the accepted limit of 5%. Overall, the results support the use of the HIV NAT positivity rate from the birth and week 1 specimens as the primary outcome measures for comparing the Antepartum Component treatment arms. To explore the extent to which the primary outcome measure may have missed infant HIV infections that occurred prior to the week 1 visit, the percentage of formula-feeding infants in 1077FF who had negative HIV NATs at birth and day 6-14 postpartum and a positive HIV NAT at week 6 postpartum or later (and therefore were incorrectly counted as uninfected in the AP Component primary analysis) will be summarized overall and according to Antepartum Component intervention arm. Also, secondary efficacy analyses will be conducted using semi-parametric methods developed by Balasubramanian and Lagakos that take into account the time-dependent sensitivity and timing of diagnostic tests in order to estimate the distribution of timing of MTCT more accurately and to assess the effect of covariates (including treatment assignment) on this distribution (3, 4).

Women are eligible to be randomized if they are at least 14 weeks gestation and are not yet in labor, with no upper limit on gestational age at entry. Thus, some women may be enrolled who are likely to deliver after receiving only a few days or weeks of study treatment, which may be an insufficient duration of treatment for any benefit or harm to become apparent. Two concerns associated with enrolling such women are that it may (a) attenuate the difference between treatment groups and thereby reduce power; (b) lead to inappropriate policy recommendations -- for example, if maternal triple ARV prophylaxis were superior overall, but this was driven by subjects who enrolled relatively early in gestation and there were no advantages if the regimen was initiated at > 37 weeks gestation, a recommendation to adopt a triple ARV prophylaxis regimen for all women might be inappropriate. The PROMISE team decided not to impose an upper eligibility limit on gestational age at entry because, for many subjects, the gestational age will not be known very precisely, and substantial reductions in plasma HIV RNA concentrations have been observed after only a few days to one week of taking a triple ARV regimen. To address the concerns noted above, power calculations were conducted which suggest that the statistical power to detect a difference of 4% vs. 2% in MTCT between study arms will remain ≥ 76% provided that no more than 20% of study participants enroll very late in gestation and do not benefit from the study interventions (i.e., assuming that the MTCT rate among these late-enrolling women would be 4% in both study arms). The percentage of study participants who enroll very late in gestation will be monitored at each interim analysis to ensure that the power of the study is maintained. Also, a secondary analysis will be conducted to assess whether the relative efficacy or safety of the Antepartum Component interventions differs according to gestational age at enrollment (e.g., < 34 weeks versus ≥ 34 weeks), although the study has not been specifically powered to detect such an interaction.

Women who have had ARV for PMTCT in prior pregnancies, including triple ARV prophylaxis, are eligible to enroll. The percentage of women enrolling in the Antepartum Component who have received prior triple ARV prophylaxis for PMTCT is anticipated to be relatively small overall, but may be substantial at certain sites that have participated in clinical trials of triple ARV prophylaxis for PMTCT (e.g., IMPAACT sites in Malawi). While it is unknown whether a woman’s response to triple ARV prophylaxis during pregnancy or to discontinuation of the regimen at delivery might differ if she previously had received triple ARV prophylaxis for PMTCT, these women have been deemed eligible for

PROMISE to make the results of the Antepartum comparisons more broadly generalizable. A secondary analysis will be conducted to assess whether the relative efficacy or safety of the Antepartum interventions differ according to prior ARV history, although the study has not been specifically powered to detect such an interaction.

6.12 Outcome Measures (Antepartum Component)

6.121 Primary Outcome Measures

- Confirmed presence of infant HIV infection defined as HIV NAT positivity of the specimen drawn at either the birth (day 0-5) or week 1 (day 6-14) visit, confirmed by HIV NAT positivity of a second specimen collected at a different time point. Infant HIV status and timing of infection will be classified using the IMPAACT consensus definitions. Cases of uncertain HIV infection status will be reviewed by the Infant Endpoint Review Committee, which will make the definitive determination concerning the presence and timing of HIV infection.
- Grade 3 or higher toxicity (for women, also selected Grade 2 hematologic, renal, and hepatic adverse events), obstetrical complications, and adverse pregnancy outcomes (e.g., stillbirth, preterm delivery at < 37 weeks gestation, and low birth weight < 2,500 grams, and congenital anomalies)

6.122 Secondary Outcome Measures

- Infant HIV infection detected by HIV NAT positivity in the birth sample
- Overall and HIV-free infant survival through 24 months of age (in conjunction with infants in the Postpartum Component)
- Adherence to the maternal ARV regimen, as measured by maternal report
- Maternal and infant viral resistance to the maternal and infant ARV strategies
- Cost effectiveness and feasibility of the trial ARV regimens
- Maternal HIV RNA < 400 copies/mL at delivery
- Antepartum change in HBV DNA viral load between week 8 and baseline levels (using log HBV DNA), among women with detectable HBV DNA viral loads at baseline and other HBV outcome measures; see Appendix VII for additional details on the HBV substudy and its outcome measures.

(Note: Maternal HIV RNA assays will be run in real time for women who are on a triple ARV regimen. Specimens are being collected and stored at all timepoints for women who are not on a triple ARV regimen, and the HIV RNA assays will be run in batch at a later date.)

6.13 Randomization and Stratification (Antepartum Component)

From 14 weeks gestation forward, prior to the onset of labor, eligible women will be randomized in a 1:1:1 ratio to one of three arms: ZDV + sdNVP + TRV tail, 3TC-ZDV/LPV-RTV, or FTC-TDF/LPV-RTV. This differs from Version 2.0 of the protocol in which HBV co-infected women were randomized as stated above, while HBV negative women were only randomized to one of two arms: ZDV + sdNVP + TRV tail or 3TC-ZDV/LPV-RTV.

The randomization will also be stratified according to positive vs. negative HBV infection status (to designate substudy participants) and by country. The number of women who intend to FF will be limited

initially to a total of 1,000 and the number of women who intend to BF will be limited initially to a total 3,400. These limits may be modified if needed as discussed in Sections 6.15 and 6.25.

6.14 Sample Size and Accrual (Antepartum Component)

The redesign of the Antepartum Component of PROMISE in Version 3.0 of the protocol requires that all subjects be randomized in equal proportions to one of three arms: Arm A (ZDV + sdNVP + TRV tail) vs. Arm B (3TC-ZDV/LPV-RTV) vs. Arm C (TRV /LPV-RTV). In the initial version of the Antepartum Component, HBV-negative subjects were randomized in equal proportions to two arms (A vs. B), while HBV-positive subjects were randomized in equal proportions to the three Arms (A vs. B vs. C). For purposes of the power calculations presented below, it is assumed that roughly half of the total antepartum accrual will have taken place by the time protocol Version 3.0 is issued and the average site has received IRB approval. The following table shows what the expected sample sizes will be for each of these three arms, assuming that the current proportion of HBV+ subjects is maintained until implementation of Version 3.0 (after which HBV status will not impact treatment assignment options). Total expected accrual per arm is presented, and expected accrual is also broken down into the time periods before and after Version 3.0 is implemented.

	**Arm**			
**Time Period**	*A*	*B*	*C*	*Total*
Before Version 3.0 implementation	1086	1086	29	2201
After Version 3.0 implementation	733	733	733	2199
Total	1819	1819	762	4400

The rate of infant HIV infection detected at birth or week 1 is anticipated to be approximately 2-4% based on the Cote D'Ivoire (5), MITRA/MITRA-PLUS (6, 7), and PHPT-2 (8) studies. Given the greater complexity, cost, and potentially greater toxicity of antenatal triple ARV prophylaxis for PMTCT compared with the ZDV + sdNVP + TRV tail regimen, the PROMISE team feels that a difference of close to 2% in MTCT with antenatal triple ARV prophylaxis vs. ZDV + sdNVP + TRV tail would be required in order change the current WHO standard of care for women with higher CD4 counts. A sample size of approximately 4,400 mother-infant pairs would provide > 90% power to detect a difference of 4% vs. 2% in MTCT between the ZDV + sdNVP + TRV tail and triple ARV prophylaxis randomization groups, based on a 2-sided Type I error of 5% and allowing for 10% loss to follow-up before or at birth (including still births), and assuming two interim analyses, along with the final analysis, with alpha spent according to O'Brien/Fleming methods. Note that the extension of the upper limit of the window for the week 1 visit in protocol Version 3.0 (increased from day 12 to day 14 postpartum) will yield a slight increase in power, provided that the effect size in these two extra days is consistent with that of the earlier antepartum period, as a few more transmissions may be included in the antepartum analysis. If the MTCT rate in the group with the highest rate were lower than 4%, even smaller absolute differences could be detected with 90% power (e.g., 3% vs. 1.4% or 2% vs. 0.7%). If the MTCT rate in the ZDV + sdNVP + TRV tail group were lower than 4%, even smaller absolute differences could be detected with 90% power (e.g., 3% vs. 1.4% or 2% vs. 0.7%). If the true MTCT rates were similar in both groups, this sample size would provide strong evidence of equivalence in the form of precise (narrow) confidence intervals for the difference in MTCT rates between groups. For example, if the true MTCT rates were 2% in both groups, the expected half-width of the 95% confidence interval for the difference in MTCT rates would be +/-0.9%.

At least 3,400 of the 4,400 mother infant pairs will be enrolled from BF regions to ensure that sufficient numbers of mother-infant pairs will be eligible for randomization in the Postpartum Component, taking

account of the expected rate of 3% MTCT, which would exclude participation in the Postpartum Component.

This sample size of breastfeeding mother-infant pairs would provide > 87% power to detect a difference between the ZDV + sdNVP + TRV tail and triple ARV prophylaxis randomization groups of 4% vs. 2% in MTCT detected at or prior to week 1 (day 6-14) , based on a 2-sided Type I error of 5% and allowing for loss to follow-up and two interim analyses, as described above.

This sample size would provide 90% power to detect a difference of 4% vs. 2% in MTCT detected at birth or week 1 among BF mother-infant pairs in the two randomization groups, based on a 2-sided Type I error of 5% and allowing for loss to follow-up, interim monitoring, and infants infected at birth.

Up to 1,000 mother-infant pairs from FF resource-limited countries will also be enrolled and their data merged with the data from the mother-infant pairs from BF regions to address the optimal antepartum regimen for prevention of MTCT to make the results of the MTCT comparisons more broadly generalizable and contribute to the Maternal Health Component. The numbers of BF and FF mother-infant pairs may be modified if needed, as discussed in Sections 6.15 and 6.25.

The sample sizes specified in the table above would also provide 95% power to detect group differences as small as 25% vs. 20% for safety outcomes, such as preterm birth and low birth weight, on a comparison between antepartum triple ARV prophylaxis vs. ZDV + sdNVP + TRV tail. Thus, such a comparison would be well powered to detect effects of a smaller magnitude to those reported in observational studies in Europe (9) and Cote D'Ivoire (10), where differences of 16.8% vs. 25.5% in preterm birth and 12.4% vs. 22.3% in low birth weight were reported. This is important, because smaller differences than these may be clinically significant. A comparison of 25% vs. 20% event rates between the ZDV + sdNVP + TRV tail arm vs. the 3TC-ZDV/LPV-RTV arm would have have power > .90. A similar comparison between either of these arms and the FTC-TDF/LPV-RTV arm, whose total accrual will be lower, would have power > .77. However, such a comparison would have .99 power to detect effects of the size seen in the Cote D'Ivoire or Europe observational studies cited above.

IMPAACT site investigators estimate that approximately 7,340 potentially eligible BF women deliver per year at the IMPAACT sites in Durban, South Africa (960 per year), Zambia (450 per year), Zimbabwe (990 per year), Uganda (1,630 per year), Moshi, Tanzania (45 per year), Blantyre, Malawi (1,400), Lilongwe, Malawi (1,800), and Pune, India (65 per year) and approximately 1,630 potentially eligible FF women deliver per year at the IMPAACT sites in Durban (1,000 per year), Capetown (230 per year), and Soweto (400 per year). Based on the above projections, we anticipate that accrual could be completed within 2-3 years.

6.15 Monitoring (Antepartum Component)

This section describes the specific monitoring plan for the Antepartum Component. Please also refer to Appendix III, which describes general considerations for the interim monitoring of all components of PROMISE.

The protocol team will review the status of the study regularly. This review will examine reports on accrual, characteristics of participants, retention, data and specimen completeness, and adverse events (including deaths). These reports will present overall results that are pooled across all randomized groups and not broken out according to groups. A full monitoring plan with specific details concerning the content and scheduling of these reports will be disseminated in a separate document before the study opens to accrual.

Accrual to this study will be monitored by the IMPAACT leadership in accordance with standard operating procedures. In addition, the team will monitor feasibility quarterly, first based on site protocol registration and then on accrual. Initially, the team will monitor site protocol registration quarterly to ensure that an adequate number of sites have registered to complete the protocol. If less than one-half of eligible IMPAACT sites have registered after the protocol has been approved for 6 months, the team will re-assess the feasibility of the protocol and determine the reasons sites have not registered, and will possibly amend the protocol accordingly. Once one-half of eligible IMPAACT sites have registered, the team will assess accrual on a quarterly basis. If fewer than 1,200 mother-infant pairs (in FF and BF regions combined) have been enrolled within 12 months after one-half of all eligible IMPAACT sites have opened to enrollment, the team will identify the reasons for lack of accrual and possibly amend the protocol accordingly. Also, if accrual to the Antepartum or Postpartum Component is slower than expected, the team will identify the reasons and may modify the numbers of FF and BF mother-infant pairs to be enrolled to the Antepartum Component accordingly.

The study will also be reviewed by an NIAID sponsored Data Safety and Monitoring Board (DSMB). Interim analyses focusing on safety, study logistics, and the accuracy of sample size assumptions will be reviewed at least annually starting within 12 months after the first woman is randomized. If the actual accrual and/or MTCT rates differ from the assumed rate(s), the overall sample size or numbers of FF and BF mother-infant pairs to be enrolled may be modified accordingly. Interim efficacy analyses will be performed annually once at least 25% of the information on the primary efficacy outcome measure is available. Under the Antepartum Component accrual assumptions, interim efficacy analyses are projected to be performed approximately one and two years after the first woman is randomized, when approximately 33% and 67% of the total information on the primary outcome measure is anticipated to be available. The interim efficacy analysis schedule may be modified if accrual assumptions turn out to be inaccurate or if recommended by the DSMB. A detailed plan for interim analyses will be developed before such analyses are undertaken.

Interim efficacy analyses will be based on group-sequential repeated confidence intervals for the difference in the proportion of infants with a confirmed positive HIV NAT at birth or week 1 (day 6-14) in FF and BF regions combined, using the Lan-DeMets approach with an O'Brien-Fleming spending function. As discussed in Section 6.11, the two triple ARV prophylaxis arms will be combined in this analysis, such that triple ARV prophylaxis is compared with ZDV + sdNVP + TRV tail. If the confidence interval excludes zero, demonstrating that one treatment condition is superior to the other, or if external results convincingly establish the superiority of one treatment condition over the other, consideration should be given to recommending that further enrollment to the inferior treatment condition be discontinued. However, in considering such a recommendation, the DSMB should also consider the consistency of the primary analysis with the results of analyses of secondary efficacy endpoints, maternal and infant safety, adherence, and other factors which may counterbalance the difference in MTCT prevention. If the DSMB decides to recommend discontinuation of further enrollment to the inferior treatment condition, the DSMB should also consider recommending the following actions:

- <u>If the superior treatment condition is maternal triple ARV prophylaxis:</u> Continue to randomize all future women to one of the two triple prophylaxis arms during pregnancy, until the planned sample size of 4,400 mother-infant pairs is reached, to collect further safety data and to permit completion of the Postpartum and Maternal Health Components (following delivery, qualifying mothers and infants would participate in the Postpartum Component and Maternal Health Component of PROMISE).
- <u>If the superior treatment condition is ZDV + sdNVP + TRV tail:</u> Continue to enroll BF mothers and directly assigning them to ZDV + sdNVP + TRV tail, until the total of 4,400 mother-infant pairs needed to proceed to the Postpartum Component is reached; and discontinue enrollment of FF mothers, because the Maternal Health comparisons to which they would contribute would no longer be feasible (see Section 6.3).

- Make public the results of the interim analysis of the Antepartum Component.

The Antepartum Component should not be stopped for equivalence or futility. When the final results of the Antepartum Component of PROMISE are ready, they will be made public, even if other PROMISE components are still ongoing.

6.16 Analysis (Antepartum Component)

Full details of the proposed analyses will be described in a statistical analysis plan that will be developed once enrollment to the study begins and prior to the commencement of analyses for the first review by the DSMB. Hence, here we limit the description of the proposed analyses to those for the primary efficacy endpoint.

As noted in Section 6.11, both the 3TC-ZDV/LPV-RTV and the FTC-TDF/LPV-RTV regimens represent the strategy of using triple ARV prophylaxis to reduce transmission, and it is expected that the efficacy of these two triple ARV regimens will be the same. Thus, results from the two triple ARV arms will be combined in the efficacy analyses and the two triple ARV arms will not be compared with one another with respect to efficacy. However, because these triple ART arms may differ with respect to the frequency and specific types of serious adverse events, as well as adverse pregnancy outcomes, all three arms will be compared with respect to these safety issues.

Analyses will use the principle of intention-to-treat (i.e., using the randomized treatment assignment, whether or not study drugs were actually taken) and will include all randomized mother-infant pairs, except women who were randomized but later discovered to be HIV-negative or not actually pregnant. Women who are randomized and later discovered to have been ineligible for other reasons will be included in the analyses. The final analysis of the primary efficacy objective of the study will be completed when data from follow-up through week 1 (day 6-14) postpartum are available from all mother-infant pairs.

The primary efficacy analysis will be based on a test for the difference between the cumulative MTCT rates at 1 week (6-14 days) of age in the two groups. The final confidence interval will be adjusted for Type I error spent at the interim efficacy analyses, to preserve an overall two-sided Type I error rate of 0.05 for the trial. For multiple births, MTCT will be considered to have occurred if one or more of the siblings has a confirmed positive HIV NAT on a specimen drawn at or prior to the week 1 (day 6-14) visit.

Sensitivity analyses will be undertaken to evaluate whether the handling of missing infant HIV-infection status at birth or 1 week of age might affect the interpretation of the results. Specifically, these analyses will impute HIV-infection status at 1 week of age for each infant so as to (a) minimize the difference between regimens and (b) maximize the difference. The interpretation will need to be more cautious if the results of these analyses suggest different conclusions. Semi-parametric methods developed by Balasubramanian and Lagakos (3, 4) that take into account the time-dependent sensitivity and timing of diagnostic tests will be used, if possible, to estimate the distribution of timing of MTCT and to assess the effect of covariates (including treatment assignment) on this distribution. As noted in Section 6.11, secondary efficacy analyses will be conducted to assess whether the relative efficacy or safety of the Antepartum interventions differ according to gestational age at entry (e.g., < 34 weeks vs. $\geq$ 34 weeks) or prior PMTCT ARV history (e.g., none vs. sdNVP only vs. ZDV + sdNVP vs. triple ARV prophylaxis regimen), although the power to detect either of these interactions is anticipated to be very low. Descriptive analyses will also be performed to examine whether the effect size for the primary efficacy analysis is relatively consistent across versions of the protocol.

6.2 Statistical Considerations for the Postpartum Component of PROMISE

6.21 General Design Issues

The Postpartum Component of PROMISE is an open-label, randomized trial. Mothers who were randomized to the Antepartum Component or were identified as HIV-infected during labor or soon after delivery ("late" presenters), have a CD4 $\geq$ 350 cells/mm^3, delivered a live-born, HIV-uninfected infant who they will BF, and their infants, will be randomized to receive either maternal triple ARV prophylaxis (with infant prophylaxis through 6 weeks of age) or infant NVP prophylaxis given until BF cessation or through 18 months postpartum, whichever comes first**,** unless stopped for toxicity, other medical reasons or confirmed infant HIV infection. The primary objectives are to compare the efficacy of the two randomized arms to reduce postpartum MTCT from BF, and to assess the safety and tolerability of these ARV regimens.

The PROMISE team considers HIV-free survival at 24 months of age to be a very important objective. However, postnatal MTCT was chosen as the primary objective and HIV-free survival, as well as overall survival, as secondary objectives because the primary effect of the study interventions are expected to be on MTCT, and because it is anticipated that only a minority of infant deaths by month 24 will be caused by HIV infection in most of the anticipated study sites.

The primary objective is to compare the relative efficacies of maternal triple ARV prophylaxis and infant NVP prophylaxis during the entire time that the infants are BF and at risk of MTCT, rather than for a fixed period of time after delivery (e.g., through six or 12 months of age). Thus, if the durations of BF differ in the two study arms, this could lead to difficulties of interpretation and may introduce bias. For example, suppose that the mean duration of BF turns out to be 9 months in the maternal prophylaxis arm and 7 months in the infant prophylaxis arm, and the MTCT rate is lower in the infant prophylaxis arm. In that case, it would not be clear whether the lower apparent reduction in MTCT is due to the infant NVP or shorter duration of exposure to HIV. It is quite plausible that the BF durations may differ in the two PROMISE postpartum study arms because (a) the mother's health status (which will be affected by maternal triple ARV prophylaxis but not infant NVP) might influence BF duration, and (b) knowledge of the subsequent (post-BF cessation) randomizations might affect maternal decisions about how long to BF; for example, knowing that she might be taken off triple ARV prophylaxis upon BF cessation might cause a mother to delay BF cessation. The PROMISE team discussed several possible approaches to try to minimize any such biases while at the same time allowing women flexibility in deciding how long they will BF, and decided that the most feasible approach would be to (a) stratify the Postpartum randomization by country, as sites are expected to vary with respect to BF durations, (b) stratify the analysis of results by each site's suggested minimum duration of BF (sites will be queried annually regarding the suggested minimum duration of BF and usual duration of BF outside PROMISE), and (c) censor the primary analysis of postpartum intervention arms for each site at the time of their suggested minimum BF duration. The properties of this approach were investigated and compared to several alternative approaches in a simulation study (details available upon request). It was found that the proposed analysis is robust in preserving desired Type I error rates without sacrificing substantial power, and superior to other adaptations of the log rank test which censor an infant's results at later times (such as at the first visit when it is determined that they are no longer BF).

6.22 Postpartum Component Outcome Measures (measured from time of randomization)

6.221 Primary Outcome Measures

- Confirmed presence of infant HIV infection, defined as HIV NAT positivity of a specimen drawn at any post-randomization visit (i.e., any visit after the week 1 (day 6-14) visit), confirmed by HIV NAT positivity of a second specimen drawn at a different time point.

Infant HIV status and timing of infection will be classified using the IMPAACT consensus definitions. Cases of uncertain HIV infection status will be reviewed by the Infant Endpoint Review Committee, which will make the definitive determination concerning the presence and timing of HIV infection.

- Grade 3 or higher adverse events (for women, also selected Grade 2 hematologic, renal, and hepatic adverse events)

6.222 Secondary Outcome Measures

- Infant HIV-free survival through 24 months post-delivery
- Overall infant survival through 12 and 24 months post-delivery
- Adherence to the maternal and/or infant ARV regimens, as measured by maternal report and hair measures
- Rates and patterns of maternal and infant resistance to the maternal and infant ARV regimens
- Cost-effectiveness and feasibility of the study ARV prophylaxis regimens
- Pharmacokinetic parameters of antiretroviral drugs measured in maternal plasma, hair, breast milk, and infant blood (plasma or dried blood spot) samples collected at birth, weeks 1, 6, 14, 26, and subsequent visits during breastfeeding (see Section 4.8 for details of the pharmacokinetic study and data analyses)
- Functional maternal antibody and HIV-envelope binding responses in breast milk and plasma, until cessation of breastfeeding or 18 months postpartum whichever comes first

6.23 Randomization and Stratification (Postpartum Component)

No later than the week 1 (day 6-14) postpartum visit, eligible mothers and infants will be randomized in a 1:1 ratio to receive maternal triple ARV prophylaxis or infant NVP prophylaxis. The randomization will be stratified according to country and the antepartum/Intrapartum maternal ARV prophylaxis [triple ARV prophylaxis regimen vs. ZDV + sdNVP + TRV tail vs. ZDV + sdNVP + TRV tail (late presenter) vs. none (late presenter)]. The number of late presenters randomized to the Postpartum Component (1077BP) will initially be limited to 1,550 mother-infant pairs; this limit may be modified if needed as discussed in Section 6.25. There will be no numeric limit on the number of late presenters registered to 1077BL; instead, registration of late presenters to 1077BL will be discontinued when the required number of late presenting mother-infant pairs have been randomized to 1077BP. Randomized mother-infant pairs for whom the infant HIV NAT result on the week 1 (day 6-14) specimen subsequently is determined to be positive will be excluded from the primary efficacy analysis of the Postpartum Component. In the case of a multiple birth, all infants will be randomized to the same study arm.

6.24 Sample Size and Accrual (Postpartum Component)

The postnatal MTCT rate through 6 months postpartum is anticipated to be approximately 1-3% among early presenters based on the MITRA and MITRA-PLUS studies (6, 7) and approximately 5-7% among late presenters based on the SWEN and PEPI-Malawi studies (11, 12). If late presenters represent about 33% of the overall sample size, the combined postnatal MTCT rate at 6 months would be approximately 2-4%. If the postnatal MTCT rate increases by approximately 0.35% per month after 6 months postpartum (half the estimated rate of 0.7% reported in the BHITS meta-analysis) (13) and the mean breast-feeding duration across all sites is approximately 9 months (based on information from the IMPAACT site investigators indicating that the usual duration of breast-feeding is 4-6 months at the Uganda, Durban, and Pune IMPAACT sites, 6-9 months at the IMPAACT sites in Tanzania and Malawi and 15-18 months at the Zimbabwe and Zambia IMPAACT sites), the projected postnatal MTCT rate at BF cessation would be approximately 2-4% among early presenters and 6-8% among late presenters. If late presenters represent about 33% of the overall sample size, the combined postnatal MTCT rate at BF cessation would be approximately 3-5%.

The primary efficacy analysis will compare postnatal MTCT between the randomization groups with early-presenters (women-infant pairs enrolling from the Antepartum Component) and late-presenters combined. A total sample size of approximately 4,650 mother-infant pairs randomized would provide at least 90% power to detect a difference of 5% vs. 3% in postnatal MTCT at BF cessation between the randomization groups, based on a 2-sided Type I error of 5% and allowing for loss to follow-up (10%) and interim monitoring (3%). If the true MTCT rates were similar in both groups, this sample size would provide strong evidence of equivalence in the form of precise (narrow) confidence intervals for the difference in MTCT rates between groups. For example, if the true MTCT rates were 3% in both groups, the expected half-width of the 95% confidence interval for the difference in MTCT rates would be +/-1%.

An important secondary efficacy analysis will assess whether the relative efficacy of the maternal prophylaxis regimen vs. infant NVP for preventing postnatal MTCT differs between early- and late-presenters by means of an interaction test. One plausible interaction scenario might be no difference in MTCT among early presenters (e.g., 4% postnatal MTCT in both arms) but a 50% reduction in MTCT among late presenters (e.g., 6% vs. 3% MTCT). Of the approximately 3,400 breast-feeding women randomized in the Antepartum PMTCT Component of the PROMISE study, approximately 3,100 of them would be available to be randomized in the Postpartum Component of PROMISE (assuming 5% loss-to-follow up before or at birth and a 3% MTCT rate at birth), so that a total of 1,550 late presenters would need to be randomized to reach the target sample size of 4,650 mother-infant pairs. A sample size of 3,100 early presenters from the Antepartum Component and 1,550 late presenters would provide at least 67% power to detect the interaction described above – no difference in MTCT among early presenters and a 50% reduction among late presenters based on a 2-sided Type I error of 10% and allowing for loss to follow-up (10%). An interaction p-value of 0.10 or smaller would be interpreted as a possible interaction, in which case separate confidence intervals for the difference in postnatal MTCT between randomized arms would be computed for the early-presenters and late presenters. With 3,100 early presenters from the Antepartum Component and 1,550 late presenters randomized, the expected half-width of the 95% CI for the difference in postnatal MTCT would be +/-1% among early presenters from the Antepartum Component (assuming true MTCT rates of 4% in both arms) and +/-2% among late presenters (assuming true MTCT rates of 6% vs. 3% in the two arms).

It will also be of interest to assess whether the relative efficacy of the maternal triple ARV prophylaxis versus infant NVP for preventing postnatal MTCT differs among early presenters who were randomized in the Antepartum Component to receive triple ARV prophylaxis versus those randomized to receive ZDV + sdNVP + TRV tail. For example, if the maternal triple ARV regimen takes 1-2 weeks after initiation to reduce maternal viral load sufficiently to fully protect against MTCT, while infant NVP provides full protection much more quickly, it is possible that the two strategies might have similar efficacy among women who received triple ARV prophylaxis during pregnancy, but that infant NVP prophylaxis could be superior to maternal triple ARV prophylaxis among women who received ZDV + sdNVP + TRV tail during pregnancy. The power to detect an interaction between the Antepartum Component and Postpartum Component interventions will be limited unless the interaction is very large. For example, the sample size of approximately 3,100 early presenters from the Antepartum Component (half of whom were randomized to antepartum triple ARV prophylaxis and half of whom were randomized to antepartum ZDV + sdNVP + TRV tail) would provide only 37-45% power to detect an interaction of no difference in BF MTCT with infant NVP prophylaxis versus maternal triple ARV prophylaxis among women who had antepartum triple ARV prophylaxis but a 2% difference (i.e., 4% vs. 2%, or 3% vs. 1%) in BF MTCT with infant NVP versus maternal triple ARV prophylaxis among women who had ZDV + sdNVP + TRV tail, based on a 2-sided Type I error of 10% and allowing for loss to follow-up (10%). However, this sample size would provide 65-70% power to detect an interaction of no difference in BF MTCT among women who had antepartum triple ARV prophylaxis but a 3% difference (4% vs. 1%) in BF MTCT among women who had antepartum short course ZDV + sdNVP + TRV tail,

based on a 2-sided Type I error of 10% and allowing for loss to follow-up (10%). As noted above, if there were evidence of an interaction, separate confidence intervals for the difference in postnatal MTCT between randomized arms would be computed for the early-presenters who had received antepartum triple ARV prophylaxis and those who received antepartum ZDV + sdNVP + TRV tail, and these confidence intervals would have expected half-width of approximately +/-1%.

IMPAACT site investigators estimate that approximately 1,140 potentially eligible late-presenting women with CD4 counts ≥ 350 cells/mm^3 deliver per year at the IMPAACT sites in Uganda (420), Lilongwe, Malawi (165 per year), Blantyre, Malawi (320 per year), Durban (105 per year), Zambia (65 per year), Zimbabwe (30 per year), Moshi, Tanzania (15), and Pune, India (20 per year). Based on the above projections, we anticipate that accrual of 1,550 late presenters could be completed within 2-3 years. Because the survey of IMPAACT site investigators also indicated that on average 62% (range 50-90%) of late presenters have CD4 counts ≥ 350 cells/mm^3, we project that approximately 2,500 late presenters (range 1,725-3,100) will have to be screened to identify 1,550 late presenting women with CD4 counts ≥ 350 cells/mm^3.

6.25 Monitoring (Postpartum Component)

This section describes the specific monitoring plan for the Postpartum Component. Please also refer to Appendix III, which describes general considerations for the interim monitoring of all four Components of PROMISE.

The protocol team will review the status of the study regularly. This review will examine reports on accrual, characteristics of participants, retention, data and specimen completeness, and adverse events (including deaths). These reports will present overall results that are pooled across all randomized groups and not broken out according to groups. If accrual is slower than expected, the team will identify the reasons for lack of accrual and may modify the number of late presenters to be enrolled accordingly (see also Section 6.15 for discussion of possible changes to the numbers of BF and FF women to be enrolled to the Antepartum Component). A full monitoring plan with specific details concerning the content and scheduling of these reports will be disseminated in a separate document before the study opens to accrual.

The study will also be reviewed by an NIAID sponsored Data Safety and Monitoring Board (DSMB). Interim analyses focusing on safety, study logistics, the accuracy of sample size assumptions and the duration of breastfeeding will be reviewed at least annually starting within 12 months after the first mother-infant pair is randomized. If the actual accrual and/or MTCT rates differ from the assumed rate(s), the overall sample size or the number of late presenting mother-infant pairs to be enrolled may be modified accordingly. Interim efficacy analyses will be performed annually, when at least 25% of the information on the primary efficacy outcome measure is available. Under the Antepartum and Postpartum Component accrual assumptions, interim efficacy analyses are projected to be performed approximately 1, 2 and 3 years after the first woman is randomized, when approximately 25%, 50%, and 75% of the total information on the primary outcome measure is anticipated to be available. The interim efficacy analysis schedule may be modified if accrual assumptions turn out to be inaccurate or if recommended by the DSMB. A detailed plan for interim analyses will be developed before such analyses are undertaken.

Interim efficacy analyses will be based on group-sequential repeated confidence intervals for the difference between treatment groups, using the Lan-DeMets approach with an O'Brien-Fleming spending function. If the confidence interval excludes zero, demonstrating that one arm is superior to the other, or if external results convincingly establish the superiority of one arm over the other, consideration should be given to recommending the following actions:

- If postpartum infant NVP prophylaxis is found to be superior to maternal triple ARV prophylaxis for preventing BF MTCT and/or infant survival, consideration should be given to recommending modification of the Postpartum Component randomization to maternal triple ARV regimen versus no maternal ARV regimen, with all infants receiving NVP prophylaxis (provided there are no concerns about overdosing infants).
- If postpartum infant NVP is found to be inferior to the maternal triple ARV regimen for preventing BF MTCT and/or infant survival, consideration should be given to recommending modification of the Postpartum Component randomization so that all future BF mothers will be directly assigned to the maternal triple ARV regimen until the planned sample size Maternal Health Component of PROMISE is reached.

The consistency of the primary analysis with the results for secondary efficacy endpoints and safety, and consistency with specific other components of PROMISE, particularly the Maternal Health Component, should be considered prior to recommending stopping a study arm. For example, because of possible long-term risks of a triple ARV regimen to the mother, a sustained benefit to (maternal) mortality and progression-free survival, as well as no contraindications with respect to infant MTCT or survival, would be required to consider stopping the infant NVP arm (see also Section 6.35).

6.26 Statistical Analysis (Postpartum Component)

Full details of the proposed analyses will be described in a statistical analysis plan that will be developed once enrollment to the study begins and prior to the commencement of analyses for the first review by the DSMB. Hence, here we limit the description of the proposed analyses to those for the primary efficacy endpoint.

Analyses will use the principle of intention-to-treat (i.e., using the randomized treatment assignment, whether or not study drugs were actually taken) and will include all randomized mother-infant pairs, including those who were later discovered to have been ineligible. The primary analysis will be based on all randomized infants found to be HIV NAT negative for the specimens taken at the week 1 visit (day 6-14 specimens), and a log-rank test that is stratified by and censored at the suggested minimal duration of breast-feeding of the participating sites. The final confidence interval will be adjusted for Type I error spent at the interim efficacy analyses, to preserve an overall two-sided Type I error rate of 0.05 for the trial. Analyses will ignore the specific postpartum triple ARV prophylaxis regimen received. It is anticipated that most women randomized to the postpartum maternal triple ARV prophylaxis arm will receive TRV/LPV-RTV, but it is acknowledged that the site PI on a case-by-case basis may continue some women on 3TC-ZDV/LPV-RTV if they received it in the Antepartum Component if switching to a different triple ARV regimen is judged not in the best medical interests of the woman. No analyses comparing FTC-TDF/LPV-RTV vs. 3TC-ZDV/LPV-RTV in the Postpartum Component will be performed, because the selection of women receiving the latter would be clearly susceptible to selection bias.

An important secondary objective is to estimate overall and HIV-free infant survival at 24 months of age for the two Postpartum Component interventions and for each of the four combinations of the Antepartum and Postpartum Component interventions. If there is no qualitative interaction between Antepartum and Postpartum Component interventions, the 24-month survival and HIV-free survival for each of the four combined strategies can be estimated efficiently by estimating these outcome measures for the Antepartum and Postpartum Components separately, and then combining these two estimates, as follows:

Probability (no event by 24 months PP) =
Probability (no AP event)*
Probability (no event by 24 months postpartum | no AP event).

If there is a qualitative interaction, it would not be valid to combine estimates for the Antepartum and Postpartum Components, but information (albeit less precise) about the efficacy of the 4 strategies could still be obtained by estimating overall and HIV-free infant survival separately for each of the 4 combinations of the Antepartum and Postpartum Component interventions.

6.3 Statistical Considerations for Maternal Health Component of PROMISE

6.31 General Design Issues

As noted earlier, for ease of use by clinical sites, this version of PROMISE is specifically for BF settings (IMPAACT 1077BF), and includes all three PROMISE components: Antepartum, Postpartum and Maternal Health randomizations. A separate version of PROMISE has been developed for FF settings (IMPAACT 1077FF), which includes only the Antepartum and Maternal Health randomizations. However, the analysis of the Maternal Health Component of PROMISE will combine mother-infant pairs from both FF and BF settings. Therefore, this statistical section describes the statistical considerations for the combined analyses of the FF and BF mother-infant pairs for the Maternal Health Component endpoints.

The Maternal Health Component of PROMISE addresses therapeutic questions for women from low-resource countries who participated in either the Antepartum Component, the Postpartum Component or both. In particular, this component is designed to address the effects on maternal health of use of a triple ARV regimen for PMTCT, with two general types of primary comparisons 1) comparison of triple ARV prophylaxis versus the less complex ZDV-based ARV prophylaxis regimen and 2) comparison of the effects of extending use of the maternal triple ARV regimen beyond the time needed for PMTCT of HIV versus stopping the ARV regimen when no longer needed for PMTCT. We will examine each primary comparison in the setting of antepartum triple ARV prophylaxis and postpartum triple ARV, leading to four specific scientific questions:

1. *Effects of maternal triple ARV prophylaxis versus the non-triple ARV prophylaxis regimen (ZDV + sdNVP + TRV tail) for MTCT interventions:*
   a. What is the effect on women of using a maternal triple ARV regimen to prevent antepartum [AP]/intrapartum [IP] MTCT, relative to using ZDV + sdNVP + TRV tail to prevent AP/IP MTCT?
   b. What is the effect on women of using a maternal triple ARV regimen to prevent postpartum MTCT, relative to using infant NVP to prevent postpartum MTCT?

2. *Effects of extending use of the maternal triple ARV regimen beyond the time needed for PMTCT:*
   a. What is the effect on women of extending the AP/IP maternal triple ARV regimen postnatally versus discontinuing the triple ARV regimen at the time of birth?
   b. What is the effect on women of extending the postpartum maternal triple ARV regimen after the cessation of risk for BF MTCT versus discontinuing the maternal triple ARV regimen with the cessation of risk for BF MTCT?

These four scientific questions will be addressed using the following four primary comparisons:

Maternal Health Comparisons #1a and 1b address the relative safety and efficacy of a triple ARV regimen, when used to prevent MTCT, compared to a non-triple ARV (ZDV + sdNVP + TRV tail) MTCT strategy. Two distinct questions are addressed:

In Maternal Health Comparison #1a, we compare women who were randomized to receive antepartum a triple ARV regimen (with no subsequent maternal ART) in the Antepartum Component with women randomized to receive a non-triple ARV regimen (ZDV + sdNVP + TRV tail) for preventing antepartum/intrapartum MTCT (with no subsequent maternal ART) in terms of the effect of antepartum ARV prophylaxis on long-term maternal health outcomes. This comparison will include both FF and BF women.

In Maternal Health Comparison #1b, we compare women who were randomized to receive triple ARV prophylaxis during BF in the Postpartum Component (with no triple ARV regimen during pregnancy or after BF cessation) with women who were randomized to infant NVP prophylaxis during BF in the Postpartum Component and therefore did *not* receive a postpartum triple ARV regimen during BF (or during pregnancy or after BF cessation) in terms of the effect of postpartum triple ARVs on long-term maternal health outcomes.

Thus, the analyses of Maternal Health Comparisons #1a and 1b seek to determine the long-term efficacy and safety of maternal triple ARV prophylaxis given to prevent MTCT relative to MTCT prevention strategies during pregnancy and BF, which do not involve a maternal triple ARV regimen.

Maternal Health Comparisons #2a and 2b address the effects of continuing a maternal triple ARV regimen beyond the time it is needed for prevention of MTCT. Two distinct questions are addressed:

Maternal Health Comparison #2a is comprised of women who participated in the Antepartum Component of PROMISE and were randomized to antepartum triple ARV prophylaxis. At delivery, these women (whether they intend to FF or BF) will be randomized to continue versus discontinue the triple ARV regimen, and we will assess the relative efficacy for maternal health of continuing vs. versus discontinuing the triple ARV regimen beyond the time it is needed for prevention of antepartum/intrapartum MTCT.

Maternal Health Comparison #2b is comprised of women who were randomized to postpartum triple ARV prophylaxis during BF as part of the Postpartum Component, and asks whether continuation of maternal HAART beyond cessation of risk for BF MTCT confers long-term benefits to mothers relative to discontinuing the triple ARV regimen upon cessation of risk of BF MTCT.

In a secondary analysis, the three sequential PROMISE randomizations will be used to form three comparison groups which correspond to the three WHO PMTCT options: Option A= antepartum ZDV + sdNVP + TRV tail and postpartum infant NVP prophylaxis; Option B= antepartum and postpartum maternal triple ARV prophylaxis; and Option B+ = maternal triple ARVs for life, regardless of CD4+ cell count). All three pairwise comparisons of these three groups will be conducted.

The Option A comparison group will consist of breastfeeding women who are randomized to receive ZDV + sdNVP +TRV tail during pregnancy and then randomized to discontinue the use of ARVs after the intrapartum period (n=611). The Option B comparison group will consist of breastfeeding women are randomized to receive triple ARV prophylaxis during pregnancy and breastfeeding, then are randomized to discontinue triple ARVs after breastfeeding cessation (n=746). The Option B+ comparison group will consist of breastfeeding women are randomized to receive triple ARV prophylaxis during pregnancy, breastfeeding, and after breastfeeding cessation (n=746). When comparing A vs. B or B+, the risk-time will start at study entry in 1077BA; when comparing B vs. B+, the risk-time will start at the time of randomization to continue or discontinue triple ARVs.

6.32 Primary and Secondary Outcome Measures (Maternal Health Component)

Note: The qualifying illnesses and conditions corresponding to the primary and secondary efficacy outcome measures below are listed in Appendix IV. Definitions of terms used follow:

- "AIDS-defining illness" refers to the WHO Clinical Stage 4 illnesses listed in Appendix IV.
- "HIV/AIDS-related event" refers to the WHO Clinical Stage 4 illnesses, pulmonary tuberculosis, and other serious bacterial infections listed in Appendix IV.
- "Other metabolic events" refers to diabetes mellitus, lipodystrophy and dyslipidemia as defined in Appendix IV.
- WHO Clinical Stage 2 and 3, cardiovascular, hepatic, and renal events, and other targeted medical conditions are listed in Appendix IV.

6.321 Primary Outcome Measures

- Composite endpoint of progression to AIDS-defining illness or death

6.322 Secondary Outcome Measures

- Death
- AIDS-defining illness
- Composite endpoint of progression to AIDS-defining illness, death, or a serious non-AIDS cardiovascular, hepatic, or renal event
- HIV/AIDS-related events
- Cardiovascular or other metabolic events
- Other targeted medical conditions
- Composite endpoint of HIV/AIDS-related event or death
- Composite endpoint of HIV/AIDS-related event or WHO Clinical Stage 2 or 3
- Composite endpoint of any condition outlined in Appendix IV or death
- Tuberculosis
- Toxicity: Grade 3 or greater laboratory results or signs and symptoms and selected Grade 2 hematologic, renal and hepatic laboratory results
- Viral resistance
- Self-reported adherence
- Quality of life
- Changes in plasma concentrations of inflammatory and thrombogenic markers
- Cost-Effectiveness

6.33 Randomization and Stratification (Maternal Health Component)

The Maternal Health Comparisons will be based on the PROMISE Antepartum, Postpartum and/or Maternal Health randomizations, as described below:

Maternal Health Comparison #1a:
As part of the Antepartum, Postpartum, and Maternal Health Components, approximately one half of the enrolled BF and FF women will be randomized to receive triple ARV prophylaxis or ZDV + sdNVP + TRV tail during pregnancy and then no maternal ARV regimen after delivery. The Antepartum Component randomization will be stratified by country and HBV status. The Postpartum Component randomization will be stratified by country and the antepartum intervention [triple ARV prophylaxis vs.

ZDV + sdNVP + TRV tail vs. IP only ZDV + sdNVP + TRV tail (late presenter) vs. none (late presenter)]. The Maternal Health Component randomization (FF women) will be stratified by country.

Cohort 1a Comparison Groups

Cohort 1a	Antepartum/ Intrapartum*	Breastfeeding/ Postpartum	Post Breastfeeding Cessation
**Arm 1**	Triple ARVs	No ARVs**	No Further Randomization
**Arm 2**	ZDV+sdNVP+TRV	No ARVs**	No Further Randomization

**Indicates time zero*
***Randomized to discontinue triple ARVs in 1077BM/1077FM or randomized to infant prophylaxis in 1077BP*

Maternal Health Comparison #1b:
As part of the Antepartum and Postpartum Component Randomizations (described above under Comparison #1a), approximately half of enrolled BF women will be randomized to receive ZDV + sdNVP + TRV tail during pregnancy and then either maternal triple ARV prophylaxis (with six weeks of infant NVP) or infant NVP prophylaxis(with no maternal ARV) during breastfeeding. Also, late-presenting BF women (and their infants) will be randomized in the Postpartum Component to receive either maternal triple ARV prophylaxis or infant NVP (with no maternal ARV) during BF. Half of the BF women who are on the triple ARV regimen upon cessation of risk for BF MTCT will be randomized to discontinue ARV regimen at that time as part of the Maternal Health Component (described below under Comparison #2b).

Cohort 1b Comparison Groups

Cohort 1b	Antepartum/ Intrapartum	Breastfeeding/ Postpartum*	Post Breastfeeding Cessation
**Arm 1**	ZDV+sdNVP+TRV or No ARV	Triple ARVs	No ARVs***
**Arm 2**	ZDV+sdNVP+TRV or No ARV	No ARVs**	No Further Randomization

**Indicates time zero*
***Randomized to discontinue triple ARVs in 1077BM or randomized to infant prophylaxis in 1077BP*
**** Randomized to discontinue triple ARVs in 1077BM*

Maternal Health Comparison #2a:
At delivery, in either the Maternal Health Component (FF women) or in the Postpartum Component (BF women), BF and FF women who were randomized to triple ARV prophylaxis in the Antepartum Component will be randomized in a 1:1 ratio to continue versus discontinue the triple ARV regimen postpartum, as described above under Comparison #1a.

Cohort 2a Comparison Groups

Cohort 2a	Antepartum/ Intrapartum	Breastfeeding/ Postpartum*	Post Breastfeeding Cessation
**Arm 1**	Triple ARVs	Triple ARVs	Triple ARVs
**Arm 2**	Triple ARVs	No ARVs**	No Further Randomization

**Indicates time zero*
***Randomized to discontinue triple ARVs in 1077BM/1077FM or randomized to infant prophylaxis in 1077BP*

Maternal Health Comparison #2b:
Upon cessation of risk for BF MTCT, in the Maternal Health Component (BF women who were randomized to triple ARV prophylaxis in the Postpartum Component), women who had been receiving triple ARV prophylaxis during BF will be randomized in a 1:1 ratio to continue versus discontinue the triple ARV regimen. The randomization will be stratified by country, infant age at randomization (< 9 months, 9-12, > 12 months), and the antepartum intervention [triple ARV prophylaxis vs. ZDV + sdNVP + TRV tail vs. IP only ZDV + sdNVP + TRV tail (late presenter) vs. none (late presenter)].

Cohort 2b Comparison Groups

Cohort 2b	Antepartum/ Intrapartum	Breastfeeding/ Postpartum	Post Breastfeeding Cessation*
**Arm 1**	Triple ARVs or ZDV+sdNVP+TRV or No ARV	Triple ARVs	Triple ARVs
**Arm 2**	Triple ARVs or ZDV+sdNVP+TRV or No ARV	Triple ARVs	No ARVs**

** Indicates time zero*
***** Randomized to discontinue triple ARVs in 1077BM***

Secondary Comparison of WHO PMTCT Options:

As part of the Antepartum and Postpartum Component Randomizations (described above under Comparison #1a), BF women will be randomized to receive (Option A): ZDV + sdNVP + TRV tail during pregnancy and then infant NVP prophylaxis (with no maternal ARV) during breastfeeding; or (Option B) triple ARV prophylaxis during pregnancy and breastfeeding, and no ARVs after breastfeeding cessation; or (Option B+) triple ARV prophylaxis during pregnancy, breastfeeding, and beyond.

WHO PMTCT Option Comparison Groups

WHO PMTCT Option	Antepartum/ Intrapartum	Breastfeeding/ Postpartum	Post-Breastfeeding Cessation
**Option A**	ZDV+sdNVP+TRV	No ARVs*	No Further Randomization
**Option B**	Triple ARVs	Triple ARV	No ARVs**
**Option B+**	Triple ARVs	Triple ARVs	Triple ARVs***

** Randomized to infant prophylaxis in 1077BP*
*** Randomized to discontinue triple ARVs in 1077BM*
**** Randomized to continue triple ARVs in 1077BM*

6.34 <u>Sample Size and Duration of Follow-Up</u> (Maternal Health Component)

The sample size available for each primary Maternal Health Comparison is determined by the number of women who were randomized to the relevant arms of the Antepartum and/or Postpartum Component to address the PMTCT objectives. Hence, the sample size calculations below indicate the effects on maternal health outcome measures that could be detected with 90% power based on the anticipated number of women and duration of follow-up for each Maternal Health comparison.

Maternal Health Comparison #1a:
Approximately 4,400 subjects will be randomized to either triple ARV (2,581) prophylaxis or ZDV+sdNVP +TRV tail (1,819) as a part of the Antepartum Component. Women who are randomized to triple ARVs after delivery will be censored for this analysis; however, they will represent a very small portion of the overall risk time, and thus they will be ignored for the power calculation. Assuming a 5% annual loss to follow-up rate, it is anticipated that approximately 1,478 evaluable BF and 614 evaluable FF women will have been randomized to one of the Antepartum Component arms and to no additional ARV use following birth (either discontinuing ARV use in FF women or randomized to infant NVP prophylaxis in BF women), with approximately 1,122 of these receiving triple ARV prophylaxis and 970 receiving ZDV + sdNVP + TRV tail during pregnancy, and followed for an average of 3 years. If the 3-year cumulative AIDS/death event rate among women who received short course ZDV + sdNVP + TRV tail during pregnancy is 10%, we will have approximately 90% power to detect a 14.6% 3-year AIDS/death event rate in women who received triple ARV prophylaxis during pregnancy, based on a 2-sided Type I error of 5%.

Maternal Health Comparison #1b:
Assuming a 5% annual loss to follow-up rate, it is anticipated that there will be approximately 1,231 evaluable women who receive triple ARV prophylaxis only during BF and 1,231 evaluable matching women with no ARV prophylaxis during (or before) BF. Of the former, the early-presenting (women enrolled following the Antepartum Component) and late-presenting BF women randomized to receive triple ARV prophylaxis only postpartum and post-BF cessation will be censored at the time of BF cessation, leaving an effective sample size of 820 women followed for 3 years. This would provide approximately 90% power to detect an increase in the cumulative 3-year AIDS/death rate from 10% to 14.9%, based on a 2-sided Type I error of 5%.

Maternal Health Comparison #2a:
Allowing for a 5% annual loss to follow-up rate, it is anticipated that approximately 1,734 evaluable BF and 510 evaluable FF women will have been randomized to the triple ARV prophylaxis arm of the Antepartum Component and will agree to be randomized to continue the triple ARV regimen (n=1,122) or discontinue the triple ARV regimen (n=1,122) after their babies are born, and followed for an average of 3 years. Of the approximately 867 who are BF and randomized to postpartum triple ARV prophylaxis, 50% will be censored for the purposes of this analysis when they are randomized to discontinue the triple ARV regimen post-BF cessation (at approximately 1 year post birth). This would result in an effective sample size in the postpartum triple ARV regimen arm of approximately 578 women followed for an average of 3 years. If the 3-year cumulative AIDS/death event rate among women that discontinue the triple ARV regimen at birth is 10%, we will have approximately 90% power to detect a reduction in the 3-year cumulative AIDS/death event rate to 6.1% in women who continue the triple ARV regimen, based on a 2-sided Type I error of 5%.

Maternal Health Comparison #2b:
Assuming a 5% annual loss to follow-up rate, approximately 1,492 evaluable early-presenters and 636 evaluable late-presenters will have received triple ARV prophylaxis during BF and be randomized to continue (n=1,064) versus discontinue (n=1,064) the triple ARV regimen after their infants cease BF, with an average follow-up period of 2 years. Assuming a 6.67% 2-year rate of AIDS/death in women that discontinue the triple ARV regimen at BF cessation, there will be approximately 90% power to detect a reduction in the 2-year rate to 3.6% in women who continue the triple ARV regimen, based on a 2-sided Type I error of 5%.

6.35 Monitoring (Maternal Health Component)

This section describes the specific monitoring plan for the Maternal Health Component. Please also refer to Appendix III, which describes general considerations for the interim monitoring of all Components of PROMISE. A detailed plan for interim analyses will be developed before such analyses are undertaken.

The protocol team will review the status of the Maternal Health Component regularly. This review will examine reports on numbers of women eligible for each comparison, characteristics of participants, retention, data and specimen completeness, and adverse events (including deaths). These reports will present overall results that are pooled across all randomized groups and not broken out according to groups. A full monitoring plan with specific details concerning the content and scheduling of these reports will be disseminated in a separate document before the study opens to accrual.

The team will regularly monitor two types of treatment non-adherence at the site level: the proportion of women randomized to continue the triple ARV regimen who prematurely discontinue the regimen, and the proportion of women randomized to discontinue the triple ARV regimen who actually re-initiate the regimen prior to meeting the CD4 cell count threshold for initiating triple ARV treatment (HAART). The study norms are that both proportions should be no greater than 10%. Appropriate remedial actions will be developed by the protocol team for any site that fails to meet either norm.

The Maternal Health Component also will be monitored by an NIAID-sponsored Data and Safety Monitoring Board (DSMB). Interim analyses focusing on safety, study logistics, and the accuracy of sample size assumptions will be reviewed at least annually starting within 12 months after the first woman is randomized. The reported adherence rates and norms described above as well as the overall and site-specific adherence rates will be included in each closed DSMB report (both pooled and by study arm). In the open DSMB report, the pooled results will be presented. These results will be discussed with the PROMISE team.

Interim efficacy analyses will be performed annually once at least 25% of the information on the primary efficacy outcome measure is available. Under the Antepartum and Postpartum Component accrual assumptions, interim efficacy analyses are projected to be performed approximately 2, 3, 4, and 5 years after the first woman is randomized, with the following anticipated information rates for the primary Maternal Health comparisons of PROMISE:

	**+2 years**	**+3 years**	**+4 years**	**+5 years**
**Comparison 1a**	33%	50%	67%	83%
**Comparison 1b**	33%	50%	67%	83%
**Comparison 2a**	33%	50%	67%	83%
**Comparison 2b**	27%	45%	66%	81%

The interim efficacy analysis schedule may be modified if accrual assumptions turn out to be inaccurate or if recommended by the DSMB.

Efficacy analyses for these comparisons will be based on group-sequential repeated confidence intervals (14), using the Lan-DeMets approach with an O'Brien-Fleming spending function. While all comparisons address the efficacy of extending triple ARV use, they represent different scientific questions and thus will be analyzed separately.

The interpretation of Comparisons 1a and 1b, which assess the benefits to mothers of maternal triple ARV prophylaxis during pregnancy or BF, will need to be balanced with the relative efficacy of triple ARV

regimens versus the non-triple ARV prophylaxis regimen (ZDV + sdNVP + TRV tail) for preventing antepartum/intrapartum or BF MTCT. Safety of the maternal triple ARV regimen will be assessed by evaluation of both drug-related toxicities and the analyses of Comparisons 1a and 1b, which assess the efficacy of triple ARV use with respect to AIDS/death relative to the less complex ZDV + sdNVP + TRV tail regimen.

Criteria for recommending the stopping of the Postpartum Component infant NVP prophylaxis arm based on analysis of the Comparison 2a results would normally require all of the following: (a) CIs for the continue: discontinue triple ARV use hazard ratio for time to AIDS/death that falls entirely below 1, (b) lack of evidence that the efficacy benefit is transient (less than 2 years), (c) the absence of any evidence supporting the superior efficacy of infant NVP prophylaxis, relative to maternal triple ARV prophylaxis, for the prevention of BF MTCT, and (d) an acceptable maternal and infant safety profile for the maternal triple ARV regimen.

With respect to Comparison 2b, criteria for recommending the stopping of the post-BF cessation component of PROMISE in favor of the continue-triple ARV use arm would normally require all of the following: (a) CIs for the continue: discontinue triple ARV use hazard ratio for AIDS/death endpoint which fall entirely below 1, (b) lack of evidence that the efficacy benefit is transient (less than 2 years), and (c) an acceptable maternal safety profile for continued maternal triple ARV use.

In addition, for Comparisons 2a and 2b, consideration would be given to the consistency of effects seen on the primary endpoint with those seen in the secondary endpoints. Except for the endpoint of death from any cause, a significant difference between the "continue the triple ARV regimen" arm versus "discontinue the triple ARV regimen" arm with respect to a secondary endpoint, in the absence of strong evidence of a difference in the primary endpoint, would not be grounds for stopping the trial. On the other hand, strong evidence of a difference in the primary endpoint favoring one arm, but with evidence favoring the other arm with an important secondary endpoint, might support the continuation of the trial.

6.36 Analyses (Maternal Health Component)

Full details of the proposed analyses will be described in a statistical analysis plan that will be developed once enrollment to the study begins and prior to the commencement of analyses for the first review by the DSMB. Hence, here we limit the description of the proposed analyses to those for the primary efficacy endpoint.

Analyses will use the principle of intention-to-treat. Specifically:

For Comparison 1a, time zero is randomization to the AP Component. All women randomized to the AP/IP component will be followed (for analysis purposes) for the duration of the trial, except for the following: (i) FF women randomized to AP/IP triple ARV prophylaxis and randomized to continue the triple ARV regimen postnatally will be censored at the time of the postnatal randomization; (ii) BF women randomized to AP/IP ZDV + sdNVP + TRV tail and randomized to PP maternal triple ARV prophylaxis will be censored at the time of the PP randomization, and (iii) BF women randomized to AP/IP triple ARV prophylaxis and randomized to PP triple ARV prophylaxis will be censored at the time of the PP randomization. It is recognized that this comparison could be biased if a considerable number of BF women enrolled in the AP/IP component decline participation in the PP randomization, or if a considerable number of FF women decline participation in the Maternal Health Component, and if women who participate differ from those who do not, as this could induce a type of dependent censoring. However, we anticipate very few such women.

For Comparison 1b, time zero is randomization to the Postpartum Component. The durations of follow-up for analysis purposes are as follows (all BF women): for women randomized to the PP infant NVP prophylaxis arm, follow-up will be for the duration of the trial. For women randomized to the PP maternal triple ARV prophylaxis arm, follow-up will be for the duration of the trial except for those who are randomized to continue the triple ARV regimen following BF cessation. This latter group will be censored at the time of this randomization. This comparison could be biased if a considerable number of BF women randomized to PP maternal triple ARV prophylaxis do not participate in the post BF cessation randomization, and if women who participate differ from those who do not, as this could induce a type of dependent censoring. However, we do not expect that this will occur.

For Comparison 2a, time zero is the post-birth randomization for both FF and BF women. The durations of follow-up for analysis purposes are as follows: (i) until the end of the trial for FF women randomized to AP/IP triple ARV prophylaxis and who participate in the post-birth Maternal Health randomization, and (ii) until the end of the trial for BF women randomized to AP/IP triple ARV prophylaxis who participate in the PP randomization, except for those who discontinue triple ARV use upon cessation of BF (either because randomized to discontinue the ARV regimen, or decline or are ineligible for the post BF cessation randomization); the latter group of BF women will be censored at the time of the post-BF cessation randomization. For this comparison, it is recognized that if the women who discontinue the triple ARV regimen upon BF cessation differ in health status at that time from those who continue, the censoring of outcomes might be informative and therefore bias the comparisons. However, it is anticipated that the large majority of those that are randomized to PP maternal triple ARV prophylaxis will participate in the post BF cessation randomization.

For Comparison 2b, time zero is the post-BF cessation randomization. All BF women who participate in the post BF cessation randomization will be followed (for analysis purposes) for the duration of the trial.

The results for comparisons 2a and 2b would become complicated if, during the conduct of PROMISE, the national criteria for initiating HAART increase to a higher CD4+ level (for example, from 350 to 500 CD4+ cells). Such a change would make the strategies of continuing versus discontinuing HAART more similar. If such a change occurred early during the PROMISE trial, the interpretation of the arms would be clear, but there might not be adequate power to detect a difference. On the other hand, if the change occurs mid-way through the trial, the comparator arm to continuing HAART becomes harder to interpret because two policies for re-initiating HAART will contribute to the results. If such changes in national criteria for initiating HAART occur, the analysis plan will be modified accordingly depending on the specifics of the change and the timing during the trial.

The primary analyses for objectives 1a and 2a will be stratified by AP/IP intended feeding category (FF vs. BF), and the primary analyses of objectives 1b and 2b will be stratified by presentation status (early presenter vs. late presenter) at the time of the postpartum randomization. The comparisons will be based on log rank tests for testing and Cox regression models for estimating treatment effect sizes. In light of the conservative spending function that will be used in interim efficacy analyses, unadjusted point estimates, p-values and 95% confidence intervals will be used to summarize the results in the final analysis. Secondary efficacy endpoints will be analyzed similarly. Secondary efficacy analyses of the primary endpoint will include Cox regression models adjusted for AP/IP randomization stratification factors (Comparisons 1a and 2a) and PP randomization stratification factors (Comparisons 1b and 2b), and for interactions between treatment group and the strata used in the primary efficacy analysis of the primary endpoint. Although the AP/IP and PP randomizations were stratified by country, maternal background mortality rates can vary substantially at different study sites within the same country (e.g., Pune vs. Chennai in India); consideration will be given to performing additional secondary efficacy analyses of the primary endpoint in which the Cox regression models described above are adjusted for study site (or groups of sites with similar background rates) instead of country, and for interactions

between treatment group and study site (or groups of study sites with similar background rates), recognizing that the analysis will have limited power to detect interactions.

6.4 Statistical References

(1) Dunn DT, Brandt CD, Krivine A, et al. The sensitivity of HIV-1 DNA polymerase chain reaction in the neonatal period and the relative contributions of intra-uterine and intra-partum transmission. AIDS 1995; 9 (9):F7-11.

(2) Personal Communication, Kim

(3) Balasubramanian R, Lagakos SW. Estimation of the timing of perinatal transmission of HIV. Biometrics 2001; 57(4):1048-1058.

(4) Balasubramanian R, Lagakos SW. Estimation of a failure time distribution based on imperfect diagnostic tests. Biometrika 2003; 90:171-182

(5) Tonwe-Gold B, Ekouevi DK,Viho I, et al. Antiretroviral treatment and prevention of peripartum and postnatal HIV transmission in West Africa: evaluation of a two–tiered approach. PLoS Med 2007; 4: e257.

(6) Kilewo C, Karlsson K, Ngarina M, et al. Prevention of mother to child transmission of HIV through breastfeeding by treating mothers prophylactically with triple antiretroviral therapy in Dar es Salaam, Tanzania – the MITRA-PLUS study. 4th International AIDS Society Conference on HIV Pathogenesis, Treatment and Prevention. Sydney Australia, July 2007; Abstract TuAX101).

(7) Kilewo C, Karlsson K, Massawe A, et al. Prevention of mother-to-child transmission of HIV through breast-feeding by treating infants prophylactically with lamivudine in Dar es Salaam, Tanzania: the Mitra Study. JAIDS 2008 Jul 1;48 (3):237-40.

(8) Lallemant M, Jourdain G, Le Coeur S, et al. Single-dose perinatal nevirapine plus standard zidovudine to prevent mother to child transmission of HIV-1 in Thailand. N Eng J Med 2004:351:217-28.

(9) European Collaborative Study. Increased risk of adverse pregnancy outcomes in HIV-infected women treated with highly active antiretroviral therapy in Europe. AIDS 2004: 18:2337-9.

(10) Coffie P, Becquet R, Tonwe-Gold B et al. Low birth weight is associated with maternal nevirapine based antiretroviral therapy in Abidjan, Cote d'Ivoire: the DITRAME Plus project and MTCT-Plus initiative (2001-2007). 4th Dominique Dormont International Conference. Paris, France, December 2007; Abstract 19.

(11) Six Week Extended-Dose Nevirapine (SWEN) Study Team. Extended-dose nevirapine to 6 weeks of age for infants to prevent HIV transmission via breastfeeding in Ethiopia, India, and Uganda: an analysis of three randomised controlled trials. Lancet 2008;372 (9635):300-12.

(12) Kumwenda NI, Hoover DR, Mofenson LM, et al. Extended antiretroviral prophylaxis to reduce breast-milk HIV transmission. N Engl J Med. 2008;359(2):119-29.

(13) The Breastfeeding and HIV International Transmission Study Group. Late postnatal transmission of HIV in breast-fed children: an independent patient data meta-analysis. J Infect Dis 2004;189:2154-66.

(14) Jennison C, Turnbull BW. Group sequential methods with applications to clinical trials. Boca Raton, FL: CRC Press, 1999.

7.0 REQUIREMENTS FOR CASE REPORT FORM RECORDING AND SERIOUS ADVERSE EVENT REPORTING

The DAIDS Table for Grading the Severity of Adult and Pediatric Adverse Events, Version 1.0, dated December 2004 (with Clarification dated August 2009), (which can be at the following website: http://rsc.tech-res.com) must be followed, with the exception of axillary-measured fever and malnutrition/failure to thrive, for which supplementary grading scales for infants are included below in Section 7.2.

7.1 Case Report Form (CRF) Recording Requirements

Signs and Symptoms
Regardless of severity grade, all signs and symptoms occurring ≤ 30 days before study entry must be recorded on the CRFs. Post-entry, all Grade 3 or higher signs and symptoms, and signs and symptoms of any grade that lead to a change in treatment must be recorded on the CRFs.

All Grade 4 signs and symptoms and any grade signs and symptoms that lead to a change in treatment will be further evaluated and may require additional supporting information to assess the relationship to study drugs. The additional evaluation(s) must be recorded on the appropriate CRF.

Laboratory Evaluations
At screening, entry and post-entry all laboratory values must be recorded on the CRFs.

All Grade 3 or higher creatinine, AST or ALT values, all Grade 4 laboratory values, and any Grade laboratory value that leads to a change in treatment will be further evaluated and may require additional supporting information to assess the relationship to study drugs. The additional evaluation(s) must be recorded on the appropriate CRF.

Diagnoses
For mothers: At entry, all diagnoses identified by the Pediatric/Maternal Diagnoses criteria during the current pregnancy are to be recorded. After entry, all diagnoses identified since the last study visit are to be recorded on the CRFs.

For infants: All diagnoses identified by the Pediatric/Maternal Diagnoses criteria are to be recorded on the CRFs.

For mothers only: With the exception of WHO Stage 2 Clinical Events, the diagnoses listed in Appendix IV (Maternal Endpoint Diagnoses) will be further evaluated at all post-entry visits and may require additional supporting information to assess the relationship to study drugs and for study endpoint verification. The additional evaluation(s) must be recorded on the appropriate CRF.

The reporting requirements specified above apply for the full duration of study participation.

Note: The Pediatric/Maternal Diagnoses can be found in the appropriate appendix (as directed on the relevant diagnosis CRF) on the IMPAACT Data Management Center website: www.fstrf.org.

7.2 Adverse Event Reporting to DAIDS

Requirements, definitions and methods for expedited reporting of Adverse Events (AEs) are outlined in Version 2.0 of the Manual for Expedited Reporting of Adverse Events to DAIDS (referred to as the DAIDS EAE Reporting Manual), dated January 2010, which is available on the RSC website at http://rsc.tech-res.com and in the study MOP.

The DAERS internet-based reporting system should be used for expedited AE reporting to DAIDS. For questions about DAERS, please contact DAIDS-ES at DAIDS-ESSupport@niaid.nih.gov or from within the DAERS application itself.

Sites that are unable to use DAERS will submit expedited AEs by documenting the information on the current DAIDS EAE Reporting Form available on the RSC website: http://rsc.tech-res.com. For questions about EAE reporting, please contact the RSC.

7.21 Reporting Requirements for this Study

The "all SAE" Reporting Category as defined in Version 2.0 of the DAIDS EAE Reporting Manual will be used. In addition, all fetal deaths occurring at ≥ 20 weeks gestation (in primary pregnancies and new pregnancies) in women taking study-supplied drugs during the pregnancy must be reported in an expedited manner to DAIDS. Also, all immune reconstitution inflammatory syndrome (IRIS) events that meet the criteria (are serious adverse events) must be reported in an expedited manner to DAIDS, as an exception to the reporting requirements specified in Section 4.2 of the DAIDS EAE Reporting Manual. Relationship to study-supplied study drug will be defined as specified in the DAIDS EAE Reporting Manual, Version 2.0.

The study agents that must be considered in determining the relationships to AEs for EAE reporting in each component of PROMISE are:

- *Antepartum Component:* For mothers and infants, the study agents for which relationship assessments are required are *study-supplied* zidovudine, lamivudine, zidovudine-lamivudine, tenofovir disoproxil fumarate, emtricitabine-tenofovir disoproxil fumarate, lopinavir-ritonavir, ritonavir, atazanavir, didanosine, efavirenz, tenofovir disoproxil fumarate -emtricitabine-rilpivirine and nevirapine.
- *Late Presenters Registration:* For mothers and infants, the study agents for which relationship assessments are required are *study-supplied* zidovudine, nevirapine, lamivudine and emtricitabine-TDF.
- *Postpartum Component:* For both mothers and infants randomized to Step 1 Arm A (maternal prophylaxis with six weeks of infant prophylaxis), the study agents for which relationship assessments are required are *study-supplied* zidovudine, lamivudine, zidovudine-lamivudine, tenofovir disoproxil fumarate, emtricitabine-tenofovir disoproxil fumarate, lopinavir-ritonavir, ritonavir, atazanavir, didanosine, efavirenz and tenofovir disoproxil fumarate-emtricitabine-rilpivirine; for infants only, *study-supplied* nevirapine also requires relationship assessment. For infants randomized to Step 1 Arm B (infant prophylaxis only), the study agents for which relationship assessments are required are *study-supplied* nevirapine and lamivudine; for mothers of infants randomized to Step 1 Arm B, there is no study-supplied drug dosing.
- *Maternal Health Component:* For mothers, the study agents for which relationship assessments are required are *study-supplied* zidovudine, lamivudine, zidovudine-lamivudine, tenofovir disoproxil fumarate, emtricitabine-tenofovir disoproxil fumarate, lopinavir-ritonavir, ritonavir, atazanavir, didanosine, efavirenz and tenofovir disoproxil fumarate- emtricitabine-rilpivirine. Infants will not be enrolled in the Maternal Health Component, so there is no study-supplied infant drug dosing as part of this component; however, exposure to the maternal study agents via breastfeeding may occur.

7.22 Grading Severity of Events

The current Division of AIDS Table for Grading the Severity of Adult and Pediatric Adverse Events, Version 1.0, dated December 2004 (with Clarification dated August 2009) must be used and is available on the RSC website at http://rsc.tech-res.com and in the study MOP.

In addition, for the purposes of expedited adverse events reporting, the severity of malnutrition and axillary-measured fever will be graded as specified below:

Malnutrition/failure to thrive:

SEVERITY GRADE	1	2	3	4
Weight-for-age	Crossing of < 2 percentiles downward on the WHO weight- for age Growth Standards	Failure to gain weight for ≥ 3 months or weight-for-age measurement crosses 2 major percentiles downward on the WHO Growth Standards	Weight-for-age measurement less than 80% and 70% or more of the median WHO reference (80% > WFA ≥ 70%)	Weight-for-age measurement less than 70% of the median WHO reference (WFA < 70%)<br>*AND/OR*<br>Bilateral pitting edema of nutritional origin
Condition according to Pediatric/Maternal Diagnoses	Growth Faltering	Failure to Thrive (FTT)	Moderate Acute Malnutrition	Severe Acute Malnutrition
Considered an SAE	No	No	Yes	Yes

Note: Malnutrition should be considered the most severe of the categories achieved.

Fever (axillary-measured):

Grade 1: 37.1 - 38.0°C
Grade 2: 38.1 - 38.7°C
Grade 3: 38.8 - 39.9°C
Grade 4: > 39.9°C

7.23 Expedited AE Reporting Period

The expedited AE reporting period for this study is the entire duration for which the subject is on or exposed to study-supplied drug and for 30 days thereafter. After this and while a participant is still in study follow-up, only suspected, unexpected, serious adverse drug reactions (SUSARs, as defined in the DAIDS EAE Reporting Manual) and fetal deaths occurring at or after 20 weeks gestation (in primary pregnancies and in new pregnancies) that are judged by the site investigator to be related to study-supplied drug must be reported in an expedited manner to DAIDS. (IRIS events are not reportable SUSARs because they are expected.)

After the end of study follow-up for a participant, only SUSARs will be reported to DAIDS in an expedited manner if the study staff become aware of the events on a passive basis (from publicly available information).

8.0 HUMAN SUBJECTS CONSIDERATIONS

8.1 IRB/EC Review and Sample Informed Consent

This protocol, the informed consent documents for each of the three main components (AP, PP, and MH), for Late Presenters, for women who get pregnant again while on study drug (Appendix V) and for specimen storage (Appendix VI) and any subsequent modifications to them must be reviewed and approved by the IRB(s) or Ethics Committees (ECs) responsible for oversight of the study. Written informed consent must be obtained from the women for their own participation and that of their infant. The informed consent form and process will describe the purpose of the study, the procedures to be followed and the risks and benefits of participation. A copy of the consent forms will be given to the subject.

Each component of 1077BF has an accompanying consent form. Should modification or amendment of the informed consent document occur during the conduct of the study, only women and their infants currently on that component will be required to re-consent using the modified or amended consent form, unless otherwise directed by the IRBs/ECs overseeing the study. If the woman and her infant have already completed the component of the study with the modified or amended consent form, they will not be required to re-consent, unless otherwise directed by the IRBs/ECs. In addition to the consent forms for enrollment or registration to each study component, sample consent forms for continuation of study drug in women who are get pregnant again while on study and for long term storage of biological specimens remaining after trial-specific assays are completed are included in Appendix V and Appendix VI, respectively. The informed consent form documenting each woman's willingness or unwillingness to have her own and her child's leftover specimens stored must be completed for each enrolled mother-infant pair. This consent form may be completed any time during study participation, though ideally as soon after entry as possible and within three months.

Should the mother of an enrolled infant die or no longer be available for any other reason, study drug (if being given at the time) should be stopped immediately, and no further study-specific evaluations or assessments can be performed until consent for the infant's continued participation in the study is obtained from a legally authorized individual, as defined locally. However, sites should continue to provide care for the infant as needed and appropriate (outside of the study). Prior to study initiation, sites will be asked to obtain documentation on local laws/regulations governing guardianship as well as their IRB/EC's interpretation of those laws in the context of research in infants and children and to develop a plan for handling these situations (if not already in place). If appropriate, the plan may also address identification of persons other than the mother or legal guardian who are allowed to bring the child for study follow-visits (e.g., a relative); however, due to concerns about confidentiality and ability to accurately identify an infant brought by someone other than the mother, such a plan would need to be shared with and possibly approved by the local IRBs/ECs prior to implementation, according to their individual requirements.

Sites will be required to submit a plan for post-study care and treatment for women and infants as part of the Site Implementation Plan.

8.2 Subject Confidentiality

All laboratory specimens, evaluation forms, reports, and other records that are transferred or transmitted off-site for processing will be identified only by a coded number to maintain subject confidentiality. All records will be kept in a secured area with access limited to authorized personnel only. All computer entry and networking programs will be performed with coded numbers only. The use of participant identifiers on study records must comply with the DAIDS SOPs for Source Documentation and Essential

Documents. Clinical information will not be released without written permission of the subject, except as necessary for monitoring by the US FDA, the Office for Human Research Protections (OHRP), the study sponsors (NIAID and NICHD) or their authorized agents, representatives or agents of the IMPAACT leadership (e.g., staff from the operations center, data management center and network lab), the IRBs/ECs, local regulatory authorities or the pharmaceutical co-sponsors.

8.3 Study Discontinuation

The study may be discontinued at any time by the IMPAACT leadership, NIAID, NICHD, and the Office for Human Research Protections (OHRP), the US FDA, the pharmaceutical suppliers, an in-country national health or regulatory agency and/or the IRBs/ECs as part of their duties to ensure that research subjects are protected.

APPENDIX IA
MATERNAL SCHEDULES OF EVALUATIONS, INCLUDING LATE-PRESENTERS AND OBSERVATIOAL WOMEN
(1077BA (ALL STEPS), 1077BP (ALL STEPS) AND 1077BL)

TIME BASED VISITS													1077BP ON STUDY			1077BA /BP OBS	EVENT BASED VISITS				
ANTEPARTUM (1077BA)								POSTPARTUM (BA, BP, BL)					FROM WK 50			FROM WK 50	ANTEPARTUM OR POSTPARTUM				
	BA SCREEN[a]	BA ENTRY[b]	WK 2[c]	WK 4[c]	WK 8[c]	WK 12 & Q4 WKS UNTIL L/D[c]	L/D[d] (LP Entry)	WK 1[g] (BP Entry)	WK 6[ij]	WK 14	WK 26	WK 38	WK 50	WK 62	WK 74	ALL WOMEN (STEPS 1, 2, 3)	EVENT DRIVEN VISIT[k]	PREMATURE D/C OF STUDY DRUG[l]	EARLY D/C or END of STUDY[m]	STEP CHANGE ENTRY[n]	STEP CHANGE WK 4[o]
CLINICAL EVALUATIONS																					
Informed Consent	X (BA)	X (BA)					X[e] (BL)	X[g] (BP)													
Documentation of HIV Infection[1]	X [3mL]						X[e] [3mL]														
History[2]	X	X					X[e]														
Interval Hx, Signs/Sx[3]			X	X	X	X	X[f]	X	X	X	X	X	X	X	X	q12 wks	X	X	X	X	X
Physical Exam[4]	X	X	X	X	X	X	X	X	X	X	X	X	X	X	X	q12 wks	X	X	X	X	X
WHO Clinical Staging	X							X			X		X		X	q24 wks		X	X	X	
Adherence Interview[5]			X		X	X (q8 wks)		X	X	X	X		X		X	q24 wks	X	X	X	X	X
QOL/ Resource Use Interview			X			X (Wk 12 only)				X	X		X		X	q24 wks		X	X	X	
Food Insecurity Questionnaire									X		X	X	X	X	X	Wks 50 & 98					
LABORATORY EVALUATIONS																					
Hep B Surface Antigen	2mL						2mL[e]														
CBC[6]	3mL	3mL		3mL	3mL	3mL (q8 wks)	3mL	3mL	3mL	3mL	3mL	3mL	3mL	3mL	3mL	3mL (q12 wks)	3mL	3mL	3mL	3mL	3mL
Chemistries[7]	2mL	2mL		2mL	2mL	2mL (q8 wks)	2mL	2mL	2mL	2mL	2mL	2mL (Hep B+ only)	2mL		2mL	2mL (q24 wks)	2mL	2mL	2mL	2mL	2mL
Pregnancy Test										Urine (5mL) or serum (1mL blood in SST or NON tube) test is acceptable. For women on EFV, required at every visit while on EFV and through 12 weeks after stopping EFV. Otherwise, to be done only when pregnancy is suspected or when considered clinically indicated by the study site clinician.											

	TIME BASED VISITS												1077BP ON STUDY			1077BA /BP OBS	EVENT BASED VISITS				
	ANTEPARTUM (1077BA)							POSTPARTUM (BA, BP, BL)					FROM WK 50			FROM WK 50	ANTEPARTUM OR POSTPARTUM				
	BA SCREEN [a]	BA ENTRY [b]	WK 2 [c]	WK 4 [c]	WK 8 [c]	WK 12 & Q4 WKS UNTIL L/D [c]	L/D [d] (LP Entry)	WK 1 [g] (BP Entry)	WK 6 [i,j]	WK 14	WK 26	WK 38	WK 50	WK 62	WK 74	ALL WOMEN (STEPS 1, 2, 3)	EVENT DRIVEN VISIT [k]	PREMATURE D/C OF STUDY DRUG [l]	EARLY D/C or END of STUDY [m]	STEP CHANGE ENTRY [n]	STEP CHANGE WK 4 [o]
VIROLOGY																					
HIV-1 RNA PCR[8]		6mL		6mL			6mL[h]	6mL[h]	6mL	6mL	6mL		6mL		6mL	6mL (q24 wks)	6mL	6mL	6mL	6mL	6mL
Stored EDTA Plasma, DBS (All women)[9]		10mL		10mL	10mL		10mL	10mL	10mL	10mL	10mL		10mL		10mL	10mL (q24 wks)	10mL	10mL	10mL	10mL	10mL
Additional Stored Plasma (HBsAg + Women only)[10]		6mL		2mL	2mL	2mL	6mL		4mL		4mL		6mL			6mL (q48 wks)	6mL	6mL	6mL	6mL	
Stored Breast Milk (during BF only)[11]								20mL	20mL	20mL	20mL		20mL		20mL	20mL (q24 wks)	20mL	20mL	20mL	20mL	20mL
IMMUNOLOGY																					
CD4 and CD8 Lymphocyte Percentage and Absolute Count[12]	3mL					3mL (Wk 12 only)	3mL[i]	3mL[i]		3mL	3mL	3mL	3mL	3mL	3mL	3mL (q12 wks)	3mL	3mL	3mL	3mL	
TOTAL BLOOD VOLUME (higher volume for HBsAg+ women)	10-13mL	21-27mL	0mL	21-23mL	15-17mL	2-10mL	15–30mL	15-24mL	21-25mL	24-25mL	24-29mL	6-9mL	24-31mL	6-7mL	24-25mL	6-31mL	24-31mL	24-31mL	24-31mL	24-31mL	21-22mL
HAIR COLLECTION																					
Hair Collection Stored Hair (during BF only)[13]								X	X	X	X	X	X	X	X				X		

a. Women may be screened starting at 10 weeks of gestation.
b. Women may be enrolled starting at 14 weeks of gestation.
c. One or more of the antepartum visits specified above will not done if a woman has already delivered by these timepoints.
d. L/D visit can be completed up to 5 days postpartum.
e. Required for late-presenting women only.

f. Women enrolled in the Antepartum Component (1077BA) only.
g. Week 1 visit can occur between 6 and 14 days postpartum. This visit is the 1077BP Entry visit. Informed consent for 1077BP must be obtained prior to entry.
h. HIV RNA PCR specimen collection is required at the Labor and Delivery Visit; if not done at the Labor and Delivery obtain a specimen at the week 1 visit.
i. The specimen for CD4 and CD8 Lymphocyte Percentage and Absolute Count may be obtained at the Labor and Delivery OR Week 1 visit. Specimen collection for CD4 and CD8 counts is not recommended during labor or within the first 24 hours postpartum
j. Late-presenting women who are NOT enrolled in 1077BP should complete evaluations for "Early D/C or End of Study" visit at Week 6. If a woman is Hep B positive it is not necessary to collected the additional stored plasma
k. Event driven visits should be performed for the following reasons:
   - Confirmation of infant HIV infection
   - Confirmation of immunologic failure
   - Confirmation of virologic failure
   - Discontinuation of all triple ARV regimens due to toxicity
   - Clinically significant event suggestive of acute exacerbation of Hepatitis B, including any grade 2 or higher liver function test result (symptomatic or asymptomatic) and/or any of the following signs/symptoms (regardless of liver function test results): scleral icterus, jaundice, petechiae, otherwise unexplained abdominal pain, abdominal swelling, lower extremity swelling, fever occurring with any of these signs/symptoms (HBsAg+ women only)

   See the study MOP for more information on the required timing of these visits.

   For event driven visits conducted for clinically significant events suggestive of acute exacerbation of Hepatitis B:
   - All evaluations indicated in the table above — including chemistries (Cr, ALT, AST, alkaline phosphatase, total bilirubin, and albumin) and additional stored plasma — must be performed regardless of the timing of the participant's previous visit.
   - If the date of the event driven visit falls within two weeks of the target date of the participant's next regularly scheduled visit, a combined visit should be conducted on the day of the event driven visit.

   For all other event driven visits:
   - Chemistries and additional stored plasma are <u>not</u> required (chemistries may be performed if considered clinically indicated by the study site clinician; for example, to follow up on a previously identified toxicity).
   - If the date of the event driven visit falls within two weeks of the participant's previous visit, evaluations performed at the previous visit need not be repeated at the event driven visit; however, CD4/CD8 counts must be performed at visits for confirmation of immunologic failure and HIV-1 RNA PCR must be performed at visits for confirmation of virologic failure.
   - If the date of the event driven visit falls within two weeks of the target date of the participant's next regularly scheduled visit, a combined visit should be conducted on the day of the event driven visit.
l. Performed when study drug permanently discontinued for reasons other than toxicity or completion of randomized regimen. If this visit falls within the acceptable study visit window for a routine scheduled visit, then a combined visit should be done.
m. For participants who are pregnant at the time of their early discontinuation or end of study visit, an additional contact will be required to ascertain the pregnancy outcome.

n. All women entering Step 2 or Step 3 will have a Step Change Entry visit. For women not on a triple ARV regimen in Step 1, the Step Change Entry visit must be completed prior to initiation of HAART (Step 2) or prior to the first dose of the second line regimen HAART (Step 3).
o. If the Step Change Week 4 visit falls within 2 weeks of the next scheduled visit (either before or after delivery), a combined visit should be done at the next scheduled visit, completing all evaluations required for both visits.

1. If sufficient documentation of HIV status as specified in Section 2.411.1(1077BA) or Section 3.312 (1077BL) is not available, HIV diagnostic testing is to be done according to the specified algorithm.
2. Medical history includes all diagnoses identified in Pediatric/Maternal Diagnoses (which can be found at www.fstrf.org) active at screening or occurring during the current pregnancy, Maternal Endpoint Diagnoses (Appendix IV), allergies, cardiovascular history, smoking status, and alcohol intake status. Results of prior HIV-1 resistance testing should also be collected. Medication history includes complete HIV-1 treatment history, immune-based therapy, and HIV-related vaccines, including blinded study medications and concomitant medications as defined in the protocol taken within 30 days prior to study entry.
3. All diagnoses identified in Pediatric/Maternal Diagnoses (which can be found at www.fstrf.org), Maternal Endpoint Diagnoses (Appendix IV), $\geq$ grade 3 signs and symptoms, and any grade sign or symptom that leads to a change in treatment, ARVs, interval bone fractures, and concomitant medications as defined in the protocol will be collected. Smoking and alcohol intake status will be collected at L/D (or week 1), week 14, then every 24 weeks, and at the end of the study. Gynecologic status will be collected at week 14, week 50 and then every 48 weeks.
4. At 1077BA screening, 1077BA entry and 1077BL entry, a complete physical examination including blood pressure and, at a minimum, examination of skin, head, mouth, neck, auscultation of the chest, and cardiac, abdominal, and extremity exam should be performed. Following entry, a targeted physical exam driven by prior and new signs, symptoms and diagnoses should be performed; blood pressure should also be measured as part of all targeted exams. Height should be measured at screening and weight should be measured at all visits. In order to calculate creatinine clearance rates (see footnote 7), weight must be measured on each day of specimen collection for serum creatinine testing.
5. Adherence questionnaires are required at indicated timepoints for mothers in 1077BA and 1077BP while receiving ARV prophylaxis and while receiving a triple ARV regimen in Step 2 and/or Step 3. Adherence questionnaires are not required for mothers registered in 1077BL who do not enter 1077BP. Adherence questionnaires also are not required in any component following premature discontinuation of study drug.
6. Complete blood count includes hemoglobin, hematocrit, WBC, differential count, ANC and platelet count; MCV, MCH and MCHC also required at all indicated visits through week 1 postpartum.
7. ALT and serum creatinine for all women. Once the creatinine result is available, the Cockroft-Gault equation to calculate creatinine clearance for women should be used. HB_sAg+ women and women co-enrolled on IMPAACT P1084s (Tenofovir substudy) will have additional chemistries performed as noted in the table below; no additional blood is required.

Study Visit(s)	*Additional Chemistries (Local Lab)*	*Targeted women*
Collect at ENTRY and every 4 weeks through L/D. Thereafter, collect at every indicated visit except Postpartum Week 1. Also collect at Event Driven visits that are indicated for possible HBV exacerbation	ALT, serum creatinine, AST, alkaline phosphatase, total bilirubin and albumin	HBsAg+ women ONLY
Postpartum Week 38	ALT, serum creatinine, AST, alkaline phosphatase, total bilirubin and albumin	HBsAg+ women ONLY
P1084s Entry (occurs at 1077BA Entry or the Antepartum Week 2 visit), L/D or Postpartum Week 1, Postpartum Weeks 6, 26 and 74	Phosphorus and calcium	Women in IMPAACT P1084s ONLY

8. Collect specimens at all indicated time points for all women. Perform real-time for women on a triple ARV regimen; store for women not on a triple ARV regimen. At entry to 1077BA, perform test in real time for women assigned to Arms B and C and store specimen for women assigned to Arm A.

9. Stored EDTA Plasma for ARV Resistance Testing and Pharmacology (to be done retrospectively on a subset of women) and DBS for other studies/back-up. Pharmacology specimens are targeted for women on the triple ARV regimen at delivery, weeks 1, 6, 14, 26, and subsequent visits during breastfeeding; the timing of the last three doses of ARVs will be collected at these timepoints.
10. HB_sAg+ women in 1077BA and 1077BP should have additional blood collected for hepatitis B studies as shown in the SoE above and in the table below.

Study Visit(s)	*Volume*	*Assays to be performed (Central Laboratory)*
(1077BA) Entry, L/D Postpartum Week 50 then q48 weeks, Early D/C or End of Study, Premature D/C of Study Drug and Step Change Entry, and Event Driven visits for possible HBV exacerbation	6 mL	HBeAg, HBeAb, HBV viral load and viral sequencing
Antepartum Weeks 4, 8, 12 and q4 weeks thereafter through delivery	2 mL	HBV viral load
Postpartum Week 6 and 26	4 mL	HBV viral load, HBeAg, and HBeAb

11. Stored breastmilk 20 mL (minimum of 10 mL) during breastfeeding at all indicated timepoints.
12. CD4/CD8 must be performed in a DAIDS IQA/UKNEQAS Lab. Additional CD4 and CD8 counts may be performed late in gestation and/or within the first week postpartum (up to day 14 postpartum). In particular, it is recommended that an additional CD4 count be performed at an antepartum study visit occurring at or after 36 weeks gestation to provide a CD4 count with a specimen collection date within 30 days prior to entry into 1077BP (required for eligibility determination). Specimen collection for CD4 and CD8 counts is not recommended during labor or within the first 24 hours postpartum. When more than one CD4 cell count with a specimen collection date within 30 days prior to entry into 1077BP is available, the count with the latest date should be used to determine eligibility for 1077BP.
13. Participating Sites (1077BP only): Collect hair from mothers in 1077BP Step 1 Arm A (maternal prophylaxis) at indicated time points while receiving triple ARV prophylaxis; also collect hair from all mothers following entry into 1077BP Step 2 or 1077BP Step 3 while breastfeeding and receiving a triple ARV regimen. Instructions for collection of the hair may be found in the Laboratory section of the MOP.

NOTE: Acceptable visit windows are +/- 1 week for all visits during pregnancy, Week 6 postpartum, and Step Change Week 4 Visits; +/- 2 weeks for the Week 14 visit and +/- 6 weeks for the Week 26, 38, 50, 62, 74 and q12 weeks visits. The L/D visit can be completed through Day 5 postpartum and the Week 1 postpartum visit can be completed on Days 6-14 postpartum. Efforts should be made to coordinate mother and infant visits. All End of Study visits are to be completed within a 12-week period.

APPENDIX IB
INFANT SCHEDULE OF EVALUATIONS
(1077BA, 1077BL, 1077BP)

	ALL INFANTS				PRIOR TO BREASTFEEDING CESSATION		AFTER BREASTFEEDING CESSATION		ALL INFANTS
	BIRTH[a]	WK 1[b]	WK 6[c]	WK 10	WKS 14, 18, 22 & 26	WKS 38, 50, 62, 74, 86, 98 & 104	WKS 14, 26, 38, 50, 62, 74, 86, & 98[f]	WK104	EARLY D/C
CLINICAL EVALUATIONS									
Birth And Neonatal Medical History[1]	X								
Physical examination[2]	X	X	X	X	X	X	X	X	X
Interval History, Signs/Sx[3]		X	X	X	X	X	X	X	X
Infant Feeding Practices Assessment		X	X	X	X	X			X
Pediatric Resource Interview					Wks 14, 26	Wks 50, 74, 98, 104	Wks 14, 26, 50, 74, 98	X	X
Motor Milestones					Wk 26	Wks 50, 74, 104	Wks 26, 50, 74	X	
Adherence Interview[4]		X	X		Wks 14, 26	Wks 50, 74			X
Socioeconomic Questionnaire		X			Wk 22	Wk 50	Wks 26, 50		
LABORATORY EVALUATIONS									
Complete Blood Count[5]	1mL	1mL	1mL		1mL[6] (Wk 14)	1mL[6] (Wk 50, 74)			1mL
Chemistries[7]	1mL	1mL	1mL		1mL (Wk 26)	1mL[7]			1mL
VIROLOGY									
HIV Nucleic Acid Test[8]	3mL	3mL	3mL		3mL (Wks 14, 26)	3mL	3mL[8] (Wks 14, 26, 50)		3mL
HIV EIA or Rapid HIV Test[8]							1mL (Wks 74, 98)		
Stored EDTA Plasma, DBS[9]	From NAT	From NAT	From NAT		From NAT	From NAT	From NAT or EIA		From NAT
Additional Stored Plasma (Infants of HBsAg + Women Only)[10]			2mL		2 mL (Wk 26)	3mL (Wks 38, 50, 104)	2-3mL (Wks 26, 38, 50)	3mL	
TOTAL BLOOD VOLUMES (Higher volumes for infants of HBsAg+ women)	5mL	5mL	5-7mL	0mL	4-6mL	4-8mL	1-6mL	3mL	5mL
HAIR COLLECTION									
Stored Hair (during BF only)[11]		X	X	X	X	X			X
IMMUNOLOGY (FOR INFANTS WITH HIV INFECTION ONLY)									
CD4 and CD8 Lymphocyte Percentage and Absolute Count[12]	1-2mL – at the time of confirmation and infants with confirmed HIV Infection thereafter<br>*CBC to be done at same timepoints as CD4/CD8, using same 1mL if lab capabilities permit; otherwise an additional 1mL is drawn for the CBC*								

a. Birth visit can be completed through day 5 of life.
b. Week 1 visit can occur between 6 and 14 days of life. This is the 1077BP entry visit.
c. Infants of late-presenting women who are NOT enrolled in 1077BP should complete evaluations for "Early D/C or End of Study" visit at Week 6. For infants of Hep B positive women it is not necessary to collect the additional stored plasma.

1. Infant history from delivery, e.g., birth weight and gestational age.
2. A complete physical examination including examination of skin, head, mouth, neck, auscultation of the chest, and cardiac, abdominal, and extremity exam should be performed. Following entry, a targeted physical exam driven by prior and new signs, symptoms, and diagnoses should be completed. Length, weight and head circumference and fontanel closure should be collected at each required visit.
3. All diagnoses identified in the Pediatric/Maternal Diagnoses (which can be found at www.fstrf.org), ≥ grade 3 signs and symptoms, and any grade sign or symptom that leads to a change in study drug regimen, interval bone fractures and concomitant medications will be collected.
4. Adherence is assessed by questionnaire. Adherence questionnaires are required for infants on study drug in 1077BA, 1077BL and 1077BP as follows:
   - Infants in 1077BA and 1077BL: Administer adherence questionnaire for all infants at indicated time points through Week 6 postpartum.
   - Infants in 1077BP: Administer adherence questionnaire for all infants at indicated time points through Week 6 postpartum. Thereafter, administer adherence questionnaires for infants assigned to Step 1 Arm B (infant prophylaxis) at indicated timepoints while receiving ARV prophylaxis.
   - Infants in 1077BA, 1077BL and 1077BP: Adherence questionnaires are not required following premature discontinue of study drug.
5. Complete blood count includes hemoglobin, hematocrit, WBC, differential count, ANC and platelet count.
6. CBC as indicated on the table and for infants with confirmed HIV infection when immunology assessments are performed.
7. Infants will have chemistries assessed at the times indicated in the table below; no additional blood is required.

Study Visit(s)	*Chemistries (Local Laboratory)*	*Targeted infants*
Birth, Weeks 1, 6 and 26	ALT	All infants
Week 38, and every 12 weeks while receiving NVP	ALT	Infants in 1077BP Step 1 Arm B (infant NVP) ONLY
P1084s Entry (Week 1), Weeks 10, 26 and 74	Creatinine, phosphorus and calcium	Infants enrolled on IMPAACT P1084s (Tenofovir substudy) ONLY

8. Infant HIV testing will be done as indicated below:
   - Prior to the 74 week visit: HIV NAT (HIV DNA PCR is preferred; if not available HIV RNA PCR can be used). If the initial HIV NAT is positive, confirm as soon as possible with a repeat HIV NAT on a second sample drawn on a different day and collect an additional 1 mL for plasma storage at the same time.
   - At or after the 74 weeks visit and have not achieved complete cessation of breastfeeding: HIV NAT (HIV DNA PCR is preferred; if not available HIV RNA PCR can be used). If the initial HIV NAT is positive, confirm as soon as possible with a repeat HIV NAT on a second sample drawn on a different day and collect an additional 1 mL for plasma storage at the same time.
   - At or after the 74 week visit and have achieved complete cessation of breastfeeding: HIV antibody testing (EIA or rapid). If HIV antibody test is negative, no further HIV testing is necessary. If HIV antibody test is positive, perform HIV NAT as soon as possible on a separate sample on a different day.
9. Stored EDTA plasma for ARV resistance testing and pharmacology (to be done retrospectively on a subset of infants) and DBS for other studies/back-up. Pharmacology specimens are targeted for weeks 1, 6, 14 and 26 and subsequent timepoints during breastfeeding; the timing of the last three doses of ARVs will be collected at these time points.

10. Infants of HB_sAg+ women should have additional blood collected for hepatitis B studies as shown in the SoE above and the table below.

Study Visit(s)	*Volume*	*Assays to be performed (Central Laboratory)*
Weeks 6 and 26	2mL	HBsAg, HBeAg, HBeAb, HBV viral load and viral sequencing
Week 38	3mL	HBsAg, HBeAg, HBeAb, HBV viral load and viral sequencing
Week 50 and 104	3mL	HBsAg, HBeAg, HBeAb, HBV viral load and viral sequencing, HBsAb

11. Assessments should be performed at the time of confirmation of infant HIV infection and every 12 weeks thereafter on infants with confirmed infection only. A CBC should also be performed at visits when the specimen for immunology assays is obtained.
12. *Participating sites:* Hair should be collected from 1077BP entry until complete cessation of breastfeeding from all infants in 1077BP (Arms A and B). Instructions for collection of the hair may be found in the Laboratory section of the MOP.

NOTE: Infant blood amounts are expected to be limited; therefore, priorities for laboratory assays will be as follows. If venipuncture is not successful, collect DBS for storage via Heel Stick Method per MOP.

1. Safety Laboratory Assessments (Chemistries and CBC)
2. HIV NAT/EIA
3. Stored DBS
4. Stored Plasma

NOTE: With the exceptions noted above for Birth and Week 1 (footnotes a and b), acceptable visit windows are +/- 1week through Week 10. For breastfeeding infants +/- 1 week from Week 14 through Week 26 and +/- 4 weeks from Week 38 through complete cessation of breastfeeding or Week 104 (whichever comes first); after complete cessation of breastfeeding, +/- 4 weeks from Week 14 through Week 104.

Management of HIV-infected infants: Infants confirmed to have HIV infection should have study drug (NVP) discontinued, if applicable, and continue to be followed according to this schedule. Blood for HIV NAT or HIV EIA should be collected for storage only (see footnote 8). CD4 and CD8 lymphocyte percentage and absolute count will be available through study laboratories at approximately q12 week intervals.

APPENDIX IC
MATERNAL HEALTH SCHEDULE OF EVALUATIONS
(1077BM, ALL STEPS)

	TIME BASED VISITS						EVENT BASED VISITS				
	SCREENING[a]	BM ENTRY[b]	WK 4	WK 8[c]	WK 12	WK 24 & Q12 WKS	EVENT DRIVEN VISIT[d]	PREMATURE D/C OF STUDY DRUG[e]	EARLY D\C OR END OF STUDY[f]	STEP CHANGE ENTRY[g]	STEP CHANGE WK 4[h]
CLINICAL EVALUATIONS											
Informed consent	X										
Interval hx, signs/sx[1]	X	X	X	X	X	X	X	X	X	X	X
Physical exam[2]	X	X	X	X	X	X	X	X	X	X	X
WHO Clinical Staging	X					X (q24 wks)		X	X	X	
Adherence Interview[3]		X	X		X	X (q24 wks)	X		X	X	X
QOL/Resource Utilization Questionnaire		X			X	X (q24 wks)		X	X	X	
Food Insecurity Questionnaire		X			X	X (through Wk 98 postpartum)		X			
LABORATORY EVALUATIONS											
Complete blood count[4]	3mL	3mL	3mL		3mL	3mL (q24 wks)		3mL	3mL	3mL	3mL
Chemistries[5]	2mL	2mL	2mL	2mL	2mL	2mL (q24 wks)	2mL	2mL	2mL	2mL	2mL
Pregnancy test		Urine (5mL) or serum (1mL blood in SST or NON tube) test is acceptable. For women on EFV, required at every visit while on EFV and through 12 weeks after stopping EFV. Otherwise, to be done only when pregnancy is suspected or when considered clinically indicated by the study site clinician.									
VIROLOGY											
HIV-1 RNA PCR[6]		6mL	6mL		6mL	6mL	6mL	6mL	6mL	6mL	6mL
Stored EDTA Plasma, DBS (All women)[7]		10mL	10mL		10mL	10mL	10mL	10mL	10mL	10mL	10mL
Additional Stored Plasma (HBsAg + Women only)[8]		6mL		4mL		4-6mL[8]	6mL	6mL	6mL	6mL	
IMMUNOLOGY											
CD4 and CD8 lymphocyte percentage and absolute count	3mL	3mL			3mL	3mL		3mL	3mL	3mL	
TOTAL BLOOD VOLUMES (higher volume for HBsAg+ women)	8mL	24-31mL	21-22mL	6-7mL	24-25mL	19-31mL	18-25mL	24-31mL	24-31mL	24-31mL	21-22mL

a. Assessments performed in previous component (AP or PP) may serve as the screening assessments for the MH Component if they are performed within the timeframes specified in the eligibility criteria.

b. Entry visit must be completed within the timeframe specified in eligibility criterion 5.311.

c. Week 8 visit is for HB_sAg+ women ONLY.

d. Event driven visits should be performed for the following reasons:
   - Confirmation of immunologic failure
   - Confirmation of virologic failure
   - Discontinuation of HAART regimen for toxicity reasons
   - Clinically significant event suggestive of acute exacerbation of Hepatitis B, including any grade 2 or higher liver function test result (symptomatic or asymptomatic) and/or any of the following signs/symptoms (regardless of liver function test results): scleral icterus, jaundice, petechiae, otherwise unexplained abdominal pain, abdominal swelling, lower extremity swelling, fever occurring with any of these signs/symptoms (HBsAg+ women only)

   See the study MOP for more information on the required timing of these visits.

   For event driven visits conducted for clinically significant events suggestive of acute exacerbation of Hepatitis B:
   - All evaluations indicated in the table above — including chemistries (Cr, ALT, AST, alkaline phosphatase, total bilirubin, and albumin) and additional stored plasma — must be performed regardless of the timing of the participant's previous visit.
   - If the date of the event driven visit falls within two weeks of the target date of the participant's next regularly scheduled visit, a combined visit should be conducted on the day of the event driven visit.

   For all other event driven visits:
   - Chemistries and additional stored plasma are <u>not</u> required (chemistries may be performed if considered clinically indicated by the study site clinician; for example, to follow up on a previously identified toxicity).
   - If the date of the event driven visit falls within two weeks the participant's previous visit, evaluations performed at the previous visit need not be repeated at the event driven visit; however, CD4/CD8 counts must be performed at visits for confirmation of immunologic failure and HIV-1 RNA PCR must be performed at visits for confirmation of virologic failure.
   - If the date of the event driven visit falls within two weeks of the target date of the participant's next regularly scheduled visit, a combined visit should be conducted on the day of the event driven visit.

e. Performed when study drug permanently discontinued for reasons other than toxicity. If this visit falls within the acceptable study visit window for a routine scheduled visit, then a combined visit should be done.

f. For participants who are pregnant at the time of their early discontinuation or end of study visit, an additional contact will be required to ascertain the pregnancy outcome.

g. All women entering Step 2 or Step 3 will have a Step Change Entry Visit. For women not on a triple ARV regimen in Step 1 the Step Change Entry visit must be completed prior to initiation of HAART (Step 2) or prior to the first dose of the second line regimen HAART (Step 3).

h. If the Step Change Week 4 visit falls within 2 weeks of the next scheduled visit, then a combined visit should be done, completing all evaluations required for both visits.

1. All diagnoses identified in the Pediatric/Maternal Diagnoses (which can be found at www.fstrf.org), Maternal Endpoint Diagnoses (Appendix IV), $\geq$ grade 3 signs and symptoms and any grade sign or symptom that leads to a change in treatment, ARVs, interval bone fractures and concomitant

medications will be collected. Smoking and alcohol intake status will be collected at entry, every 24 weeks, and at end of study. Gynecologic status will be collected at entry, week 12 (only if the mother did not participate in the BP Component (1077BP), week 48 and then every 48 weeks.

2. At entry to 1077BM, a complete physical examination including blood pressure and, at a minimum, examination of skin, head, mouth, neck, auscultation of the chest, and cardiac, abdominal, and extremity exam should be performed. Following entry, a targeted physical exam driven by prior and new signs, symptoms and diagnoses should be performed; blood pressure should also be measured as part of all targeted exams. Weight should be measured at all visits, in order to calculate creatinine clearance rates (see footnote 5), weight must be measured on each day of specimen collection for serum creatinine testing.
3. Adherence questionnaires are required at indicated timepoints for mothers in 1077BM Step 1 Arm A, Step 2 and/or Step 3 while receiving a triple ARV regimen. Adherence questionnaires are not required following premature discontinuation of study drug.
4. Complete blood count includes hemoglobin, hematocrit, WBC, differential count, ANC and platelet count.
5. ALT and serum creatinine for all women. Once the creatinine result is available, the Cockroft-Gault equation to calculate creatinine clearance for women. In addition, HB_sAg+ women and women co-enrolled on IMPAACT P1084s (Tenofovir substudy) will have additional chemistries performed as noted in the table below; no additional blood is required.

Study Visit(s)	*Additional Chemistries (Local Lab)*	*Targeted women*
Every indicated visit and only Event Driven Visits for possible HBV exacerbation	AST, alkaline phosphatase, total bilirubin and albumin	HB_sAg+ women ONLY
At 1077BM visits closest in time to the following P1084s visits: Weeks 6, 26 and 74	Phosphorus and calcium	Women in IMPAACT P1084s ONLY

6. Collect specimens at all indicated time points for all women. Perform real-time for women on a triple ARV regimen; store for women not on a triple ARV regimen.
7. Stored EDTA plasma for ARV resistance testing (to be done retrospectively on a subset of women) and DBS for other studies/back-up.
8. HB_sAg+ women should have additional blood collected for hepatitis B studies as shown in the SoE above and the table below.

Study Visit(s)	*Volume*	*Assays to be performed (Central Lab)*
Entry, Week 48 then q48 weeks, Step Change Entry, Premature D/C of Study Drug, End of Study, and Event Driven visits for possible HBV exacerbation	6mL	HBeAg, HBeAb, HBV viral load and viral sequencing
Weeks 8 and 24 in women randomized to discontinue their triple ARV regimen (Step 1 Arm B) ONLY	4mL	HBeAg, HBeAb, HBV viral load

NOTE: Acceptable visit windows are +/- 1 week for the Week 4 visit and Step Change Week 4 visit, +/-2 weeks for Weeks 8 and 12 visits and +/- 6 weeks for the q12 week visits. Efforts should be made to coordinate mother and infant visits. All End of Study visits are to be completed within a 12-week period.

APPENDIX II
TOXICITY MANAGEMENT

Unanticipated and anticipated toxicities will be graded according to the Division of AIDS Table for Grading the Severity of Adult and Pediatric Adverse Events, Version 1.0, December 2004 with Clarification dated August 2009. As described in the remainder of this appendix, site investigators will manage toxicities based on severity grade and, in some cases, relationship to study drug. Site investigators should consult on toxicity management with the study Clinical Management Committee (CMC) as directed in this appendix and may additionally consult with the CMC when needed, at their discretion. When consulting with the CMC, site investigators should follow the CMC communication procedures contained in the study Manual of Procedures. *Information on study step or randomization arm should NOT be included in correspondence with the CMC unless this information is specifically requested by the CMC.*

General Guidelines for Management of Toxicities Not Detailed in the Guidance on Toxicity Management Tables

The following general guidelines apply to management of study drug regimens in response to all toxicities, unless superseded by directions in the Guidance on Toxicity Management Tables (provided below) that give specific information on management of the following:

- Anemia and neutropenia
- Elevated ALT or AST
- Decreased creatinine clearance
- Rash
- Elevated serum triglycerides or cholesterol

For participants for whom study drug is held for toxicity management, relevant clinical and laboratory evaluations should be repeated per the grade- or toxicity-specific guidance provided below until the toxicity resolves or is stabilized.

For participants on a triple ARV regimen, if one ARV must be held for toxicity management, all ARVs in the regimen should be held concurrently.

For pregnant women, additional clinical evaluation may be required to rule out gestational diabetes, pre-eclampsia, or other treatable causes of anemia.

Toxicities assessed as related to non-study drugs (concomitant medications) should be handled according to the relevant package inserts and the best medical judgment of the site investigator.

General Guidelines for other Grade 1 or Grade 2 Toxicities

Participants who develop a Grade 1 or Grade 2 toxicity may continue study drug without alteration, with the exceptions noted in the tables below for specific toxicities.

General Guidelines for other Grade 3 Toxicities

For Grade 3 laboratory abnormalities, the site investigator should attempt to repeat the test to confirm the Grade 3 value as soon as possible and generally within 3 working days of site awareness. If the test cannot be repeated within 3 working days, it should be repeated as soon as possible and the CMC notified when the result is available. The result of the repeat test should be used to guide management of the toxicity.

If the result of the repeat test is Grade 1 or 2, the relevant management guidelines (Grade 1 or 2) should be followed. In this case, the initial grade 3 result should be recorded on case report forms (and included in EAE reports, if applicable).

For Grade 3 clinical and laboratory toxicities, alternate explanations for the toxicity should be sought prior to holding study drug.

For Grade 3 clinical and laboratory toxicities assessed as possibly, probably or definitely related to study drug, with the exception of isolated Grade 3 hyperbilirubinemia attributed to ataznavir (ATV), the implicated study drug(s) should be replaced or the entire regimen held, unless the site investigator feels that continuation of the current regimen is in the participant’s best interest. If the site investigator feels that continuation of the current regimen is in the participant’s best interest, the CMC should be informed. For Grade 3 isolated hyperbilirubinemia attributed to ATV, ATV may be continued unless associated with jaundice or scleral icterus that presents an intolerable cosmetic concern to the participant.

For Grade 3 clinical and laboratory toxicities assessed as probably not or not related to study drug, study drug may be continued.

For all Grade 3 toxicities, the participant should be re-evaluated weekly until the toxicity improves to Grade ≤ 2 or until stabilized.

If a study drug regimen is held due to a Grade 3 toxicity, the site investigator may resume the regimen once the toxicity improves to Grade ≤ 2. Following resumption of study drug, if the Grade 3 toxicity recurs, the implicated study drug(s) should be permanently discontinued. If one or more study drugs are not clearly implicated, the site investigator should consult the CMC prior to permanent discontinuation.

Participants experiencing Grade 3 toxicities requiring permanent discontinuation of an implicated study drug should be re-evaluated at least weekly until improvement to Grade ≤ 2 or until stabilized and no longer in need of such frequent monitoring, as determined by the site investigator.

Grade 3 and 4 acute and worsening depression, including suicidal ideation and suicide attempts, have been reported infrequently with use of FTC/TDF/RPV. Participants on FTC/TDF/RPV should be counseled to report severe depressive symptoms immediately because discontinuation of FTC/TDF/RPV may be required. In the event that a participant experiences treatment-limiting (in the opinion of the site investigator) depressive symptoms attributed to FTC/TDF/RPV, FTC/TDF/RPV should be permanently discontinued.

Guidelines for Grade 4 Toxicities

For Grade 4 laboratory abnormalities, the site investigator should attempt to repeat the test to confirm the Grade 4 value as soon as possible and generally within 3 working days of site awareness. Study drug (entire regimen) should be held pending the result of the repeat test. If the test cannot be repeated within 3 working days, it should be repeated as soon as possible and the CMC notified when the result is available. The result of the repeat test should be used to guide management of the toxicity (based on severity grade).

If the result of the repeat test is Grade 1, 2, or 3, the relevant management guidelines (Grade 1, 2, or 3) should be followed. In this case, the initial grade 4 result should be recorded on case report forms (and included in EAE reports, if applicable).

For all Grade 4 toxicities, with the exception of isolated Grade 4 hyperbilirubinemia attributed to ataznavir (ATV), all study drugs should be held until improvement of the toxicity to Grade ≤ 2 (for infants on NVP prophylaxis, NVP should be replaced with 3TC). Alternatively, the site investigator may continue study drug only if he or she has compelling evidence that the toxicity is NOT related to study drug. In this case, consultation with the CMC is required within 3 working days. The participant should be re-evaluated weekly until the toxicity improves to Grade ≤ 2 or until stabilized. For Grade 4 isolated hyperbilirubinemia attributed to ATV, ATV may be continued unless associated with jaundice or scleral icterus that presents an intolerable cosmetic concern to the participant.

Once a Grade 4 toxicity improves to Grade ≤ 2, use of study drug may be resumed; in this case, alternative study-provided or non-study-provided drugs should replace the implicated study drug(s). Alternatively, if the Grade 4 toxicity was assessed as probably not or not related to the study drug, the original regimen may be resumed at the discretion of the site investigator, with approval in advance from the CMC. Following resumption of study drug, if the Grade 4 toxicity recurs, the implicated study drug(s) should be permanently discontinued. If one or more study drugs are not clearly implicated, the site investigator should consult the CMC prior to permanent discontinuation.

Participants experiencing Grade 4 toxicities requiring permanent discontinuation of an implicated study drug should be followed at least weekly until improvement to Grade ≤ 2 or until stabilized and no longer in need of such frequent monitoring, as determined by the site investigator.

Grade 3 and 4 acute and worsening depression, including suicidal ideation and suicide attempts, have been reported infrequently with use of FTC/TDF/RPV. Participants on FTC/TDF/RPV should be counseled to report severe depressive symptoms immediately because discontinuation of FTC/TDF/RPV may be required. In the event that a participant experiences treatment-limiting (in the opinion of the site investigator) depressive symptoms attributed to FTC/TDF/RPV, FTC/TDF/RPV should be permanently discontinued.

Guidance on Toxicity Management Table for Specified Toxicities:

ANEMIA AND NEUTROPENIA		
CONDITION AND SEVERITY	STUDY DRUG USE	FOLLOW-UP AND MANAGEMENT
Grade 1	Continue study drug	
Grade 2	Continue study drug<br>(or manage as in management box)	Participants on ZDV may continue study drug unchanged or may substitute TDF or another NRTI for ZDV at the discretion of the site investigator
Grade 3 possibly related, probably not related, or not related	Continue study drug	Repeat test to confirm within 3 working days.<br>If repeat assessment is Grade ≤ 2 manage as per Grade 2.<br>If repeat assessment is Grade 3:<br>- For asymptomatic infants on NVP prophylaxis, repeat test again every 7-10 days until improvement to grade ≤ 1. If Grade 3 values persist over the course of three additional repeat tests, consult the CMC on study drug regimen and frequency of repeat assessments. Consider holding cotrimoxazole prophylaxis.<br>- For all other participants, repeat test again within 7 days. If Grade 3 persists, consult the CMC on study drug regimen and frequency of repeat assessments.
Grade 3 probably related or related<br>OR<br>Grade 4 that is not immediately life threatening	For infants on NVP prophylaxis, continue NVP pending repeat testing for confirmation of grade<br>For all other participants, hold all study drugs or replace suspect study drug	Repeat test to confirm within 3 working days.<br>If repeat assessment is Grade ≤ 2 manage as per Grade 2 (infants may continue NVP prophylaxis).<br>If repeat assessment is Grade 3:<br>- For infants on NVP prophylaxis, upon confirmation of Grade 3, replace NVP with 3TC and consider holding cotrimoxazole prophylaxis. Consult the CMC on study drug regimen and frequency of repeat assessments.<br>- For all other participants, continue immediate action (hold all study drugs or replace suspect study drug) and consult the CMC within 3 working days on study drug regimen and frequency of repeat assessments.

ANEMIA AND NEUTROPENIA		
CONDITION AND SEVERITY	STUDY DRUG USE	FOLLOW-UP AND MANAGEMENT
Grade 4 that is immediately life threatening based on clinical findings (e.g., fever, illness)	Hold all study drugs	Repeat test to confirm within 3 working days.<br>If repeat assessment is Grade < 4, manage per the grade of the repeat assessment (asymptomatic infants may resume NVP prophylaxis once the toxicity grade improves to Grade ≤ 2).<br>If repeat assessment is Grade 4:<br>- For infants on NVP prophylaxis, upon confirmation of Grade 4, replace NVP with 3TC and consider holding cotrimoxazole prophylaxis. Consult the CMC on study drug regimen and frequency of repeat assessments.<br>- For all other participants, continue immediate action (hold all study drugs) and consult the CMC within 3 working days on study drug regimen and frequency of repeat assessments.

Guidance on Toxicity Management Table for Specified Toxicities:

ASYMPTOMATIC ELEVATIONS in AST or ALT		
CONDITION AND SEVERITY	STUDY DRUG USE	FOLLOW-UP AND MANAGEMENT
Grade 1<br>Asymptomatic	Continue study drug	Repeat test as soon as possible and within 14 days.<br>If repeat assessment is Grade ≤1, continue study drug.<br>If participant becomes symptomatic, follow guidance for symptomatic hepatitis.
Grade 2<br>Asymptomatic	Continue study drug	Repeat test as soon as possible and within 14 days.<br>Assess for alcohol use, non-study medication-related drug toxicity, lactic acidosis syndrome, pre-eclampsia, fatty liver of pregnancy, and viral hepatitis as the cause of the AST/ALT elevation. If the AST/ALT elevation is considered most likely to be due to concomitant illness or medication, standard management, including discontinuation of the likely causative agent, should be undertaken.<br>If repeat assessment is Grade ≤2, continue study drug.<br>If participant becomes symptomatic, follow guidance for symptomatic hepatitis.<br>Note: For HBsAg+ mothers, perform event-driven visit per the relevant schedule of evaluations for clinically significant event suggestive of acute exacerbation of hepatitis.

Guidance on Toxicity Management Table for Specified Toxicities:

ASYMPTOMATIC ELEVATIONS in AST or ALT		
CONDITION AND SEVERITY	STUDY DRUG USE	FOLLOW-UP AND MANAGEMENT
Grade 3<br>Asymptomatic	Continue study drug	Repeat test within 3 working days.<br><br>Assess for alcohol use, non-study medication-related drug toxicity, lactic acidosis syndrome, pre-eclampsia, fatty liver of pregnancy, and viral hepatitis as the cause of the AST/ALT elevation.<br><br>If repeat assessment is Grade ≤2, manage as per Grade 2.<br><br>If repeat assessment is Grade 3 and is attributed to concomitant illness or medication (probably not or not related to study drug), study drug may be continued at the discretion of the site investigator. Treat the underlying illness or remove the likely causative agent.<br><br>If the repeat assessment is Grade 3 and is assessed as possibly, probably, or definitely related to study drug, hold study drug (entire regimen); for infants on NVP prophylaxis, replace NVP with 3TC upon confirmation of Grade 3. Repeat testing weekly and once the toxicity grade is Grade ≤2, study drug may be resumed with replacement of the implicated study drug(s). If one or more study drugs are not clearly implicated, the site investigator should consult the CMC on the regimen to be resumed. Should the site investigator wish to resume an implicated study drug, consultation with the CMC is required in advance.<br><br>If study drug is resumed following a hold for Grade 3 AST/ALT, repeat testing should be performed one week after resumption. If the result of this testing is Grade 3 or 4, consult the CMC. Otherwise, it is not necessary to report the results to the CMC.<br><br>If participant becomes symptomatic, follow guidance for symptomatic hepatitis.<br><br>Note: For HBsAg+ mothers, perform event-driven visit per the relevant schedule of evaluations for clinically significant event suggestive of acute exacerbation of hepatitis.

ASYMPTOMATIC ELEVATIONS in AST or ALT		
CONDITION AND SEVERITY	STUDY DRUG USE	FOLLOW-UP AND MANAGEMENT
Grade 4<br>Asymptomatic	Hold study drug	Repeat test within 3 working days, in addition to total bilirubin and INR if available at the site.<br><br>Assess for alcohol use, non-study medication-related drug toxicity, lactic acidosis syndrome, pre-eclampsia, fatty liver of pregnancy, and viral hepatitis as the cause of the AST/ALT elevation.<br><br>If repeat assessment is Grade < 4, manage per the grade of the repeat assessment.<br><br>If repeat assessment is Grade 4, continue to hold study drug (entire regimen); for infants on NVP prophylaxis, replace NVP with 3TC upon confirmation of Grade 4. Consult the CMC on study drug regimen and frequency of repeat assessments while following ALT/AST at least weekly. Once the toxicity grade is Grade ≤ 1, study drug may be resumed with replacement of the implicated study drug(s). If one or more study drugs are not clearly implicated, the site investigator should consult the CMC on the regimen to be resumed. Should the site investigator wish to resume an implicated study drug, consultation with the CMC is required in advance.<br><br>If study drug is resumed following a hold for Grade 4 AST/ALT, repeat testing should be performed one week after resumption. If the result of this testing is Grade 3 or 4, consult the CMC. Otherwise, it is not necessary to report the results to the CMC.<br><br>If participant becomes symptomatic, follow guidance for symptomatic hepatitis.<br><br>Note: For HBsAg+ mothers, perform event-driven visit per the relevant schedule of evaluations for clinically significant event suggestive of acute exacerbation of hepatitis.

Guidance on Toxicity Management Table for Specified Toxicities:

SYMPTOMATIC HEPATITIS	
CONDITION AND SEVERITY	STUDY DRUG USE, FOLLOW-UP, AND MANAGEMENT
Signs and symptoms of hepatitis include but are not limited to fatigue, malaise, anorexia, nausea, acholic stools, bilirubinuria, jaundice, liver tenderness, and/or hepatomegaly (icteric sclera in isolation without systemic complaints would not be considered symptomatic).	If participant is on NVP:<br><br>- Immediately perform AST and ALT tests, in addition to total bilirubin and INR if available at the site. If AST or ALT has increased one or more grades above the participant's baseline value, immediately hold NVP. Also hold NVP if the participant's signs and symptoms include acholic stools, bilirubinuria, jaundice, liver tenderness, or hepatomegaly.<br><br>- Consult the CMC on study drug regimen and frequency of repeat assessments (in general, at least weekly re-assessment is recommended). .<br><br>- If it is determined that the participant has clinical hepatitis with or without liver function test abnormalities and NVP cannot be excluded as the cause, NVP should be permanently discontinued.<br><br>Note: For HBsAg+ mothers, perform event-driven visit per the relevant schedule of evaluations for clinically significant event suggestive of acute exacerbation of hepatitis.
	If participant is not on NVP:<br><br>- Immediately perform AST and ALT tests, in addition to total bilirubin and INR if available at the site; follow general management guidelines based on the highest grade sign or symptom.<br><br>- Consult the CMC on study drug regimen and frequency of repeat assessments (in general, at least weekly re-assessment is recommended).<br><br>Note: For HBsAg+ mothers, perform event-driven visit per the relevant schedule of evaluations for clinically significant event suggestive of acute exacerbation of hepatitis.

Guidance on Toxicity Management Table for Specified Toxicities:

CREATININE CLEARANCE (CrCl)		
CONDITION AND SEVERITY	STUDY DRUG USE	FOLLOW-UP AND MANAGEMENT
Estimated CrCl < 50 (initial value)	Continue study drug unless participant is symptomatic	Repeat test and CrCl calculation (Cockcroft-Gault formula) as soon as possible (and within 1 week)
Confirmed CrCl < 50	Manage study drug as defined here or in package inserts	Participants with a confirmed CrCl rate < 50 mL/min should undergo a thorough evaluation for potential causes of decreased renal function in addition to receiving treatment, as appropriate. May substitute ZDV or d4T or ABC for TDF with appropriate renal dosing adjustments (see below) while the etiology of the renal insufficiency is being investigated and renal function is being closely followed. Consult the CMC as needed on evaluating causes of renal insufficiency and potential relationship to TDF.<br><br>If TDF is the only potential cause of renal insufficiency found, TDF should be permanently discontinued.<br><br>Follow weekly until CrCl rate returns to ≥ 60 mL/min. Once CrCl rate is ≥ 60 mL/min, and if the renal insufficiency was ascribed to etiologies other than TDF, TDF-containing regimens may be resumed with careful monitoring* of renal function.<br><br>If the CrCl remains < 60, and TDF has been excluded as a cause of the renal insufficiency, after consultation with the CMC, a TDF-containing regimen may be restarted with careful monitoring* and appropriate renal dosing adjustments of the drugs in the regimen.<br><br>*Careful monitoring of renal function should include weekly re-assessment of CrCl for one month and monthly re-assessment for the next three months.<br><br>For Lamivudine (3TC), recommended renal dosing adjustments are as follows:<br>• If CrCl ≥50, 150 mg twice daily or 300 mg once daily<br>• If CrCl 30-49, 150 mg once daily<br>• If CrCl 15-29, 150 mg first dose, then 100 mg once daily<br>• If CrCl 5-14, 150 mg first dose, then 50 mg once daily<br>• If CrCl <5, 50 mg first dose, then 25 mg once daily<br><br>For Stavudine (d4T), recommended renal dosing adjustments (assuming a starting dose of 30 mg every 12 hours) are as follows:<br>• If CrCl >50, 30 mg every 12 hours<br>• If CrCl 26-50, 15 mg every 12 hours<br>• If CrCl 10-25, 15 mg every 24 hours

Guidance on Toxicity Management Table for Specified Toxicities:

<table>
<tr><th colspan="3">RASH</th></tr>
<tr><th>CONDITION AND SEVERITY</th><th>STUDY DRUG USE</th><th>FOLLOW-UP AND MANAGEMENT</th></tr>
<tr><td rowspan="2">Grade 1 or Grade 2</td><td>If on NVP, EFV, or abacavir: study drugs may need to be held depending on rash distribution and relatedness assessment</td><td>If the rash is generalized and there is no definitive explanation for the rash:
• Hold study drug (entire regimen)
• Test ALT within 3 working days, and
• Evaluate for symptoms of clinical hepatitis and hypersensitivity reaction.
If any clinical symptoms of hepatitis or ALT elevation or hypersensitivity reaction, permanently discontinue NVP, EFV, or abacavir and consult with CMC on study drug regimen.

If the rash is not generalized or if there is a definitive explanation for the rash (e.g., varicella), study drug may be continued with no additional evaluation required.</td></tr>
<tr><td>If not on NVP, EFV, or abacavir: continue study drug</td><td>May be treated symptomatically, but should be monitored closely by the site investigator.</td></tr>
<tr><td>Grade 3</td><td>Hold all study drugs unless the rash is determined to be unrelated to study drug</td><td>If there is no definitive explanation for the rash (e.g., varicella), test ALT and manage per the ALT/AST elevation table.

If on NVP, EFV, or abacavir, permanently discontinue this/these drugs. When the rash resolves, study drug may be resumed (except abacavir or NVP or EFV).</td></tr>
<tr><td>Grade 4</td><td>Hold all study drugs</td><td>Consult the CMC on possible alternative study drug regimens.</td></tr>
</table>

Management of Immune Reconstitution Inflammatory Syndromes

Inflammatory syndromes have been reported to occur shortly after the initiation of potent combination ART. When these syndromes are suspected the following management plan should be followed, and consultation with the study CMC is recommended:

- Continue ARV treatment.
- Confirm diagnosis of opportunistic infection (OI).
- Continue or initiate specific therapy for the infection.
- Evaluate the participant clinically to exclude a new infectious process if the participant was already receiving therapy for the OI.

Initiate anti-inflammatory agents, initially nonsteroidals or, if needed corticosteroids at the discretion of the site investigator in consultation with the CMC

APPENDIX III
OVERVIEW OF INTERIM MONITORING OF PROMISE

I. Safety Monitoring

Participant safety is of paramount importance to the PROMISE team. A multi-tiered safety review process will be followed for the duration of this study. The review process includes several levels of evaluation by various Network members and groups. This process, which is both timely and extensive in scope, includes review of medical history information, laboratory values, adverse events and - in the DSMB reviews - outcome measures.

The study site investigators are responsible for continuous close safety monitoring of all study participants, for the initial evaluation and reporting of safety information at the participant level, and for alerting the Protocol Chairs and Clinical Management Committee (CMC) if unexpected concerns arise.

A subgroup of the CMC, the Toxicity Review Group, will convene routinely to review clinical and laboratory data reports (pooled across randomized treatment groups) generated by the SDMC. The Toxicity Review Group will include the Protocol Chairs or designees, a DAIDS Medical Officer or Monitor, the Protocol Statistician(s) and Data Manager(s), an NICHD Medical Officer, and a representative of the PROMISE Operations Center. The content, format and frequency of the clinical and laboratory data reports will be agreed upon by the CMC and the SDMC in advance of study implementation, and will be specified in the protocol monitoring plan. In addition to the routine safety data reviews, the CMC will convene on an ad hoc basis as needed to discuss any potential safety concerns. The CMC may be divided into components, most likely based on PMTCT and maternal health, as determined by the Protocol Chairs and Medical Officers. If divided, the divided groups will be constituted and will meet as described above.

EAE reports will be submitted in an expedited manner to the DAIDS Safety Office and will be forwarded upon receipt to the DAIDS Medical Officer and Safety Specialist for immediate review.

PROMISE will be monitored by a DSMB as described below.

Copies of IND safety reports that are submitted to the US FDA as well as summaries of DSMB reviews will be provided to participating sites both for their information and for required submission to the IRBs/ECs.

II. Interim Analyses for DSMB Review

Although the sequential randomization design of PROMISE allows different components to be analyzed separately, decisions about early stopping of an intervention in one component for either efficacy, toxicity, or futility may be informed by consideration of interim results for certain interventions in other components. For example:

- Within the Antepartum and Postpartum PMTCT components, use of a maternal triple ARV regimen for prevention of MTCT may also affect maternal health; for example, when evaluating the postpartum MTCT results for maternal triple ARV prophylaxis versus infant NVP, the primary consideration for determining which intervention to regard as the preferred treatment for this purpose is infant HIV infection rates and infant HIV-free survival. However, if maternal triple ARV prophylaxis had a positive or negative impact on maternal health, this would weigh into the decision of whether to adopt it for use to prevent postpartum MTCT.
- Interim results on the efficacy and safety of continuing versus discontinuing a maternal triple ARV regimen after delivery in resource-limited countries (Maternal Health Comparison #2a) may have implications for the post-BF cessation component (Maternal Health Comparison #2b).

Consequently, it is recommended that all components of PROMISE be monitored by the same DSMB.

Another consideration in the monitoring of PROMISE is that the accumulating results for its various Components will become mature at different times. For example, information about the relative efficacy of the antepartum/intrapartum treatments for prevention of MTCT will become available somewhat sooner than that about the relative efficacy of the postpartum interventions for prevention of MTCT. Table 1 presents a projected timeline for the interim monitoring of PROMISE based on the accrual assumptions in the protocol. Interim administrative and safety data for each Component will be reviewed at least annually after the first subject is enrolled to that Component. Annual interim efficacy analyses for each Component will be conducted once at least 25% of the information is available on the primary efficacy outcome measure. For example, the second column of Table 1 indicates that the interim efficacy analyses for the Antepartum Component are projected to be reviewed approximately 1 year and 2 years after the PROMISE study opens to accrual, when approximately 33% and 67% of the total information on the primary outcome measure for the Antepartum Component become available. The interim efficacy analysis schedule may be modified if accrual assumptions turn out to be inaccurate or if recommended by the DSMB.

Table 1: Projected interim monitoring schedule (% of information on primary outcome measure)

Year(s) from start accrual	Antepartum	Postpartum	Maternal Health Comparison 1 (a or b)	Maternal Health Comparison 2a	Maternal Health Comparison 2b
**+ 1 year**	S and E (33% Information)	S and E (25% information)	S	S	S
**+2 years**	S and E (67% information)	S and E (50% information)	S and E (33% information)	S and E (33% information)	S and E (27% information)
**+3 years**	F (100% information)	S and E (75% information)	S and E (50% information)	S and E (50% information)	S and E (45% information)
**+4 years**		F (100% information)	S and E (67% information)	S and E (67% information)	S and E (66% information)
**+5 years**			S and E (83% information)	S and E (83% information)	S and E (81% information)
**+6 years**			F (100% information)	F (100% information)	F (100% information)

S: Safety review/analysis
E: Efficacy interim analysis
F: Final analysis

The specific guidelines for considering early stopping or study modification based on the primary outcome measure for each PROMISE Component are described in the Component-specific statistical sections (protocol sections 6.1 for Antepartum, 6.2 for Postpartum, and 6.3 for Maternal Health. These sections also discuss additional considerations that should be taken into account when evaluating each Component, including consistency of the primary analysis with the results for secondary efficacy endpoints and safety, and consistency with specific other Components of PROMISE.

Because of these other considerations, interim analyses will be reported in terms of repeated confidence intervals as opposed to formal 'stopping' p-values using an O'Brien-Fleming spending function to control Type I error. An advantage of this approach is that the decision of whether or not to stop a particular Component of PROMISE need not be linked to a specific p-value. Thus, if for any reason, a component of PROMISE is modified or stopped, the corresponding confidence interval for the parameter reflecting the treatment difference (e.g., odds-ratio or hazard ratio) will be valid and 'adjusted' for the multiple interim analyses.

APPENDIX IV
MATERNAL ENDPOINT DIAGNOSES

The following AIDS-defining illnesses (WHO Clinical Stage 4), WHO Stage 2 and Stage 3 clinical events, non-AIDS-defining cancers and other targeted medical conditions have been identified for endpoint analysis.

The occurrence of these conditions during the study may trigger the collection of additional information for inclusion in the study database. The definitions of these conditions can be found on the DMC Web Site.

WHO Stage 4 Clinical Events

- Bacterial pneumonia, recurrent, severe (> 2 episodes in 12 months)
- Candidiasis of bronchi, trachea, lungs, esophagus
- Cryptococcosis, extrapulmonary including meningitis
- Cryptosporidiosis, chronic intestinal (> 1 month duration)
- Cytomegalovirus disease (retinitis or infection of other organs)
- Encephalopathy, HIV-related
- Herpes simplex, chronic (orolabial, genital, or anorectal site, > 1 month duration), or bronchitis, pneumonitis, esophagitis, or visceral at any site
- Isosporiasis, chronic intestinal (> 1 month duration) (confirmatory diagnostic testing required)
- Leishmaniasis, atypical, disseminated
- Mycobacterium avium complex (MAC) or M. kansasii, disseminated or extrapulmonary
- Mycobacterium tuberculosis (extrapulmonary)
- Mycobacterial infection, other species or unidentified species, disseminated or extrapulmonary
- Mycosis, disseminated (extrapulmonary histoplasmosis or coccidiomycosis)
- Penicilliosis, disseminated
- Pneumocystis pneumonia
- Progressive multifocal leukoencephalopathy (PML)
- Septicemia, recurrent, including non-typhoidal Salmonella
- Toxoplasmosis of brain/central nervous system
- Wasting syndrome due to HIV (involuntary weight loss > 10% of baseline body weight) associated with either chronic diarrhea (> 2 loose stools per day > 1 month) or chronic weakness and documented fever > 1 month
- Cervical carcinoma, invasive, confirmed by biopsy
- Kaposi Sarcoma
- Lymphoma (primary central nervous system/cerebral, B cell non-Hodgkin (confirmatory diagnostic testing required))
- Atypical disseminated leishmaniasis
- Symptomatic HIV-associated nephropathy
- Symptomatic HIV-associated cardiomyopathy
- Reactivation of American trypanosomiasis (meningoencephalitis or myocarditis)

WHO Stage 3 Clinical Events

- Acute necrotizing ulcerative stomatitis, gingivitis, or periodontitis
- Unexplained severe weight loss (> 10% body weight)
- Unexplained chronic diarrhea
- Unexplained persistent fever
- Oral candidiasis, persistent
- Oral hairy leukoplakia

- Pulmonary Tuberculosis
- Severe Bacterial Infections (other than recurrent bacterial pneumonia)
- Acute necrotizing ulcerative stomatitis, gingivitis, or periodontitis
- Unexplained anemia (hemoglobin <8 g/dL)
- Neutropenia (neutrophils <500 cells/µL)
- Chronic thrombocytopenia (platelets <50,000 cells/µL

WHO Stage 2 Clinical Events

- Moderate, unexplained weight loss (< 10% body weight)
- Upper respiratory tract infections, recurrent (sinusitis, tonsillitis, otitis media and pharnygitis)
- Herpes zoster
- Angular cheilitis
- Oral ulcerations, recurrent
- Papular pruritic eruptions
- Seborrhoeic dermatitis
- Fungal nail infections

Non-AIDS-Defining Cancers

- Lung Cancer
- Liver Cancer
- Anal Carcinoma
- Hodgkin's Lymphoma
- Oropharyngeal Carcinoma
- Melanoma
- Colorectal Carcinoma
- Breast Cancer
- Burkitt's Lymphoma

Other Targeted Medical Conditions

- Pulmonary Tuberculosis
- Severe Bacterial Infections (other than recurrent bacterial pneumonia)
- Diabetes mellitus
- Lipodystrophy (lipohypertrophy or lipoatrophy)
- Idiopathic thrombocytopenia
- Malaria
- Idiopathic thrombocytopenic purpura
- Sensory peripheral neuropathy
- Malignancy, newly diagnosed, excluding squamous cell and basal cell cancer of the skin
- Renal insufficiency
  - Acute
  - Chronic
- Liver disease
  - Cirrhosis
  - Idiopathic sclerosing cholangitis
- Lactic acidosis
- Symptomatic HIV-associated nephropathy

- Immune reconstitution inflammatory syndrome (IRIS)

Major Cardiovascular Disease Outcomes

- Hypertension
- Congestive heart failure
- Stroke
- Transient Ischemia Event (TIA)
- Pulmonary Embolism
- Myocardial Infarction (MI)
    - Acute symptomatic (non-fatal myocardial infarction (MI) requiring hospitalization)
    - Silent (diagnosed by serial Q-wave change on electrocardiogram (ECG))
- Coronary Artery Disease requiring percutaneous or surgical intervention
- Coronary Artery Disease requiring medical therapy
- Deep Vein Thrombosis
- Peripheral Vascular Disease
- Symptomatic HIV-associated Cardiomyopathy

APPENDIX V
SAMPLE INFORMED CONSENT FOR WOMEN WHO BECOME PREGNANT WHILE ON STUDY SUPPLIED STUDY DRUGS

Informed Consent Form – Women Who Become Pregnant While on Study-Supplied Study Drugs
IMPAACT 1077BF
Breastfeeding Version of the PROMISE Study
(Promoting Maternal and Infant Survival Everywhere)
Protocol Version 3.0, Dated 15 October 2012

Note to Sites: Version number and date of the protocol should be included on the first page of the consent form and the version number and date of the consent form should be included in a header or footer on each page of the consent form along with page numbering in the following format: Page 1 of x, Page 2 of x, Page 3 of x.

INTRODUCTION

Because you are now pregnant, you are being asked if you want to continue taking medications from the research study. If you were receiving antiretroviral medications from the study when you got pregnant, you need to receive information about what is known about use of these drugs in pregnancy and what your alternatives are before deciding if you want to continue the drugs.

This is a consent form. It gives you more information about the anti-HIV medications from the study and how they may affect your pregnancy and your unborn baby. The study staff will talk with you about this information. You may also talk with your own doctor about what is best for you and your baby. If you agree to stay on anti-HIV-medications provided through the study, you will be asked to sign this consent form. You will get a copy to keep. You are free to ask questions of the study staff at any time.

WHAT DO I HAVE TO DO IF I STAY ON THE anti-HIV MEDICATIONS FROM THE STUDY?

Whether or not you choose to stay on the anti-HIV medications from the study, you will continue to have study visits and tests as stated in the main study PROMISE consent form.

If you choose to continue taking anti-HIV medicines from the study, the study staff will talk more with you about the medicines you are taking and make recommendations about whether to keep taking those medicines or to switch to different medicines. The study staff will also tell you if the dose of your medicines should be changed while you are pregnant. If you are still pregnant at your last study visit, the study staff will contact you again to find out about the outcome of your pregnancy.

Care related to your pregnancy, the delivery and care of your baby will not be provided by this study. You must arrange for pregnancy-related care and your baby's care outside of this study. The study staff will talk to you about care that may be available *[Sites – include any locally relevant on provision of or referral for care.]*. Long-term follow-up is recommended for a baby whose mother takes anti-HIV drugs during pregnancy.

WHAT ARE THE RISKS OF CONTINUING TO TAKE HIV MEDICINES FROM THE STUDY?

The possible risks of taking part in this study were described in the consent form that you signed when you first joined the study. This form describes additional possible risks for you and your baby from taking HIV medicines during pregnancy.

<u>Risks to You</u>:

- Different side effects or more severe side effects may occur in pregnant women taking HIV medicines. This may make it more difficult to take your HIV medicines. Not taking your medicines as directed could cause the medicines to not work on the HIV in your body.
- The amount of HIV medicine in the blood may change during pregnancy. Because of this, the amount of medicine in your body may be decreased and the medicines may not work as well as usual. This could also cause the HIV in your body to become resistant. When resistance occurs, a medicine no longer works against HIV, which can limit the choices of HIV medicines that a person can take in the future.
- It is not known if some risks of pregnancy might be made worse by HIV medicines, possibly resulting in death.

Risks to Your Baby:
- It is not known if some HIV medicines may cause babies to be born early or dead.
- It is not known if some HIV medicines may cause babies to be sick or have birth defects. Not all birth defects are seen at birth. Some birth defects are seen later as the baby grows.

The World Health Organization recommends the use of several HIV medicines during pregnancy that are available through the study, including zidovudine (ZDV), lamivudine (3TC), tenofovir (TDF), and lopinavir-ritonavir (LPV-RTV). The clinical staff will describe the country-specific standard of care to prevent transmission of HIV from a mother to her baby during pregnancy and delivery. If you choose not to continue taking HIV medicines from the study while you are pregnant, it is important that you take HIV medicines from outside the study to decrease the risk of passing HIV to your baby.

ARE THERE BENEFITS TO STAYING IN THIS STUDY?

If you continue to take part in this study, there may be a benefit to you and your baby, but no guarantee can be made. It is also possible that you and your baby will receive no benefit from continuing in this study. Information learned from this study may help others who have HIV.

Anti-HIV drugs, whether taken as part of this study or through the government antiretroviral treatment (ART) program, can help decrease the chance of your baby becoming infected with HIV during pregnancy and delivery. These medications are used throughout the world for this purpose.

WHAT OTHER CHOICES DO I HAVE BESIDES STAYING ON STUDY DRUGS?

Instead of staying on the study drugs, you have the choice of receiving the standard drugs used locally from another program or provider outside the study to prevent passing HIV from a mother to her infant.

Please talk to your doctor about the choices available to you. Your doctor will explain the risks and benefits of these choices.

WHAT ABOUT CONFIDENTIALITY?

As explained when you agreed to join the study, efforts will be made to keep your personal information confidential. We cannot guarantee absolute confidentiality. Your medical records, name, address, and identification number will be kept in a locked room. Only the study staff will have the keys. No publication of this study will use your name or identify you personally.

Your records may be reviewed by the ethics committee that oversees research at this site, the US Food and Drug Administration (FDA), the study sponsor (the US National Institutes of Health) or its agents, the US Office of Human Research Protections, IMPAACT leadership (e.g., staff from the operations center, data

management center and network lab), local regulatory authorities, study staff, study monitors, and the drug companies supporting this study.

WHAT ARE THE COSTS TO ME?

In addition to any costs that are described in the study consent you already signed; this study will not cover any cost related to your pregnancy, delivery of your baby, or care of your baby. If you take HIV medicines from another program or provider outside the study, you will need to pay for the medicines, unless the medicines are available free of charge. The study cannot pay for medicines obtained from other programs or providers.

WILL I RECEIVE ANY PAYMENT?

You will receive reimbursement for 1077/PROMISE study visits as described in the original consent form you signed for the study.

WHAT HAPPENS IF MY BABY OR I AM INJURED?

It is possible that either you or your baby could experience a problem or injury that would not have occurred if you did not participate in this study. If [the study doctor] determines that you or your baby has been injured as a direct result of being in this study, you and/or your baby will be given immediate treatment for those injuries at no cost to you and then referred for further care if needed. *[Sites: add local information regarding treatment for injury]*.

However, [the study doctor] may determine that your or your baby's illness or injury would have happened even if you did not participate in this study. In that case, appropriate care and/or referral will likewise be provided for any illness or injury that occurs during the study *[Sites: Add local information regarding care/referral, and explain whether participants will bear the costs of treatment for non-study-related injury, or if there is some mechanism for covering these costs as well]*.

There are no plans to give you money if you or your baby experiences a complication, whether or not the problem or injury was related to study participation. You will not be giving up any of your legal rights by signing this consent form.

WHY MIGHT THE DOCTOR TAKE ME OFF THIS STUDY EARLY?

The study doctor may need to take you off the study early without your permission if the study is cancelled or stopped.

WHY MIGHT THE DOCTOR TAKE ME OFF THE STUDY DRUGS EARLY?

The study doctor may need to take you off the study drugs early for any of the reasons explained to you when you joined this part of the study.

WHAT ARE MY RIGHTS AS A RESEARCH PARTICIPANT?

Continuing to take part in this study is completely voluntary. You may choose not to continue in this study or leave this study at any time. If you leave the study, you will not be penalized or lose any benefits to which you would otherwise have access outside of the study.

We will tell you about new information from this or other studies that may affect your health, welfare or willingness to stay in this study. If you want the results of the study, inform the study staff.

WHAT DO I DO IF I HAVE QUESTIONS OR PROBLEMS?

For questions about this study or a research-related injury, contact:
- *[Sites: insert name of the investigator or other study staff]*
- *[Sites: insert telephone number and physical address of above]*

For questions about your rights as a research participant, contact:
- *[Sites: insert name or title of person on the Institutional Review Board (IRB) or other organization appropriate for the site]*
- *[Sites: insert telephone number and physical address of above]*

SIGNATURE PAGE

If you have read this consent form (or had it explained to you), all your questions have been answered, and you want to continue taking the study medications during your pregnancy, please sign your name below.

______________________________<br>Participant's Name (print)	__________________<br>Participant's Signature	____________<br>Date
______________________________<br>Study Staff Conducting<br>Consent Discussion (print)	_________________<br>Study Staff Signature	____________<br>Date
______________________________<br>Witness's Name (print)<br>(As appropriate)	_______________<br>Witness's Signature<br>(As appropriate)	____________<br>Date

APPENDIX VI
SAMPLE INFORMED CONSENT FOR SPECIMEN STORAGE AND FUTURE USE

Informed Consent Form – Specimen Storage and Future Use
IMPAACT 1077BF
Breastfeeding Version of the PROMISE Study
(Promoting Maternal and Infant Survival Everywhere)
Protocol Version 3.0, Dated 15 October 2012

Note to Sites: Version number and date of the protocol should be included on the first page of the consent form and the version number and date of the consent form should be included in a header or footer on each page of the consent form along with page numbering in the following format: Page 1 of x, Page 2 of x, Page 3 of x.

<u>Introduction</u>
You have decided that you and your baby will participate in this research study to help us find the best ways to prevent babies from getting HIV and to keep mothers and babies healthy. In addition to the tests that you have as part of the study, we are asking now for your permission to save any leftover blood, breast milk and cells from your blood and any of your baby's leftover blood and cells for future use. These specimens would be saved in a place called a repository, which is a special laboratory with freezers to store the specimens. There are no names on any of the specimens, only a special study number. The people who run the repository and the scientists who later use the specimens will not know your name or your child's name.

Researchers can learn a lot from a study but as time goes by the tests that they use get better or brand new tests are developed, and more can be learned with these better or new tests by using them on stored specimens. If a researcher wants to do a test on specimens from the repository in the future, he or she will write up the idea and it will have to be approved by the study team leaders and other groups to make sure that the research is worthwhile. If the idea is approved, then coded specimens and coded information will be given to the researcher. They would never know your name or your baby's name.

Because of the location of the repositories and/or the place where the tests will be conducted, these stored samples may be shipped to another country for storage and/or future use.

<u>What about confidentiality</u>?
There are no names on any of the specimens, only a special study number. The people who run the repository and the scientists who later use the specimens will not know your or your baby's name or any other information about you that might identify you. As explained when you agreed to join the study, your records may be reviewed by the ethics committee that oversees research at this site, the US Food and Drug Administration (FDA), the study sponsor (the US National Institutes of Health) or its agents, the US Office of Human Research Protections, IMPAACT leadership (e.g., staff from the operations center, data management center and network lab), local regulatory authorities, study staff, study monitors, and the drug companies supporting this study.

<u>How often will these specimens be collected</u>?
As described to you when you agreed to join the study, blood and breast milk will be collected for study tests at each study visit. After all testing that is planned to be done for the study has been completed, some of your and your baby's blood, your breast milk and cells from your or your baby's blood may be leftover. It is these leftover specimens that you are being asked to have stored for future use. You are not being asked to give additional specimens for long term storage.

<u>What kind of tests might be done on my or my baby's specimens?</u>
Tests that might be done include tests to see how much HIV is in the blood, what type of HIV it is and whether it is resistant to some of the anti-HIV drugs, how the body responds to HIV, how HIV causes disease, how HIV is transmitted from mother to baby, the levels of HIV drugs in the blood or breast milk, and how drugs cause side effects. The tests might also look at other infections like malaria or other conditions like diabetes

that people with HIV may get. The tests might look at how a person's genetic makeup (your DNA) either protects them or puts them at greater risk. This kind of information is important for scientists who are working on an HIV vaccine.

Will I get the results of these tests?
Most of the time, you will not get results from these tests. This is because research can take a long time and must use specimens from many people before results are known. Results from research using the specimens may not be ready for many years.

The researchers who use stored samples for a study approved by NIH will not contact you with the results of their tests as they use samples labeled only with codes and would not know who to contact. If their findings could provide important information for your or your child's medical care, then the investigators would contact the research staff at your site with the results, and the staff at your clinic can link the code with your name and notify you of the results. If you would like to be contacted with this sort of information, you must notify the study staff of any changes in your address or phone number.

How long will the specimens be stored?
There is no time limit on how long the samples will be stored.

What if I don't want my samples saved for future use?
You may decide that you do not want your samples stored for future research studies. You can still participate in this study even if you make this decision, any leftover specimens from you or your baby will be destroyed at the end of the study.

What if I agree to have my or my baby's specimens stored and then change my mind?
People always have the right to stop participating in research. So if you decide that you do not want researchers to be able to use the specimens in the repository, you can contact the clinic staff. They will tell the repository that the specimens with your study code number should not be studied, and these specimens will be destroyed. If you change your mind after your specimens have already been shipped for testing, the samples that have been shipped will still be tested but your specimens still remaining in the repository will be destroyed

What are the benefits to me and my baby from agreeing to store specimens?
There are no direct benefits to you or your child from storing your specimens. You may be helping people in the future from the results of studies using the stored specimens.

What are the risks to me and my baby from agreeing to store specimens?
These specimens are being collected as part of the PROMISE study in which you are participating. We are not asking you to give any additional specimens for storage, so there is no additional risk associated with collection. The specimens are stored only by code number (not your name or your child's name) so there is no risk of loss of privacy.

What are the costs to me?
There is no cost to you for having your or your baby's specimens stored.

Will I receive any payment?
You will not receive any payment for providing these specimens for storage. Your samples will not be sold or directly used to produce commercial products. In the future, some of the research may help to develop new products, such as tests and drugs. If this would happen and these tests or drugs make money, there are no plans to share that money with the people who gave the specimens

What do I do if I have questions or problems?
For questions about this study, contact:

- *[insert name of the site investigator or other study staff]*
- *[insert telephone number and physical address of above]*

For questions about your rights as a research participant, contact:

- *[name or title of person on the Institutional Review Board (IRB) or other organization appropriate for the site]*
- *[insert telephone number and physical address of above]*

SIGNATURE PAGE

I give permission for the storage and use of my child's stored specimens for future tests as discussed in this consent form, including genetic testing:

No ____ Yes ____

I give permission for the storage and use of my child's stored specimens for future tests as discussed in this consent form, EXCEPT for genetic testing

No ____ Yes ____

I refuse to have any specimen that was collected from my child stored in the repository.

No ____ Yes ____

____________________________	____________________________	_________
Mother's Name	Signature	Date
Infant's Father's Name<br>(if reasonably available)	Signature<br>(if reasonably available)	Date

I give my permission for the storage and use of my stored specimens for future tests as discussed in this consent form, including genetic testing.

No ____ Yes ____

I give my permission for the storage and use of my stored specimens for future tests as discussed in this consent form, EXCEPT for genetic testing.

No ____ Yes ____

I refuse to have any of my specimens stored in the repository.

No ____ Yes ____

____________________________	____________________________	_________
Participant's Name	Signature	Date
Name of Person Conducting<br>Consent Discussion	Signature	Date
Witness's Name<br>(if required)	Signature	Date

APPENDIX VII
IMPAACT 1077BF/FF HEPATITIS B SUBSTUDY
ANALYSIS AND MONITORING PLAN
Impact of HIV PMTCT Interventions on HBV Disease in HIV/HBV Co-infected Women and their Infants

IMPORTANT: This appendix describes plans for analyzing and monitoring data collected as part of IMPAACT 1077BF and IMPAACT 1077FF and is NOT for separate site implementation. All human subjects procedures and assessments are to be performed as part of the main studies and are described above in the main study protocols and informed consent forms.

Substudy Co-Chairs
Debika Bhattacharya, MD
Assistant Clinical Professor
David Geffen School of Medicine, UCLA
Division of Infectious Diseases 37-121 CHS
Los Angeles, CA 90095
Phone: (310) 206-0527
E-mail: debikab@mednet.ucla.edu

Judith Currier, MD, MSc
Professor of Medicine
UCLA CARE Center
Los Angeles, CA 90035
Phone: (310) 557-1891
Fax: (310) 557-1899
Email: jscurrier@mednet.ucla.edu

Marion Peters, MD
Professor of Medicine/Director - Hepatology Research
University of California, San Francisco
Gastroenterology Division
San Francisco, CA 94143-0538
Phone: (415) 476-2777
E-mail: marion.peters@ucsf.edu

Protocol Virologist
Wendy Stevens, MD
Professor
University of Witwatersrand
7 York Road, Park Town
Johannesburg, Gauteng 2193
South Africa
Phone: 27 11 489 8505
Email: wendy.stevens@nhls.ac.za

Investigator
Heather Watts, MD
Ped. Adolescent & Maternal AIDS Branch/CRMC
National Institute of Child Health & Human Development (NICHD)
Bethesda, MD 20892
Phone: (301) 435-6874
Email: wattsh@mail.nih.gov

Protocol Statisticians
Camlin Tierney, PhD
Center for Biostatistics in AIDS Research
Harvard School of Public Health
Boston, MA 02115
Phone: (617) 432-0547
E-mail: tierney@sdac.harvard.edu

Sharon Huang, MS
Center for Biostatistics in AIDS Research
Harvard School of Public Health
Boston, MA 02115
Phone: (617) 432-2873
Email: sharon@sdac.harvard.edu

SCHEMA: HBV SUBSTUDY ANALYSIS AND MONITORING PLAN

Impact of HIV PMTCT Interventions on HBV Disease in HIV/HBV Co-infected Women and their Infants

DESIGN This fully nested substudy will explore HBV disease outcomes among HIV/HBV co-infected women entering the PROMISE Antepartum Component (1077BA or 1077FA). Follow-up of these women (and their infants) will be through 1077BF or 1077FF. All human subject procedures and assessments are performed as part of the main studies and are described in the relevant sections of the main protocol and the schedules of evaluations and informed consent forms therein.

DURATION As part of the main study (1077BF or 1077FF) women will be followed until 96 weeks after the last woman in the Antepartum Component (of 1077BA or 1077FA) delivers (approximately 2-5 years, depending on rate of accrual/delivery in the Antenatal Component); infants will be followed through 104 weeks of age.

SAMPLE SIZE Assuming a prevalence of HBV co-infection between 3.5% and 7% within the main Antepartum Component population, an estimated 154-308 women and their infants will be included in the substudy.

POPULATION Women qualifying and consenting for the Antepartum Component randomization in 1077BF or 1077FF who are HBsAg+ and their infants will be included in the substudy. As described in the main study protocols, these mother-infant pairs will be followed regardless of their qualification for subsequent randomizations.

REGIMEN The Antepartum Component study drug regimens for all women (HBsAg+ and HBsAg-) are described in Section 2.0 of the main protocol. After delivery, these women and their infants will remain in study follow-up and may be eligible for the subsequent, post-delivery component randomizations in 1077BF or 1077FF, which will be identical for HBsAg+ and HBsAg- women; for details, refer to the schemas of 1077BP, 1077BM and 1077FM.

HYPOTHESIS AND SUB-STUDY ANALYSIS OBJECTIVES

Hypothesis After eight weeks on the triple ARV regimen, HIV/HBsAg+ co-infected pregnant women assigned in the main study to receive TDF/FTC/LPV-RTV will have larger decreases in hepatitis B viral load from baseline, when compared to women who were assigned to receive ZDV/3TC/LPV-RTV.

Primary Objective To compare the anti-HBV efficacy of antepartum ZDV/3TC/LPV-RTV versus TDF/FTC/LPV-RTV, assessed as change in hepatitis B viral load during the antepartum period.

Secondary Objectives

- To estimate and compare (among groups defined by the Antepartum Component randomization in 1077BF or 1077FF) vertical transmission of HBV and to describe HBV characteristics (including genotype, drug resistance, precore and core promoter mutants and DNA viral load) among infants contracting HBV and among transmitting mother-infant pairs

- To evaluate and compare (among groups defined by the Antepartum, Postpartum, and Maternal Health Component randomizations in 1077BF or 1077FF) maternal HBV DNA viral load levels and presence of HBV drug resistance at delivery and through up to four years post-partum

- To estimate HBV virologic, safety outcomes (LFT) and HBV serologic changes (specifically HBeAb and HBsAb seroconversion and seroreversion) over time following anti-HBV ARV regimen cessation

- To estimate and compare (among groups defined by the Antepartum Component randomization in 1077BF or 1077FF) maternal anemia at delivery

1.0 INTRODUCTION

1.1 Background and Rationale (HBV Substudy)

Hepatitis B virus (HBV) coinfection is common; affecting greater than 10% of HIV-infected individuals in some resource-limited settings (1-3). Although the impact of HIV disease on HBV coinfection has been studied in non-pregnant adults, little is known about the effects of HIV on HBV during pregnancy, particularly the optimal short-course antepartum HAART regimen in HIV/HBV coinfection. Accordingly, in its 2009 consensus statement on hepatitis B, the NIH identified the study of the risks and benefits of antiviral therapy in pregnancy as a top research priority (4).

However, in many resource-limited settings, HBV screening is not available and HIV/HBV co-infected pregnant women subsequently receive various regimens of HBV-active PMTCT regimens. Additionally, although US and WHO guidelines recommend the use of two drugs active against HBV in co-infected patients starting HAART (5), this standard has not been routinely applied to pregnant women. Specifically, uncertainty about the safety of TDF in pregnancy has limited the widespread use of dual HBV-active HAART therapy in this setting.

Hepatitis B viremia in the antenatal period is a key prognostic factor for HBV vertical transmission; (6-8) transmission occurs despite infant immunoprophylaxis in women with high HBV antepartum viral loads (7, 9). In Lee (8), within the context of infant immunization, HBV DNA detection in maternal serum was independently associated with transmission, even after controlling for HBe-antigen status (approximately 57% of mothers with detectable HBV DNA transmitted versus 0% with undetectable levels, in both E-antigen positive and E-antigen negative subgroups).

HBV is endemic in resource-limited settings and, in these settings, pregnant women often present late to antenatal care, highlighting the need to identify effective, short-course therapies for HBV PMTCT. Some studies that have examined short course antepartum lamivudine (single HBV active therapy) as an adjunct therapy to immunoprophylaxis in HBV PMTCT have demonstrated low rates of HBV virologic suppression and high rates of HBV transmission (9). Combination therapy for HBV may result in greater decreases in HBV DNA viral load and more rapid virologic suppression.

As HIV/HBV co-infected women receive triple ARV regimens for PMTCT of HIV, it will be crucial to evaluate strategies for optimal short-term HBV virologic reduction in a population with a potentially greater risk of HBV vertical transmission.

As its primary objective, this substudy will compare the efficacy of the HIV PMTCT regimens of antepartum ZDV/3TC/LPV-RTV (single HBV-active therapy) vs. TDF/FTC/LPV-RTV (combination HBV-active

therapy) on HBV DNA viral loads after eight weeks. By comparing Week 8 antepartum HBV viral load changes between single and combination HBV therapy, this substudy will help establish the optimal short-course HBV regimen for HBV virologic suppression, a key predictor of HBV vertical transmission.

In the context of the large PROMISE studies (1077BF and 1077FF), we will investigate HBV outcomes, specifically changes in HBV DNA viral loads during the antepartum period (primary endpoint at 8 weeks of dosing), mother to child transmission of HBV, maternal HBV drug resistance at delivery, HBV virologic and biochemical changes after cessation of the triple ARV regimen, and maternal anemia at delivery among HIV/HBV co-infected women entering 1077BF and 1077FF.

Hepatitis B Viremia Influences HBV Vertical Transmission
The risk of HBV vertical transmission is increased with elevated maternal HBV DNA levels in pregnancy (6-8). Xu and colleagues demonstrated that in women with high antepartum HBV DNA levels, HBV vertical transmission occurred despite immunoprophylaxis with vaccine and hepatitis B immunoglobulin, with transmission rates as high as 39% (9). This is of particular relevance in HIV infection, where elevated HBV DNA levels in pregnancy are more prevalent than in HBV infection alone (10). Therefore, it will be critical to identify methods to reduce antepartum HBV viremia in HIV infection, especially given the importance of HBV viremia in perinatal transmission.

Association of Pregnancy and HIV Disease with Hepatitis B Viremia
Pregnancy and its relative immunosuppression may affect HBV viral load levels; in one study, elevated HBV viral loads were found in 25% of HBeAg-negative mothers (7), the time period before which the majority of HBV infant transmission occurs. HIV induced immunosuppression, in turn, is associated with higher HBV viral loads. In a study comparing HIV/HBV co-infected and HBV monoinfected men, HIV/HBV coinfected men had HBV DNA levels of 200 pg/mL, compared to 86 pg/mL in HBV monoinfection (11). In the only study to compare HIV/HBV and HBV infected pregnant women, Rouet and colleagues demonstrated that HIV-infected women had a higher prevalence of detectable HBV viremia (27% in co-infected vs. 7% in HBV mono-infected) (10). It is important to note that the lower limit of detection in this study was 375,000 copies/mL (2.5 pg/mL).

Antepartum ARV Regimens
Although nucleoside analogues have been a suggested intervention for the PMTCT of HBV in women with high HBV viral loads, there are still few published data to support this strategy. Antenatal hepatitis B antiviral therapy in the last four to eight weeks of pregnancy for HBV PMTCT has been evaluated in three published studies (9, 12, 13), only one has been a randomized clinical trial(9). The largest study examining nucleoside analogues (specifically lamivudine) for the prevention of HBV transmission in HIV uninfected pregnant women involved 155 women (9). In this study, HBV virologic suppression to less than 10x5 copies/mL occurred in only (12/89) 13% of lamivudine-treated pregnant women and HBV vertical transmission occurred in 18% of infants treated with lamivudine, despite immunoprophylaxis with HBV vaccine and immunoglobulin. Given the high prevalence of HBV viremia and subsequent HBV vertical transmission despite antepartum single HBV-active lamivudine therapy in women with high HBV viral loads, it will be important to assess the effect of dual, or combination, HBV therapy on HBV virologic suppression.

Combination Therapy for Hepatitis B
Long-term combination nucleos(t)ide therapy is advocated in HIV/HBV coinfection to prevent the emergence of HBV drug resistance (14), a consequence of long-term HBV monotherapy. *However, the virologic response to short-term combination therapy on HBV, critical in the antepartum phase to decrease HBV transmission, is not well defined.* There are no large-scale, comparative studies examining short-term virologic response between 3TC and TDF+FTC in HIV/HBV coinfection. Data are instead obtained from smaller studies. In one study of HIV/HBV coinfection, TDF+FTC was associated with a 3-log decrease in wild type HBV viral load at week 4 (15). In contrast, HBV monoinfected patients experienced a mean 2-log decline in HBV DNA after being treated with 3TC (9). In 21 Thai patients, TDF+3TC, when compared to

3TC alone, was associated with a 0.6 log greater difference in HBV VL decline at week 12, but this did not reach statistical significance (16).

Rationale for Eight-Week Viral Load Primary Endpoint
This substudy will examine hepatitis B viral load changes after eight weeks on the antepartum ARV regimen and compare responses to women randomized to ZDV/3TC/LPV-RTV versus TDF/FTC/LPV-RTV. This eight week time frame is particularly important for two reasons. First, when antepartum antiviral therapy is considered in HBV infection for HBV PMTCT, regimens are initiated in the third trimester (17); thus, it will be important to investigate the durations that are currently standard of care in HBV monoinfection guidelines. Second, in resource-limited settings, most HIV-infected women present to antenatal care late in pregnancy, thus it will be critical to identify an appropriate short-course antenatal regimen. This study will evaluate the efficacy of short-course single versus dual HBV therapy (as part of the HIV antiretroviral regimen for PMTCT) using HBV viral load level as the measure of efficacy. (Note: If a sufficient number of women have exposure to ARVs for longer than eight weeks, we will also assess the proportion of women with undetectable DNA at delivery as a secondary endpoint. However, we have chosen eight weeks for the primary endpoint based on the assumption that most women will have this duration of exposure.)

Rationale for Randomization to a Non-HBV Active Antepartum Regimen
HIV/HBV co-infected women enrolling into 1077BF and 1077FF will be randomized as described above in the main study protocol.

As noted above, the primary substudy analysis will compare the two triple ARV arms (B and C) with respect to HBV viral load changes after 8 weeks of dosing. Secondary substudy analyses will assess HBV viral load at delivery and HBV vertical transmission between all three regimens in the main study as it will be important to establish HBV virologic changes and vertical transmission in the absence of HBV therapy.

Although HBV-active HAART is recommended for the long-term therapy of HBV in HIV infection (14), the role of short course HBV-active triple ARV drugs in pregnancy is not clear. The WHO guidelines recommend use of two drugs active against HBV (e.g., TDF + 3TC) for pregnant women with HBV coinfection who require HBV treatment but acknowledge the limited data about potential maternal and infant bone toxicity with use of TDF. The rationale for HBV treatment in HIV infection is based on the accelerated frequency of complications of long-standing untreated HBV infection such as cirrhosis and hepatocellular carcinoma (14). It is unclear whether this rationale can be extrapolated to the short-term management of HBV in a population of HIV/HBV-coinfected women who may be later randomized to TDF/FTC-LPV-RTV, a long-term HBV-active triple ARV regimen. HBV management guidelines do not routinely recommend the treatment of HBV during pregnancy (18) while US HIV perinatal guidelines include a regimen of non-HBV active ARVs (i.e., ZDV+ddI) in the management of HIV/HBV co-infected pregnant women (19).

Implications for HBV Management
Approximately 154-308 women are expected to be included in this substudy - the largest randomized investigation to evaluate nucleoside therapy in pregnant, HBV-infected women. If this substudy demonstrates that short course dual-HBV active therapy is superior to single-HBV active therapy in short-term HBV virologic reduction, then this strategy may be considered for all HBV-infected women as a strategy to decrease antenatal maternal HBV viremia and subsequent HBV vertical transmission.

Rationale for Secondary Endpoints

- <u>Mother-to-Child Transmission of HBV</u>
  Without immunoprophylaxis, >75-90% of infants born to HbsAg+ and HBeAg+ mothers will develop chronic hepatitis B infection (20-22). Immunization with HBIG and HBV vaccine reduces the risk of transmission to <10% (23) while immunization with HBV vaccine alone reduces the risk of transmission to <15% (24). However, in women with elevated HBV viral loads, as may be anticipated in HIV/HBV co-

infected women, vertical transmission can occur despite lamivudine and immunoprophylaxis (25). Because we expect a range of maternal HBV viral loads (low and high), we expect that the overall HBV transmission rates will be low. In studies that examined HBV vaccine alone for PMTCT, the vaccine prevented transmission in 75-90% of HbsAg+/HBeAg+ women. Although the overall HBV transmission rates are expected to be low, this study will evaluate the association between maternal HBV viral load and infant vertical transmission of HBV, in the setting of antiviral therapy and immunoprophylaxis. HBV is not thought to be transmitted by breastfeeding. In a cohort of 369 infants born to HBsAg+ mothers and who received HBIG and the first dose of vaccine at birth, followed by vaccine at 1, and 6 months, HBV vertical transmission occurred in 0% of breast-fed infants and 3% of formula fed infants (26).

All infants of HIV/HBV co-infected mothers participating in 1077BF or 1077FF are to receive the complete HBV vaccine series, with the birth dose administered within 24 hours of birth, regardless of maternal randomization arm. HBV vaccine will be provided locally as standard of care for infants of mothers with HBV or purchased with study-related funding, if necessary.

- HBV Resistance in HIV/HBV Coinfection

  In HIV infection, sdNVP for PMTCT results in the rapid evolution of NVP resistance, often detected as minority variants, with the potential for decreased response to future NVP-based HAART in NVP-exposed women (27). In HBV disease, it is unknown whether short course therapy with single drug HBV-active HAART (e.g., containing 3TC as the only HBV-active agent) for PMTCT will result in similar 3TC resistance.

  3TC resistance also compromises future HBV virologic response to some, but not all, alternate HBV agents. In HBV monoinfection, 15-19% of 3TC-resistant patients develop resistance to adefovir at 2 years (28), compared to just 2% of 3TC-naïve patients. Entecavir resistance occurs at an even higher rate, occurring in 32% of 3TC-resistant patients after 3 years of therapy (29, 30). Conversely, with subsequent tenofovir containing regimens, HBV virologic suppression is not compromised. In a 5 year analysis of HIV/HBV coinfected patients receiving tenofovir-based antiretroviral therapy, there was no difference in virologic response between patients with and without baseline lamivudine resistance (p=0.39) (31).

  During extended therapy, 3TC resistance occurs at a higher rate in HIV/HBV co-infected individuals, occurring in 90% of HIV/HBV co-infected patients at four years of 3TC-based therapy (32) compared to 66% in HBV monoinfection (33). In HIV infection, HBV 3TC resistance has been associated with fulminant hepatitis in some patients (34, 35).

- Maternal safety of antepartum triple ARV regimens containing ZDV vs. TDF

  Anemia during pregnancy is associated with an increased risk of low birth weight, preterm delivery, and perinatal mortality (36, 37). Anemia, in turn, is a common side effect of ZDV, a critical component of short-course and maternal ARV regimens for PMTCT. In resource-limited settings, moderate to severe anemia occurred in 5-9% of women on short-course ZDV regimens (38-40). In ZDV-containing regimens, moderate or severe anemia was present in 20% of women at delivery(41). In a meta-analysis of ZDV PMTCT trials, late ART initiation with ZDV was associated with an odds ratio of 2.0 for anemia (42). Rates of anemia for TDF and ZDV-containing treatment have been compared in randomized trials of non-pregnant adults. In Gilead 934, none of the subjects receiving TDF+FTC+EFV developed anemia while 14 (5.5%) of 254 subjects receiving ZDV+3TC+EFV experienced a decline in hemoglobin from a median of 14 g/dL to a median 7 g/dL ; anemia was the most common reason for regimen discontinuation (43). Still unknown is whether similar rates of anemia will be observed during the last trimester in pregnancy, when comparing TDF and ZDV-containing triple ARV regimens for HIV prophylaxis .

The substudy analyses will include the subset of 1077BF and 1077FF women who are HBsAg+ and randomized to one of three antepartum intervention arms in 1077BF or 1077FF. Both the women and their infants will be followed according to the schedules of evaluations in the main study protocol. This substudy

analysis and monitoring plan includes no additional human subjects participation requirements beyond those specified in the main study protocols; all relevant assessments and evaluations are included therein.

2.0 STUDY DESIGN (HBV Substudy)

The HBV substudy will explore HBV outcomes (specifically HBV DNA virologic changes during the antepartum phase, HBV mother-to-child transmission, HBV resistance and HBV virologic and serologic changes after triple ARV regimen cessation), in addition to examining the safety endpoint of anemia at delivery among HIV/HBV co-infected women entering the main study, 1077BF or 1077FF.

As part of the main studies, all women screening for the Antepartum Component of 1077BF and 1077FF will be tested for active HBV infection by HBsAg. Eligible HbsAg+ women who consent to enrollment in 1077BF or 1077FF will be identified for inclusion in the substudy analyses via stratification factor (rather than a separate enrollment) and will be randomized as described in the main study protocol (with 1:1:1 allocation) to one of three antepartum arms: ZDV + sdNVP/TRV (Arm A); ZDV/3TC-LPV-RTV (Arm B); or TDF/FTC-LPV/RTV (Arm C).

All HBsAg+ women who enroll in either 1077BF or 1077FF will be included in the substudy; there is no separate substudy enrollment. HBsAg+ women will be followed in 1077BF or 1077FF for the same duration as HBsAg- women. As described in the main study protocols, after the Antepartum Component, eligible women (HIV/HbsAg+ and HBsAg-) may be randomized to the post-delivery components of 1077BF or 1077FF (the Postpartum Component (1077BP) and/or the Maternal Health Component (1077BM)), which are the same for HBsAg+ and HBsAg- women.

As part of the main study protocols, HIV/HBV coinfected women who discontinue their triple ARV regimen postpartum (those not eligible for 1077BP, 1077BM or 1077FM or those randomized to the NVP arm in 1077BP) will be followed according the SoEs in the main study after stopping their triple ARV regimen to assess for clinical or laboratory evidence of HBV flare. Also as part of the main studies, women who discontinue their triple ARV regimen as part of 1077BM or 1077FM will be followed according the SoEs in the main study protocols after stopping their triple ARV regimen to assess for clinical and laboratory evidence of HBV flare.

3.0 SELECTION AND ENROLLMENT OF SUBJECTS

Among women enrolled to the Antepartum Component of the main study (1077BF or 1077FF), only those women who are surface antigen positive for HBV during study screening, and their infants, will be included in the substudy analyses.

As part of the main studies, all women screening for the Antepartum Component of 1077BF and 1077FF will be tested for HBV infection by HBsAg. All HbsAg+ women who are enrolled in 1077BF or 1077FF (and their infants) will be included in the substudy. These women will be identified via stratification factor as there is no separate substudy enrollment.

Other inclusion and exclusion criteria, concomitant medications, prohibited medications, and all enrollment and follow-up procedures and assessments are described in the relevant sections in main study protocols, SoEs and informed consent forms.

4.0 STUDY TREATMENT

4.1 Regimens, Administration, and Duration

In the Antepartum Component of PROMISE (1077BA or 1077FA), all eligible women (HBsAg+ and HBsAg-) will be randomized in a 1:1:1 allocation to one of the three arms as described in the main protocol (Section 2.0). The regimens, administration and duration of dosing are described in the main study protocol.

5.0 EVALUATIONS FOR SUBSTUDY ANALYSES

5.1 Clinical and Laboratory Assessments for HBV Substudy Analyses

All clinical and laboratory assessments for the HBV substudy are undertaken as part of the main study and specified in the main protocol, schedules of evaluation and informed consent forms.

Women in the HBV substudy and their infants will be followed according to the same schedules of evaluations as HbsAg-negative women and their infants. As specified in the main protocol schedules of evaluation, some evaluations specific to the HBV substudy are included. Real-time evaluations versus assays that will be performed retrospectively using batched testing are indicated therein. A hepatitis-specific driven event is a clinically significant event suggestive of an acute exacerbation of hepatitis including any grade 2 or higher liver function test result (symptomatic or asymptomatic) and any of the following signs/symptoms (regardless of liver function test results): scleral icterus, jaundice, petechiae, otherwise unexplained abdominal pain, abdominal swelling, lower extremity swelling, fever occurring with any of these signs/symptoms.

5.2 Discontinuation of Triple ARV Regimen in HBsAg+ women

HBsAg+ women who discontinue their triple ARV regimen may be at risk of rebound HBV viremia and subsequent transaminitis. In the STACCATO HIV treatment interruption trial, 5/6 HIV/HBV co-infected patients who stopped ART developed HBV viremia and transaminitis and 1/6 had a severe flare (44). Additional case reports have also documented transaminitis after HBV-active ART cessation, one resulting in severe hepatitis (45).

HIV/HBsAg+ women who discontinue their triple ARV regimen as part of the main study will have transaminases measured at the time points specified in the SoEs of the main protocol after discontinuation. If, after triple ARV regimen cessation, liver function tests (transaminases or total bilirubin) are Grade 3 or above or if women are symptomatic (e.g., jaundice, severe fatigue), women may be considered for ART re-initiation following discussion with CMC, which includes HBV substudy clinicians.

6.0 STATISTICAL CONSIDERATIONS (HBV Substudy)

6.1 General Design Issues

This document describes analyses and monitoring of data collected through the PROMISE studies (1077BF and 1077FF) among HIV/HBV co-infected women and their infants. Co-infection with HBV will be defined as HBsAg positivity at study screening. The substudy analyses will focus on scientific questions unique to this subpopulation, such as whether short-course use of two anti-HBV agents (i.e. Arm C) can reduce HBV DNA viral load levels more than short-course 1 anti-HBV agent (i.e. Arm B) during the antepartum phase. While the primary objective and its associated statistical hypothesis test for primary group comparison is between only two of the three randomized arms from the Antepartum Component of 1077BF and 1077FF, secondary analyses of the primary endpoint, as well as secondary objectives and their associated analyses and comparisons will include data from women randomized to Arm A (no anti-HBV drugs in antepartum period). Data from HIV/HBV co-infected women enrolled in 1077BF and 1077FF will be combined for analysis. In

general, data from women and their infants collected during any component will be combined for analysis in the HBV substudy.

Substudy outcomes and endpoints can be arranged into four distinct groups: antepartum, postpartum, during anti-HBV therapy (which may include antepartum and postpartum follow-up), and post-cessation (of HBV therapy). Antepartum outcomes include the following: HBV viral load, HBV resistance, and maternal anemia at delivery. Postpartum outcomes include longer-term follow-up of: HBV acquisition by infants born to co-infected mothers, as well as maternal HBV viral load and HBV drug resistance during HBV therapy. During anti-HBV therapy includes the hepatotoxicity outcome on women. Post-cessation outcomes include shorter term follow-up of: HBV (viral loads and e-antigen and e-antibody status), and safety responses (specifically ALT levels and clinical manifestations of HBV).

The primary efficacy endpoint is antepartum change in HBV DNA viral load from pre-triple ARV regimen levels (baseline) to Week 8 of dosing. Relevant comparison groups for secondary endpoints are discussed below in the secondary analysis section.

6.2 Endpoints (HBV Substudy)

6.21 Primary Endpoint (antepartum)

- Antepartum change in HBV DNA viral load between Week 8 and baseline levels (using log HBV DNA)

Note: This endpoint is evaluable only among the subset of women who have detectable HBV DNA Viral load levels at baseline.

6.22 Secondary Endpoints

Antepartum endpoints:

Efficacy:
- Antepartum change in HBV DNA Viral load between week 4 and baseline
- Proportion of women with detectable HBV DNA at delivery

Safety:
- Presence of maternal anemia at delivery, measured as < 10 mg/dL
- Antepartum change in hemoglobin level from baseline to delivery
- Maternal hepatotoxicity (defined as grade 3 or 4 elevations in AST/ALT)

Post-partum endpoints:

- HBV positivity in infants, defined as positive HBV PCR any time up to 12 months of age
- Maternal HBV drug resistance -- measured at labor and delivery, and at years 1-4 postpartum. Resistance mutations will be defined based on literature at the time of analysis and expert opinion.
- Maternal HBV virologic suppression, (i.e., < 200 IU/mL using Roche assay), at labor and delivery and at years 1-4 postpartum

During anti-HBV ARV therapy endpoints (safety):

- Maternal hepatotoxicity (defined as grade 3, 4 elevations in AST/ALT)

Post-cessation (of anti-HBV ARV therapy) endpoints (safety):

- Changes in maternal HBV DNA viral load in plasma from last measurement during anti-HBV ARV therapy to measurements within 3 months following cessation of an anti-HBV ARV regimen
- Changes in maternal transaminase (specifically ALT and AST) levels from last measurement during anti-HBV ARV therapy to measurements within 3 months following cessation of an anti-HBV ARV regimen
- Changes in HBV serology (specifically HBV E antigen and E antibody) from to last measurement during anti-HBV therapy measurements within 3 months following cessation from an anti-HBV ARV regimen

Tertiary endpoints (evaluated only among HBV positive infants from samples collected during first year of life (see secondary endpoint on vertical transmission above), and their mothers – unless already defined above):

- HBV genotype
- Presence of HBV drug resistance
- Presence of precore and core promoter mutants
- Presence of detectable HBV plasma DNA viral load (and changes over time)

6.3 Sample Size and Accrual (HBV Substudy)

Substudy Sample Size at Antepartum Component Randomization:
A total of 4,400 women are expected to enter the Antepartum Component of 1077BF or 1077FF. As described above, all HBsAg+ women enrolled in 1077BA or 1077FA (and their infants) will be included in this substudy. Based on the HBV prevalence observed in the study population over the first year of study implementation, we anticipate that between 154 and 308 women will be included in the substudy.

Effective Sample Size:
Study design feasibility considerations are included below for two alternate estimates of HBV prevalence, 7% and 3.5% (in Tables 1 and 2, respectively).

The tables below provide estimations regarding the number of women who will have the primary efficacy endpoint available for analyses, acknowledging that the primary endpoint comparison will only utilize 2/3 of this number (i.e. those assigned to arms B and C). Parameters include a) proportion of women from the main study who are HBV surface antigen positive (assumed to be 7% in Table 1 and 3.5% in Table 2), b) sample size inflation/adjustment for contingency of interim monitoring (2%), c) proportion of women who are lost-to-follow-up for the primary endpoint – two main ways for this to occur are either women present late and therefore cannot be expected to deliver more than 8 weeks after randomization, or women deliver early (total LFU proportion assumed to between 5 and 10 %), d) proportion of HBV positive women who are E-antigen positive, c) proportions of women who have detectable HBV viral loads at baseline (prior to or at randomization), within each subpopulation defined on E-antigen positivity.

These last 3 parameters are introduced into the effective sample size calculation because changes in HBV DNA viral load cannot be calculated among women who enter the study with left-censored (i.e. undetectable) viral loads. The most recent versions of available assays, which are designed to detect HBV DNA viral loads at low levels (e.g., to 200 IU/mL), will be used to minimize this censoring problem, but it cannot be assured that the proportion undetectable at baseline will be negligible. Also, because previous research has suggested that HBV DNA viral load levels are associated with E-antigen status (7, 8, 10) the calculations below allow different detection rates within these subgroups.

In Rouet, 22% of HIV/HBV co-infected pregnant women from Cote D'Ivoire were E-antigen positive, and so we consider proportions between 20% and 30%. From mono-infected pregnant women tested early and late in pregnancy (7) using a modern HBV DNA viral load assay, all E-antigen positive women had detectable levels, and approximately 75% of E-antigen negative women had detectable HBV DNA levels in late pregnancy. Therefore, we assume 100% of E-antigen positive women in this study will have detectable baseline HBV DNA levels, and a range from 55-75% of E-antigen negative women will have detectable levels. If more women are E-antigen positive, or the proportion of E-antigen negative women with detectible levels is higher, then the effective sample size will be larger, and so these estimates may be considered conservative.

The different scenarios presented in Tables 1 and 2 below suggest a range of effective sample sizes in all three arms. Therefore the power calculations below consider the both the lower and upper bounds of this interval, as well as an approximation of the midpoint. With an estimated HBV prevalence of 7%, the range of effective sample sizes is from 182 to 246 with a midpoint of 215. With an estimated HBV prevalence of 3.5%, the range of effective sample sizes is from 91 to 123 with a midpoint of 108. With a prevalence of 7%, a sample size of 214 results from 20% of the study population being E-antigen positive (and all of these being HBV DNA detectable at baseline), 65% of the E-antigen negative women being detectable, and 5% LFU of women prior to assessment for the primary endpoint at antepartum Week 8. If the prevalence of HBV among enrollees to the Antepartum Component of the main study is lower, then the effective sample size available in this substudy will be reduced accordingly, as shown in Table 2 using an estimated prevalence of 3.5%.

Power calculations for the primary efficacy endpoint:
For estimated HBV prevalence of 7% and 3.5%, respectively, Tables 3 and 4 below show the differences (in standard deviation units) detectable with 80% and 90% power for hypothesis testing for the primary objective of comparing HBV antiviral activity between the Arm B (ZDV/3TC/LPV-RTV) versus Arm C (TDF/FTC/LPV-RTV). The hypothesis testing framework is superiority (null hypothesis of no difference between the two groups) and uses a t-test to compare groups. Assumptions needed for this test include the following: the within group standard deviation of the endpoint which is assumed to be the same in each group; the minimum clinically meaningful difference between groups, effective sample size assumed to be split approximately equally between groups, and significance level of 5%. As there are not good data on the standard deviation of changes in HBV viral load among this population, a range of estimated variabilities has been used.

With a HBV prevalence of 7%, the minimum difference between groups is 0.47; with a prevalence of 3.5%, the minimum difference between groups is 0.67.

As shown in Table 3, under an assumption of 7% prevalence and a total sample size of 214 participants (on all three arms, and the assumptions below), the primary endpoint comparison between Arms B and C will have 80% power to detect mean differences of 0.59, 0.47, or 0.35 if the standard deviation is 1.25, 1.0, or 0.75, respectively. The same sample size will provide 90% power to detect mean differences of 0.68, 0.54, or 0.41 if the standard deviation is 1.25, 1.0, or 0.75, respectively. Therefore, assuming this sample size and this range of standard deviations, the substudy is well powered to detect differences on the order of approximately 1/2 to 2/3rd of the standard deviation of the endpoint.

If the sample sizes are reduced due to lower observed prevalence of HBV positive women enrolling, then the minimum mean difference detectable with adequate power is increased. As shown in Table 4, for the instance of an observed prevalence of 3.5% and a total effective sample size of 108 participants (on all 3 arms, and the assumptions below), the primary endpoint comparison between Arms B and C will have 80% power to detect mean differences of 0.84, 0.67, or 0.50 if the standard deviation is 1.25, 1.0, or 0.75, respectively.

Table 1: Effective Sample Size estimates for Primary Endpoint - HBV prevalence = 7%

Interim monitoring adjustment= 0.02

		Proportion HBV E antigen positive								
		0.20			0.25			0.30		
		HBV DNA viral load detection among E+			HBV DNA viral load detection among E+			HBV DNA viral load detection among E+		
		1.0			1.0			1.0		
		HBV DNA viral load detection among E-			HBV DNA viral load detection among E-			HBV DNA viral load detection among E-		
		0.75	0.65	0.55	0.75	0.65	0.55	0.75	0.65	0.55
Proportion HBV surface antigen positive	Proportion LFU (late presenters + early deliverers)									
0.07	0.05	238	214	190	242	219	197	**246**	226	204
	0.07	234	210	187	238	**215**	194	241	220	200
	0.1	228	205	**182**	231	210	189	234	213	194

Note: These sample sizes are for all 3 study arms; in the primary endpoint comparison, the sample size will be 2/3 of the values in this table. Bolded value in the table represent the smallest, largest and midpoint (i.e., range) of effective sample sizes.

Table 2: Effective Sample Size estimates for Primary Endpoint - HBV prevalence = 3.5%

Interim monitoring adjustment= 0.02

		Proportion HBV E antigen positive								
		0.20			0.25			0.30		
		HBV DNA viral load detection among E+			HBV DNA viral load detection among E+			HBV DNA viral load detection among E+		
		1.0			1.0			1.0		
		HBV DNA viral load detection among E-			HBV DNA viral load detection among E-			HBV DNA viral load detection among E-		
		0.75	0.65	0.55	0.75	0.65	0.55	0.75	0.65	0.55
Proportion HBV surface antigen positive	Proportion LFU (late presenters + early deliverers)									
0.035	0.05	119	107	95	121	110	99	**123**	112	102
	0.07	117	106	94	119	**108**	97	121	111	100
	0.1	113	102	**91**	115	104	94	115	106	96

Note: These sample sizes are for all 3 study arms; in the primary endpoint comparison, the sample size will be 2/3 of the values in this table. Bolded values in the table represent the smallest, largest and midpoint (i.e., range) of effective sample sizes.

Table 3: Differences detectable for Primary Endpoint of Change in HBV DNA viral load between baseline and Week 8 antepartum (<u>with 7% prevalence assumption</u>); 2-sided hypothesis test (T-test) between ZDV/3TC and FTC/TDF arms

Alpha: 0.05

		Mean difference (log10 IU/mL) between ZDV/3TC and FTC/TDF groups detectable with specified power and total study sample size		
		**Total SS=182**	**Total SS=215**	**Total SS=246**
		**Sum**	**Sum**	**Sum**
**Power**	**Standard deviation of wk 8 change in HBV viral load within arm (log10 IU/mL)**			
**0.800**	**1.25**	0.64	0.59	0.55
	**1.00**	0.51	**0.47**	0.44
	**0.75**	0.38	0.35	0.33
**0.900**	**1.25**	0.74	0.68	0.64
	**1.00**	0.59	0.54	0.51
	**0.75**	0.44	0.41	0.38

Total Study Sample Size (i.e., adjusted for loss to endpoint evaluation, IM, and HBV undetectability at baseline), in all 3 groups, noting that power calculations used 2/3 this size because only 2 of 3 arms being compared

Table 4: Differences detectable for Primary Endpoint of Change in HBV DNA viral load between baseline and Week 8 antepartum (<u>with 3.5% prevalence assumption</u>); 2-sided hypothesis test (T-test) between ZDV/3TC and FTC/TDF arms

Alpha: 0.05

		Mean difference (log10 IU/mL) between ZDV/3TC and FTC/TDF groups detectable with specified power and total study sample size		
		**Total SS=92**	**Total SS=108**	**Total SS=123**
		**Sum**	**Sum**	**Sum**
**Power**	**Standard deviation of wk 8 change in HBV viral load within arm (log10 IU/mL)**			
**0.800**	**1.25**	0.90	0.84	0.78
	**1.00**	0.72	**0.67**	0.63
	**0.75**	0.54	0.50	0.47
**0.900**	**1.25**	1.05	0.97	0.91
	**1.00**	0.84	0.77	0.72
	**0.75**	0.63	0.58	0.54

Total Study Sample Size (i.e., adjusted for loss to endpoint evaluation, IM, and HBV undetectability at baseline), in all 3 groups, noting that power calculations used 2/3 this size because only 2 of 3 arms being compared

6.4 Randomization

Randomization will be performed as part of the main study protocols as described in Section 6.0 of the 1077BF protocol and Section 4.0 of the 1077FF protocol.

6.5 Monitoring (HBV Substudy)

Routine on-study monitoring, which will be performed by the substudy team (or a subset of the study team), includes the following: accrual, study status/progress, safety (i.e. maternal anemia, HBV flares, collapsed over study arm), data (and specimen collection) timeliness, quality and completeness.

As requested by the DMSB, one interim review of HBV outcomes is planned. After the first 100 HBV/HIV co-infected women are enrolled in 1077BA or 1077FA (and therefore included in the substudy), baseline maternal samples are to be shipped and tested for HBV E-antigen and HBV viral load levels. These data will then be summarized and presented to the DSMB. Comparisons will be made between the estimated proportions of E-antigen positive and proportion with detectable HBV viral loads and assumptions of these two parameters from the study design, as these each relate to statistical power for the primary efficacy outcome.

Additional interim reviews by the DSMB will be triggered if any of the following conditions are met: at least 15% of women in the substudy have either grade 3 or higher liver function test results (AST or ALT) or HBV flares; or at least a 10 percentage point difference between any two randomized arms for women experiencing either grade 3 or higher liver function test results or HBV flares.

Additionally, at each time the main study is reviewed by the DSMB, there will be a parallel interim review of the HBV substudy to contain information on accrual, study conduct and monitoring, and safety. Any HBV-specific laboratory results (other than those referenced above) will be presented only if available, which is not planned due to the batched and retrospective nature of the plan for laboratory testing for HBV outcomes.

6.6 Analysis (HBV Substudy)

6.61 Primary Endpoint Analyses

Primary Endpoint Calculation
The distribution of changes in $\log_{10}$ HBV DNA viral load from baseline to Week 8 (antepartum) will be summarized within each group (mean, sd, and 95% confidence interval). For the primary analysis, levels at Week 8 which are below the limit of detection will be set equal to the limit of detection (and secondary analyses will use methods to estimate changes that incorporate the left censoring information). As noted in the definition of the primary endpoint, only those women with detectable HBV DNA viral load at baseline will have the primary endpoint calculated. While this represents an analysis that does not include all randomized women within each group, randomized allocation should provide balance (on average) for pre-randomization factors such as baseline HBV DNA levels. Analyses will investigate the impact of missing information, specifically, how chance imbalance of baseline HBV DNA viral load detectability between groups might induce selection bias for the observation of the primary efficacy endpoint. In addition, among the subgroup of women who were undetectable at baseline, the distribution of Week 8 DNA viral loads will also be described (specifically % remaining undetectable versus % observed) overall, and by arm.

Primary Endpoint Comparison
The mean difference between groups will be estimated along with a corresponding 2-sided, 95% confidence interval (using normality assumptions). The two groups (B versus C) will be compared for this primary endpoint (whether the mean difference in changes from baseline to Week 8 is significantly different from one another) using a Wilcoxon Rank Sum (i.e. non parametric) test.

Secondary Comparison of Primary Endpoint
A secondary analysis comparison of the primary endpoint will include Arm A and will also utilize trend tests to explore a "dose effect" defined by the number of antepartum anti-HBV drugs (i.e. with a restricted

alternative compared to the omnibus test of all groups equal versus some group(s) different from other(s)), in addition to unrestricted (omnibus) alternatives for hypothesis testing.

6.62 Secondary Endpoint Analyses

There are four general types of endpoints in the substudy: antepartum, postpartum, during anti-HBV therapy (which may include antepartum and postpartum follow-up), and post-cessation (of HBV therapy). The relevant groups for comparison and summary of these endpoints differ for each type of endpoint/outcome.

Antepartum endpoints: Groups will be defined by antepartum randomization (Arms A, B and C)

Postpartum endpoints: Groups will be defined depending on the timing of the endpoint evaluation.

1) HBV vertical transmission endpoint: because there is no risk of transmission of HBV via breastfeeding, groups will be defined by AP randomization: 3 groups defined by AP randomization

2) Maternal HBV drug resistance and HBV DNA viral load levels
   a. At L&D, groups compared will be defined by AP randomization
   b. Primary: For 1-4 yrs PP, the primary comparison will be defined only by the postpartum randomizations (resulting in 3 groups: no HAART pp, only HAART during BF, continuous HAART)
   c. Secondary: F1-4 years PP, groups will be defined by all randomizations, though these data may be too sparse for comparisons, and groups may be combined based upon similar ARV usage patterns

During anti-HBV ARV therapy endpoint: Groups will be defined by Antepartum Component randomization (Arms A, B and C)

Post–HBV therapy cessation endpoints: There are two times structured by the main study design when women may be randomized (or assigned based upon the Antepartum Component randomization) to stop the triple ARV HIV prophylaxis and therefore also stop anti-HBV ARVs: following delivery, and after the breastfeeding period ends. Therefore, the primary comparison groups for post-HBV therapy endpoints will be defined based upon groups defined by those study defined allocations to group. However, it is anticipated that the groups available for comparison from the randomization after the breastfeeding period ends may be very small (e.g., estimated to be no more than 69 for HAART and 69 for no HAART). Additionally, there are other times (e.g., Steps 2 and 3) when women may meet criteria specified in the main study protocol (e.g., toxicity or intolerance, or regimen failure), for changing the triple ARV regimen in such a way that anti-HBV ARVs are discontinued outside a randomization or assignment indicated by the main study design. Therefore, to gain as much power as possible to explore for associations between HBV responses post-therapy and HBV treatment history, in secondary analyses, all women stopping anti-HBV ARVs will be included, and the association with anti-HBV exposure will be explored by defining covariates that express the potency (e.g., number of anti-HBV drugs) and length of exposure (and the interaction between potency and length of exposure). Groups based upon similar HBV treatment history will be formulated, but may not necessarily reflect the randomizations or study treatment assignments.

Dichotomous endpoints (e.g., vertical transmission, presence of anemia, presence of HBV drug resistance, presence of hepatotoxicity) will be summarized by estimating within group proportions and associated confidence intervals, using binomial distribution. Hypothesis testing among groups will use Fisher's exact test (or Fisher-Freeman-Halton extension for more than 2 groups). Comparisons including adjustment for covariates will utilize multivariable logistic regression.

Continuous endpoints (e.g., changes in HgB levels, changes in HBV DNA viral load) will be summarized and compared in a manner similar to the primary study endpoint, but will utilize groups as indicated above.

Change in HBV serology will be summarized with contingency tables and compared among groups using chi-square tests.

For post-cessation endpoints, longitudinal methods to incorporate responses correlated over time within participant will be used (and can incorporate both categorical and continuous endpoint measurements).

A full analysis plan will be developed prior initiating any analyses. This plan will include more extensive details of administrative analyses, and primary and secondary analyses, and will be reviewed and approved by the substudy team.

7.0 DATA COLLECTION AND MONITORING AND ADVERSE EVENT REPORTING (HBV SUBSTUDY)

Data collection and adverse experiences reporting will be performed via the main studies (1077BF and 1077FF).

8.0 REFERENCES (HBV Substudy)

(1) Law WP, Duncombe CJ, Mahanontharit A, et al. Impact of viral hepatitis co-infection on response to antiretroviral therapy and HIV disease progression in the HIV-NAT cohort. *Aids*. 2004;18(8):1169-77.

(2) Matee MI, Magesa PM, Lyamuya EF. Seroprevalence of human immunodeficiency virus, hepatitis B and C viruses and syphilis infections among blood donors at the Muhimbili National Hospital in Dar es Salaam, Tanzania. *BMC Public Health*. 2006;6:21.

(3) Sutcliffe S, Taha TE, Kumwenda NI, Taylor E, Liomba GN. HIV-1 prevalence and herpes simplex virus 2, hepatitis C virus, and hepatitis B virus infections among male workers at a sugar estate in Malawi. *J Acquir Immune Defic Syndr*. 2002;31(1):90-7.

(4) Belongia EA CJ, Gareen IF, Grem JL, Inadomi JM, Kern ER, McHugh JA, Petersen GM, Rein MF, Sorrell MF, Strader DB, Trotter HT. National Institutes of Health Consensus Development Conference Statement: management of hepatitis B. *Ann Intern Med*. 2009;150:104-10.

(5) Hammer SM, Eron JJ, Jr., Reiss P, et al. Antiretroviral treatment of adult HIV infection: 2008 recommendations of the International AIDS Society-USA panel. *Jama*. 2008;300(5):555-70.

(6) Ip HM, Lelie PN, Wong VC, Kuhns MC, Reesink HW. Prevention of hepatitis B virus carrier state in infants according to maternal serum levels of HBV DNA. *Lancet*. 1989;1(8635):406-10.

(7) Soderstrom A, Norkrans G, Lindh M. Hepatitis B virus DNA during pregnancy and post partum: aspects on vertical transmission. *Scand J Infect Dis*. 2003;35(11-12):814-9.

(8) Lee SD, Lo KJ, Wu JC, et al. Prevention of maternal-infant hepatitis B virus transmission by immunization: the role of serum hepatitis B virus DNA. *Hepatology*. 1986;6(3):369-73.

(9) Xu WM, Cui YT, Wang L, et al. Lamivudine in late pregnancy to prevent perinatal transmission of hepatitis B virus infection: a multicentre, randomized, double-blind, placebo-controlled study. *J Viral Hepat*. 2009;16(2):94-103.

(10) Rouet F, Chaix ML, Inwoley A, et al. HBV and HCV prevalence and viraemia in HIV-positive and HIV-negative pregnant women in Abidjan, Cote d'Ivoire: The ANRS 1236 study. *J Med Virol*. 2004;74(1):34-40.

(11) Colin JF, Cazals-Hatem D, Loriot MA, et al. Influence of human immunodeficiency virus infection on chronic hepatitis B in homosexual men. *Hepatology*. 1999;29(4):1306-10.

(12) Van Nunen AB, De Man RA, Heijtink RA, Niesters HGM, Schalm SW. Lamivudine in the last 4 weeks of pregnancy to prevent perinatal transmission in highly viremic chronic hepatitis B patients [1]. *Journal of Hepatology*. 2000;32(6):1040-1041.

(13) van Zonneveld M, van Nunen AB, Niesters HG, de Man RA, Schalm SW, Janssen HL. Lamivudine treatment during pregnancy to prevent perinatal transmission of hepatitis B virus infection. *J Viral Hepat*. 2003;10(4):294-7.

(14) Soriano V, Puoti M, Peters M, et al. Care of HIV patients with chronic hepatitis B: updated recommendations from the HIV-Hepatitis B Virus International Panel. *AIDS*. 2008;22(12):1399-410.

(15) de Vries-Sluijs TE, van der Eijk AA, Hansen BE, Osterhaus AD, de Man RA, van der Ende ME. Wild type and YMDD variant of hepatitis B virus: no difference in viral kinetics on lamivudine/tenofovir therapy in HIV-HBV co-infected patients. *J Clin Virol*. 2006;36(1):60-3.
(16) Lewin S, Ribeiro RM, Avihingsanon A, et al. Viral dynamics of hepatitis B virus DNA in human immunodeficiency virus-1-hepatitis B virus coinfected individuals: Similar effectiveness of lamivudine, tenofovir, or combination therapy. *Hepatology*. 2008.
(17) Keeffe EB, Dieterich DT, Han SH, et al. A treatment algorithm for the management of chronic hepatitis B virus infection in the United States: 2008 update. *Clin Gastroenterol Hepatol*. 2008;6(12):1315-41
(18) Keeffe EB, Dieterich DT, Han SH, et al. A treatment algorithm for the management of chronic hepatitis B virus infection in the United States. *Clin Gastroenterol Hepatol*. 2004;2(2):87-106.
(19) Perinatal HIV Guidelines Working Group. Public Health Service Task Force Recommendations for Use of Antiretroviral Drugs in Pregnant HIV-Infected Women for Maternal Health and Interventions to Reduce Perinatal HIV Transmission in the United States. 2008:pp 1-98.
(20) Okada K, Kamiyama I, Inomata M, Imai M, Miyakawa Y. e antigen and anti-e in the serum of asymptomatic carrier mothers as indicators of positive and negative transmission of hepatitis B virus to their infants. *N Engl J Med*. 1976;294(14):746-9.
(21) Beasley RP, Trepo C, Stevens CE, Szmuness W. The e antigen and vertical transmission of hepatitis B surface antigen. *Am J Epidemiol*. 1977;105(2):94-8.
(22) Wong VC, Ip HM, Reesink HW, et al. Prevention of the HBsAg carrier state in newborn infants of mothers who are chronic carriers of HBsAg and HBeAg by administration of hepatitis-B vaccine and hepatitis-B immunoglobulin. Double-blind randomised placebo-controlled study. *Lancet*. 1984;1(8383):921-6.
(23) Stevens CE, Toy PT, Tong MJ, et al. Perinatal hepatitis B virus transmission in the United States. Prevention by passive-active immunization. *Jama*. 1985;253(12):1740-5.
(24) Lee C, Gong Y, Brok J, Boxall EH, Gluud C. Effect of hepatitis B immunisation in newborn infants of mothers positive for hepatitis B surface antigen: systematic review and meta-analysis. *Bmj*. 2006;332(7537):328-36.
(25) Xu W, Cui Y, Wang L, et al. Lamivudine in late pregnancy to prevent perinatal transmission of hepatitis B virus infection: a multicentre, randomized, double-blind, placebo-controlled study. *Journal of Viral Hepatitis*. 2009;16(2):94-103.
(26) Hill JB, Sheffield JS, Kim MJ, Alexander JM, Sercely B, Wendel GD. Risk of hepatitis B transmission in breast-fed infants of chronic hepatitis B carriers. *Obstet Gynecol*. 2002;99(6):1049-52.
(27) Lockman S, Shapiro RL, Smeaton LM, et al. Response to antiretroviral therapy after a single, peripartum dose of nevirapine. *N Engl J Med*. 2007;356(2):135-47.
(28) Lee YS SD, Lim YS, et al. Emergence of rtA181V/T and rtN236T mutations after 48 weeks of adefovir dipivoxil therapy in patients with lamivudine-resistant chronic hepatitis B [abstract]. *Hepatology*. 2005;42(Suppl 1):578A.
(29) Colonno RJ RR, Pokornowski K, et al. Assessment at three years shows high barrier to resistance is maintained in entecavir-treated nucleoside naïve patients while resistance emergence increases over time in lamivudine refractory patients [abstract]. *Hepatology*. 2006;44((Suppl 1)):229A-230A.
(30) Lok AS FS, Han SH, et al. Virological response and resistance to adefovir (ADV) therapy in liver transplant (OLT) patients [abstract]. *Hepatology*. 2005;42((Suppl 1)):232A.
(31) de Vries-Sluijs TE, Reijnders JG, Hansen BE, et al. Long-term therapy with tenofovir is effective for patients co-infected with human immunodeficiency virus and hepatitis B virus. *Gastroenterology*. 2010;139:1934-41.
(32) Matthews GV, Bartholomeusz A, Locarnini S, et al. Characteristics of drug resistant HBV in an international collaborative study of HIV-HBV-infected individuals on extended lamivudine therapy. *Aids*. 2006;20(6):863-70.
(33) Lok AS, Hussain M, Cursano C, et al. Evolution of hepatitis B virus polymerase gene mutations in hepatitis B e antigen-negative patients receiving lamivudine therapy. *Hepatology*. 2000;32(5):1145-53.
(34) Bruno R, Sacchi P, Malfitano A, Filice G. YMDD-mutant HBV strain as a cause of liver failure in an HIV-infected patient. *Gastroenterology*. 2001;121(4):1027-8.
(35) Bonacini M, Kurz A, Locarnini S, Ayres A, Gibbs C. Fulminant hepatitis B due to a lamivudine-resistant mutant of HBV in a patient coinfected with HIV. *Gastroenterology*. 2002;122(1):244-5.

(36) Scholl TO. Iron status during pregnancy: setting the stage for mother and infant. *Am J Clin Nutr*. 2005;81(5):1218S-1222S.
(37) Rasmussen K. Is There a Causal Relationship between Iron Deficiency or Iron-Deficiency Anemia and Weight at Birth, Length of Gestation and Perinatal Mortality? *J Nutr*. 2001;131(2S-2):590S-601S; discussion 601S-603S.
(38) Dabis F, Msellati P, Meda N, et al. 6-month efficacy, tolerance, and acceptability of a short regimen of oral zidovudine to reduce vertical transmission of HIV in breastfed children in Cote d'Ivoire and Burkina Faso: a double-blind placebo-controlled multicentre trial. DITRAME Study Group. DIminution de la Transmission Mere-Enfant. *Lancet*. 1999;353(9155):786-92.
(39) Wiktor SZ, Ekpini E, Karon JM, et al. Short-course oral zidovudine for prevention of mother-to-child transmission of HIV-1 in Abidjan, Cote d'Ivoire: a randomised trial. *Lancet*. 1999;353(9155):781-5.
(40) Shaffer N, Chuachoowong R, Mock PA, et al. Short-course zidovudine for perinatal HIV-1 transmission in Bangkok, Thailand: a randomised controlled trial. Bangkok Collaborative Perinatal HIV Transmission Study Group. *Lancet*. 1999;353(9155):773-80.
(41) Jamisse L, Balkus J, Hitti J, et al. Antiretroviral-associated toxicity among HIV-1-seropositive pregnant women in Mozambique receiving nevirapine-based regimens. *J Acquir Immune Defic Syndr*. 2007;44(4):371-6.
(42) Tuomala RE, Watts DH, Li D, et al. Improved obstetric outcomes and few maternal toxicities are associated with antiretroviral therapy, including highly active antiretroviral therapy during pregnancy. *J Acquir Immune Defic Syndr*. 2005;38(4):449-73.
(43) Gallant JE, DeJesus E, Arribas JR, et al. Tenofovir DF, emtricitabine, and efavirenz vs. zidovudine, lamivudine, and efavirenz for HIV. *N Engl J Med*. 2006;354(3):251-60.
(44) Nuesch R, Ananworanich J, Srasuebkul P, et al. Interruptions of tenofovir/emtricitabine-based antiretroviral therapy in patients with HIV/hepatitis B virus co-infection. *Aids*. 2008;22(1):152-4.
(45) Altfeld M, Rockstroh JK, Addo M, et al. Reactivation of hepatitis B in a long-term anti-HBs-positive patient with AIDS following lamivudine withdrawal. *J Hepatol*. 1998;29(2):306-9.
