# Supplementary material for: The impact of short term Antiretroviral Therapy (ART) interruptions on longer term maternal health outcomes—A randomized clinical trial
Source: PLoS One. 2020 Jan 30;15(1):e0228003. doi: 10.1371/journal.pone.0228003 (PMC6992010; doi:10.1371/journal.pone.0228003)
Supplement: S1 File — (DOCX) [file pone.0228003.s002.docx]

Table 1: Sensitivity analysis comparing the prespecified log-rank test to a post-hoc permutation test for the primary outcome of AIDS defining illness or death.

|  | **P-Value** | |
| --- | --- | --- |
| **Comparison** | **Log-Rank Test** | **Permutation Test^1^** |
| Antepartum Only | 0.79 | 0.19 |
| Postpartum Only | 0.75 | 0.31 |

^1^100,000 permutations were created consistent with the stratification factors and block sizes.
